# Decomposition and Growth Pathways for Ammonium Nitrate Clusters and Nanoparticles

**DOI:** 10.1021/acs.jpca.4c04630

**Published:** 2024-10-14

**Authors:** Ubaidullah S. Hassan, Miguel A. Amat, Robert Q. Topper

**Affiliations:** Department of Chemistry, Albert Nerken School of Engineering, The Cooper Union for the Advancement of Science and Art, 41 Cooper Square, New York, New York 10003, United States

## Abstract

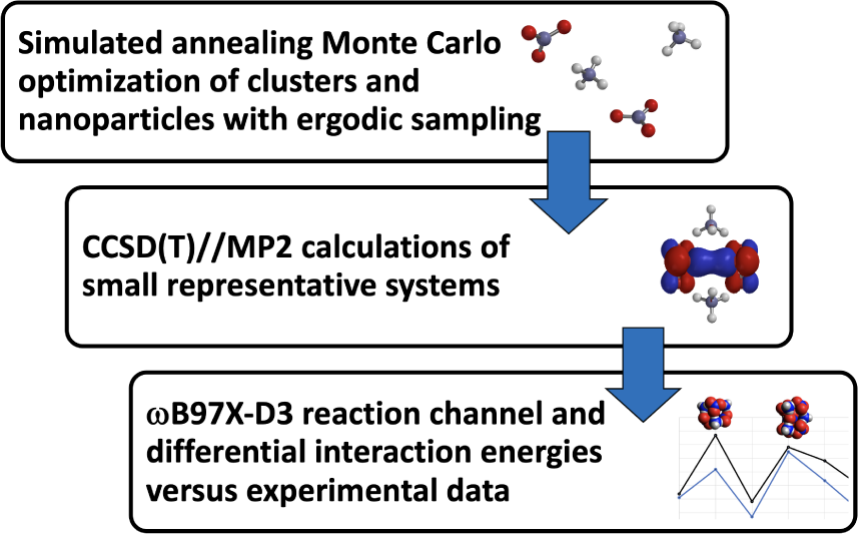

Understanding the formation and decomposition mechanisms of aerosolized ammonium nitrate species will lead to improvements in modeling the thermodynamics and kinetics of aerosol haze formation. Studying the sputtered mass spectra of cation and anion ammonium nitrate clusters can provide insights as to which growth and evaporation pathways are favored in the earliest stages of nucleation and thereby guide the development and use of accurate models for intermolecular forces for these systems. Simulated annealing Monte Carlo optimization followed by density functional theory optimizations can be used reliably to predict minimum-energy structures and interaction energies for the cation and anion clusters observed in mass spectra as well as for neutral nanoparticles. A combination of translational and rotational mag-walking and sawtooth simulated annealing methods was used to find optimum structures of the various heterogeneous clusters identifiable in the mass spectra. Following these optimizations with ωB97X-D3 density functional theory calculations made it possible to rationalize the pattern of peaks in the mass spectra through computation of the binding energies of clusters involved in various growth and dissociation pathways. Testing these calculations against CCSD(T) and MP2 predictions of the structures and binding energies for small clusters demonstrates the accuracy of the chosen model chemistry. For the first time, the peaks corresponding with all detectable species in both the positive and negative ion mass spectra of ammonium nitrate are identified with their corresponding structures. Thermodynamic control of particle growth and decomposition of ions due to loss of ammonia or nitric acid molecules is indicated. Structures and interaction energies for larger (*NH*_4_*NO*_3_)*_n_* nanoparticles are also presented, including the prediction of new particle morphologies with trigonal pyramidal character.

## Introduction

The prevalence of haze in urban environments due to atmospheric pollution and the consequent formation of aerosols reduces visibility and may lead to increases in the incidence of asthma, allergies, and cancer.^1,2^ The formation of aerosols under different conditions must be accounted for in assessments and modeling of global climate change. Aerosols mainly affect global climate through either directly absorbing and scattering solar radiation or acting as nuclei for cloud condensation.^3^ The process of new aerosol particle formation is one of the least understood aspects of aerosol composition and growth.^4,5^ Ammonium nitrate is one of the most common atmospheric aerosol constituents,^6^ with one study showing that it made up an average of 40% by mass of all PM_2.5_ in Utah and up to 70% during persistent cold air pool events.^7^ The major trace gases nitric acid and ammonia are precursors to particles of ammonium nitrate. Nitric acid in the atmosphere is generally derived from nitric oxide from combustion, while trace ammonia originates mostly from fertilizers and animal waste.^8,9^

The decomposition of solid ammonium nitrate is also of great interest and importance, as a number of disastrous incidents involving its handling and storage have occurred over the last century. One of these occurred in 2020 in Beirut, and has been referred to as “the largest non-nuclear blast in modern history.”^10^ For some time it was presumed that under ideal, dry conditions *NH*_4_*NO*_3_(*s*) undergoes low-to-warm-temperature decomposition primarily to *NH*_3_(*g*) and *HN*_3_(*g*), accompanied by a near-negligible (irreversible) amount of direct decomposition to *N*_2_*O* and *H*_2_*O*.^11,12^ However, a number of studies predicted the possibility of the formation of a monomer hydrogen-bonded species (*NH*_3_–*HNO*_3_), as well as larger, ionic nanoparticles of the form (*NH*_4_*NO*_3_)*_n_*,^13−15^ Experimental work in 2010 by Hildenbrand, Chandra and co-workers provided direct evidence that *NH*_4_*NO*_3_(*s*) does not decompose solely to ammonia and nitric acid. They showed that at a minimum, the (*NH*_3_–*HNO*_3_)cluster is also formed by thermal decomposition of the solid and is a measurable fraction of the vapor’s equilibrium composition in the range of temperatures studied (313–360 K).^16^ From their experiments, they estimated the standard enthalpy of dissociation into *NH*_3_(*g*) and *HNO*_3_(*g*)to be *D*_298_ = 78 ± 21 kJ/mol and the standard enthalpy of formation for the monomer cluster as  = 259 ± 21 kJ/mol^17^ However, frozen-core CCSD(T)/CBS// MP2/aug-cc-pVTZ calculations by Irikura, which included corrections for anharmonicity and internal rotations, predicted that *D*_298_ = 49 ± 3 kJ/mol and  = 231 ± 3 kJ/mol.^18^ These calculations were verified with multiple consistency checks as well as consistency with other experiments, and they also agree well with other calculations for *D*_0_ (the dissociation energy at 0 K).^13,14^ Our calculations in this paper provide additional support for Irikura’s work. We note that this hydrogen bond is of theoretical interest, as it would be considered unusually strong using the energy criterion suggested by Marechal.^19^

A number of subsequent papers were published on theoretical predictions of the properties of neutral heterogeneous clusters and nanoparticles of *NH*_3_, *HNO*_3_, and .^20,21^ Most recently, Ling et al. carried out a study of particles formed by various relative numbers of *n* ammonia and *m* nitric acid molecules.^22^ Their work, based on the B3LYP-D3/6-311++G(d,p) model chemistry, showed that the greatest stability of uncharged nucleating particles is generally obtained when *m* = *n*.

The present work is focused on the formation, growth, and decomposition of ammonium nitrate clusters and nanoparticles under conditions of zero humidity, with a focus on fully interpreting mass spectral data for ammonium nitrate for the first time at the molecular level. We present a computational study of , , , and  and show that trends in these species’ computed binding energies are consistent with the mass spectra observed by Dunlap and Doyle.^23^ Simulated annealing Monte Carlo methods are used to locate (global) minimum energy structures, and density functional theory calculations using the ωB97X-D3^24^ method are then used to further refine them. Binding energies of the various systems are then calculated and used to interpret experimental data from positive-ion and negative-ion sputtered mass spectra. Calculations of (*NH*_4_*NO*_3_)*_n_* nanoparticles that are significantly larger than those which have previously appeared in the literature are also presented, with evidence presented for notably stable structures (“magic numbers”) at certain particle sizes.

## Methods

Minimum-energy structures for the systems considered here were located using TransRot, an open source simulated annealing Monte Carlo software project for locating minimum-energy structures of aggregate systems.^25^ During annealing, TransRot treats all component molecules as vibrationless rigid bodies, and internal covalent bonds may not be broken or formed during simulations (but this is of course possible in subsequent quantum-mechanics optimizations). The program is compatible with a variety of models for atomic and molecular interaction energies, including the OPLS-AA force field and parameters used in this study.^26−28^

A problem that can arise when searching for the global minimum-energy structure of a nanoparticle is when an algorithm finds and optimizes a local minimum (of higher energy) rather than the globally minimum-energy structure. TransRot addresses these problems by using mag-walking Monte Carlo sampling (MW)^29^ and sawtooth simulated annealing (SSA).^30^ MW modifies ordinary Barker-Watts sampling of rotational motion and Metropolis sampling of translations by assigning a finite probability for a particle to make a significantly larger translational or rotational move at each step of the simulation. This can help positionally trapped or orientationally frustrated particles to get out of a local minimum energy well and help find the global minimum. On the other hand, SSA helps the entire system move to explore more conformations, rather than individual particles. It does so by modifying ordinary simulated annealing, which involves carrying out a simulation at an initial high temperature (*T*_*0*_) and cooling the system monotonically to 0 K. In ordinary simulated annealing, at high temperature the system will be able to access more kinds of configurations, and there is a higher probability of making an uphill move in energy to prevent getting stuck in a local minimum energy well. However, since the cooling happens at a finite rate it is possible for the system to become trapped. To circumvent this, in SSA a series of *N* annealing cycles (“teeth”) are carried out in which the uppermost temperature for the *N*^*th*^ cooling phase is given as
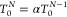
1where α is a fractional parameter chosen by the user. The sequence of repeated heating, cooling, and reheating stages has some resemblance to the metallurgical process of spheroidization, also known as “short cycle” annealing.^31^

Several parameters must be chosen and optimized to achieve success in the MW-SSA calculations. All interaction potential parameters are summarized in the Supporting Information. For the annealing calculations we obtained reliable results using α = 0.725 and 10 teeth, with  and ten temperatures per tooth, with a 10% probability for any given attempted move to use a translational stepsize which is ten times the base value or a rotational stepsize of 360°(the base values chosen were 0.25 Å for translations, 30° for rotations).^25^ The number of Monte Carlo moves carried out at each temperature depended on the system size and ranged from 500,000 for the smallest systems to 6,000,000 for the largest. The MW-SSA calculations show good reproducibility for varying parameter values; in every case, we found that the located structures for each system always landed within the same energy basin. Moreover, these structures always converged to the same result after a final refinement optimization.

For the (*NH*_4_*NO*_3_)*_n_* nanoparticles, the minimum-energy OPLS-AA structure found for each annealed system was also rapidly refined and confirmed using the monotonic version^32,33^ of the Monte Carlo basin hopping (BH) algorithm.^34^ This was done as a confirmatory calculation, to ensure that the MW-SSA structures were well optimized. In this approach, only moves that result in an energy decrease of the locally minimized coordinates are accepted, along with the resulting new set of coordinates. The implementation employed in this work consists of 10 000 compound steps beginning from each annealed structure. Each of these compound steps consists of 100 consecutive translations of, or rigid rotations about, the center of mass. Acceptance ratios for each type of move are set to 0.25 and 0.15, respectively. At each compound step one of these two sequences of moves, i.e., translations and/or rotations, is selected at random, and the moves within each one are carried out through five possible perturbation modes. The choice of modes is motivated in part by the method described by Gonzalez et al.^35^ The possibilities include: (i) all molecules are perturbed independently, (ii) *M* out of the total number of molecules, *N*, are selected randomly as a group and are perturbed in equal measure, (iii) *M* molecules are selected randomly and perturbed independently, (iv) *N* molecules are selected one-at-a-time randomly and perturbed independently, or (v) one molecule is selected randomly and perturbed with a magnitude equal to twice the running value.

At each of the 100 steps, one of the five modes is selected at random and when the selection corresponds to mode (v), its contribution is not accounted into the acceptance ratio statistics. The rotational space for each molecule is probed by the conversion of the corresponding polar spherical angles to qubits, which are then rotated by the action of an operator about the different Cartesian axes, i.e., [Rx, Ry, Rz].^36^ This approach avoids spurious numerical effects found with the sampling of the Euler angles and provides a more natural correspondence to physical angular-valued changes than those associated with the direct sampling of quaternions. The minimization protocol was implemented with the conjugate gradient relaxation algorithm and translations and rotations of, and about, the center of mass, were implemented using the structure of the NO-SQUISH algorithm.^37^ The protocol is iterated until the desired tolerance is satisfied, which in this case corresponds roughly to machine precision. It is emphasized that in this minimization construct the spatial changes imparted on each molecule includes both translations and rotations, irrespective of the type of perturbation selected in the preceding step of the algorithm.

Density functional theory (DFT) methods were used for refining the structures and obtaining electronic energy values for the systems presented here. The ωB97X-D3 method was used for all of the systems presented here, mostly using the Ahlrichs def2 basis sets.^38^ This functional is reported to generally have good accuracy for noncovalent systems and nonbonding interactions.^39^ In addition we tested the ωB97M-V,^40^ M06-2X,^41^ MN12-SX,^42^ and B3LYP-D3^43^ functionals, which also have been reported to perform well for these types of systems and interactions.^44^

All DFT calculations were completed using ORCA 5.0.2^45^ and Q-Chem 6.1.^46^ Geometry optimizations were completed using tight optimization criteria. The DFT calculations were tested on four model systems (described below) for convergence with respect to grid densities. Cross-checks of the different DFT implementations in ORCA and Q-Chem were carried out on the smallest systems; in all cases, the computed interaction energies differed by a negligible amount (less than 0.1 kJ/mol in all cases). We also carried out MP2 and CCSD(T) calculations; these were carried out using Q-Chem and Psi4,^47^ again employing cross-checks to ensure consistency between the two codes. All methods described as “CBS” calculations involve a three-point Helgaker extrapolation of the correlation energy using the aug-cc-pV[TQ5]Z basis sets.^48,49^ We found that single-point CCSD(T) calculations using a slight variation of the “Sherril group gold standard” method available in Psi4, and also employing a frozen core, yielded interaction energies for the two smallest model systems that differed from CCSD(T)/CBS calculations by less than 0.1 kcal/mol. We refer to the method we used simply as an Au-CCSD(T) calculation for the total energy (, which is shown in eqs (2–3):
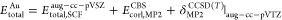
2
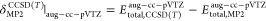
3

In applying eqs 1 and 2), MP2 calculations are carried out to obtain the needed SCF and correlation energies for the aug-cc-pVTZ and aug-cc-pV5Z basis sets; all other required quantities are obtained from a single CCSD(T)/aug-cc-pVTZ calculation. By comparison, the Sherril group gold standard method from Psi4 (“Au std”) for the total energy ( uses a two-point extrapolation and smaller basis sets for the first two terms, as indicated in eq 4:
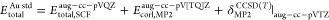
4

Since the Au-CCSD(T) calculations are more numerically tractable full CCSD(T)/CBS for the larger model systems, we present results using the Au-CCSD(T) method for all four model systems.

We first carried out calculations of (*NH*_3_–*HNO*_3_) (our simplest model system) and compared them to previous CCSD(T)/CBS calculations presented by Irikura.^18^ Following his lead, we predicted zero-temperature dissociation energies (*D*_*0*_) for the hydrogen-bonded monomer via the endothermic reaction (R1) shown below.
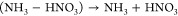
R1

**Figure 1:**
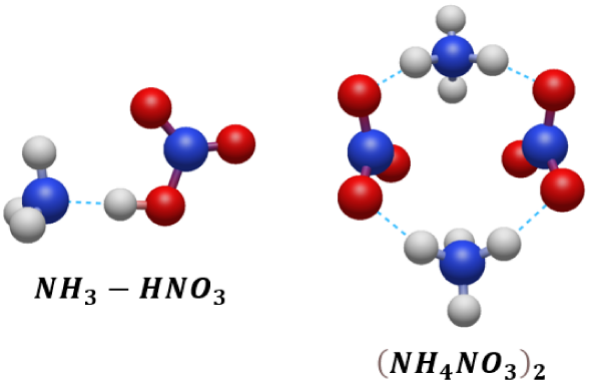
(*NH*_3_–*HNO*_3_)and (*NH*_4_*HNO*_3_)_2_ structures at the ωB97X-D3/def2-SVPD level of theory.

Here *D*_0_=*D+*Δ_r_*E*_*0*_, where *D*_*e*_ is the electronic contribution to the dissociation energy and Δ_r_*E*_*0*_ is the corresponding difference in zero-point energies between products and reactants. The structure of the hydrogen-bonded (*NH*_3_–*HNO*_3_)species is shown in Figure 1.

In Table 1 (see also Table 4 from Irikura^18^ we compare our calculations of *D*_0_ using various methods to other investigators’ work. In general, we find a model to be satisfactory if it agrees with CCSD(T) calculations to within “chemical accuracy,” or about 1 kcal/mol (4 kJ/mol).^50^ Irikura’s CCSD(T)/CBS and MP2/CBS calculations agree to 0.3 kJ mol^–1^. Finite-basis effects are negligible when aug-cc-pVTZ basis sets are used in an MP2 calculation. All of the DFT calculations are within our benchmark window, with the exception of M06-2X (+6 kJ/mol) and B3LYP-D3 (+10 kJ/mol).^22^

**Table 1:** Zero-Temperature Dissociation Energies of (NH_3_– HNO_3_)^a^

Model chemistry	*D*_0_	Reference
CCSD(T)/CBS//MP2/aug-cc-pVTZ^b^	48.6 ± 3	Irikura 2010^18^
MP2/CBS//MP2/aug-cc-pVTZ	48.9	″
MP2/aug-cc-pVTZ	48.9	Present work
MP2/aug-cc-pVTZ//MP2/6-31+G(d)	48.6	″
MP2/6-311++G(d,p)	51.3	Nguyen 1997^13^; Alavi 2002^15^
MP2/6-311++G(d,p) + CP^c^,^d^	46.1	Dmitrova 2000^14^
ωB97X-V/def2-TZVPD	50.8	Present work
ωB97X-D3/def2-TZVPD	51.9	″
B3LYP-D3/6-311++G(d,p)^d^	58.6	Ling 2019^22^
B3LYP/6-311++G(d,p)^d^	51.8	Alavi 2002^15^
M06-2X/def2-TZVPD	55.1	Present work
MN12-SX/def2-TZVPD	48.2	″
ωB97X-D3/def2-TZVPD//ωB97X-D3/def2-SVPD	52.3	″

a *D*_*0*_ is the zero-temperature dissociation energy in kJ/mol. Unless otherwise indicated, all zero-point energy corrections used unscaled harmonic frequencies obtained at the optimization level.

b Includes an anharmonic zero-point energy correction at the MP2/aug-cc-pVTZ level of theory as well as a torsion-corrected partition function.

c Includes a counterpoise correction to the energy for basis set superposition error (BSSE).

d Calculations presented originally did not include a zero-point energy correction, but this has been implemented in the present work.

Next, we investigated which DFT model chemistries would accurately describe the structures and interaction energies of [(*NH*_4_)_2_*NO*_3_]^+^, [*NH*_4_(*NO*_3_)_2_]^−^, and [(*NH*_4_)_2_(*NO*_3_)_2_]. This was done using Au-CCSD(T) and MP2 calculations as benchmarks, We expected that the model requirements might be different than for the monomer because the larger systems all exhibit complete proton transfer between ammonia and nitric acid pairs^15^ and therefore the energetics of their interactions could have different theoretical requirements for accurate treatment. The inclusion of diffuse functions in chosen basis sets is important for accurate interaction energy calculations (since all particles include the nitrate anion), as is the use of triple-ζ basis sets.^39^ We define and calculate the electronic interaction energy (*V*_*e*_*)* which represents the electronic energy change for a gas-phase reaction (hypothetical or otherwise) forming the nanoparticle from its constituents. This can be done unambiguously for the current systems. The usual (products-reactants) difference in electronic energies for the exothermic reactions (R2–R4) define the interaction energies of the three species of interest.
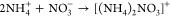
R2
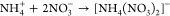
R3
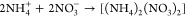
R4

**Figure 2:**
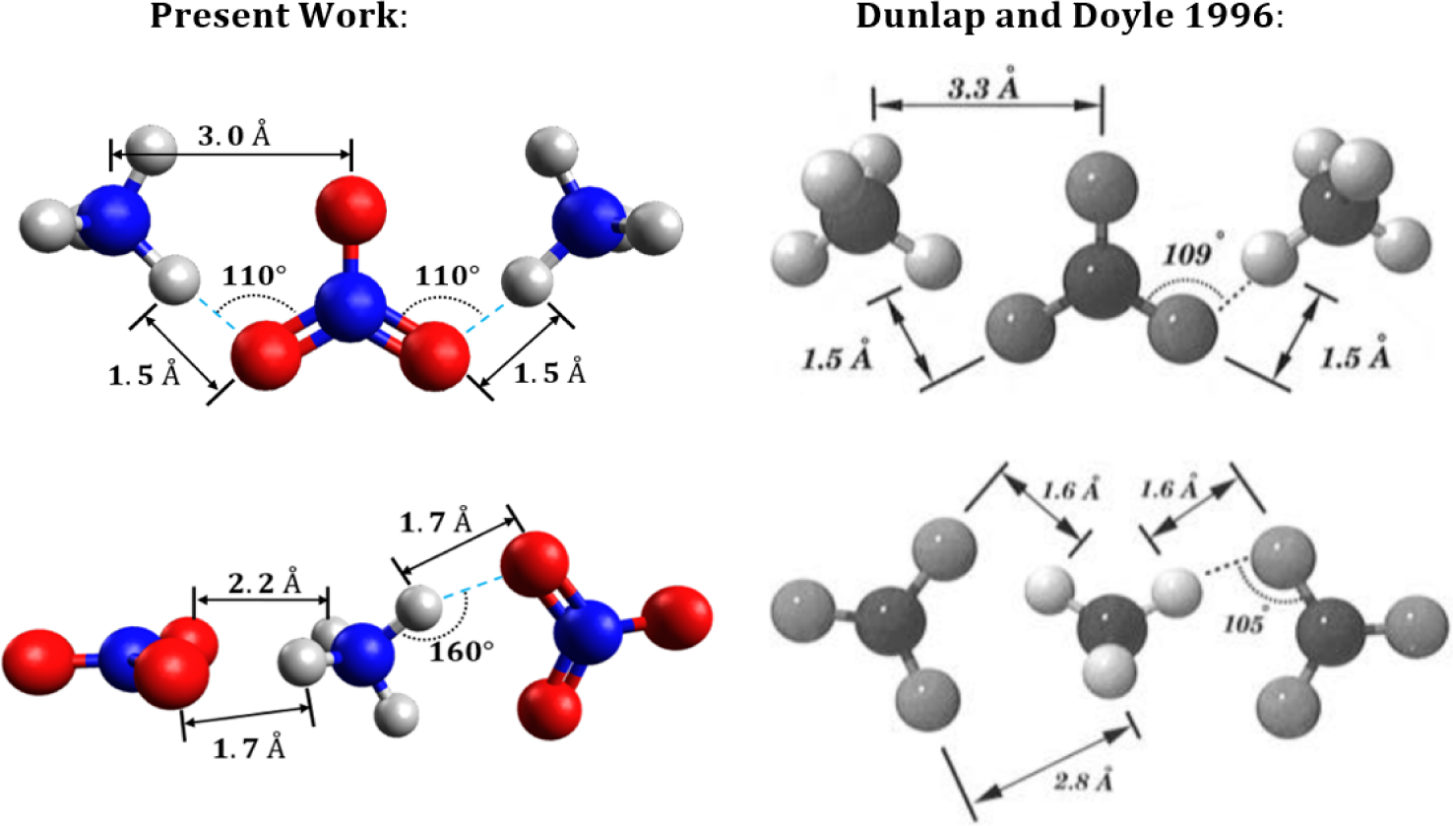
[(*NH*_4_)_2_*NO*_3_]^+^ (upper) and  (lower) structures optimized at the ωB97X-D3/def2-SVPD level of theory and those reported by Dunlap and Doyle^23^ using the BP functional (right). Adapted from 23. Copyright 1996 American Chemical Society.

The MW-SSA method was used to find the minimum-energy structure of each species as previously described (Methods). We refer to these as OPLS/TR calculations, since they use parameters from the OPLS-AA force field to directly estimate the interaction energies for the TransRot annealing calculations. These final structures were then submitted to quantum-mechanical optimizations and energy calculations. We found that the[(*NH*_4_)_2_*NO*_3_]^+^ and  structures are visibly different from those previously reported by Dunlap and Doyle using the BP functional.^23^ In Figure 2 we compare their reported structures to the ones we obtained using an OPLS/TR search followed by ωB97X-D3/def2-SVPD calculations. Both new predictions were further confirmed by MP2/aug-cc-pVTZ optimizations, which yielded very similar structures. These new structures were also obtained by all the other model chemistries presented here. Further investigation of the BP [(*NH*_4_)_2_*NO*_3_]^+^ structure using a BP/6-31G(d) model showed it to be a transition structure with an imaginary frequency. The transition between structures corresponds to rotation of the ammonium ions around the O–H vectors. There is a much larger difference in structures for ; our calculations showed the two nitrates to be perpendicular to one another rather than coplanar. Unfortunately, we were not able to reproduce the structure presented by Dunlap and Doyle using a variety of standard basis sets. For example, tight BP/6-31G(d) optimization of a starting structure similar to the one they reported yields a doubly proton-transferred structure best described as ; this anomalous behavior persisted at the BP/6-311G(d,p) level. We assume that the structure they presented was an artifact of their chosen optimization parameters (for example symmetry may have been enforced, or a loose set of convergence parameters employed).

In Table 2, we compare the predicted interaction energies and the nearest-neighbor interaction distances for the three species formed by (R2–R4) to CCSD(T)/CBS//MP2/aug-cc-pVTZ predictions. For the cation and anion clusters, we found that *Ve* is captured accurately at most of the levels of theory we considered, achieving roughly 1 kcal/mol or better agreement with our Au-CCSD(T) benchmarks. However, there was more differentiation between methods in the case of the neutral [(*NH*_4_)_2_(*NO*_3_)_2_] nanoparticle, for which the MP2 and MN12-SX calculations differed by 3–4 kcal/mol from the Au-CCSD(T) benchmark. We note in passing that the interaction energy of the cation cluster is nearly the same as that of the anion, which is consistent with the fact that similar sputtering energies may be used for either the cation or anion mass spectrum.^23^

**Table 2:** Computed Interaction Energies and Nearest-Neighbor Distance Variations for [(NH_4_)_2_NO_3_]^+^, [(NH_4_)_2_(NO_3_)_2_]^−^, and [(NH_4_)_2_(NO_3_)_2_]^a^

	Cation		Anion		Neutral	
Model chemistry	–Ve	RMSD	–Ve	RMSD	–Ve	RMSD
Au-CCSD(T) //MP2/aug-cc-pVTZ^b^	163.5		164.0		280.3	
MP2/CBS//MP2/aug-cc-pVTZ^b^	163.5		163.6		280.6	
MP2/aug-cc-pVTZ	165.2		164.8		283.5	
ωB97M-V/def2-TZVPD	164.4	0.8%	163.9	1.8%	280.1	1.5%
ωB97X-D3/def2-TZVPD	164.8	0.8%	164.0	0.8%	280.2	0.9%
M06-2X/def2-TZVPD	165.2	1.7%	164.7	0.3%	281.3	1.7%
MN12-SX/def2-TZVPD	162.8	0.7%	162.5	1.5%	277.2	3.4%
B3LYP-D3/6-311++G(d,p)	165.3	1.6%	165.2	2.3%	281.8	2.0%
ωB97X-D3/def2- TZVPD // ωB97X-D3/def2- SVPD	164.8	0.3%	163.9	0.9%	280.2	0.6%

a *V*_*e*_ is the interaction energy in kcal/mol; RMSD is the root-mean-squared percent deviations of nearest-neighbor distances from MP2/aug-cc-pVTZ values.

b The Au-CCSD(T) and MP2/CBS calculations are described in the Methods section.

Since many of the tested model chemistries performed well for predicting interaction energies, we also looked to see which models could predict the root-mean-squared percent deviations of nearest-neighbor distances from MP2/aug-cc-pVTZ predictions (RMSD) by less than 1%. We found that geometry optimization at the ωB97X-D3/def2-SVPD level of theory yielded RMSD values of less than 1%, whereas the other model chemistries often did not perform as well across all cases. Optimization at the ωB97X-D3/def2-TZVPD level also was successful, but the best combination of efficiency and reliability was achieved by the ωB97X-D3/def2-TZVPPD//ωB97X-D3/def2-SVPD model chemistry. The use of a small basis set for geometry optimization and a triple-ζ diffuse basis for the energy made it practical for us to characterize all of the charged systems identified in the mass spectra, as well as to make reliable predictions for the neutral nanoparticles.

## Results

**Figure 3:**
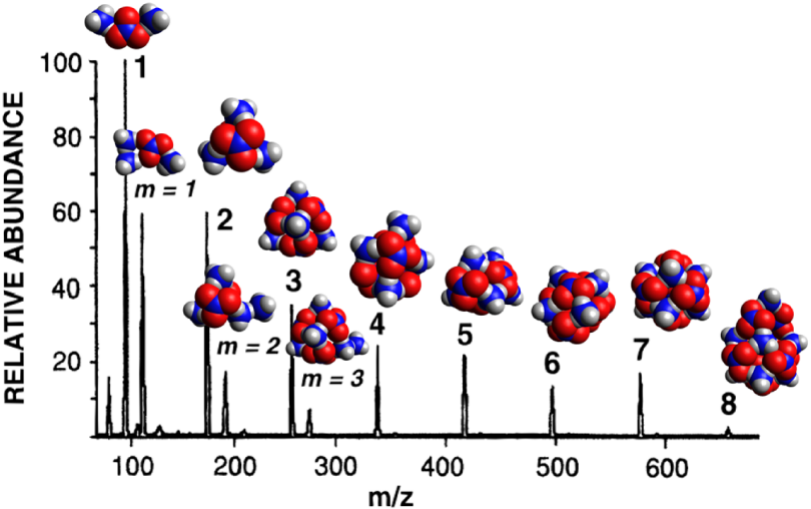
Positive-ion sputtered mass spectrum of ammonium nitrate clusters.^23^ Predicted ωB97X-D3 structures of parent and daughter ions corresponding to the numbered peaks have been added to the original spectrum. Adapted from 23. Copyright 1996 American Chemical Society.

Both positive-ion and negative-ion sputtered mass spectrum (MS) for ammonium nitrate clusters were acquired and presented by Dunlap and Doyle.^23^ In their paper they carried out calculations of the smallest ions corresponding to the two earliest peaks; here we present a complete set of calculations for all observed species. Figure 3 presents these structures along with the positive-ion mass spectrum from their work. The peaks labeled (1–8) are the ammonium nitrate cations (or “n-series”) represented by . The “m-series” peaks are related to the n-series through loss of a molecule of nitric acid, shown by the following equation,
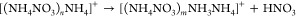
R5where *m = n°–*1. Although loss of nitric acid from the parent cation particles is observed, there is no evidence for loss of ammonia in the mass spectrum:
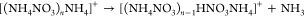
R6

In principle ammonium could also be lost, but this would not be observable in the mass spectrum since the “daughter” species are uncharged:
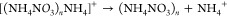
R7

**Figure 4:**
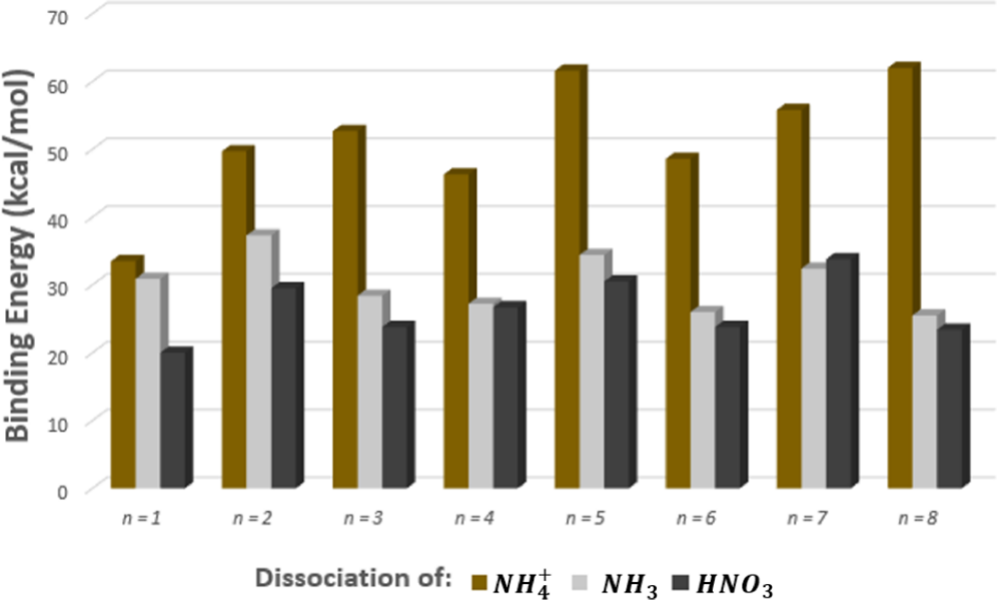
ωB97X-D3 electronic binding energies for different fragmentation channels of .

In order to fully assess the system, OPLS/TR searches followed by ωB97X-D3/def2-TZVP//ωB97X-D3/def2-SVPD calculations were used to predict electronic contributions to the reaction energies of the parent cations with respect to these three dissociation pathways, referring to all of these quantities as “binding energies.” From Figure 4 we see that NH_4_^+^ is generally very difficult to remove, and loss of HNO_3_ is much more facile than loss of NH_3_ for almost all cases of *n*. For *n* = 7, the data suggests that ammonia is more easily evaporated from the nanoparticle than nitric acid. For the region *n* > 4 there is virtually no intensity for the related *m*-series peaks. The drop in parent peak intensity may explain this lack of daughter peaks.Our predicted binding energies for the *n* = 1 parent structure may be compared to the BP calculations presented by Dunlap and Doyle.^23^ Our calculations with respect to the dissociation of this parent to ammonium, ammonia, or nitric acid yielded (33.5, 30.9, 20.0) kcal/mol respectively; Dunlap and Doyle reported calculated binding energies of (32.8, 33.2, 17.6) kcal/mol.

**Figure 5:**
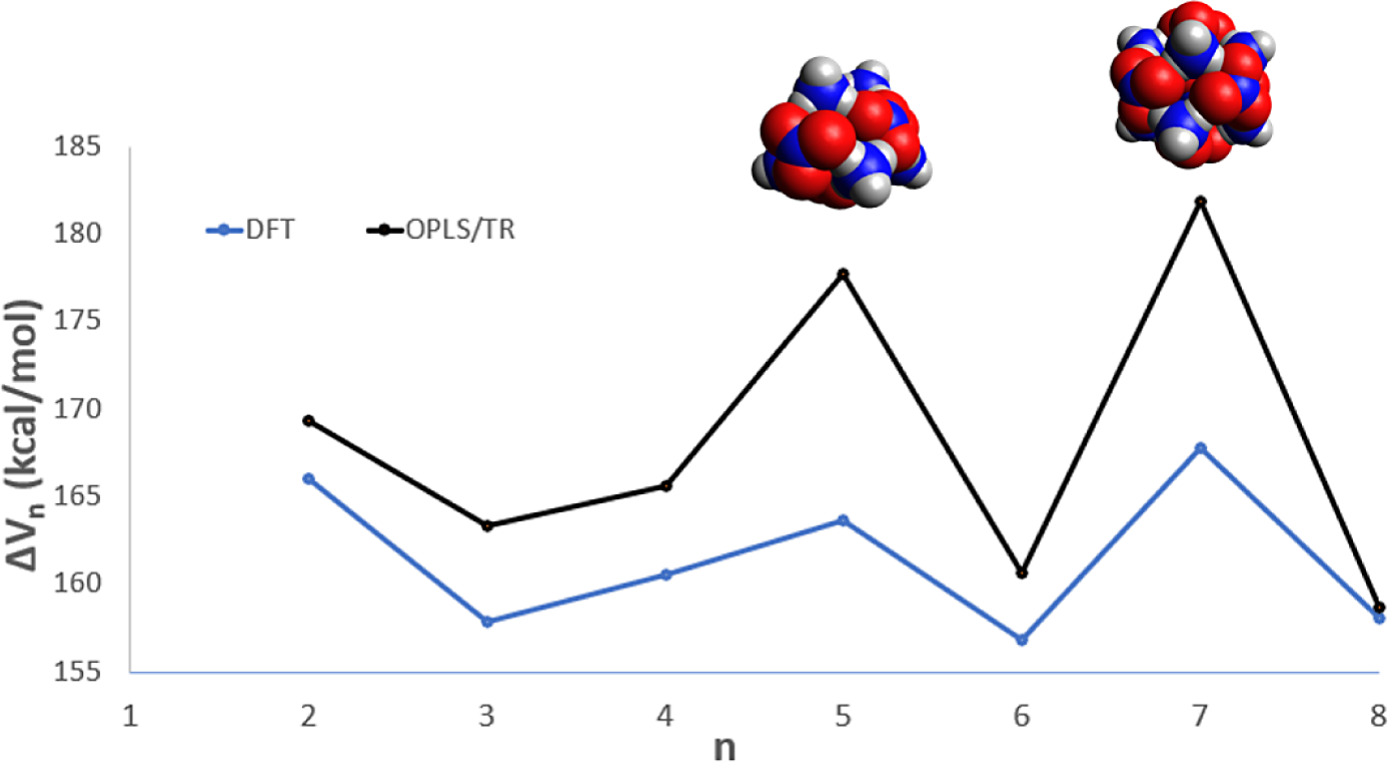
ωB97X-D3 and OPLS/TR differential interaction energies for the parents.

To further analyze the positive-ion MS, in Figure 5 we plot the differential interaction energy (diff-IE), Δ*V*_*n*_, of the parents, calculated according to Δ*V*_*n*_*=V*_*n*–1_*V*_*n*_. A peak in the diff-IE plot predicts that a structure is energetically disfavored to lose or gain a monomer subunit, which may correspond experimentally to a magic number.^51^ From Figure 5 we see that there are peaks in Δ*V*_*n*_ as a function of *n* at *n* = 5 and *n* = 7, also, the *n* = 7 structure has the highest change in interaction energy and therefore is highly stable when formed. Notably, the *n* = 5 cation cluster takes the shape of a triangular prism, and the *n* = 7 structure is a cubic form.

Referring to Figure 3, we see that the *n = 5* peak in the mass spectrum appears to be slightly taller than the trend in successive peak heights would predict from a uniform decline, and the *n* = 7 peak is slightly taller than its neighbors. Repeated experiments with varied sputtering energies and temperatures would be helpful in confirming that these species are in fact unusually stable, as predicted here.

**Figure 6:**
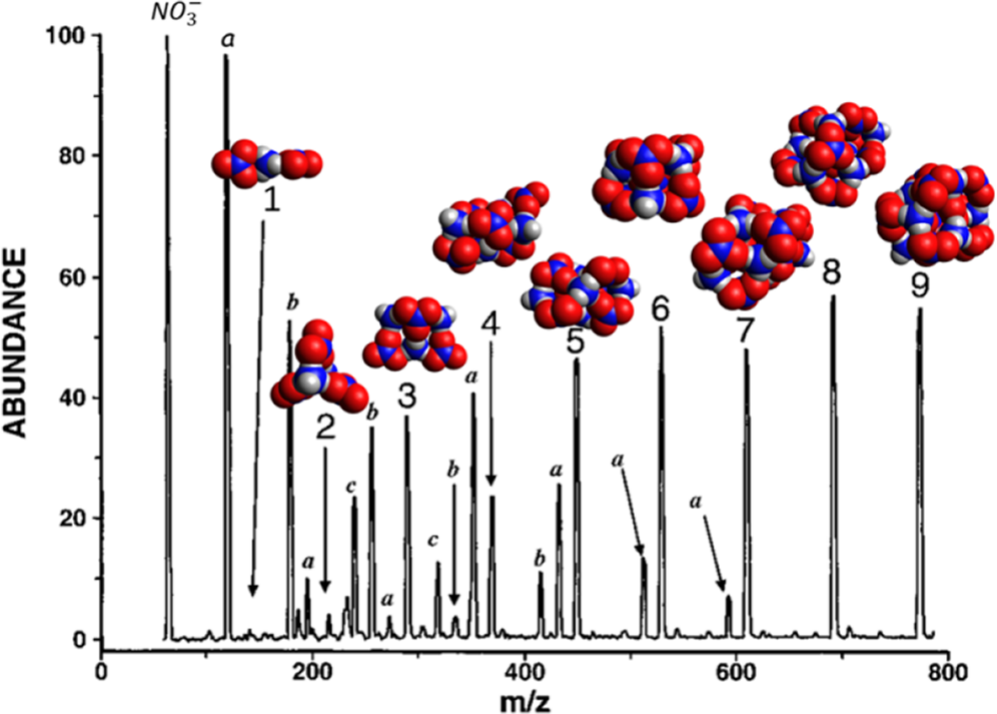
Negative-ion sputtered mass spectrum of ammonium nitrate clusters.^23^ Predicted structures of parent ions corresponding to the numbered peaks have been added to the original spectrum. Adapted from 23. Copyright 1996 American Chemical Society.

We then considered the negative ion sputtered mass spectrum. Figure 6 shows this spectrum along with the structures of the parent ammonium nitrate anions found through our calculations. The peaks labeled (1–9) are the ammonium nitrate anions (or n-series) represented by  The *a* peaks are related to the n-series peaks through the dissociation of one ammonia molecule, while the *b* peaks are related through the dissociation of two ammonias and the *c* peaks are related through the dissociation of three ammonias.

The reactions leading to the *a, b,* and *c* peaks are summarized in the following equations, respectively.
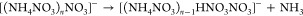
R8
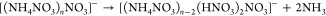
R9
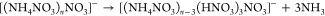
R10

In addition to this complexity, the anion mass spectrum has an initially large peak at *n* = 1, a much smaller peak at *n* = 2, and sizable peaks for *n* > 2, which is notably different than the parents’ peak trends in the cation mass spectrum. Another interesting phenomenon in the mass spectrum is the lack of *c* peaks after *n* = 4, the lack of *b* peaks after *n* = 5, and the lack of *a* peaks after *n* = 7.

As before, OPLS/TR searches were used to find minimum-energy structures for subsequent refinement and computation using the ωB97X-D3 functional. These calculations were then used to calculate dissociation energies of the negative n-series peaks with respect to these dissociation pathways. In analogy to the cation clusters, the possibility of an anion cluster’s dissociation to form nitrate or nitric acid was also considered and is represented by reactions (R11 and R12):
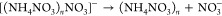
R11
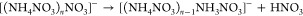
R12

**Figure 7:**
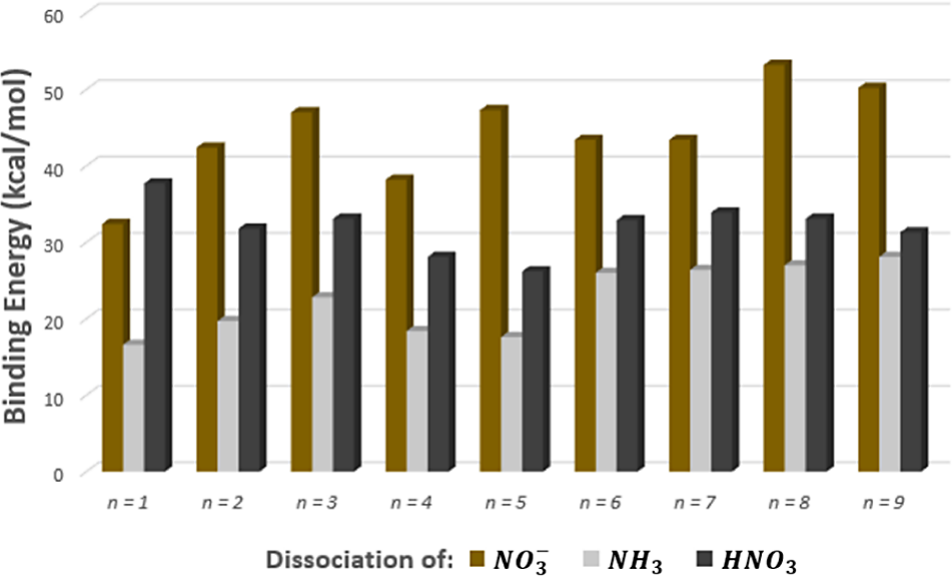
ωB97X-D3 electronic binding energies for different fragmentation channels of  cations.

In Figure 7, which shows binding energies for the anion parent clusters, we see that for all *n* loss of ammonia from  is strongly preferred to loss of nitrate or nitric acid. This explains why in the negative-ion MS we observe that the n-series peaks almost all have a satellite *a* peak in this region.

Again, we compare reaction energies found for the *n* = 1 parent structure to those found by Dunlap and Doyle.^23^ The binding energies found from our calculations with respect to the dissociation of nitrate or ammonia were 32.6 and 16.9 kcal/mol respectively. Using BP calculations, Dunlap and Doyle found binding energies of 32.6 and 15.9 kcal/mol; here, the agreement between methods is noticeably better than was the case for the *n* = 1 cation parent, and the reported structures are similar.

**Figure 8:**
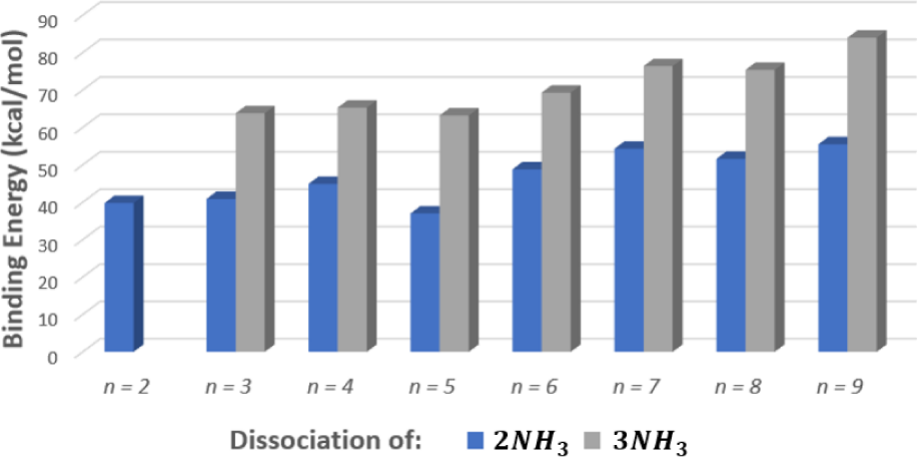
ωB97X-D3 electronic binding energies for fragmentation of  anions by successive ammonia loss, according to reactions (R9,R10).

To gain insight on the presence of the *b* and *c* peaks, we look at the binding energies for the ammonium nitrate anion clusters with respect to the dissociation of either two or three ammonias represented by reactions (R9,R10). In Figure 8 we see that the lowest binding energies are found for dissociation of 2*NH*_3_ from the *n* = 2 and *n* = 5 anion parent. This may be compared to the fact that the mass spectrum from Figure 6 shows a high intensity peak for the *b* peak that is formed from the *n* = 2 fragment, . This supports the favorability of this fragmentation pathway for the *n* = 2 parent anion. Additionally, there is a jump in the binding energies with respect to the dissociation of two ammonias for *n* > 5. This jump helps to explain the lack of *b* peaks after *n* = 5.

**Figure 9:**
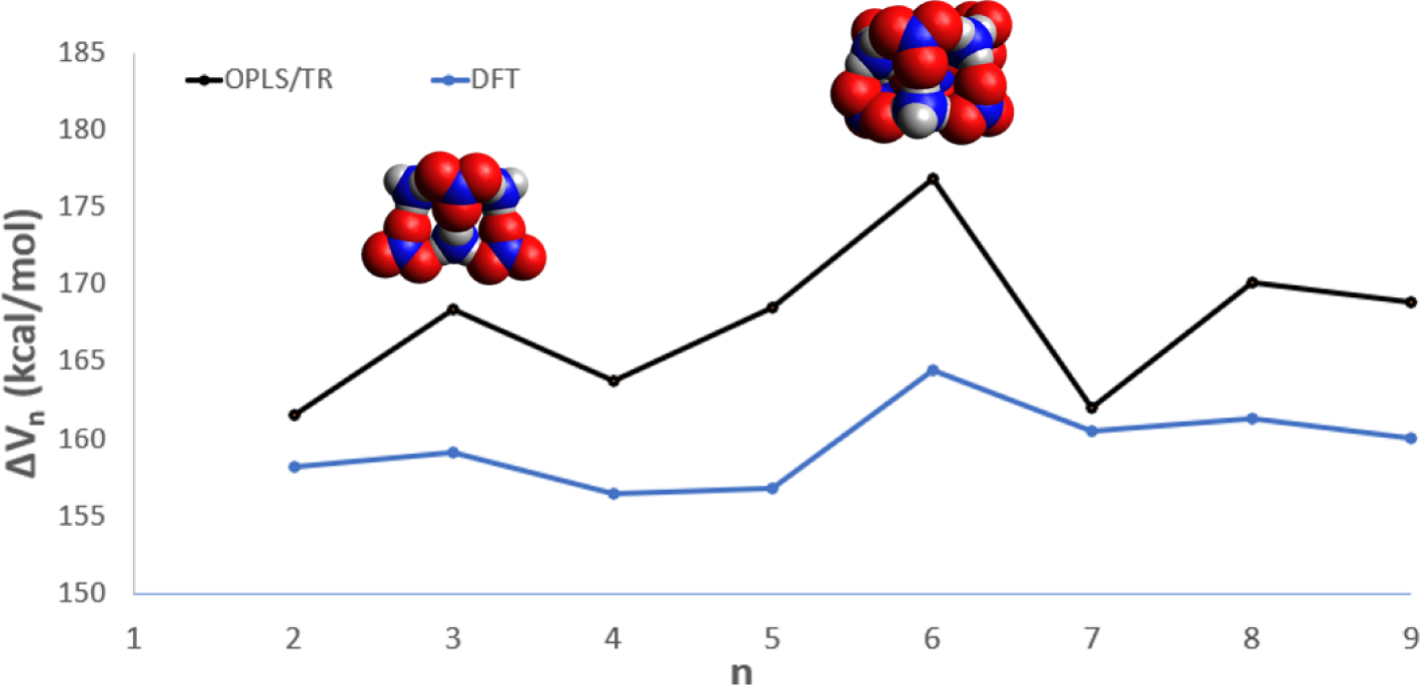
ωB97X-D3 (DFT) and OPLS/TR differential interaction energies for the parents.

A plot of the diff-IE as a function of *n* for the anion parents is shown in Figure 9. We see that there are peaks at *n* = 3 and *n* = 6; moreover, the *n* = 6 system has the highest change in interaction energy. Both are expected to correspond to magic numbers. The *n* = 3 structure can be described with two trapezoid shaped faces and a diamond shaped face, while the *n* = 6 nanoparticle takes on a cube-like shape. Referring to Figure 4, we see that the *n* = 3 and *n* = 6 peaks are taller than peaks for neighboring parent ions, which provides preliminary evidence for their “extra” stability. Again, additional experiments with different sputtering energies could verify these predictions.

**Figure 10:**
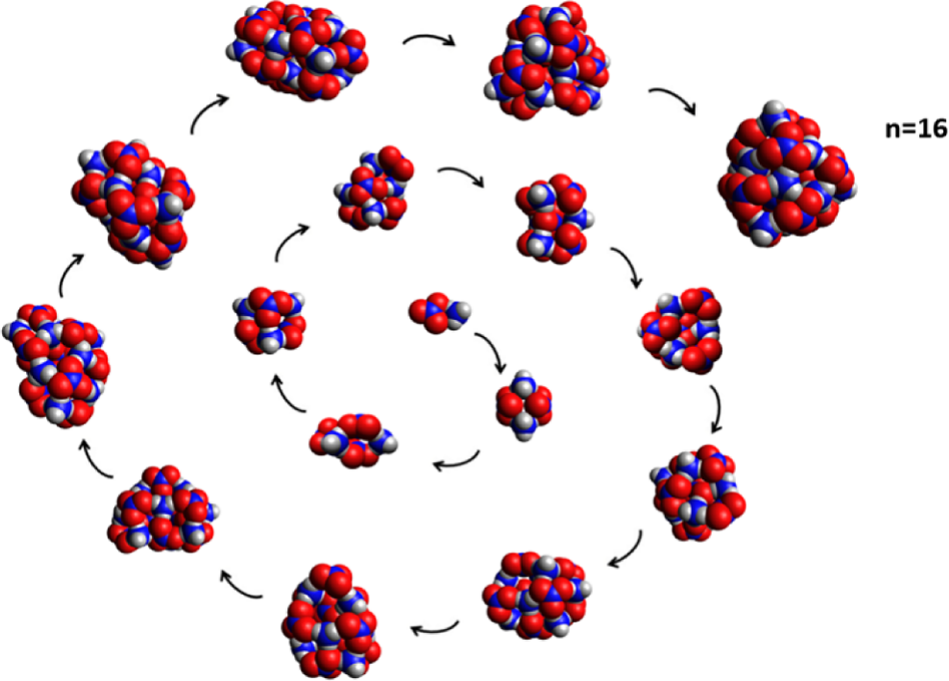
Predicted structures of selected (*NH*_4_*NO*_3_)*_n_* nanoparticles from OPLS/TR calculations followed by ωB97X-D3/def2-SVPD geometry optimization.

**Figure 11:**
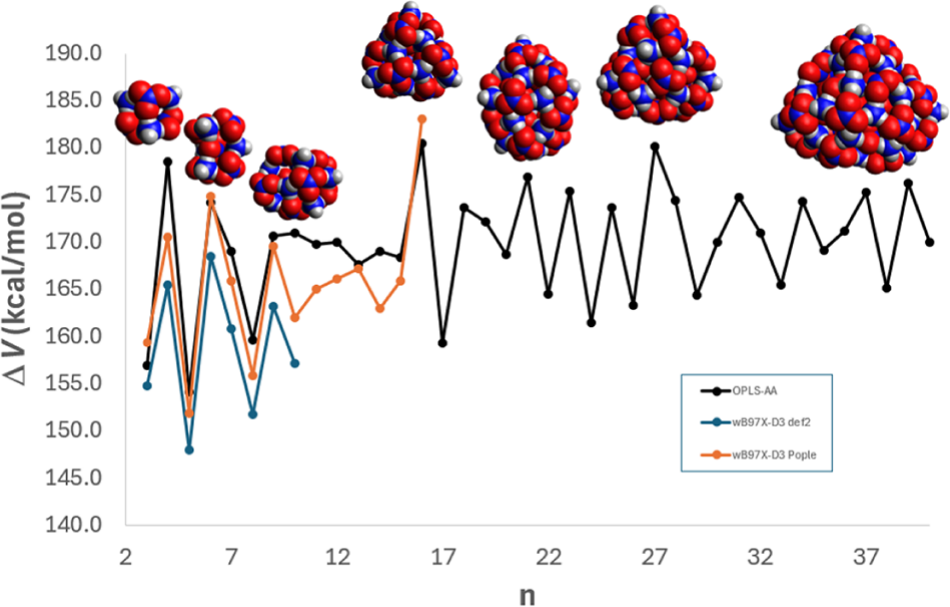
ωB97X-D3 differential interaction energies for (*NH*_4_*NO*_3_)*_n_* from OPLS-AA (black) and ωB97X-D3/def2-TZPD calculations (blue). Also shown: ωB97X-D3 calculations using a 6-311+G(2df,2p)[6-311G(d)] “Pople” basis; see text (orange). The optimized *n* = 4, 6, 9, 16, 21, 27, and 37 structures are shown.

Given the consistency of our calculations with experiment, we then moved with reasonable confidence toward a study of larger, neutral nanoparticles of ammonium nitrate (for which no experimental data is available). This is with the hope of better understanding the earliest stages of growth and decomposition of ammonium nitrate nanoparticles in the air. To this end, OPLS/TR searches were used to find minimum-energy structures which were then refined with subsequent ωB97X-D3/6-31G* optimization. In Figure 10 we show the changes in the structures of ammonium nitrate nanoparticles which occur with the addition of each additional ion pair for (*NH*_4_*NO*_3_)_*n*_,*n =*(2–16). One notable similarity between this system and the ammonium halides is that the *n* = 4 structure is a cube of alternating cation and anion corners; in the case of the ammonium halides, this structure is unusually stable.^52,53^ As discussed later, most of these structures are very different from the cubic structure of bulk ammonium nitrate as well as the cubic and icosahedral structures reported for nanoparticles of ammonium halides.^54^ The diff-IE as a function of size for these structures is shown in Figure 11; the structures are also shown for some of the species for which the diff-IE shows a pronounced peak.

Since the calculations using def2 basis sets proved to be numerically challenging for larger nanoparticles, we also carried out ωB97X-D3/6-311+G (2df,2p)[6-311G(d)]//wB97X-D3/6-31G(d) calculations. We found that this model, which uses much smaller Pople basis sets, captured the same peak positions as if larger basis sets were used and also allowed us to make predictions of peak positions for even larger nanoparticles. Figure 11 shows that despite the differences in energies, the OPLS-AA calculations seem to generally capture the trend of peak locations in the diff-IE when compared to the DFT calculations using def2 or Pople basis sets, with notable deviations for *n* = (10–16).

We observed that for all particles with *n* > 1, and at all levels of theory, no recombination of protons from NH_4_^+^ to NO_3_^–^ was observed following optimization. All of the ammonium cations remained essentially tetrahedral in all clusters, and all nitrate anions remained planar. The nearest-neighbor interactions along intermolecular bond axes are analogous to hydrogen bonds, albeit hydrogen bonds with complete proton transfer.^15^ This particular, fundamental aspect of the structure matches that of all phases of solid ammonium nitrate.^55^ As expected, all three models predict that the *n* = 4 heterocubane structure is quite stable. Conversely, the strong stabilities of the butterfly shaped *n* = 6 structure, the near-triangular-prism *n* = 9 particle, and the near-trigonal pyramidal *n* = 16 species were not anticipated but these are also clearly predicted to be very stable. Under dry conditions, we would expect the (*n* = 4) structure to be a significant contribution to the composition of a dense fog of ammonia and nitric acid vapor; the (*n* = 6, 9, 16) species may be observable as well, depending on the vapor density and conditions.

Considering the structural trends of the larger nanoparticles is of some interest, as they may speak to the general question of when the structure of a nanoparticle might eventually become similar to that of the bulk, solid material. One complication to the analysis is the observation that the OPLS-AA model predicts that for 8 < *n* < 16, Δ*V* is more or less monotonically decreasing while the DFT calculations predict much more pronounced changes in Δ*V*. Since the structures do not change qualitatively following DFT optimization, we tentatively assign this behavior to the fact that the OPLS-AA model cannot account for certain contributions to the interaction energy. In particular, the nitrate ions should exhibit significant π electron delocalization, which may affect the strength of both NH_4_^+^ - NO_3_^–^ and NO_3_^–^ - NO_3_^–^ interactions within the clusters. We have noticed that the locally stable structures with *n* > 15 all have triangular faces, while smaller structures have rectangular or square faces; this represents a significant structural transition. The *n* = 16 structure also shows up as part of the *n* = 21 structure, which is a triangular pyramid with one of its faces “capped” by five ion pairs. The *n* = 27 structure resembles a nonregular triangular pyramid, meaning the faces are not all equilateral triangles like in the *n* = 16 structure. The faces for the *n* = 27 structure seem to be two scalene triangles, one equilateral triangle, and one isosceles triangle. The isosceles triangle face is shown in Figure 11 and has an apex angle of around 70° (measuring with respect to the ammonium’s outermost hydrogen). The *n* = 37 is a flat structure with only one clear triangular face. This face seems to be approximately an isosceles triangle with an apex angle of around 82°.

Bulk ammonium nitrate exists in several solid-state phases, each with unique structures.^55−58^ At atmospheric pressure, phase I and II of ammonium nitrate occur at high temperatures of above 125.2 °C. These phases are characterized by cubic and tetragonal crystal structures, respectively. Phase III is stable between 32.3 and 84.2 °C and adopts a cubic structure. This phase is important due to its relevance in the storage and handling of ammonium nitrate, as it may exist in hotter climates at atmospheric pressure. As temperatures decrease further, ammonium nitrate enters phase IV, which is stable from −18.0 to 32.3 °C. Finally, phase V occurs below −18 °C. Phases III, IV, and V are particularly relevant to our analysis of minimum energy structures of ammonium nitrate nanoparticles as these phases occur at lower temperatures. All three of these phases have orthorhombic crystal structures; none of the patterns in these crystal structures seem to appear in any of the discussed nanoparticles for the size range *n* = (1–40). As noted above, size-dependent variance of nanoparticles from the low-temperature crystal structure was observed in our previous study of ammonium chloride nanoparticles.^54^ However, in that work we were able to see similarities between certain nanoparticles and the high-temperature phase of solid ammonium chloride; ammonium nitrate is apparently even more complex. Since it appears that nanoparticles of ammonium nitrate have different structural patterns than the bulk material, the use of models for nucleation rates and growth which assume that nanoparticles behave in the same manner as the bulk material may be questioned.

## Conclusions

Using simulated annealing calculations designed to find the lowest energy structure for each system of interest followed by density functional theory calculations, we were able to characterize the *m*-series in the positive-ion sputtered mass spectrum (corresponding to loss of nitric acid) and the *a*- series in the negative-ion mass spectrum (corresponding to loss of ammonia) using reaction channel energies. Our analysis helped us to rationalize the energetic tendencies toward loss of NH_3_, HNO_3_, NH_4_^+^ and NO_3_^–^ molecules from the various parent cluster ions as being energetically controlled (as opposed to kinetic control). Additionally, through analysis of the differential interaction energies of the parents we predict that mass spectra at various sputtering energies would show magic numbers for ,  and .

Examination of the growth of neutral ammonium nitrate series in the nanoparticle regime revealed unusually stable structures for (*NH*_4_*NO*_3_)_4_ (as a heterocubane structure), (*NH*_4_*NO*_3_)_6_ (a bilaterally symmetric “butterfly”), and (*NH*_4_*NO*_3_)_16_ (a quasi-trigonal pyramid). We also showed preliminary evidence for magic numbers for larger nanoparticles, including larger pyramidal structures. Although a transition is observed from structures with cubic faces to trigonal forms, there does not seem to be a second transition to one of the bulk structures of *NH*_4_*NO*_3_(*s*)in the size regime we considered.

Although the OPLS/TR calculations were very well suited for locating preliminary structures for subsequent DFT optimization, it was not always able to qualitatively reproduce the trends in differential interaction energies predicted by density functional theory calculations. This tends to indicate that the optimized cluster and nanoparticle systems considered here could be used as benchmarks for the development and critical testing of improved empirical models for the interaction potential, as well as for DFT methods. In future work we anticipate calculations of free energies and nucleation rates, with and without solvent and/or microhydration. Preliminary work has been carried out in our group on solvent effects, using an implicit solvent model to carry out geometry optimizations, free energy calculations, and kinetic modeling, starting from structures starting from gas-phase structures presented in earlier work.^22,59^ These calculations predict that water generally tends to weaken hydrogen bond interactions and favors proton transfer from acid to base for even the smallest particles, and that as the particles grow they become kinetically and thermodynamically less likely to absorb water. The implicit solvent results are highly suggestive, but contributions from microhydration are unlikely to be small or perturbative, especially for small nanoparticles.^60^ For example, previous work by Tao and co-workers showed that in (*NH*_3_···*HNO*_3_)(*H*_2_*O*)*_n_* clusters, at least two water molecules are required to achieve proton transfer from nitric acid to ammonia.^13^ We believe that structural sampling of the kind we have used in this work, followed by quantum calculations, will enable microhydration and bulk solvation to be assessed for larger clusters and in somewhat more detail than has been previously achieved. The application of other structural sampling strategies to this problem^61^ could challenge our predictions and perhaps provide structural and thermodynamic information for even larger nanoparticles.

## Supporting Information Available

Detailed description of optimization parameters. OPLS-AA parameters used in the simulated annealing calculations are provided. Computed interaction energies of (neutral) nanoparticles and cation and anion clusters. The computed energies of all species presented in this work. The coordinates in XYZ format for all ωB97X-D3/def2-SVPD optimized cation and anion clusters and nanoparticles. The optimized OPLS-AA and ωB97X-D3/6-31G(d) structures in XYZ format for the nanoparticles presented in this work (PDF)
Structures of ammonium nitrate(ZIP)
