# Supplementary material for: Decomposition and Growth Pathways for Ammonium Nitrate Clusters and Nanoparticles
Source: J Phys Chem A. 2024 Oct 14;128(42):9184–94. doi: 10.1021/acs.jpca.4c04630 (PMC11514028; doi:10.1021/acs.jpca.4c04630)
Supplement: Supplementary file 1 — jp4c04630_si_001.pdf [file jp4c04630_si_001.pdf]

Supporting Information for:

Decomposition and Growth Pathways for Ammonium Nitrate Clusters and Nanoparticles

Ubaidullah S. Hassan, Miguel A. Amat, and Robert Q. Topper*

Author Affiliations:

Ubaidullah S. Hassan, Department of Chemistry, The Cooper Union for the Advancement of Science and Art, New York NY 10003, United States.

Miguel A. Amat, Department of Chemistry, The Cooper Union for the Advancement of Science and Art, New York NY 10003, United States.

Robert Q. Topper, Department of Chemistry, The Cooper Union for the Advancement of Science and Art, New York NY 10003, United States. Email: topper@cooper.edu; Phone: 212-353-4370.

Overview

The computed interaction energies of all species presented in this work are presented in this document, in kcal/mol. The parameters used to implement the OPLS-AA model in TransRot simulated annealing calculations are also documented here.

In separate files, we also provide ωB97X-D3/def2-SVPD optimized coordinates in XYZ format (Å) for the molecules HNO_3, NO_3^-, NH_3, and NH_4^+ as well as for the cation, anion, and neutral nanoparticle/cluster species presented in this work. We also provide optimized coordinates at the OPLS-AA (2-40) level for the neutral nanoparticles. A list of all tables follows this overview.

Optimization details

The cation and anion clusters were optimized and computed using ORCA 5.0. All of the DFT calculations used ORCA's intrinsic grid generation scheme (DEFGRID2) with adaptive pruning. The structures were optimized using the VERYTIGHTOPT keyword, which uses a gradient tolerance of 3.0 x 10^{-5} au, a RMS gradient tolerance of 8.0 x 10^{-6} au, a distance tolerance of 2.0 x 10^{-4} bohr, an RMS displacement tolerance of 1.0 x 10^{-4} bohr and an energy tolerance of 2.0 x 10^{-7} hartree. Tests against Q-Chem calculations on the smallest representative systems using similar optimization criteria yielded nearly identical interaction energies. We also found insensitivity of these results towards using higher grid densities. All optimizations were verified by normal-mode frequency calculations.

The neutral nanoparticles presented here were optimized and computed using Q-Chem 6.1. Again, the SCF calculations in the geometry optimizations were converged using the Pulay DIIS algorithm. These DFT calculations employed the SG-2 quadrature grid. Calculations on our test set using a larger (99,590) Lebydev grid (without pruning) yielded nearly identical interparticle distances and interaction energies. For calculations using the def2 basis sets, all structures were optimized using a gradient tolerance of 9.5x10^{-5} au, a distance tolerance of 8.0 x 10^{-4} Å and an energy tolerance of 1.0x10^{-7} hartree. All nanoparticle structures presented in this paper were

confirmed to be minima. For nanoparticles with 14 ion pairs or fewer, this was done via normal-mode frequency calculations. The largest nanoparticles (15 or more ion pairs) were verified to be minima using Q-Chem's implementation of the finite-difference Davidson method for optimum characterization, as described by Head-Gordon and coworkers (S.M. Sharada et al., J. Chem. Phys. 140, 164115 (**2014**); see also J. Chem. Phys. 140, 229902 (**2014**)).

List of Tables

This file:

Table S1: OPLS-AA Parameters Used in TransRot Calculations
Table S2: Interaction Energies of Neutral Nanoparticles
Table S3: Interaction Energies of Cation and Anion Clusters
Table S4: Energies of Cation and Anion Clusters and Component Molecules
Table S5: Energies of Neutral Nanoparticles and Component Molecules

Separate archive (zip):

Table S6: Cartesian Coordinates of p=(1-9) $[(NH_4NO_3)_p(NO_3)]^-$: ωB97X-D3/def2-SVPD
Table S7: Cartesian Coordinates of p=(0-7) $[(NH_4NO_3)_p(HNO_3)_2(NO_3)]^-$: ωB97X-D3/def2-SVPD
Table S8: Cartesian Coordinates of p=(0-6) $[(NH_4NO_3)_p(HNO_3)_3(NO_3)]^-$: ωB97X-D3/def2-SVPD
Table S9: Cartesian Coordinates of p=(0-8) $[(NH_4NO_3)_p(HNO_3)(NO_3)]^-$: ωB97X-D3/def2-SVPD
Table S10: Cartesian Coordinates of p=(0-8) $[(NH_4NO_3)_p(NH_3)(NO_3)]^-$: ωB97X-D3/def2-SVPD
Table S11: Cartesian Coordinates of p=(1-8) $[(NH_4NO_3)_p(NH_4)]^+$: ωB97X-D3/def2-SVPD
Table S12: Cartesian Coordinates of p=(0-7) $[(NH_4NO_3)_p(NH_4)(HNO_3)]^+$: ωB97X-D3/def2-SVPD
Table S13: Cartesian Coordinates of p=(0-7) $[(NH_4NO_3)_p(NH_3)(NH_4)]^+$: ωB97X-D3/def2-SVPD
Table S14: Cartesian Coordinates of n=(1-10) $[NH_4NO_3]_n$: ωB97X-D3/def2-SVPD
Table S15: Cartesian Coordinates of n=(2-40) $[NH_4NO_3]_n$: OPLS-AA
Table S16: Cartesian Coordinates of n=(1-16) $[NH_4NO_3]_n$: ωB97X-D3/6-31G(d)

Table S1: OPLS-AA Parameters Used in TransRot Calculations. The structural and OPLS-AA interaction parameters for HNO_3, NH_3, NH_4^+ and NO_3^- which were used in the TransRot calculations (as appropriate) are given below in the code's expected input format. Energies are in kcal/mol , coordinates are in Å, masses are in amu, and partial charges are in atomic charge units. The format used is documented at the TransRot project website on GitHub: https://github.com/steventopper/TransRot . The table below can be directly used as an input parameter file for TransRot.

```
4
NH3    2
N    0.000000   0.000000   0.000000   0.0000   0.0000   1088.092    1741093.866   -1.0200   14.007
H    0.000000  -0.937700  -0.381600   0.0000   0.0000   0.000000    0.000000000    0.3400    1.008
H    0.812100   0.468900  -0.381600   0.0000   0.0000   0.000000    0.000000000    0.3400    1.008
H   -0.812100   0.468900  -0.381600   0.0000   0.0000   0.000000    0.000000000    0.3400    1.008
5
HNO3   2
N    0.000000   0.155100   0.000000   0.0000   0.0000   664.3122    648986.3436    0.9832   14.007
O   -0.267400  -1.225300   0.000000   0.0000   0.0000   459.7010    251577.4343   -0.5738   15.999
O    1.174400   0.450400   0.000000   0.0000   0.0000   459.7010    251577.4343   -0.4867   15.999
O   -0.984400   0.839600   0.000000   0.0000   0.0000   459.7010    251577.4343   -0.4288   15.999
H   0.619200  -1.603700   0.000000   0.0000   0.0000   0.000000    0.000000000    0.5062    1.008
5
NH4+    2.0
N   0.000000  -0.000000   0.999988   0.0000   0.0000   801.3257    944298.3796   -0.4000   14.007
H  -0.000000  -0.000000  -0.026772   0.0000   0.0000   0.000000    0.000000       0.3500    1.008
H   0.968038  -0.000000   1.342241   0.0000   0.0000   0.000000    0.000000       0.3500    1.008
H  -0.484019  -0.838346   1.342241   0.0000   0.0000   0.000000    0.000000       0.3500    1.008
H  -0.484019   0.838346   1.342241   0.0000   0.0000   0.000000    0.000000       0.3500    1.008
4
NO3-   2.0
N   0.000000   0.000000   0.959250   0.0000   0.0000   664.3122    648986.3436    0.7940   14.007
O   1.075762   0.000000   1.580341   0.0000   0.0000   459.7010    251577.4343   -0.5980   15.999
O  -1.075762   0.000000   1.580341   0.0000   0.0000   459.7010    251577.4343   -0.5980   15.999
O   0.000000   0.000000  -0.282933   0.0000   0.0000   459.7010    251577.4343   -0.5980   15.999
```

Table S2: Interaction Energies of Neutral Nanoparticles. Interaction energies calculated using the indicated models for the geometries and energies of $(NH_4NO_3)_n$ nanoparticles following TransRot simulated annealing. The n=1 calculations assume that the interacting molecules are HNO_3 and NH_3; all other calculations assume that the interactions are between NH_4^+ and NO_3^- molecules. All values are given in kcal/mol.

**Geometry --->**	ωB97X-D3/def2-SVPD	ωB97X-D3/6-31G(D)	OPLS-AA
**Energy --->**	ωB97X-D3/def2-TZVPD	ωB97X-D3/6-311+G(2DF,2P)[6-311G(d)]	"
**n**			
1	-14.3	-15.6	-14.2
2	-280.2	-286.5	-274.0
3	-434.9	-445.9	-431.0
4	-600.4	-616.5	-609.5
5	-748.4	-768.4	-763.7
6	-916.9	-943.3	-937.9
7	-1077.7	-1109.2	-1107.0
8	-1229.4	-1265.1	-1266.6
9	-1392.7	-1434.6	-1437.3
10	-1549.8	-1596.7	-1608.3
11		-1761.8	-1778.1
12		-1927.8	-1948.0
13		-2095.0	-2115.7
14		-2258.0	-2284.7
15		-2423.9	-2453.4
16		-2607.0	-2633.6
17			-2792.9
18			-2967.1
19			-3138.8
20			-3307.5
21			-3484.5
22			-3648.9
23			-3824.3
24			-3985.7
25			-4159.3
26			-4323.4
27			-4505.0
28			-4677.4
29			-4842.8
30			-5012.1
31			-5186.6
32			-5357.9
33			-5523.0
34			-5697.5
35			-5866.7
36			-6037.8
37			-6213.2
38			-6378.3
39			-6555.3
40			-6724.8

Table S3: Interaction Energies of Cation and Anion Clusters. Energies are given in kcal/mol.

p	$[(NH_4NO_3)_pNH_4]^+$	$[(NH_4NO_3)_pNO_3]^-$
1	-164.8	-163.9
2	-329.5	-322.2
3	-486.9	-481.3
4	-645.8	-637.7
5	-808.8	-794.6
6	-964.2	-959.0
7	-1131.4	-1119.5
8	-1289.7	-1280.8
9	---	-1440.9

Table S4. Energies of Cation and Anion Clusters and Component Molecules. Electronic energies calculated using ORCA 5 and the ωB97X-D3/def2-TZVPD//ωB97X-D3/def2-SVPD model chemistry following TransRot simulated annealing. All energies are given in hartree.

NH_3	HNO_3	NH_4^+	NO_3^-
-56.5670690	-280.9360534	-56.9078449	-280.4083933

p	$[(NH_4NO_3)_pNH_4]^+$	$[(NH_4NO_3)_pNH_3NH_4]^+$	$[(NH_4NO_3)_pNH_4HNO_3]^+$	$[(NH_4NO_3)_pNO_3]^-$
0	---	-113.5187604	-337.8703674	---
1	-394.4866600	-451.0816131	-675.4388114	-617.9858919
2	-732.0653385	-788.6585115	-1013.0202672	-955.5542652
3	-1069.6325518	-1126.2232201	-1350.5915281	-1293.1240415
4	-1407.2019600	-1463.7934376	-1688.1560644	-1630.6896575
5	-1744.7779895	-1801.3673339	-2025.7333683	-1968.2558601
6	-2082.3418037	-2138.9324387	-2363.3066991	-2305.8341207
7	-2419.9245746	-2476.5195446	-2700.8852749	-2643.4061666
8	-2757.4929512	---	---	-2980.9794453
9	---	---	---	-3318.5506810

p	$[(NH_4NO_3)_pNH_3NO_3]^-$	$[(NH_4NO_3)_pHNO_3NO_3]^-$	$[(NH_4NO_3)_p(HNO_3)_2NO_3]^-$	$[(NH_4NO_3)_p(HNO_3)_3NO_3]^-$
0	-336.9892829	-561.3919200	-842.3566851	-1123.3212107
1	-674.5674567	-898.9558041	-1179.9247403	-1460.8832952
2	-1012.1351634	-1236.5206041	-1517.4840357	-1798.4538584
3	-1349.7087447	-1574.0933266	-1855.0627111	-2136.0224819
4	-1687.2781085	-1911.6607794	-2192.6221544	-2473.5777262
5	-2024.8455732	-2249.2256073	-2530.1855709	-2811.1580426
6	-2362.4161327	-2586.7970241	-2867.7631308	-3148.7156418
7	-2699.9906657	-2924.3692708	-3205.3280690	---
8	-3037.5647816	-3261.9389168	---	---
9	---	---	---	---

Table S5. Energies of Neutral Nanoparticles and Component Molecules. Entries labeled “def2” were calculated using Q-Chem 6.1 via the ωB97X-D3/def2-TZVPD//ωB97X-D3/def2-SVPD model chemistry, following TransRot simulated annealing. The entries labeled “Pople” instead used the ωB97X-D3/6-311+G(2DF,2P)[6-311G(d)] //ωB97X-D3/6-31G(d) model chemistry. All energies are given in hartree.

	**NH_3**	**HNO_3**
def2	-56.5669393	-280.9358594
Pople	-56.5626555	-280.9105361

	**NH_4^+**	**NO_3^-**
def2	-56.9077021	-280.4082612
Pople	-56.9049384	-280.3798249

**p**	**$(NH_4NO_3)_n$** def2	**$(NH_4NO_3)_n$** Pople
1	-337.5255554	-337.4980662
2	-675.0783764	-675.0260212
3	-1012.6410091	-1012.5649232
4	-1350.2206253	-1350.1214677
5	-1687.7723924	-1687.6483095
6	-2025.3569386	-2025.2117896
7	-2362.9291430	-2362.7610040
8	-2700.4868982	-2700.2941031
9	-3038.0629987	-3037.8490750
10	-3375.6293888	-3375.3921141
11		-3712.9399414
12		-4050.4893616
13		-4388.0405270
14		-4725.5850252
15		-5063.1342038
16		-5400.7106854
