# Supplementary material for: Decomposition and Growth Pathways for Ammonium Nitrate Clusters and Nanoparticles
Source: J Phys Chem A. 2024 Oct 14;128(42):9184–94. doi: 10.1021/acs.jpca.4c04630 (PMC11514028; doi:10.1021/acs.jpca.4c04630)
Supplement: Supplementary file 2 — jp4c04630_si_002.zip [file jp4c04630_si_002.zip › SI_ammoniumnitrate particle structures_PDF_XYZ/HassanAmatTopper_SuppMats_S09.pdf]

Supporting Information for:

Decomposition and Growth Pathways for Ammonium Nitrate Clusters and Nanoparticles

Ubaidullah S. Hassan, Miguel A. Amat, and Robert Q. Topper*

Author Affiliations:

Ubaidullah S. Hassan, Department of Chemistry, The Cooper Union for the Advancement of Science and Art, New York NY 10003, United States.

Miguel A. Amat, Department of Chemistry, The Cooper Union for the Advancement of Science and Art, New York NY 10003, United States.

Robert Q. Topper, Department of Chemistry, The Cooper Union for the Advancement of Science and Art, New York NY 10003, United States. Email: topper@cooper.edu; Phone: 212-353-4370.

Table S9: Cartesian Coordinates of p=(0-8) $[(NH_4NO_3)_p(HNO_3)(NO_3)]^-$: ωB97X-D3/def2-SVPD

p=0 [(NH4NO3)p (HNO3)(NO3)]- ωB97X-D3/def2-SVPD

N	0.70819726263365	1.26090662744877	-1.84007050244191
O	0.20620353861387	1.17267813646978	-0.66052911415843
O	1.70601994561934	0.60232508257358	-2.10882265767121
O	0.15709581412931	2.00069118064465	-2.64239196048427
N	2.28529282861727	0.10606471443099	1.56707870462848
O	1.26610878877123	-0.35933932669247	0.89408360968025
O	2.65409789848466	1.24378771260131	1.35554154818668
O	2.78993472845898	-0.64975473521181	2.37358574016922
H	0.88252319467169	0.38510960773520	0.14887363209119

p=1 [(NH4NO3)p (HNO3)(NO3)]- ωB97X-D3/def2-SVPD

N	2.73980032589097	1.37077218953845	3.01033816081283
O	1.92980149498699	0.85019668166972	3.78699429326084
O	2.55321577113259	1.18709895447247	1.75876617870285
O	3.68034779061248	2.04141725310967	3.39123720185491
N	-3.30753107551406	0.11270405866122	1.84764483086515
O	-2.48614388722633	-0.49925056220817	1.13683921631215
O	-2.89657704630134	1.07186296703644	2.56528569661746
O	-4.48956301676951	-0.19443253020160	1.86395307695237
N	1.97695431516022	3.74701196151544	-0.02140629643794
O	3.08639618498609	3.09295635069009	0.26020796561767
O	0.93187483933761	3.36062026335315	0.47342519753890
O	2.08932928552175	4.68967170158172	-0.75810777522419
H	2.88106821542554	2.30352237788605	0.92998575292413
N	-0.29308245417336	1.04401539229997	2.00246592414253
H	0.12051687511531	1.81673770942738	1.47197944643917
H	-1.28697438855739	1.22663050674388	2.33119379901745
H	0.37085884952447	0.80979692498648	2.76043744523883
H	-0.41789907915203	0.23824979943758	1.38639588536484

p=2 [(NH4NO3)p (HNO3)(NO3)]- ωB97X-D3/def2-SVPD

N	-0.82791214480991	-1.14456261998168	-0.42352463075044
O	-1.06293659616446	0.04901886555967	-0.68007573097833
O	-0.15643603110388	-1.82995487175496	-1.19904189623522
O	-1.27744227541119	-1.65092195564521	0.62405094801289
N	4.14921308310676	0.06524444727491	-1.33774972824294
O	3.74465008089171	0.10451596578247	-2.52853465598985
O	5.32371088506837	-0.12485054113000	-1.09038765316463
O	3.31238274611072	0.21944436403837	-0.41522448108311
N	0.44928793817099	3.54588470774400	-3.14300855591015
O	-0.05822075645615	2.39943246542083	-3.25160711062863
O	0.26319579528609	4.39071199387949	-3.99753589056094
O	1.14377866552409	3.79900660757865	-2.12863382529356
N	-1.38484210113544	0.97284103123895	2.64273516909957
O	-2.24022212087692	0.02607295540270	2.28575017596126
O	-0.26981073602159	0.93993501149128	2.16392904106130
O	-1.79847634960981	1.78565870813063	3.42478057584541
H	-1.79644197303763	-0.58305247232904	1.58427113003579
N	1.10332281299119	1.82171386285178	-0.27463499492174
H	1.10280084446003	2.60463799715868	-0.98461185857428
H	1.96971276967881	1.24240476348976	-0.37540968475901
H	0.27746063601007	1.21781693222786	-0.42145319622434
H	1.04787236533013	2.17836218172636	0.67729542699242
N	1.11201812701393	-0.04143763750543	-3.00907993198774
H	2.14012853950339	0.01005293894732	-2.74790825707151
H	1.03756053307510	-0.42387157905107	-3.94808040485520
H	0.69342491762061	0.92013414497087	-3.01933691862005
H	0.59842734478497	-0.66433326751721	-2.35449206115695

p=3 [(NH4NO3)p (HNO3)(NO3)]- ωB97X-D3/def2-SVPD

N	1.04177547249646	5.46828796769386	0.60289628891474
O	0.88909506072405	4.25440415875194	0.37254149764343
O	0.18583610945926	6.10435938133054	1.21368780844634
O	2.08215712807168	6.04492745312622	0.21679888404370
N	-2.08734425923446	0.91100003982448	-0.83163886237376
O	-3.01207111011353	0.09601537983268	-0.73098571148534
O	-2.19234611709392	2.03280916029753	-0.29800181946498
O	-1.06319063770489	0.62735201221936	-1.47334107907087
N	-4.43491395749484	2.81611577865497	2.77425712703883
O	-4.82373436571695	3.47335974147525	1.76735615507255
O	-3.47841480140349	3.26834657767690	3.42897929316066
O	-4.98333902080531	1.76424173749844	3.07162581745577
N	-3.39305368305257	4.29455918760840	-1.85910755334846
O	-3.86430313821485	3.35812860993586	-2.52471400701645
O	-4.03061015548233	4.75253560890946	-0.89980662855677
O	-2.27475747565136	4.76535030925257	-2.13933845852828
N	3.55924105849052	4.25074981151831	-2.14511662419651
O	3.87881756769298	4.71723039051315	-0.95141505832233
O	2.43314377772716	4.46241320582135	-2.55404360648043
O	4.42248925343333	3.65383855543273	-2.72911483340008
H	3.05746170141468	5.19489576180763	-0.53426219574017
N	-2.14525488048691	4.42036770910088	1.22334966317962
H	-2.60534071512511	4.19927275144507	2.12984861269104
H	-1.81186950611142	3.53671584060236	0.82350355971565
H	-1.34451541765839	5.06650074211965	1.31580715419110
H	-2.85675250965778	4.78274544768537	0.56579070309823
N	-0.08002757306719	3.15297950283362	-2.03475920182213
H	-0.34634737376419	2.16104613939583	-1.84016806976163
H	0.33082436518076	3.57082474656644	-1.18272630034471
H	-0.94333659042943	3.69603836003013	-2.25771553062644
H	0.60895043699705	3.23110286255975	-2.78007335429164
N	-5.11281124123323	1.93308174312992	-0.40836227852030
H	-5.06021655431456	2.52497548012198	0.46793444950938
H	-6.05112603051585	1.56190145245660	-0.51889436664300
H	-4.41928005731294	1.16035531828821	-0.35176591722481
H	-4.84484376004233	2.51602407448227	-1.22642955694190

p=4 [(NH4NO3)p (HNO3)(NO3)]- ωB97X-D3/def2-SVPD

N	4.97404876934408	3.29729161086312	4.92224935742450
O	5.17500194177967	4.37445314815248	4.31745711249358
O	4.50448905485882	2.32186113064026	4.26961954164650
O	5.21778902247740	3.18246858711012	6.10589214685260
N	6.34327504714923	3.39520808677437	-0.71658367293308
O	6.47822688201635	3.36268825137946	-1.94446191989284
O	6.99512268612654	2.63291724431582	0.01680646544744
O	5.52228701366125	4.18973057467636	-0.21434018471329
N	1.84772538540059	6.47750818404569	0.08433626868049
O	2.34274363421295	5.47247270557599	-0.47168331929826
O	0.77512346638372	6.94241938977275	-0.33779164188767
O	2.41265042586817	6.98906103356569	1.05343824196722
N	1.42581346244682	3.92693486144647	-4.97656080777039
O	0.56959665801942	3.63057478748378	-5.83313363359196
O	1.25212968534279	4.92206659938893	-4.24724300150385
O	2.43994441197500	3.23912718809072	-4.86200623250195
N	1.90352617412071	2.53834671907509	0.11447263937563
O	3.07286617992669	2.16461647211138	-0.17306169262697
O	1.64019392812270	2.87739127038059	1.26683793428391
O	1.05560711333913	2.55721433020316	-0.78179474566380
N	-2.30159070241370	5.02688229999821	-4.93692860892576
O	-1.51389727032945	5.06855901868133	-5.99812181813884
O	-1.95341432275487	4.32979019089919	-4.00187008584006

```
 O   -3.30262164499952      5.68546241877656     -4.99394073875382
 H   -0.67438769897516      4.49517463953248     -5.82058034187409
 N   -0.15581962117854      4.83877813280343     -1.82949075842306
 H   -1.14838739955877      4.78583124577219     -2.05339467502569
 H   0.36758962401737      4.84796283901400     -2.72258779446762
 H   0.15224409660732      4.01167471740723     -1.28902756166821
 H   0.09706683457353      5.70125979486685     -1.29034076099765
 N   3.80782163407903      4.70180776353292      1.96011918001305
 H   4.49206822720951      4.50589345081247      1.21228999979384
 H   3.03132058581333      4.02983079111602      1.86824122350078
 H   3.42225795376291      5.64605484492672      1.78209135794482
 H   4.27385959975394      4.60958156121857      2.89335118134868
 N   5.04350437409734      1.70984825823328      1.73965551618216
 H   4.86775270037384      2.07745181209296      2.72031206238375
 H   4.22297560461172      1.87999477477005      1.11819156635289
 H   5.19780972217557      0.70665212807762      1.80203278637427
 H   5.87627084106560      2.14363080880491      1.28348542957787
 N   3.69594664642573      3.84892910633639     -2.39589202612019
 H   4.72991427273351      3.86594230517615     -2.43787855749004
 H   3.43392106484643      3.08212704095088     -1.75315087966564
 H   3.26520987630534      3.70224455189558     -3.32787775103283
 H   3.35204605918561      4.71203132925128     -1.95929680083633
```

```
p=5 [(NH4NO3)p (HNO3)(NO3)]-  ωB97X-D3/def2-SVPD
 N   -5.08668444520748      0.67316576074766     -7.08285227632663
 O   -5.80876916991694      0.34958407036346     -6.12841795776928
 O   -4.57196737743660     -0.18966708840789     -7.80629260549985
 O   -4.83750205445293      1.88087565451096     -7.29493189127984
 N   -1.09927376187897     -1.78724913589471     -6.06490941164343
 O   -1.99410645249071     -1.79887510734035     -6.92484002513084
 O   -0.17608387470036     -0.95219445584601     -6.15697100364242
 O   -1.13666272861358     -2.58775240454002     -5.12496215790396
 N   2.79680393426441     -0.31338502671389     -1.76402062660970
 O   2.11993586054893     -1.33841606922667     -2.03076811882930
 O   2.42620259578396      0.79290538641398     -2.22908389162023
 O   3.79485423344625     -0.39283842840305     -1.07483684980008
 N   -2.93395438116773      0.59128875750601     -3.34180891324759
 O   -2.67676287029735      0.33729194776261     -4.53882688744523
 O   -2.03484753855090      0.49913261537552     -2.49175885402894
 O   -4.06914691978039      0.93778672084934     -3.01196895736461
 N   -3.22788532399748     -3.55842492631919     -2.80323440982756
 O   -2.31070988124770     -4.15047495930331     -2.24117129161534
 O   -3.31221822097165     -2.32116752334987     -2.77116721191394
 O   -4.11001631666946     -4.19634197434554     -3.43240936041722
 N   -1.40399895045387      3.38464613285847     -5.60180928621905
 O   -0.25762700496452      2.95618383328454     -5.79972874277357
 O   -2.14054044325827      3.65582642857824     -6.56676455264472
 O   -1.84096367266575      3.50404359370428     -4.44179269702917
 N   -4.21167769790183     -5.97593185363699     -6.08885067313995
 O   -3.56636046887160     -6.17712859935912     -4.94459051343519
 O   -4.83930837756820     -4.94344473295374     -6.21879021049337
 O   -4.11731703975848     -6.85213843853674     -6.90075419655502
 H   -3.74524806984794     -5.38780596944909     -4.32225794880582
 N   -0.49045052695675     -1.93700951855527     -2.51077025593448
 H   0.48616698373833     -1.66268206758095     -2.26092011473934
 H   -0.83352661525631     -2.77207298864559     -2.02273333622526
 H   -0.56109524354844     -2.15125847924349     -3.52405970801366
 H   -1.15535816792743     -1.17364239136585     -2.32671020977540
 N   -4.56688684485605      2.95292179085551     -4.80340466137318
 H   -3.60747727207002      3.35262261224862     -4.71276022755116
 H   -4.73875999988860      2.65473498187330     -5.79392822092621
 H   -5.26593719062068      3.62914677960623     -4.51156215568202
 H   -4.60575628352336      2.11176758074005     -4.18999420152159
 N   0.38243386350980      0.88310288647598     -3.99028179300267
 H   1.21224857053707      0.80721175996857     -3.34002794830062
 H   0.35017193353698      1.77942373162580     -4.50237721233519
 H   -0.48774732851215      0.81532211414316     -3.43841258472322
 H   0.36915858941971      0.12349225428102     -4.68627320967631
 N   -2.04434899362750      0.94960606573647     -7.27016529871066
 H   -2.79358744566817      0.50918513688878     -7.82785259042648
 H   -2.32037186007277      0.84102150500412     -6.28303923462427
 H   -1.96053477509293      1.95921308329950     -7.45707295047985
 H   -1.16302297931969      0.43331564443888     -7.36541268574520
 N   -4.38997135004050     -2.05798374871043     -5.40440514613826
 H   -4.81673723340650     -2.98047989965122     -5.52115751147410
 H   -3.56287855521481     -2.00902439420431     -6.02695352404852
 H   -4.07853544730897     -1.98090073656037     -4.42001190959410
 H   -5.03983640920263     -1.29108691099724     -5.64398878596538
```

```
p=6 [(NH4NO3)p (HNO3)(NO3)]-  ωB97X-D3/def2-SVPD
 N   -7.27234238765882      5.59933700489889      0.76954691684839
 O   -7.72815152232451      6.17721603018627      1.76760007132189
 O   -6.44770715467976      6.20526725157308      0.03100372913548
 O   -7.60485243061336      4.45399002481260      0.49396847540241
 N   -0.92924403910298      3.89371676600972      0.44233595023676
 O   -0.23306974602171      3.15503607856191      1.14948669490063
 O   -0.65657751234709      5.10594472721055      0.34745376508605
 O   -1.92620340780090      3.44121541122270     -0.15304875379147
 N   -2.98804028027183      8.24177935566514      2.98371659874627
 O   -3.43677434646644      7.41817527954736      2.15192619568213
 O   -1.76674431596544      8.32731994098279      3.17429107866339
 O   -3.77349738845326      8.95362716092992      3.61267388296685
 N   -4.01767901565807      4.80578039974704      2.94868345320473
 O   -5.05034315379239      4.78450350778302      2.27986687655108
 O   -2.96509450407070      4.35444720832409      2.47105366355146
 O   -4.00884045189123      5.30160937593256      4.08615833799733
 N   -5.23470361987189      1.00793772644449      3.35676151226438
 O   -4.45977054406196      0.15509165178862      2.91470690640201
 O   -6.46314954409546      0.83346706737814      3.30028432212687
 O   -4.78708010557810      2.06394137259378      3.85939943119744
 N   -1.16473411852232      3.30999596454272      5.57911087108045
 O   -0.24735893122081      3.61809659873239      4.80912469916901
 O   -1.52592659665864      4.07351189486683      6.48547004684787
 O   -1.75473047754056      2.21530798022942      5.43167302311369
 N   -6.76366224641023      5.57151695700736      6.47344553798559
 O   -6.80968061506051      4.86577469809463      5.43901317103360
 O   -6.30465572133018      5.08357926162283      7.51628282633425
 O   -7.14524631674794      6.74540518300926      6.43200406443185
 N   -4.96731964263243      9.11793353048212      0.22191795275453
 O   -6.03251338591544      8.61753801369534      0.84980521440355
 O   -4.46439803565560      8.43590219884077     -0.64793473543698
 O   -4.61966888414611     10.20730345474164      0.56922899295549
 H   -6.18593301069410      7.67542136878566      0.49534697597949
 N   -6.05278821772887      7.30430640521535      3.82580839262147
 H   -5.42500099176510      6.49521838699669      3.95234466785755
 H   -6.65338968512107      7.10310150023492      3.01386860326448
 H   -5.43903790051494      8.12176852770554      3.65556557994563
 H   -6.61408974789219      7.37735272934872      4.68886589128504
 N   -7.35483776973683      3.45930155563066      3.17353658609058
 H   -7.96209302383368      3.67468861146814      2.37596304624568
 H   -6.44016285482174      3.88654498337836      2.95517468204119
 H   -7.19267810383798      2.43699718610937      3.29786861055440
 H   -7.66582964596157      3.90796624595180      4.04084483537591
 N   -0.67026910985365      5.77711359899102      3.07095064444737
 H   -0.91775764900980      6.78497229375656      3.14469487157143
 H   -0.16893660829637      5.39287967358398      3.87882768878876
 H   -0.18786678395387      5.58841633676147      2.18019253882870
 H   -1.55915842307650      5.24953669284560      3.00790736839839
 N   -4.28705668436417      3.55048877835751      6.25910763267421
 H   -5.03309092494905      3.87263798149733      6.90201362269036
 H   -4.27013854208100      4.21350026818215      5.46895606382890
 H   -4.49027842025611      2.63872193485010      5.84136236953800
 H   -3.33528924221844      3.56257631680071      6.66576278319024
 N   -2.09459812080571      1.71212411020463      2.73482674640346
 H   -1.32032113256009      1.70291365847134      2.06235642752899
 H   -1.75893237989192      1.79444563403474      3.71780585972009
 H   -2.76559951343163      0.93003721433469      2.67088269754700
 H   -2.62314840866224      2.57387803189979      2.54455116906533
 N   -3.54997236282633      5.74976918112471     -0.10400229869875
 H   -4.56762840509882      5.61132576206079     -0.02567577506356
 H   -3.35896774167611      6.30826380496060     -0.93261901962612
 H   -3.02392730843688      4.84936973852605     -0.14243255750667
 H   -3.24317525017457      6.30328240417058      0.71309084074397
```

```
p=7 [(NH4NO3)p (HNO3)(NO3)]-  ωB97X-D3/def2-SVPD
 N   1.23035803396013     -6.50712084307728     -2.41948910340305
 O   2.47485122799088     -6.47493115794404     -2.20868853205362
 O   0.75617939983667     -5.83392891540432     -3.33688051840508
 O   0.51008201129759     -7.18608864475088     -1.67950121025641
 N   4.43825902016255     -3.49795446663790      0.97604341998502
 O   4.66419020481256     -2.34944346559653      1.39502362844733
 O   4.39723737727720     -4.45203894307650      1.76276121386204
 O   4.24655558941469     -3.68664989077390     -0.23998653813305
 N   4.43248180817011     -1.29793186197881     -4.40813995887780
 O   4.02785582192726     -0.95861964725220     -3.27212162292822
 O   4.33329879150372     -0.48765652671115     -5.34264902054883
 O   4.89004658246699     -2.42883567576528     -4.58678791881848
 N   -0.50293567310169     -6.27825277424386      2.09477953457069
 O   -0.33149135787495     -5.42175790142180      1.19042420539826
 O   0.34318670391436     -7.14902522374927      2.28183550498882
 O   -1.52470985368567     -6.20995321793041      2.79682109737238
 N   -0.13831848183468     -1.95048671025880     -3.67926412107016
 O   -0.73303679834982     -2.83540688806791     -3.06919427096957
 O   1.05948293258515     -2.09345767122672     -4.00788892176435
 O   -0.71527112737588     -0.88722747306582     -3.96481567253430
 N   -4.30340343799535     -2.61358267821335     -0.37821127747927
 O   -3.44119661454941     -3.20950076143471     -1.05283058870466
 O   -4.67552377063258     -1.48568473223220     -0.72534876278832
 O   -4.75961300619337     -3.14174498133975      0.65024427966606
 N   1.17660258733092      1.47133243969169     -1.07262960088313
 O   0.07775895864497      1.51850138306302     -1.61526567801121
 O   2.22675180731942      1.55779685458177     -1.73457805336082
 O   1.27747614349333      1.32348824792191      0.16896522511320
 N   0.46761669307041     -2.50182139047809     -0.49013702837980
 O   -0.69147742646457     -2.58157894288985     -0.03152967273767
 O   1.18609773463670     -3.49742970404321     -0.55912617581158
 O   0.86624210415956     -1.40273457390889     -0.90262869263549
 N   -0.90750579237787     -0.41291779126605      1.93976779419946
 O   -0.97367974090668      0.59333330385611      1.07642815441528
 O   0.17701576785615     -0.80769532173744      2.27781928911025
 O   -1.97868312838037     -0.83766497634033      2.30891387073041
 H   -0.02050490012333      0.84632457934296      0.76881326726126
 N   2.37145290169965     -5.97934919943776      0.56085223696329
 H   3.23070436722423     -5.60871624763907      1.00956525009747
 H   1.72060754606412     -5.19575349906902      0.42552202692380
 H   1.86170516455238     -6.64995359038351      1.15367854850006
 H   2.56960787316631     -6.37306581364914     -0.37621151461831
 N   -2.39629910242924     -3.66555097416224      2.00954584543785
 H   -2.20605025262146     -4.60772915331459      2.41167536922565
 H   -3.33261963032284     -3.61525876126199      1.55455071695713
 H   -1.66919697274847     -3.49196029585307      1.29935602761582
 H   -2.32021252383427     -2.91613675135108      2.69844646618195
 N   -1.33822519047910     -5.05195593842018     -1.43715506109295
 H   -2.26803987413292     -4.62147002702035     -1.33051922825056
 H   -0.76968159084424     -4.44032472128936     -2.03944031160665
 H   -1.33844917428708     -5.98348532785846     -1.85799256005416
 H   -0.89952619812839     -5.13588655332432     -0.50529798162322
 N   1.92822117820248      0.53787522741742     -4.31779960228423
 H   1.57506723310991     -0.43658033196503     -4.20002217170398
 H   1.25514912820591      1.05783377816258     -4.87334911478177
 H   2.85605176911086      0.45816750910037     -4.77672150608703
 H   2.02941665510224      0.97455306410675     -3.37878715419867
 N   -2.10348757132160     -0.58530370444555     -1.51385892936676
 H   -3.12187717722369     -0.73187786288974     -1.39329220529125
 H   -1.81269899829286     -0.67654737185649     -2.50256641335025
 H   -1.62238250775174     -1.32948528631288     -0.97700331429802
 H   -1.78224503357003      0.31567045037016     -1.15439531441805
 N   3.05254712018452     -3.78917457316313     -2.83380787353864
 H   2.19510524508971     -3.32675128698653     -3.17539873632209
 H   3.84673491496247     -3.48039953623881     -3.41813447106323
 H   2.92205185532622     -4.81689063395025     -2.84533040423562
 H   3.22695278214677     -3.51822992569384     -1.85937598182441
 N   3.56763735412189     -0.75450269892551     -0.55713437468032
 H   2.59615201957683     -1.10543964700785     -0.65349242678858
 H   4.03204746000428     -0.91423896268588     -1.46134307037839
 H   4.05627145612659     -1.27294665606682      0.20919068027129
 H   3.49629758202530      0.25026424742550     -0.38910299088257
```

```
p=8 [(NH4NO3)p (HNO3)(NO3)]-  ωB97X-D3/def2-SVPD
 N   4.89572556094170      5.73753520859142      6.86295955757785
 O   4.41691562318421      5.72287686807426      7.99910736694964
 O   5.97891033573721      6.28909596932182      6.62844939570727
 O   4.27511047711593      5.19762924081259      5.91498206672865
 N   -0.77082201789503      3.66937726560510      5.81326768339907
```

O	-0.67197237889065	2.49676614043125	6.16710155383741
O	0.23919385711695	4.33589222097907	5.51355933190660
O	-1.88948738896550	4.21051594990073	5.74876314647097
N	-1.06136987855923	7.52105967659771	7.86909065823267
O	-1.30305670220439	6.31470047323567	7.61433053316446
O	-0.21629182422521	7.80443176455044	8.72645083254776
O	-1.64142991825565	8.39894754424309	7.22220560494084
N	-3.36489251470082	1.23354518218240	7.12736398430924
O	-3.50397663818900	0.43739406012606	6.19420224647968
O	-2.64834202119709	0.93641992529652	8.10595271421742
O	-3.91480208494649	2.34947343467908	7.09466238008283
N	0.01252793098263	1.09069002772070	3.69661778375497
O	-0.78396673178671	1.66525358579960	2.93430173474293
O	-0.33457370513369	0.06302022437911	4.29572684845710
O	1.14953896304674	1.56225411861098	3.84786727931014
N	3.99443704739636	4.08084634969467	2.08373398673104
O	5.22161587897742	4.38900930436244	1.97504938761096
O	3.27190923764203	4.14438916622597	1.09096858317774
O	3.57299231668695	3.73705819324282	3.18217461740696
N	2.39603155617908	7.09480456244724	4.11158393811387
O	3.15193100033352	6.94775861524582	3.14577917924436
O	1.25874882022552	6.57180881478065	4.08562835561767
O	2.74838277571925	7.73818686519959	5.10536767759759
N	-3.14759524499481	5.16561169404648	2.60849119435118
O	-4.14809301074274	4.44980463889460	2.46193313163588
O	-2.02572628444858	4.63843950791174	2.75733861874298
O	-3.25512838154021	6.40133415800946	2.63845878108874
N	1.00945753358044	2.42687147254701	8.52515431725365
O	1.74744356046252	1.53032302146490	8.04116408451259
O	0.01030768767511	2.08585913638421	9.17608494565845
O	1.26839790850223	3.61073585802634	8.33010105826529
N	6.89299313484166	6.37882548999716	0.06335935818509
O	6.00631359797557	5.43581971066348	-0.21812113651976
O	7.11780870778569	6.61640153772948	1.23563016447061
O	7.40525427769628	6.91845345453619	-0.87611282662883
H	5.65398451594799	5.03660380723409	0.66088454340919
N	0.79291460887690	4.20565991717127	2.56185929176452
H	1.03429659792370	5.01163162946155	3.16524210764612
H	1.44839971491671	4.19973384942245	1.76844178915620
H	-0.19363468510720	4.28415208275002	2.29104990111162
H	0.92737570048798	3.31579055496241	3.06856044640737
N	-1.49732009136467	6.86405578204540	4.77698481320555
H	-1.76255987671689	7.55722085489318	5.49346212929722
H	-0.49537727169553	6.95142023412242	4.55759187179124
H	-2.06977109157173	6.91972134507972	3.91334793074534
H	-1.63742626532268	5.92223559116444	5.18610674098160
N	-3.22427750193856	2.33259889016965	4.09474375000747
H	-2.37906590560440	1.95246518826416	3.62672542831162
H	-3.77033333285993	2.87583219861439	3.40386211260774
H	-2.90775196583897	2.99988353724739	4.81658162477319
H	-3.73041195722417	1.58304023878378	4.57992816817084
N	-2.11256004264558	3.84877153708646	8.68933152223273
H	-1.29401584801041	3.22049573231652	8.84561617699751
H	-1.80774868224857	4.77375260247037	8.33397334725531
H	-2.58368327007408	3.97048069882616	9.58183575497445
H	-2.77518784303917	3.38346076295640	8.03106537207612
N	5.77911955838289	6.41602791547231	3.83211193623814
H	5.73249776429753	5.47290350773603	3.41301186227725
H	5.97665945596963	6.36681922782631	4.85300967712424
H	4.83250824944313	6.82054132547011	3.68231409242099
H	6.47006005931006	6.95134289285969	3.31061187930947
N	-0.32053819787698	-0.12969482839227	7.05544281512863
H	-0.26926352874936	-1.12269112299076	7.26110757764068
H	-0.29891912151101	0.00697273689588	6.02213240095783
H	0.48688045277818	0.36586456493777	7.48066473487150
H	-1.20687272346812	0.25766125711925	7.45604243061787
N	2.68661801111588	2.95561443759217	5.77443236038173
H	2.94830140055444	2.42743342949582	4.94334263706472
H	3.35825876340984	3.72965610147822	5.93938094824253
H	2.58522063384638	2.35048838774832	6.60460428053853
H	1.75336412712627	3.37095536510780	5.60370017708160
N	1.71884081782849	6.24536585482919	7.32186314544760
H	2.59127098899048	5.85123110664016	7.71703281855256
H	1.98389352421899	6.89720510828174	6.56088408376355
H	1.17256872599463	6.74093089345497	8.04540865075344
H	1.13402746831842	5.49893837084588	6.93025054932889
