# Supplementary material for: Decomposition and Growth Pathways for Ammonium Nitrate Clusters and Nanoparticles
Source: J Phys Chem A. 2024 Oct 14;128(42):9184–94. doi: 10.1021/acs.jpca.4c04630 (PMC11514028; doi:10.1021/acs.jpca.4c04630)
Supplement: Supplementary file 2 — jp4c04630_si_002.zip [file jp4c04630_si_002.zip › SI_ammoniumnitrate particle structures_PDF_XYZ/HassanAmatTopper_SuppMats_S08.pdf]

Supporting Information for:

Decomposition and Growth Pathways for Ammonium Nitrate Clusters and Nanoparticles

Ubaidullah S. Hassan, Miguel A. Amat, and Robert Q. Topper*

Author Affiliations:

Ubaidullah S. Hassan, Department of Chemistry, The Cooper Union for the Advancement of Science and Art, New York NY 10003, United States.

Miguel A. Amat, Department of Chemistry, The Cooper Union for the Advancement of Science and Art, New York NY 10003, United States.

Robert Q. Topper, Department of Chemistry, The Cooper Union for the Advancement of Science and Art, New York NY 10003, United States. Email: topper@cooper.edu; Phone: 212-353-4370.

Table S8: Cartesian Coordinates of p=(0-6) $[(NH_4NO_3)_p(HNO_3)_3(NO_3)]^-$: ωB97X-D3/def2-SVPD

```
p=0 [(NH4NO3)p (HNO3)3(NO3)]- ωB97X-D3/def2-SVPD
  N   -2.67770349451978      0.26543908901724     -0.03813796660825
  O   -3.31973061074842     -0.49815206736888     -0.77853435039386
  O   -1.49328940881624      0.52115806558566     -0.31190980591302
  O   -3.22803187002408      0.75005827046736      0.95517587648872
  N   -1.26028746576788     -2.53726375900575     -2.44650518958013
  O   -2.07984320626465     -1.53884851492014     -2.77809761562491
  O   -1.15147961400652     -2.81179859874050     -1.27616999737033
  O   -0.70598833367207     -3.07604583718885     -3.36983772923320
  H   -2.47401182181242     -1.14842359894525     -1.92366367875795
  N    0.22014781777400      2.95716270471620      1.26543751462580
  O    0.30157096889207      1.62818236525736      1.18012680046475
  O   -0.74594307994431      3.48999465067208      0.77017546807085
  O    1.13304393636630      3.49556929594814      1.82902278675863
  H   -0.50483040865059      1.30032338126636      0.66836646422223
  N   -2.67828385945648      3.32703846986505      3.19168803465866
  O   -3.46281691537955      3.06274148004823      2.13967978022622
  O   -1.78515376025271      2.55116712800412      3.42956916967196
  O   -2.96008711743340      4.32233387494288      3.80116249054676
  H   -3.13513275628320      2.21885060037866      1.71762794774706
```

```
p=1 [(NH4NO3)p (HNO3)3(NO3)]- ωB97X-D3/def2-SVPD
  N   -1.29926687909026      0.71169358360537      0.95430056947732
  H   -1.65251954242409      1.66978861093433      1.05205777691124
  H   -1.99180498270859      0.09120882649588      0.48518727640065
  H   -1.03216215773912      0.36977818469198      1.88845099775088
  H   -0.46111600814581      0.71793878097398      0.36124007769402
  N   -1.88383977869243     -1.21125972908503     -1.51415905929304
  O   -1.19351510072757     -0.17029200023466     -1.75915017093287
  O   -2.70219407710980     -1.14490195025821     -0.58049643838933
  O   -1.73039950372173     -2.21985688478863     -2.17390888535495
  N   -0.23511951484768      1.62971244940963      3.80333911880926
  O   -1.40652528151034      2.05626526263023      3.66541054365468
  O    0.02724896056247      0.47140928830157      3.49372641132926
  O    0.64437170835199      2.40008323106865      4.22756293692654
  N    1.95365967026577     -0.20425931375924     -1.24428306093588
  O    1.23729506155901     -0.46413055787625     -2.32793698699391
  O    1.36281195588418      0.09778690892921     -0.22297256198275
  O    3.14226656506897     -0.29690892876125     -1.36488598284723
  H    0.22812776136789     -0.38169484746915     -2.10163570204223
  N   -1.94557757666931      4.55570051869542      1.67299704421951
  O   -1.46076937349924      4.50500634945508      2.90770626838277
  O   -2.30193754329559      3.51556210132427      1.15441892163206
  O   -2.00344996181120      5.64619258786241      1.18046860315507
  H   -1.43673700087645      3.53023145125184      3.21242106858802
  N    1.94375030223871      2.77675379138795      1.29004819064048
  O    2.56511500483899      2.55907426174657      2.45494330901581
  O    0.73219673620795      2.71736877318299      1.28712936857929
  O    2.65416371830116      3.01523854549379      0.35997544032518
  H    1.85148950082211      2.42939306739119      3.15317466788010
```

```
p=2 [(NH4NO3)p (HNO3)3(NO3)]- ωB97X-D3/def2-SVPD
  N    1.67829773480405     -0.45967167649849      1.39076254479790
  H    0.90686085128207     -1.10053907612029      1.57021936857894
  H    2.57232764652588     -0.82084006954390      1.77026966252603
  H    1.50192479862918      0.49485877424653      1.77179633393000
  H    1.76817875871845     -0.36821439409010      0.36349499749496
  N    4.57171442195463      1.36438592788339     -1.27529103029679
  H    3.96992949907346      1.76299305828637     -0.53474399776014
  H    5.03614836614372      2.15002514892424     -1.72834893996728
  H    3.98961419636214      0.81230832178288     -1.93613249139380
  H    5.25332362233178      0.75814488003287     -0.79166353158899
  N    2.34798687991694      2.73219576511597      1.49468281344731
  O    1.37081864793155      2.22047081207032      2.05379990606053
  O    3.29588430505810      2.01802993012256      1.12310855356146
  O    2.37742392778841      3.96068335333722      1.31270107547615
  N    4.75983798046324     -0.87656866900848      0.93687139364987
  O    4.35664628515806     -1.02913679906340      2.09598015364892
  O    4.14972118277757     -1.33180307897414     -0.02373273467800
  O    5.81500335798708     -0.20742524478605      0.73077765711687
  N    1.95515859130867     -0.45945674599779     -2.34343540283600
  O    1.66346359828866      0.21667772387939     -1.34283933244319
  O    1.18697772490867     -1.36786884281180     -2.72656691406630
  O    2.98959533799862     -0.23492192631482     -2.97201713473413
  N   -1.25259903014672     -1.89100946279880     -0.50950472292751
  O   -1.13588848270704     -1.50033881524959     -1.76499821316001
  O   -0.23288529745788     -2.15726266650500      0.09895326399896
  O   -2.37032395681921     -1.95427176520834     -0.07530735977727
  H   -0.13263204886140     -1.44309667275223     -2.02505608775331
  N    6.46482035670571      2.78557444759059      1.79416787876660
  O    6.32840381888634      1.64169771735473      2.46314538614890
  O    6.35229774138588      2.74940000497752      0.58624592496775
  O    6.69939556262215      3.75225598724733      2.46113297983564
  H    6.10498452793267      0.90914631097214      1.79562029098531
  N    4.90042082578300      5.04302046291288     -0.67415079101085
  O    4.43202565505696      5.27680009133660      0.54249043919356
  O    4.35251129483448      4.17779095089562     -1.33036013512536
  O    5.82209758673731      5.72293024113676     -1.02397630282176
  H    3.66307873063606      4.63612199561702      0.73354749815497
```

```
p=3 [(NH4NO3)p (HNO3)3(NO3)]- ωB97X-D3/def2-SVPD
  N    4.52167475364750      1.01258726079503     -0.08962029448611
  H    4.80377303818964      0.05983193048567      0.15632981936634
  H    5.28470893022037      1.70306922048172     -0.04217202466097
  H    3.75579267477559      1.30206923563691      0.54342967474372
  H    4.15395028292403      1.04461798812479     -1.06049521323968
  N    2.22848214891402      4.57394456691084     -2.33263254500029
  H    2.69640566162265      4.44774858993023     -1.41905974937506
  H    1.92956154690723      3.63660947595485     -2.66734055148167
  H    2.89966619598196      4.98485157869769     -2.98718404290260
  H    1.36978911008090      5.12781362979429     -2.20590947555901
  N   -0.28007738149645      1.13575624436522     -0.18228233502697
  H    0.39623177398578      0.73777506491203     -0.86255534402280
  H   -0.20776825415796      0.56557034590813      0.67199278539236
  H   -1.21905497400214      1.16485800099309     -0.58824876566515
  H    0.01806075427544      2.10441096934086      0.02693658569662
  N    5.03976282341309      4.07296054856803     -0.85173773567980
  O    6.03142285631866      3.35107378706140     -0.74047940399972
  O    4.01246145246897      3.86057907686901     -0.19832174524933
  O    5.05121759568014      5.04675933698327     -1.64936355899608
  N    2.16586507822837      1.30558539242340     -2.32321022093096
  O    3.33883760142949      1.65474018484021     -2.53565312131354
  O    1.93242148640756      0.27844137166227     -1.66459870743049
  O    1.22488528484065      2.00227949078974     -2.73988706499261
  N    2.03488968491946      0.05439696705401      1.72533126216280
  O    3.10460026243660     -0.56143458379285      1.97084329514797
  O    2.10216936214151      1.25318504304752      1.42981263424363
  O    0.95440565203854     -0.53461605437746      1.77378948955463
  N   -0.62160342238859      4.24118253904922     -0.93782167742199
  O    0.35671434663807      3.92712825432221     -0.24993802713211
  O   -1.71409289513074      3.65320990623155     -0.72663141775084
  O   -0.53799476475429      5.09984997233903     -1.81661734305117
  N    5.34201870373787      5.09224960369400     -4.83218425599371
```

```
 O   6.24278135116923      4.88581632857686     -3.88003535501472
 O   4.18189342726496      5.25252343738958     -4.50232544691836
 O   5.76195140712475      5.10784131617281     -5.95400550996246
 H   5.77763788985636      4.91269330741263     -2.96537568898192
 N   4.57792863615345     -2.97521495145257      0.47834635449979
 O   3.47451647804633     -2.95522582153123      1.21542223310141
 O   5.13564847313909     -1.91870013522899      0.24786158069157
 O   4.93565339706137     -4.05512157094955      0.10322960172211
 H   3.28245064554549     -1.99113347808967      1.50746109988907
 N  -3.44617566588078      1.75170993462915     -2.63001521760452
 O  -3.29993304620668      3.07066424305027     -2.61789008486857
 O  -2.79178172179623      1.09283014262932     -1.84423930845365
 O  -4.22907490584435      1.32071465244435     -3.42798530082356
 H  -2.63007089971135      3.32847497556707     -1.88474874383202
```

p=4 [(NH4NO3)p (HNO3)3(NO3)]- ωB97X-D3/def2-SVPD

```
 N   2.58457317913887      1.83633419387078     -2.37132462738968
 H   2.32130105370869      1.42995245614860     -3.27580821570506
 H   1.75626364648799      1.70073029178354     -1.75871180241287
 H   2.70178130077068      2.84263438704408     -2.48502037925173
 H   3.42505155563269      1.35725588252960     -1.98055115129850
 N   1.19676368091972     -0.86731247229260      1.69626342765912
 H   2.05178787015801     -0.71400686431485      1.12658377022073
 H   0.64483095509343      0.00272702014345      1.63340067753080
 H   1.44302099774510     -1.10575636802972      2.67607696188746
 H   0.68455384665961     -1.63229764541023      1.25077450855632
 N   3.60158373018848     -3.65330213361166      0.92831001550582
 H   2.96221434060249     -4.06015461555614      0.20444162711301
 H   4.00042445028129     -2.75861014439376      0.57650329585009
 H   4.34992750602466     -4.30342462709655      1.16830493911934
 H   3.05526294372381     -3.49694495273522      1.79225554323536
 N  -0.80558925526720     -1.28231771175623     -2.62321317882477
 H  -0.11524112394810     -0.74953695972182     -3.16884046481437
 H  -1.67088651576839     -1.31401287791474     -3.16924783198779
 H  -0.96139879666600     -0.78049960364855     -1.72190406419411
 H  -0.44865805072008     -2.24547570605039     -2.42705410412228
 N   1.06030969052186     -3.69305636730641     -1.20560349443080
 O   0.23357190075653     -3.80165282442977     -2.12301257669080
 O   1.83540177934458     -4.62889168840427     -0.95452026235399
 O   1.11984768302919     -2.64088038247974     -0.54283970969551
 N   0.03968983561595      1.48643679098587     -4.36814734029678
 O  -1.18215042869917      1.35326584970193     -4.54762540523208
 O   0.52210403901078      2.61694603763083     -4.20452494898519
 O   0.78509180171050      0.49572082609017     -4.34597479804945
 N   4.22160070359712     -0.40094802467081     -0.57368973359687
 O   3.11650984917932     -0.06761013706845     -0.10365873542599
 O   4.82155543453107     -1.39034256655768     -0.13794608304444
 O   4.71450195183622      0.27750542594938     -1.48730353462258
 N   2.36886867115475     -2.73509793693354      4.24011950036706
 O   2.98743569404408     -3.27315034518525      5.17657449869589
 O   2.08104193057800     -1.53812238847451      4.29965431998285
 O   2.03823290593462     -3.41175611778727      3.24640455937110
 N  -0.24859289162120      1.03083202537840     -0.32089418411352
 O  -0.99963002054462      0.04582812182841     -0.18672290577360
 O   0.02809825473910      1.43411547291147     -1.46849715991169
 O   0.23769185808943      1.57907608932825      0.66918669270157
 N  -2.86889721819102     -1.29793188279422     -5.76653014092638
 O  -1.98865674099846     -0.41554280285032     -6.23600184129786
 O  -3.04348636066709     -1.33217055435050     -4.56314784290848
 O  -3.40976016231013     -1.98071294553382     -6.58423043036014
 H  -1.67265437722970      0.17130314902269     -5.47227485141036
 N   0.28074273892047      4.89179858927901     -1.85127704175507
 O  -0.67357922435831      4.34274895932717     -2.60744005791465
 O   1.42709217278459      4.54856186580209     -2.05309273135787
 O  -0.09538720578318      5.69347815469127     -1.04859795057427
 H  -0.23772704006591      3.67011707346418     -3.20802342051935
 N   4.50938225745032     -5.92289748159943      3.86514024957542
 O   3.70532942593136     -5.70378479184555      4.89423071803400
 O   4.82811139617965     -4.96622064199817      3.18450170358163
 O   4.85804677427393     -7.05821923025982      3.70019766044201
 H   3.43205060648893     -4.71322786984870      4.90152433181971
```

p=5 [(NH4NO3)p (HNO3)3(NO3)]- ωB97X-D3/def2-SVPD

```
 N  -0.24894782831889     -0.83130040718057     -0.14649202066942
 H  -0.10302376516711     -0.76463393916407      0.86199824142391
 H  -0.95458714622921     -1.56478576172833     -0.33716166752835
 H   0.63832057079226     -1.11275711470147     -0.60361400092539
 H  -0.58896397643511      0.08508601685884     -0.47421837938655
 N  -0.25731839388587     -5.63359090112844     -0.54433798211817
 H  -0.57670300462136     -6.31087167468272      0.14185993619587
 H   0.24818015557645     -4.85023282722179     -0.08024146030923
 H  -1.08590242731773     -5.24975939649154     -1.05936459432277
 H   0.37594973261691     -6.09772555677519     -1.23048354363796
 N   2.42457441162261     -4.09950676680010     -4.03646198670675
 H   1.82137627908528     -4.86203263536875     -4.39734098828902
 H   3.32745714979409     -4.08218803231863     -4.50526529714393
 H   2.54665923338949     -4.20247851557987     -3.00932113206593
 H   1.97960250304116     -3.18134008924321     -4.20973922580945
 N  -1.62922410972878     -4.15003526767881     -4.75463148790727
 H  -1.05177063042233     -3.40696272256673     -4.32655252240828
 H  -1.00501104689218     -4.95480108182273     -4.94526558352454
 H  -2.02537448296171     -3.75749222887005     -5.61901613562144
 H  -2.34182077100529     -4.40740903826961     -4.06315068999827
 N  -2.50252932511561      0.06276298534090     -3.08015540642348
 H  -2.77939181868665     -0.89075383443221     -2.79226279551467
 H  -3.01251362410527      0.76392159541217     -2.51345788421604
 H  -2.64923195389337      0.15565004812104     -4.09702741731477
 H  -1.49406275415682      0.17723303996968     -2.90285498609475
 N  -2.59910203570684      1.97786139650419     -0.38351372782095
 O  -2.75715188360845      2.50897916959359      0.73341140883558
 O  -1.45618764681564      1.65556627733464     -0.75850559110951
 O  -3.57508218944663      1.78410032055795     -1.11057257500179
 N   1.83783279366986     -3.23545275872238     -0.74796915925132
 O   2.33746396298441     -4.25391792516046     -1.25077385311316
 O   2.01440662404921     -2.12364809210496     -1.26391944828978
 O   1.13028014942729     -3.34426300144910      0.27388405974684
 N  -2.52945295041208     -1.36692380943361     -6.01191747909595
 O  -1.95730776656671     -2.18178732122949     -6.79997019993888
 O  -2.05726519972161     -0.23371518349639     -5.87190019940124
 O  -3.51530357246599     -1.74013727833898     -5.38981012312556
 N   0.48555112438446     -1.20847133802515     -3.52397431825932
 O  -0.35386136392924     -2.08933295055577     -3.27691435396546
 O   1.43121015417513     -1.47191398692123     -4.30105480162043
 O   0.39492243260156     -0.09577264259229     -3.01653348969849
 N   0.21770543625403     -6.56215625661881     -3.55624173773513
 O  -0.91097289536935     -6.95173439473249     -3.28246318978754
 O   0.45115264034454     -6.03363042907021     -4.67476716502821
 O   1.14642105259961     -6.63890852374123     -2.72865022271976
 N  -2.52136754944959     -3.35615923569041     -1.62804113438672
 O  -2.02369381950522     -2.98039376316072     -0.55656048528450
 O  -3.19372403507838     -2.60471366282405     -2.33145845215400
 O  -2.33258727737590     -4.53788129230306     -2.00376886712012
 N   1.22779144392252     -2.56914097848357     -6.89374336137796
 O   0.47049243252961     -1.59864343458630     -7.37675225233149
 O   0.67293869223249     -3.52075587463729     -6.38036975388893
 O   2.41427461180759     -2.42418058027484     -7.00515672844472
 H  -0.50634389923484     -1.81240302539936     -7.14551851013869
 N   4.64086038309322     -0.83428600021046     -4.36507488623276
 O   3.60159338922321      0.00208753835312     -4.38817108363317
 O   4.40764419123457     -2.01802367459420     -4.25458426422448
 O   5.71960278572515     -0.32173889195982     -4.45456587210215
 H   2.76267850341287     -0.54730354533297     -4.31869954010305
 N  -0.12442042685727      1.75087643569143      2.60087982966767
 O  -0.70496908546256      2.86487342305201      2.18681609201974
 O  -0.62637028563534      0.69409822496923      2.26367248987636
 O   0.85193406483345      1.88462300517410      3.28347640814103
 H  -1.49600520678698      2.62393552038948      1.57105211839852
```

p=6 [(NH4NO3)p (HNO3)3(NO3)]- ωB97X-D3/def2-SVPD

```
 N   4.30014389264856      0.61545462808930     -3.41830825440129
 H   5.03311881776731      1.30729072572952     -3.54411742978492
 H   3.80642719916147      0.77098938756518     -2.52079935849856
 H   4.73000871102578     -0.32660296243756     -3.40720002705741
 H   3.60150667145068      0.69428103972529     -4.19270770299631
 N   4.95903938820148     -2.94350069308022     -0.29693874364482
 H   4.20546349752905     -3.14873442338061     -0.96999123929503
 H   4.89085524369431     -1.94169052948369     -0.06695418091457
 H   5.85781934137225     -3.11648522407869     -0.78224495624801
 H   4.80291743356362     -3.53006685903567      0.54439667891993
 N   4.12749371770189     -2.82647187763568     -5.46886791745498
 H   4.16519214503473     -2.04546609433445     -6.13984083143281
 H   3.60641485105946     -2.48392572026103     -4.64476623561529
 H   5.09250743926932     -3.04500464011607     -5.15028503377694
 H   3.62680410690938     -3.64525003652148     -5.86173277207992
 N   0.14887994062055     -2.75647736176270      0.72464529673636
 H  -0.63727654230174     -2.19976296549744      1.07232117474998
 H  -0.21992965646753     -3.48005918153391      0.09130236583887
 H   0.69064678730587     -3.22791220931517      1.48342762107759
 H   0.78369988382164     -2.15046790013829      0.19215934834891
 N   1.35507380905044     -5.61260438150908     -2.01009725766091
 H   2.02991696653840     -4.82274228004080     -1.98692725440602
 H   0.40649915329103     -5.21062866528258     -1.91892147375490
 H   1.55015675789839     -6.21673359913033     -1.21529718970402
 H   1.44692037141871     -6.08728929610176     -2.93083667212025
 N   0.15945555518279     -1.72059356069280     -4.92818014185888
 H   0.47234110631086     -2.70390992761414     -4.88323469520011
 H   0.58284604935841     -1.28172972686048     -5.76608237974679
 H   0.53797727216517     -1.22857846051015     -4.11179998943181
 H  -0.86503186584076     -1.66886617295321     -4.92474448041507
 N   1.91442344811420     -5.26657642488751     -5.15712587465334
 O   2.61139093641598     -5.09010849018987     -6.16318182278167
 O   1.80419621333383     -6.39459795996821     -4.65518968365622
 O   1.33027616655172     -4.30146556818431     -4.61958293059929
 N   2.77527911284702     -4.33292105186574      1.60802522527465
 O   3.97285176975191     -4.33067855518899      1.92945253533335
 O   1.90415501884295     -4.02315487922956      2.43994707018602
 O   2.44147350942108     -4.62300050829835      0.44422167490893
 N   3.15767492069967     -0.19522565714111     -0.44053208763663
 O   2.59254380206094      0.68002261929874     -1.16325440392353
 O   2.50407224376397     -1.10315252968532      0.06059905234039
 O   4.37689363558684     -0.11345643973579     -0.28255297781293
 N   6.46426370245437     -2.72761229337840     -3.13233890175365
 O   6.62706290041718     -3.13142018271357     -4.29299963195826
 O   5.72688266206010     -1.74455114004677     -2.90699715102780
 O   7.01723866417776     -3.30070068509392     -2.18573135493464
 N  -1.89700275598722     -3.72185447586433     -1.65430145389145
 O  -2.56744803477827     -3.05127060715418     -0.86120337537417
 O  -2.03991483238263     -3.60021505620233     -2.87946211377771
 O  -1.07455771231245     -4.53755183876784     -1.20332918569685
 N   2.22291988169659     -2.52877397094292     -2.61563887130666
 O   2.56361873194958     -1.60772440915610     -3.38114824690592
 O   1.09067631370705     -2.55876601268866     -2.14359663667574
 O   3.04376742089423     -3.43505464682588     -2.36157322345623
 N   2.75051909841848     -0.08529744689335     -6.20005006238326
 O   2.29808651813711      0.71227355578329     -5.35747037204927
 O   3.98066827721948     -0.17365819004780     -6.36208546373421
 O   1.98443510749764     -0.81609441893208     -6.84392424855681
 N  -2.99646980684314     -0.37318280696673      1.04464689371190
 O  -3.45372787393162     -0.62187604629606     -0.18630696522084
 O  -2.29435140113342     -1.21025061951602      1.57062367477143
 O  -3.34803117213414      0.66655655178236      1.51864729596134
 H  -3.04639892629194     -1.46990439571545     -0.50111550395727
 N  -0.59656594941941     -0.20687863323415     -1.86827040166060
 O   0.44010782710512      0.33116169905925     -2.50029368866780
 O  -0.54033707632337     -0.29260140718851     -0.66534562971738
 O  -1.50244558617324     -0.56001961275603     -2.57454812273579
 H   1.21166492691808      0.40619842758597     -1.83963677059839
 N  -3.74253141750105     -1.34057882447320     -4.62651228199447
 O  -3.96333895046284     -1.96837034003758     -3.47963713106452
 O  -2.73191368406897     -1.62673241085825     -5.24314917839495
 O  -4.57598047119934     -0.55324139294649     -4.96452181020896
 H  -3.16829120384173     -2.56823995623901     -3.28153713189223
```
