# Supplementary material for: Decomposition and Growth Pathways for Ammonium Nitrate Clusters and Nanoparticles
Source: J Phys Chem A. 2024 Oct 14;128(42):9184–94. doi: 10.1021/acs.jpca.4c04630 (PMC11514028; doi:10.1021/acs.jpca.4c04630)
Supplement: Supplementary file 2 — jp4c04630_si_002.zip [file jp4c04630_si_002.zip › SI_ammoniumnitrate particle structures_PDF_XYZ/HassanAmatTopper_SuppMats_S11.pdf]

Supporting Information for:

Decomposition and Growth Pathways for Ammonium Nitrate Clusters and Nanoparticles

Ubaidullah S. Hassan, Miguel A. Amat, and Robert Q. Topper*

Author Affiliations:

Ubaidullah S. Hassan, Department of Chemistry, The Cooper Union for the Advancement of Science and Art, New York NY 10003, United States.

Miguel A. Amat, Department of Chemistry, The Cooper Union for the Advancement of Science and Art, New York NY 10003, United States.

Robert Q. Topper, Department of Chemistry, The Cooper Union for the Advancement of Science and Art, New York NY 10003, United States. Email: topper@cooper.edu; Phone: 212-353-4370.

Table S11: Cartesian Coordinates of p=(1-8) $[(NH_4NO_3)_p(NH_4)]^+$: ωB97X-D3/def2-SVPD

p=1 [(NH4NO3)p (NH4)]+ ωB97X-D3/def2-SVPD

N	-3.01995264060671	0.00007895790124	0.32094814432510
H	-3.60487074940036	-0.83307014717935	0.24393611351967
H	-3.60415198640116	0.83371551660193	0.24382842190612
H	-2.24346171308170	-0.00028508225783	-0.44570238294638
H	-2.53778365726380	-0.00009535713078	1.22255657779830
N	3.02027881111363	0.00010661489948	0.31780582814622
H	3.60497863775445	-0.83313343326273	0.24013951693836
H	2.53905395437815	-0.00003176675513	1.21992210532950
H	2.24299116219469	-0.00010419480543	-0.44803723060485
H	3.60454023944074	0.83365059287517	0.24011692490348
N	-0.00040256120220	-0.00021354110589	-0.78170393873732
O	1.07015321713352	-0.00013956507568	-1.41515982029575
O	0.00025170988511	-0.00022034697901	0.45538422908174
O	-1.07162342394434	-0.00025624772599	-1.41403448936421

p=2 [(NH4NO3)p (NH4)]+ ωB97X-D3/def2-SVPD

N	0.54942867291397	-0.77228084293733	1.49534759075358
H	0.42788200433816	-1.34158234024452	0.62225565767286
H	0.58811302006353	0.23067861355611	1.18832336332957
H	1.42002739499294	-1.02103863301269	1.96349343839822
H	-0.24404576476093	-0.92371500281244	2.11688028005886
N	-1.66166183709353	2.01165139131600	-1.81524041170115
H	-0.62668204949998	2.15993643778165	-1.90506616067953
H	-2.10944399593923	2.06492103477554	-2.72938658208114
H	-2.05430984679547	2.71062818271904	-1.18572494481131
H	-1.78627314016951	1.04906024516818	-1.41616067910068
N	2.20441924712965	-0.64267119769450	-3.07870563076762
H	1.16036176704191	-0.74515721523639	-3.10913936870999
H	2.64106355647816	-1.56317001031659	-3.10994091018687
H	2.42458017956310	-0.16235092892542	-2.17197863042203
H	2.52760437813178	-0.06951874336627	-3.85723502869634
N	-0.57757210104423	-0.99734025183590	-1.50063393734591
O	0.26257891968959	-1.79046740258358	-1.04438929846883
O	-0.51898937037393	-0.64680748989966	-2.69119851917221
O	-1.44973930866154	-0.51992109889884	-0.75553191190072
N	1.31039834765057	1.39325642044688	-0.75775581768192
O	0.56948177023606	1.65629104541148	0.20426424901539
O	2.24606917743212	0.58783448693477	-0.61858326439203
O	1.08842397867680	1.90115729965446	-1.86959148311018

p=3 [(NH4NO3)p (NH4)]+ ωB97X-D3/def2-SVPD

N	-0.69609607871509	1.77496725181934	0.16447393066611
H	-0.27637296498278	2.70249807117597	0.18513594725312
H	-0.28366209961085	1.16821832309664	0.90264203164075
H	-0.53739824741868	1.31974001809974	-0.75768374331584
H	-1.72205939059926	1.83105547141580	0.32674883043645
N	-4.37462023017150	-0.80881274727033	2.39172996325043
H	-4.42391349843069	-0.77814248763273	1.34269174374125
H	-5.11908535293821	-0.23371536544845	2.78287805473714
H	-3.43688123789087	-0.41429796690856	2.66748238297803
H	-4.45213295820197	-1.76573954463199	2.73266108321178
N	0.66586519879040	-2.99622935771511	0.93032258736754
H	-0.15364893682899	-2.66775672660438	1.49948282143194
H	1.47836426344724	-3.10219284543167	1.53578873227014
H	0.85498108951302	-2.25435461898273	0.20515774029869
H	0.45581228049662	-3.87793565451934	0.46505367415447
N	-2.99575956676393	-1.26710305149707	-3.09627533081453
H	-2.15996282501817	-1.68184708845457	-2.61389101144167
H	-3.21489860268157	-0.36026375953794	-2.60536402443134
H	-3.80028323938441	-1.88865805452934	-3.03034275593515
H	-2.76943523643658	-1.08539044897888	-4.07280641491198
N	-1.10646210639080	-0.65883375414390	2.35371347153519
O	-0.00249517906319	-0.31753235746041	1.90517785157868
O	-1.44942348296329	-1.85976937499924	2.30690099910007
O	-1.87925228113862	0.18572296922895	2.82079205916588
N	-3.69892341394202	0.56862253504349	-0.48675452515833
O	-3.38222697354543	0.97675067824800	-1.61013646781777
O	-3.43596005341287	1.24031002082838	0.52062953795043
O	-4.26210858529456	-0.54040624228906	-0.36177020926279
N	-0.13776132385360	-1.00290334578597	-1.54108371095374
O	-0.75042489023448	-2.08147908019338	-1.69361074149091
O	-0.58975746452098	0.03597468027478	-2.04318344001156
O	0.89979438818622	-0.97211614621602	-0.86898406722246

p=4 [(NH4NO3)p (NH4)]+ ωB97X-D3/def2-SVPD

N	-4.93661531691588	2.29602581162233	-0.44799803247936
H	-4.04997325728311	2.39835486402209	0.09961508906934
H	-5.41321444166257	1.41722135645043	-0.14420623095562
H	-4.69277326024678	2.18572196172748	-1.43615164839579
H	-5.55045055221142	3.09663334111393	-0.31368190552806
N	0.18285877407438	1.12169606950263	-0.19448884595780
H	-0.40424142742606	1.56736152809667	-0.91984966252534
H	0.57177695921163	0.26727048981850	-0.62107900356590
H	-0.43291412027865	0.86101744093408	0.62079271851579
H	0.91785701670775	1.75825595867148	0.10516226202866
N	-3.50382136123613	-1.96417209949181	0.97198699253694
H	-3.67545521812294	-1.05150768469029	1.44871292978621
H	-2.62264983243236	-1.89227033317448	0.44055822693117
H	-4.26831067626179	-2.12615104813013	0.28321980947289
H	-3.44720368460778	-2.71655186130949	1.65473506622099
N	-4.11209523579975	-1.13575376565975	-3.90527682446279
H	-3.54471629150089	-0.35580092770144	-4.28565220121514
H	-3.49575864692038	-1.66727800209767	-3.27652882034934
H	-4.47145188020321	-1.74058413866957	-4.64005774441567
H	-4.89970652452416	-0.74435128174967	-3.32749078132896
N	0.33143232578415	-0.14594039355750	-4.63968826151311
H	0.42523656764493	-0.87866006238916	-5.34081591447052
H	0.44066280898286	-0.55697347488430	-3.67195287951263
H	-0.62576377950275	0.28663045365698	-4.67594380697457
H	1.03162410140347	0.57756279582453	-4.79566475248205
N	-0.80822506641916	-1.43323325087879	-1.84986727776559
O	-1.52865919110031	-1.73772202115885	-2.80463682502255
O	-1.20803663065889	-1.46616031824508	-0.68774264610430
O	0.37222381514643	-1.05088659837364	-2.07137977480887
N	-5.65247642156215	-0.76162096745667	-1.13412176456316
O	-5.90664185688311	-0.09010893404618	-2.15104660787365
O	-5.84908662888596	-0.28496571340946	-0.01014905825483
O	-5.16397991010263	-1.90041185916856	-1.25192457612530
N	-2.09834322683987	1.26155942164340	-3.12398372432338
O	-3.08221371507191	1.21379575372770	-2.39310715552914
O	-0.99632848095725	1.64401520012020	-2.71381074852228
O	-2.19472251047276	0.88307780425595	-4.32421805796860
N	-2.60491371677345	1.09798459654661	1.45573656942821
O	-3.72942747897238	0.68681175247412	1.76903369104983
O	-2.48281912085132	2.17519119212811	0.84506793545767
O	-1.59899393200920	0.41592701464151	1.71793114623467

p=5 [(NH4NO3)p (NH4)]+ ωB97X-D3/def2-SVPD

N	1.89917852818857	1.12812370583047	2.24077225858376
H	2.28381069853681	0.45191749184524	2.91247328428220
H	1.32529845517167	0.59070980804046	1.56333917370840

```
H   2.71334453354143     1.56535702974384     1.74343648304099
H   1.35615724495958     1.83994081136322     2.72378168266418
N   5.42421605042206    -0.10134180077080    -0.87861425923393
H   4.39545598796827    -0.14913126348719    -0.74290190881674
H   5.64460265307782     0.43866722121743    -1.71211205638787
H   5.76653598163112    -1.07340841695509    -0.95007287219962
H   5.83841223933063     0.35539848505819    -0.03302750875674
N   5.99479461940946    -0.95036346213436     3.83269830501607
H   5.31918640305407    -1.56195384769702     4.32811257042276
H   6.88194585552293    -0.87719069970396     4.32425111696170
H   5.57666307533741    -0.00259557413491     3.69344311347459
H   6.13251899086704    -1.37601843413481     2.89762310901538
N   4.04724450245625    -5.43243214514849     3.46701460817958
H   4.00251477742203    -4.62171823181212     4.11187082827003
H   3.08017915884189    -5.68266786338253     3.14338469492198
H   4.49464444215108    -6.23740355685210     3.89955919324501
H   4.57400162097992    -5.12733008929046     2.63841517099299
N   3.50293739768402    -4.64369104903948    -1.39797317778995
H   3.40761901023416    -5.39828401113951    -0.67895770998137
H   2.67418266087204    -4.04713922025146    -1.31630147662505
H   3.56221019351671    -5.04459703381611    -2.33114911852937
H   4.33544917939796    -4.05253750939147    -1.17264649179899
N   0.05766413073219    -3.32577414538564     1.68897677334028
H   0.75533933185891    -3.27409157956132     2.45517670983994
H   -0.87417732651219     -3.46591184806960     2.07181435967374
H   0.31884898480045    -4.12815362855953     1.07512441504713
H   0.13006255231834    -2.43345929295538     1.16107794803000
N   5.50549985615823    -2.93437241230375     0.65593746169289
O   4.87189393844971    -3.80192481749733     1.25762837596694
O   5.52140516139786    -2.93705832957587    -0.60027351182658
O   6.12075446375658    -2.04182083766366     1.24568301597232
N   3.04081594892025    -2.41815448762503     3.77751467316881
O   4.00510475749229    -2.79771166120281     4.48565065562793
O   2.09105866304656    -3.19054123630628     3.61799214774614
O   3.07192830132500    -1.30435243463260     3.25047863969603
N   5.05680613000426     1.46649531954937     1.92031002146406
O   4.74308896612878     1.41063877075250     3.12236783094968
O   4.25947743185690     1.93619427308448     1.09266855217025
O   6.15052645825589     1.01601100316598     1.54401715355581
N   1.93484880995416    -5.87415982540602     1.08283446249104
O   1.61204887226138    -5.86520727868130     2.28134408642895
O   1.16270829538982    -5.40564937393869     0.22931988231271
O   3.04364221347445    -6.31817905054399     0.74034415211431
N   1.89917875029672    -1.17023342035534    -0.01281422570936
O   2.12541307798669    -2.32934920035244    -0.34985480610003
O   2.70497866580649    -0.25641538005398    -0.22405289816149
O   0.82700887566495    -0.89041443903855     0.58145296844837
```

p=6 [(NH4NO3)p (NH4)]+   ωB97X-D3/def2-SVPD E -2080.055384932274

```
N    -5.904128    6.420798   -0.064042
H    -6.669996    7.057358    0.144332
H    -5.947786    5.601159    0.549709
H    -4.978022    6.874613    0.063964
H    -5.983368    6.066593   -1.049247
N    -1.503850    5.965497    2.152618
H    -0.637401    5.633495    1.688676
H    -1.270427    6.473987    3.002516
H    -2.099007    5.140144    2.385460
H    -2.042860    6.562092    1.492967
N     0.448090    0.866810   -2.371062
H    -0.329886    0.531909   -1.767437
H     1.133150    0.137001   -2.553088
H     0.892969    1.660379   -1.899354
H     0.058809    1.243689   -3.266590
N    -6.144380    1.595409    0.176284
H    -6.631040    2.444835   -0.214270
H    -5.397223    1.365205   -0.486263
H    -6.790881    0.816813    0.277678
H    -5.677920    1.833920    1.078611
N    -1.933227    1.028606    2.082561
H    -1.283176    1.845366    2.029687
H    -1.627801    0.386244    2.809990
H    -1.922768    0.574633    1.148864
H    -2.890332    1.375423    2.283630
N     0.917718    5.645656   -1.914032
H     1.597143    6.384823   -2.076892
H     0.958482    5.303859   -0.929217
H     1.071294    4.840471   -2.574800
H    -0.036246    5.997107   -2.056073
N    -3.332144    4.118140   -3.437915
H    -4.251994    3.963010   -3.854463
H    -3.376885    4.976452   -2.861924
H    -2.565459    4.220782   -4.121367
H    -3.101567    3.303176   -2.840926
N    -4.138953    3.425350    2.196163
O    -4.534663    2.242492    2.340376
O    -4.799575    4.223842    1.538217
O    -3.063535    3.745617    2.721936
N    -2.474284    1.377634   -1.066815
O    -2.230849    1.880910   -2.164234
O    -1.762829    0.422625   -0.663695
O    -3.388744    1.795342   -0.352627
N    -2.749369    6.565806   -0.827789
O    -1.592662    6.136207   -0.842945
O    -3.187512    7.105366    0.220676
O    -3.492779    6.461753   -1.803271
N    -0.064722    3.443708   -4.088060
O     1.039726    3.452797   -3.492138
O    -0.527026    2.371801   -4.490364
O    -0.684795    4.503687   -4.237275
N    -6.291178    3.930327   -1.990494
O    -7.006139    3.875285   -0.961182
O    -5.874763    2.901402   -2.517789
O    -5.985990    5.047098   -2.447012
N     0.260812    3.584876    0.781203
O    -0.242038    3.209172    1.850335
O     0.341000    2.839490   -0.191301
O     0.673221    4.768555    0.710283
```

p=7 [(NH4NO3)p (NH4)]+   ωB97X-D3/def2-SVPD

```
N   -2.13548806540998      0.85463107731885     -3.13307432744114
H   -2.40201380608065      1.55747129754429     -2.40800706506741
H   -2.79876425114513      0.86550048921339     -3.90442768575194
H   -2.10194917346967     -0.09494662756279     -2.69347426890315
H   -1.20014415668296      1.13523880126593     -3.45816833080666
N   2.20085036604746    -2.66369941067712    -0.07798697146599
H   2.45106467753797    -3.62877453629691     0.12642615560191
H   2.25501064178240    -2.10005436474688     0.79573954348667
H   1.23379166196238    -2.63043422019030    -0.45691316324653
H   2.86335616395359    -2.26898639868260    -0.77655970821017
N   3.99891577812145     3.23413802539166    -1.67651925782688
H   3.69098082525575     3.49158664951446    -0.72382166323394
H   4.10024922473623     2.20320642595385    -1.78312502062787
H   4.87359421807866     3.69915009801286    -1.90793425232146
H   3.24213977922694     3.55845836850296    -2.31003460617247
N   4.11153298775500     1.17026778056016     1.86214513668566
H   4.33410034938827     0.94641042718233     0.87839177515735
H   3.57260848986948     2.05672069898794     1.95213982127150
H   4.96424284872866     1.22156680593340     2.41459670502684
H   3.52401335471158     0.37421583383400     2.18730426846431
N   -1.82269762312884    -1.22929934284296     1.17568536436604
H   -2.58568803275643    -1.69939944545305     1.65726378079902
H   -1.45937665180932    -1.83836916862265     0.41773957732971
H   -1.03777200121895    -1.05530890661515     1.82461372403748
H   -2.16365900478147    -0.34261562247238     0.75807837163508
N   1.97081318085768    -0.66264276472125    -3.88271576886716
H   2.06095783860011    -0.97923628278712    -4.84502136380021
H   1.11021127767822    -1.04691805807288    -3.46246870344186
H   1.96164231103492     0.37805736402774    -3.84820145651252
H   2.77438242209198    -1.00471305458850    -3.31327734172392
N   -0.30331205178371     4.66591033422432    -0.95421781709412
H   -1.10728876334547     4.00558064902880    -0.98978551736220
H   0.37636420131095     4.41539538625750    -1.70134576550383
H   0.16762887343083     4.61348616243406    -0.02569071693256
H   -0.63819743171601     5.61524658176671    -1.10526331716149
N   -0.32176383081820     2.42974987855106     2.97812118751704
H   0.22108478845502     3.22743435230862     2.58576319905654
H   -1.05635389178014     2.22116702979617     2.28306271205303
H   -0.74097966090090     2.69813447377255     3.86533252067237
H   0.30795470364883     1.61521940234809     3.11218035512577
N   2.12473387243439     4.11841651489905     1.43964580180925
O   2.61488471348733     3.42659055075586     2.33720190698377
O   2.78413326816158     4.49297476042752     0.47161847288636
O   0.91750099137047     4.44742754742265     1.53110896978636
N   4.24609225367809    -0.41630044128249    -1.56149252953426
O   4.50890832368122    -0.34849986799191    -0.36004726543285
O   3.83786687849029    -1.50418729331736    -2.03593307091564
O   4.36799345502855     0.55348980124056    -2.31239982691029
N   1.45302544994278    -0.67982307829633     2.79448100254913
O   2.48444113417994    -1.05053223452358     2.18279037243615
O   0.43938226167990    -1.37491450745380     2.75565388035798
O   1.49515549841181     0.38025348344027     3.42588679372940
N   -2.50159355720636     2.15990297259091    -0.06454430774813
O   -2.92344301317557     1.00168570000162    -0.09430643025054
O   -2.36584484650981     2.77898035053119    -1.14988290254425
O   -2.21269281405478     2.72536295580973     0.98862465065829
N   1.12274104034647     0.82701806812710    -0.28605323410417
O   1.94986894754251     1.75326971798856    -0.25360333305982
O   0.19467947315294     0.79488461307874     0.53972011989765
O   1.22626423619164    -0.06965613337198    -1.13865018947643
N   1.25459520510820     2.81340399311816    -3.35677352338589
O   2.12054552282697     2.10053805962519    -3.86941846401856
O   1.59043646670267     3.92364545926308    -2.88263148193166
O   0.07522091038145     2.46043784543574    -3.28829849904163
N   -0.89376404372988    -2.10067535985976    -1.85862654844788
O   -1.98544205697485    -1.51582679366527    -1.79544040910829
O   -0.22648458685773    -2.09139469568341    -2.89190827168808
O   -0.49048455172697    -2.70800717770947    -0.84415679230679
```

p=8 [(NH4NO3)p (NH4)]+   ωB97X-D3/def2-SVPD

```
N   -2.67153916536794      2.91728046954004      0.44193839093913
H   -3.30440405758911      3.64981261737228      0.10801365570948
H   -1.89484847866617      2.71928338541490     -0.20717507491155
H   -3.19764777423176      2.05507702134256      0.63437626992473
H   -2.26544267568633      3.23638351089956      1.34632635319452
N   -1.35576119582012     -1.00614538979563      2.58295941976544
H   -2.40183833709745     -1.00147927254666      2.61741136376067
H   -0.99293187883700     -1.95626355410074      2.57889846523456
H   -1.03210907893287     -0.48944638363912      3.41626623065716
H   -1.05063376847670     -0.48604722241115      1.72629338838495
N   -6.15182844692425      3.54563571603161      2.99762061382054
H   -5.87663874807148      2.54523006735271      2.94528349598726
H   -5.97047821961712      3.88708214281678      3.95917539908878
H   -7.14204387958926      3.63570833522393      2.78388778134951
H   -5.58586064087339      4.10013710460972      2.32053631386776
N   -0.51098061489236      6.93001642932163      2.11353886439104
H   0.27511189851301     6.33112824856836     1.78881086662928
H   -1.41706437864359      6.58991117881039      1.72047206610916
H   -0.35015710952900      7.89126865490924      1.82100526922933
H   -0.53883297378621      6.88654652658985      3.15126776905830
N   0.96152863729476     3.51223045440489     4.00437780614529
H   0.86386644150986     2.79002553530317     4.73305681602627
H   1.65160033185325     3.28379104681977     3.27214184586045
H   1.18824980576115     4.40495588280851     4.45479098857069
H   0.03936453724058     3.64341101454687     3.54819836598360
N   -3.71939319443065      6.55352320297955      5.49306647466257
H   -3.47539305819137      6.53064737894926      4.49447016290192
H   -4.31941343393776      5.71985239955223      5.68753062002384
H   -4.23152740337305      7.40950152906080      5.69298191223519
H   -2.84243430923110      6.52038974238716      6.05610249409194
N   -1.59176700222350      3.64717769882499      7.70771767564956
H   -0.88517630046545      3.01804762962761      7.27855952172484
H   -1.34963428022377      4.63754092607799      7.48659366611907
H   -1.63003404998282      3.49589775965197      8.71244766252305
H   -2.51520516302712      3.44763879702122      7.29018340497119
N   -4.29722210923271      0.07796546617948      5.75393217234981
H   -4.61959657957111     -0.60932583087065      6.43142966134939
H   -3.31880434684945      0.34846338658719      5.96945880533742
H   -4.91309357044391      0.92170251931306      5.79630262520935
H   -4.32868593776026     -0.31817404340177      4.79120514072388
N   2.80726050449002     2.27506994147549    -1.23919312878929
H   3.78586247189247     2.10192602519199    -1.01769225197902
H   2.19566516459776     1.56069509614183    -0.76454068640905
H   2.66226543043878     2.22970942716322    -2.24593555649981
H   2.52962704329050     3.22475172700579    -0.87146612938084
```

N	-3.78393119916784	5.66221931220315	1.58835307217012
O	-2.79030591086790	5.96023863231016	0.91160668796210
O	-4.62827421251579	4.88363734321140	1.10300452906173
O	-3.94143595991485	6.10931801809757	2.72923874875606
N	2.02330652059890	4.39729635194100	1.08783889415602
O	2.10216470894467	4.65793068605521	-0.12026995440379
O	2.37580237358455	3.28489857869778	1.51328962979127
O	1.58917497440690	5.24582685759683	1.88458085739293
N	-2.38903731037345	3.55153987749006	3.84107267768942
O	-2.08434197758942	3.94105084028742	4.97059067121481
O	-3.40828738429380	2.90023779971680	3.63253594107067
O	-1.62031424084060	3.83282616321555	2.87686742498624
N	-0.00628372162931	1.10456537769751	-0.09284083036241
O	1.08449202137605	0.51748822960239	-0.03859463082834
O	-0.12232144692911	2.16054938304799	-0.73095451057031
O	-0.99645702099388	0.63897993375040	0.49712429336905
N	-5.18963110285089	3.32187042101165	6.15848500463160
O	-5.84314399520678	2.34338564744106	5.77717602530212
O	-5.41728328173944	4.42963227961647	5.61669637454892
O	-4.34297606064291	3.21771679257838	7.04347478576839
N	-4.56711717216567	0.25949710794007	2.47575777099330
O	-4.12554160507728	0.59368368252083	1.38320597295913
O	-4.01840279737117	-0.69250348581152	3.10229323229007
O	-5.53584871432521	0.81282041303154	2.99972814569715
N	-0.77068029414558	1.20334239218272	5.74244523718901
O	-0.69473845129052	0.88098761999037	4.54994149405703
O	0.09147690196439	1.96651184330483	6.22213706004688
O	-1.69116602247474	0.80410701031711	6.46484554609072
N	-0.52342003164936	6.27200603073253	5.82346029400342
O	-0.85317844250254	6.97029530300608	4.85369631912957
O	0.51398935702149	5.62691124138721	5.84311914367860
O	-1.30385760664405	6.23571601671587	6.81264912056553
