# Supplementary material for: Decomposition and Growth Pathways for Ammonium Nitrate Clusters and Nanoparticles
Source: J Phys Chem A. 2024 Oct 14;128(42):9184–94. doi: 10.1021/acs.jpca.4c04630 (PMC11514028; doi:10.1021/acs.jpca.4c04630)
Supplement: Supplementary file 2 — jp4c04630_si_002.zip [file jp4c04630_si_002.zip › SI_ammoniumnitrate particle structures_PDF_XYZ/HassanAmatTopper_SuppMats_S10.pdf]

Supporting Information for:

Decomposition and Growth Pathways for Ammonium Nitrate Clusters and Nanoparticles

Ubaidullah S. Hassan, Miguel A. Amat, and Robert Q. Topper*

Author Affiliations:

Ubaidullah S. Hassan, Department of Chemistry, The Cooper Union for the Advancement of Science and Art, New York NY 10003, United States.

Miguel A. Amat, Department of Chemistry, The Cooper Union for the Advancement of Science and Art, New York NY 10003, United States.

Robert Q. Topper, Department of Chemistry, The Cooper Union for the Advancement of Science and Art, New York NY 10003, United States. Email: topper@cooper.edu; Phone: 212-353-4370.

Table S10: Cartesian Coordinates of p=(0-8) $[(NH_4NO_3)_p(NH_3)(NO_3)]^-$: ωB97X-D3/def2-SVPD

```
p=0 [(NH4NO3)p (NH3)(NO3)]-  ωB97X-D3/def2-SVPD
 N   0.15237301533715      1.26938755374143      1.01060376178298
 O  -1.00137836670200      0.86890383434421      1.25036429146711
 O   0.36115142055624      2.46308353008358      0.74840592628131
 O   1.10637916756603      0.46046918903861      1.03495441696874
 N   0.00762389686585     -1.91515119851528      2.59909207309882
 H   0.31832176103713     -1.69782582495112      3.54319124060852
 H  -0.95192692068891     -1.57739822223034      2.55240520529033
 H   0.50096802602850     -1.24545686151109      1.99617108450217

p=1 [(NH4NO3)p (NH3)(NO3)]-  ωB97X-D3/def2-SVPD
 N  -1.62044512271979      0.09756981536800      0.71891404351266
 H  -1.96472774820944     -0.63560923649215      1.38480727584631
 H  -0.64762423778098     -0.04583190850890      0.34263812011389
 H  -1.69915159056111      1.00876939180268      1.20683319978020
 H  -2.19712860737646      0.12600872539081     -0.12098087054172
 N  -3.61080375563751     -1.13740894513012      3.17263967588016
 O  -4.17109671685250     -1.71541128865772      4.09450051298009
 O  -2.59779209223125     -1.64463831161644      2.62847867701637
 O  -4.03154946820270     -0.03620206361360      2.75297740819025
 N   0.34591747390109      0.27240241040073     -1.82205787121702
 O  -0.86595751364388      0.46848880443194     -2.03354218035151
 O   0.71669664781708     -0.05591495408348     -0.66003727640299
 O   1.17604175835152      0.39309218396244     -2.71527000077232
 N  -2.29036402716335      2.30400659656893      2.52040279008857
 H  -2.79846791608610      3.14451300599045      2.26509579379288
 H  -1.72005534350246      2.52082873147304      3.33139929842474
 H  -2.97472274010214      1.58850704271337      2.79087640365942

p=2 [(NH4NO3)p (NH3)(NO3)]-  ωB97X-D3/def2-SVPD
 N  -2.39530740954283      1.81348143260093     -2.89693023781373
 H  -2.14227077173224      2.12628622948117     -3.85363984909455
 H  -3.27954065491069      1.30081240708144     -3.00179503040467
 H  -2.63766365259158      2.66144331630622     -2.35207787352911
 H  -1.60097802968998      1.28639668845266     -2.45861145705212
 N  -0.68269852397786      4.66679103995053     -3.20529189949246
 H   0.10851799659563      5.30250799437457     -3.25856049223207
 H  -1.20882489146175      4.70215427709475     -4.11364604704352
 H  -1.31807399638295      4.98121427597631     -2.42216297645092
 H  -0.30804111288195      3.71778777930461     -3.00674387772732
 N   0.81371513843663      1.27909050209227     -1.99879989983434
 O   1.90825975692030      0.87554383536799     -1.64606702469007
 O  -0.22054567283816      0.60812900431741     -1.76809145802672
 O   0.70379644260255      2.37294697749485     -2.60480818869548
 N  -3.41619682867513      5.08095472542457     -1.19169991215912
 O  -4.31409920005491      5.61594388701374     -0.56858140477491
 O  -3.63476143083163      4.05224317423872     -1.87830220409839
 O  -2.24983196088340      5.54410875245514     -1.16631493156326
 N  -2.71692927116239      3.81578154927234     -5.72566015985419
 O  -2.27525575748668      4.94811415009138     -5.43513947640864
 O  -2.01828749469619      2.81203985614432     -5.44505985782980
 O  -3.80928395300768      3.68567144465720     -6.26483950045138
 N  -5.11291732215664      2.07244784958811     -3.78953082198600
 H  -5.12236578647463      2.87994720270748     -3.16591813026168
 H  -6.06957510262114      1.74811250297550     -3.88124611072381
 H  -4.82252751049433      2.41791014553526     -4.70590217780124

p=3 [(NH4NO3)p (NH3)(NO3)]-  ωB97X-D3/def2-SVPD
 N  -3.89705666386494      1.80609754216251      2.39554275416786
 H  -3.19421477576561      2.44355913614096      2.78176145281686
 H  -4.03375185590449      1.03089978490494      3.06934842489343
 H  -3.51748145566610      1.46417616934351      1.49581072574753
 H  -4.76034482766554      2.35456100048957      2.21343779208569
 N  -0.00088644012927      1.63459183212074      3.62908227147371
 H  -0.92593200610530      1.52872854868266      4.09210108422744
 H  -0.09766338818472      1.25116515730376      2.67070946467535
 H   0.65922250164126      1.07993342450856      4.19906490732085
 H   0.21173292207115      2.64768452011542      3.56654001869889
 N  -2.23503805610630      4.28521649287079     -0.53220648606617
 H  -1.61171149433499      3.45320389208034     -0.59677246184290
 H  -2.22565441189356      4.81148433195017     -1.40018762447103
 H  -1.87153042196211      4.86839589691963      0.25046866176755
 H  -3.21925760725974      3.96680394713395     -0.32078908544016
 N  -1.22176949606421      1.31295020905810      0.32601738873200
 O  -0.79878747307234      0.53902101868580      1.19949470152490
 O  -2.42053899963319      1.29382640250898      0.00336789080427
 O  -0.45238397939438      2.12047652759460     -0.22506888146235
 N  -5.36226164685460      4.19976317833988      0.81459540307197
 O  -5.90059247477986      3.74706906862756      1.84078969494541
 O  -4.69860906565133      3.40192073138319      0.09495391159357
 O  -5.45225593301209      5.38013987970211      0.50428789004173
 N  -0.78098900948154      4.49889194919580      2.33274828479197
 O   0.15261103921976      4.39965718895010      3.14239543688916
 O  -1.64288350524188      3.60194425473439      2.25935959345834
 O  -0.85736540644966      5.48281656516790      1.58082749065252
 N  -2.75026883980523     -0.06128667085531      4.84274763705327
 O  -2.40229139774386      1.14502490801927      4.90186525954672
 O  -3.80744116637700     -0.35279513991876      4.26597329626056
 O  -2.04140536505491     -0.93378058590327      5.34990671640563
 N   0.90621231208252     -0.32592347215024      5.57258561602400
 H   1.39422959647102     -1.18402440725463      5.33394407691623
 H  -0.09545621947268     -0.54842599843336      5.61645897065537
 H   1.19840901144594     -0.06089928417980      6.50822072203957

p=4 [(NH4NO3)p (NH3)(NO3)]-  ωB97X-D3/def2-SVPD
 N  -6.07424195176003      1.02324756446861     -0.76749117074154
 H  -6.07102082566718      0.71639490153971     -1.78062655546990
 H  -6.58636056456880      0.35578326847524     -0.14758965624468
 H  -6.53018977769636      1.93060726960842     -0.71536174559196
 H  -5.10356148022344      1.11829609205520     -0.39346234565523
 N  -4.00863624586260     -1.32194866454567     -1.63563056989162
 H  -4.70005816373627     -1.42091291036579     -2.41527361571056
 H  -4.49767404061491     -1.38333272078782     -0.73046184466691
 H  -3.53246711903627     -0.40827378362916     -1.68152357479740
 H  -3.27138203449929     -2.05254259432553     -1.64355133891334
 N  -3.06329274821982     -0.59370198170240      2.64445818494142
 H  -3.98781507644726     -0.98430668491449      2.89291201089339
 H  -3.22774203118498      0.20159748390835      2.00368338820206
 H  -2.52074882084151     -1.28053253486126      2.11156586461971
 H  -2.52769925405383     -0.28163669437294      3.47962935369221
 N   0.63548857114062     -0.10250531146571      1.06190847306513
 H   0.26902927121569     -0.38831566057486      1.99299876636924
 H   0.64642916976884     -0.95045863429304      0.46062049006788
 H   1.55880897293864      0.32780240574380      1.24520827921302
 H  -0.03230791412776      0.57189505381468      0.66087144722829
 N  -6.36780841807119     -0.47177801811412     -4.04189883723605
 O  -6.98822244072187     -0.42837251488471     -5.08598891281536
 O  -6.13670181242200      0.58833302565947     -3.39467954915982
 O  -5.93978399045060     -1.55439466116585     -3.58497557646711
 N  -6.08248401134136     -1.03952126358326      1.71572529994284
 O  -5.23841712770113     -1.49120514141400      0.91640461134314
 O  -7.11274518224978     -0.49274375410909      1.28302013969955
```

O	-5.87186207289956	-1.11961690074624	2.93030948226833
N	-2.53640658873284	1.35149393942822	-0.12827800370811
O	-1.51650719616906	1.65374924581389	0.49066740837883
O	-3.61773008588520	1.21887136924237	0.51064170287650
O	-2.53304763845927	1.17327418397313	-1.34690798617351
N	-1.10794174558500	-2.29032031355697	-0.54896601581829
O	-1.72255973299359	-2.93911807818253	-1.40435456013995
O	0.12866226557693	-2.36364343604672	-0.47605302882178
O	-1.73413237235298	-1.54576529252408	0.23554037717514
N	-0.36021293547726	0.23520063925456	4.32556615747867
O	0.60819908823904	0.93368624528667	4.62424114602381
O	-0.20892499637463	-0.77626171194497	3.59303791926049
O	-1.50056852519158	0.51349320961253	4.72661215466387
N	2.81864549265499	1.08836165607494	2.54960928181250
H	3.07563742571789	2.06860846516992	2.48187905852397
H	3.66053476243910	0.56915333684475	2.77927637992919
H	2.17621590192783	0.99762990613679	3.34453951035386

p=5 [(NH4NO3)p (NH3)(NO3)]- ωB97X-D3/def2-SVPD

N	-4.37510697150117	-6.70577357823078	5.44770401301989
H	-4.58448776827585	-5.70777800857694	5.23191167114891
H	-5.03084984061115	-7.05450902897543	6.13988086458817
H	-3.39761975739424	-6.74674878432543	5.81050963975380
H	-4.45601706001910	-7.26125462525109	4.56145234153483
N	-1.90105879018978	-6.74578047044141	2.23464469565587
H	-2.33118922499869	-6.00684132394265	2.80985571050197
H	-1.65493448753077	-7.51685155998646	2.87367561035268
H	-1.09162500596895	-6.29698543551774	1.78452103726397
H	-2.61075431329298	-7.02151575977035	1.54094708946377
N	0.12259630445690	-3.83523423987711	4.28692474418357
H	-0.79361822756980	-3.51364976667628	4.63728855207605
H	0.05746606835325	-3.93102129180994	3.26060936741650
H	0.27782791022130	-4.77968254422194	4.67145559046380
H	0.87558162853545	-3.14780104207359	4.56046517895679
N	-4.74105950806951	-3.72210297495204	1.35961422174500
H	-4.51680602478950	-2.89182376222783	1.94880068799053
H	-5.43546342118987	-3.40957350521362	0.64985345967782
H	-5.13718871012672	-4.48833558226367	1.92314398996411
H	-3.87943023610922	-4.05444950361401	0.89909901148013
N	-1.33236559724789	-1.02122349165324	3.30336626093245
H	-0.40095128271990	-0.75563659767008	3.68749212381594
H	-1.22326551137500	-1.62104339181997	2.47020842926322
H	-1.93501684625095	-0.22310847382077	3.02651646436827
H	-1.87083431829762	-1.56624463316285	3.98915998100003
N	-3.24179874699235	-3.79333992823835	4.64523975413530
O	-2.48174342515399	-2.94140623124257	5.13015120909209
O	-4.35923211491498	-3.98060967589240	5.13043318866257
O	-2.85994776265934	-4.46889360338372	3.66495499825490
N	-4.21334263682747	-0.33696715325236	2.09938865766378
O	-5.12120982924790	-0.20252039744186	1.27591646615506
O	-4.09486179117546	-1.40719847919894	2.74280447443296
O	-3.40452671760940	0.58089078685412	2.30482017568952
N	-1.12103503533239	-7.00736244856861	5.18143745098833
O	-1.67430224267032	-6.37123255123873	6.09763185320408
O	-0.03565172698458	-6.63073979645156	4.71967249733465
O	-1.68588400945743	-8.00901212276491	4.70319225135397
N	-1.33942993185121	-3.93390639792703	0.98393668510477
O	-0.35408071542052	-4.48557375924685	1.52686110761177
O	-2.17132909947997	-4.63797511514038	0.39093856587734
O	-1.48606080409816	-2.71244043014112	1.04698014121798
N	-4.93514257316629	-6.92868046111607	2.25676853885364
O	-4.50733155186049	-6.80704350680393	1.10218465169860
O	-4.50317963183295	-7.85856100393738	2.97749420432583
O	-5.74952716329483	-6.11852404250676	2.72206261945141
N	2.12091586923423	-0.89782244414568	4.82293015044975
O	1.15059539149112	-0.34239909672590	4.25679855662767
O	3.09853723516751	-0.25076864773725	5.15132351195086
O	2.07400529081197	-2.13361961542861	5.04813144277138
N	-6.59043345857591	-2.25154924117658	-0.30284430229278
H	-6.45257054570703	-2.09366831534166	-1.29602690205648
H	-6.21219256647850	-1.44235932323126	0.19892401483247
H	-7.58998105145626	-2.28895592283661	-0.13065107956740

p=6 [(NH4NO3)p (NH3)(NO3)]- ωB97X-D3/def2-SVPD

N	2.26361184795267	-1.72461224442108	0.41436469530264
H	3.07238557356200	-1.09356093009931	0.50085555938584
H	2.64884406346494	-2.67775458461346	0.33027540067232
H	1.77537444362345	-1.45562017764034	-0.45353830106749
H	1.62076576901077	-1.52825563738128	1.20399725113625
N	2.73770493724138	-0.90615378787592	-3.79562212035512
H	3.10628758481374	-0.53042885469695	-4.69160522865674
H	3.20074218258108	-1.77816475523299	-3.52003508000250
H	1.70515271903214	-1.01551213274749	-3.78632380819993
H	2.97088281906962	-0.24012835170610	-3.04365498157912
N	4.32719192773573	3.51508022103864	-2.86821311623688
H	3.54736116018312	4.14301614414430	-2.59872669694177
H	4.07817365640521	2.56690279156591	-2.54017210286608
H	5.17764736031750	3.76760286050340	-2.36219099974998
H	4.48322837578095	3.44587523049444	-3.88941775743614
N	0.28949273016049	2.38275805749714	-0.10318996588701
H	0.14667139369470	2.13461105737166	0.89649947141045
H	0.25666464173700	3.39911897139305	-0.21817504620166
H	-0.33903961914421	1.85805016652535	-0.73662646523685
H	1.25638397377460	2.10750371637107	-0.32688947769605
N	4.71112004546434	3.50617968383861	1.53788403439345
H	4.36289466705011	3.51404025581169	2.52529647321633
H	4.21392845140424	2.73286467596333	1.07522973332858
H	5.72717778361980	3.34816522993622	1.41352967685093
H	4.42035107033818	4.35066138412825	1.01605224583946
N	6.23571435493862	-0.43818597673733	-2.12254681008277
H	5.92030797839806	-0.08490351034154	-3.03534230057622
H	5.46440053958054	-0.29370441132192	-1.45231177393142
H	7.03066508354885	0.13171044907155	-1.77213909253214
H	6.41161238557701	-1.45321071867851	-2.13656421879110
N	2.50894396937104	4.85839145948987	-0.52684104978947
O	2.01907900974787	4.58747965680826	-1.64192673581007
O	3.70632199765147	5.20377922066337	-0.45694510786563
O	1.82322525654073	4.76405207457230	0.49409897490850
N	-0.01650902707326	-0.08085717898328	-2.30111445508423
O	-0.03055869652268	-0.77313833143717	-3.32961817952363
O	0.95406967027122	-0.16435824880779	-1.51365927025274
O	-0.93761354044007	0.70290463469123	-2.05357138785126
N	3.66232844531632	0.99747200496463	-0.64207437108222
O	3.03906160781921	1.85254812734090	-0.00271421136406
O	3.62008913164309	0.99707282149628	-1.88190029179050
O	4.33917446387513	0.14717376146620	-0.04847624458494
N	7.13069312224135	2.17183967468342	-0.30391517855113
O	7.34830521271836	2.76627935072506	0.76062462270096
O	7.92882566586300	1.32005766511498	-0.72217302232057
O	6.09575212924832	2.42358853054924	-0.95645110771042
N	4.48441504982209	1.40425275068317	-5.28458856100585
O	5.24644818288260	1.03557221015096	-4.36985883934706
O	3.81502577601103	0.56659336824401	-5.91149236788198
O	4.38378235697899	2.61144172694871	-5.54515297928028
N	4.66894741419887	-3.13693751849409	-1.18585317266736
O	3.92803014837355	-2.40891201242124	-1.88843390019664
O	4.18203049027716	-3.74155520987179	-0.22214952886517
O	5.87339246222497	-3.22004840669793	-1.45211899094761
N	1.07920902669715	0.58529796228815	2.54809801717832
O	2.12568089378030	0.30671578264535	3.14021255514134
O	0.62387289126855	1.74664105821654	2.58449742889088
O	0.47217839271355	-0.28446007744900	1.89277870837248
N	3.18522266483704	3.14806987704821	3.90740681313786
H	3.43708359303999	3.15320787343922	4.89090811535338
H	2.81660009425297	2.21576237985898	3.69359878122599
H	2.39343118585189	3.77632084701237	3.79259942465410

p=7 [(NH4NO3)p (NH3)(NO3)]- ωB97X-D3/def2-SVPD

N	0.42956473545567	-2.50602762231807	1.45830481153622
H	0.10963119591874	-3.19821586214448	2.13080179328667
H	0.79775815860795	-1.67085961969632	1.96850191721311
H	-0.37712017199782	-2.20912647416945	0.86854455052231
H	1.12825516027528	-2.93618186975258	0.81812845965580
N	-4.38597879568927	6.11129686783955	-1.64670064849616
H	-3.37780607054131	5.96232789198913	-1.51574415856094
H	-4.58990371033177	7.03666266896145	-2.07545715500836
H	-4.84731823974238	5.98389844842405	-0.72405240562097
H	-4.73365664201053	5.38318740123318	-2.29973882208270
N	-4.45940979718189	1.87711012663604	-1.99572887928537
H	-4.70813431329586	2.60430243133226	-2.68762523559379
H	-4.83470172942476	2.17486003515006	-1.09008350359264
H	-3.43859206836149	1.83104831967476	-1.88056052447877
H	-4.81400526907988	0.94362326787378	-2.30373638861927
N	-2.79085643906850	2.44856291445214	-4.81837805875267
H	-3.01401154370951	1.48670029421101	-4.51668856320689
H	-2.55047687065460	3.03335443332819	-3.99347664882265
H	-3.61020213745354	2.88042512866852	-5.28098103016882
H	-1.94641296407566	2.41237353403713	-5.41103761416450
N	0.39853126876869	0.74009655811752	-1.47767393854194
H	0.71898826208950	-0.23922517070016	-1.52087740669020
H	0.64314326706933	1.10637804551096	-0.55349051658497
H	-0.62554319168054	0.76863946621664	-1.55131341391577
H	0.80690264673897	1.26952107058205	-2.27727531945810
N	-2.25184861490872	2.22385690932807	1.41644128495423
H	-2.99995158212076	2.84855830074843	1.77265506629750
H	-2.59025623396144	1.25115609783640	1.37019261664881
H	-1.41133134573409	2.23110235133324	2.01788308152081
H	-1.99506345232209	2.51300862031517	0.45672369305236
N	-1.11428783990693	-1.81571633299015	-3.16450982507668
H	-1.29181181873162	-1.70317232561029	-2.14972074582568
H	-0.87180187468327	-0.90431889698248	-3.57973976977194
H	-2.00220338390533	-2.14349361320867	-3.58083775579117
H	-0.33159849622337	-2.48480965540499	-3.25763027031386
N	-2.23493373321168	-0.78069631814044	-0.01063013782728
O	-2.64705770481318	-0.65237665370343	1.13888419577346
O	-1.57173079705407	-1.79719691015848	-0.33264321696155
O	-2.44295994227420	0.08721399826002	-0.87093536899336
N	-5.36992717924181	4.59340527495586	-4.67754177379998
O	-5.21928101645048	4.13608682018266	-3.51113845371780
O	-5.06306110790164	3.88981810285773	-5.64685360298569
O	-5.81371563388673	5.72828337319939	-4.82829270993678
N	0.41192267467506	0.62531635305857	2.43875017193920
O	0.99429304681957	-0.29639270853562	3.03466550919637
O	0.18970605218775	0.50934800688211	1.21380253538093
O	0.04007416388707	1.63428123985744	3.04130011233455
N	-1.92522131593187	3.99795005798486	-1.62364454339791
O	-1.71751531734100	2.79119905526562	-1.35398844074728
O	-2.26655124173585	4.30803011693673	-2.77783108729026
O	-1.82369015325952	4.85777090438179	-0.75286391952721
N	-4.72867358194781	4.39685148104585	1.05883649479363
O	-4.34710990177336	3.97384161978390	2.15580971525215
O	-5.24445055571307	5.51887824360879	0.95679037730649
O	-4.57690826483345	3.69631386216201	0.03463163221381
N	0.22227427918581	1.72233915915851	-4.53222627118402
O	-0.03144325585014	2.41133996720219	-5.52365704800619
O	1.22310810111540	1.95033330519528	-3.83458528547439
O	-0.55918967599682	0.79812657508820	-4.21106946220631
N	1.37404303675218	-3.12114048448738	-1.53630405703920
O	1.38122764483626	-1.89222286925257	-1.31479022636689
O	1.04627484421309	-3.55501551925841	-2.64601951113838
O	1.68407083985758	-3.89576039761060	-0.61691741525515
N	-4.15731343183477	-1.01429442329179	-3.48364629880152
O	-5.18770920987386	-0.62629157867258	-2.91047217694782
O	-3.89662370605299	-2.21770122740828	-3.57262831024421
O	-3.35903095298059	-0.17969129055681	-3.96041434306631
N	-5.23680185310770	8.29237368626865	-3.34457580374263
H	-5.57072312748146	7.54833578319054	-3.96141610990850
H	-4.57955426161586	8.85607470798483	-3.87496828611607
H	-6.02917985949261	8.88303994574249	-3.11181155977065

p=8 [(NH4NO3)p (NH3)(NO3)]- ωB97X-D3/def2-SVPD

N	6.12609435134135	-4.21166908980658	-2.34042861825446
H	6.95744694165128	-4.23253218407987	-2.92427627816492
H	5.42122521827179	-4.88605256251184	-2.70479871588041
H	5.72741644936310	-3.24892219155830	-2.34161325988407
H	6.37929918010661	-4.50521125224996	-1.36597033183977
N	2.40883641606316	0.85365373116716	4.70000311402634
H	2.43150027779185	1.63768236996750	4.03015339969533
H	2.18578718641101	0.00300683687025	4.15963610567200
H	1.66991145676704	0.98704615597173	5.41402747939275
H	3.36251271325557	0.73826034200236	5.09647154780333
N	2.21873358111340	-2.16289050508074	-1.24896104599439
H	1.20051855528910	-2.00659183443030	-1.14090193593227
H	2.68764906505943	-1.34942735018312	-1.67963789837935

H	2.42170287244183	-3.02598405454388	-1.77678503619059
H	2.62498918182947	-2.25566437227790	-0.30454518918561
N	-0.61952219510228	-2.69567655587015	3.85068273768254
H	-1.00629063331595	-2.25247472396498	4.69958748172303
H	0.33224592831681	-2.33517359336125	3.71130257474111
H	-1.15051638182433	-2.40951948243386	3.01322989002603
H	-0.54306477556545	-3.72811304286342	3.90867697688495
N	0.07623270131763	1.47986087297582	1.99434075427820
H	0.51918120065707	2.41436281019046	1.91513294202511
H	-0.25964076724846	1.29395849752078	2.95041386075453
H	0.80223387740512	0.77113173077503	1.80031267193491
H	-0.69946778488395	1.36683134103774	1.29206388871923
N	4.27965699035972	-3.80720783208570	3.64204908637146
H	3.89918619657457	-3.21926995729596	2.88617857714939
H	4.91012373034950	-3.21443725046862	4.21021106722022
H	4.78895705891071	-4.58539520816516	3.18868394970591
H	3.47982486516511	-4.15500089996206	4.19805877260748
N	4.77629784516086	0.75977424623991	1.11545422679135
H	4.70151514329832	1.78768865672011	1.04616058207590
H	5.43130704037200	0.36626796552119	0.41742881936388
H	3.84536988179832	0.38772058929381	0.89323770826431
H	4.99595346217281	0.46412377545781	2.07977260563526
N	2.12306619487731	-5.14581091447492	0.63119538540087
H	2.01258813357156	-5.70099392987335	-0.23833555839084
H	1.47082094790483	-4.35172523015054	0.63958739064450
H	3.10691104311820	-4.81083199415792	0.65731003421703
H	1.97046478867418	-5.70775321731451	1.48963420737076
N	-0.61291637102682	-2.15307329753106	0.55547796501001
O	-1.52968244018091	-1.76278309695128	1.28685650408311
O	-0.50114508300144	-1.70085559484112	-0.59997537475635
O	0.20793116151010	-2.98529275302865	0.97859657491050
N	1.32486672919407	-5.29718841354559	3.84744745442177
O	1.70976306527565	-4.49862992422537	4.71789240279441
O	0.12069564120589	-5.37196568930871	3.55636267755997
O	2.15713479229036	-6.00242573996649	3.24716573395225
N	5.19532121311435	-0.78945912053517	4.68058260946874
O	5.67816821065063	-1.83047930574501	5.13518197048786
O	4.84788322987428	-0.74270010755517	3.47793678635618
O	5.02830276390866	0.20172978774881	5.40725590370714
N	5.01622110963334	-0.79670468818690	-1.71884509277883
O	4.02500967595185	-0.06055927529989	-1.55539509711016
O	6.03711307733327	-0.62882887995367	-1.03979043970554
O	4.95343912203467	-1.71287227966708	-2.55811987132791
N	2.79580315068929	3.22714996576161	1.60350392642022
O	3.72849949409347	3.44768022574588	0.82815989909825
O	1.68003241334190	3.73945006293868	1.43641041155281
O	2.97960900722907	2.44440059219043	2.56572421319270
N	3.24993927891239	-5.75461175684086	-2.38102073726768
O	4.25554453575311	-6.24753130451808	-2.91052263888065
O	3.09862660721736	-4.51619892940861	-2.41433152370325
O	2.41463533535573	-6.47140466698867	-1.81521060981423
N	-0.54170864804848	-0.01269724947095	5.59297119906179
O	-0.53492121992933	0.15366196117679	4.34990637012328
O	0.10650895494391	0.75569874120262	6.31736260604198
O	-1.17090059097186	-0.96146234921941	6.07022119777165
N	5.81271390665310	-5.05235886472801	0.86282451347178
O	4.81843307895613	-4.43883151821889	0.40999585625032
O	6.82269365034793	-5.16126663969520	0.15098080355270
O	5.78944700750427	-5.53344400415435	1.99679633587818
N	2.36203900823853	-1.45008555189171	2.01551586363971
O	3.05472244663313	-2.33598666784312	1.49693273080038
O	1.98392161317044	-1.55978642415888	3.18714068784593
O	2.06582762548862	-0.44572279745907	1.34386146143005
N	-1.91527328814618	0.95783223987910	0.01250779335769
H	-2.15883115842073	1.63385457133694	-0.70434613354692
H	-2.77456884833171	0.62986359914311	0.44476905346553
H	-1.50618219726867	0.13769845126663	-0.44071295689669
