# Supplementary material for: Decomposition and Growth Pathways for Ammonium Nitrate Clusters and Nanoparticles
Source: J Phys Chem A. 2024 Oct 14;128(42):9184–94. doi: 10.1021/acs.jpca.4c04630 (PMC11514028; doi:10.1021/acs.jpca.4c04630)
Supplement: Supplementary file 2 — jp4c04630_si_002.zip [file jp4c04630_si_002.zip › SI_ammoniumnitrate particle structures_PDF_XYZ/HassanAmatTopper_SuppMats_S06.pdf]

Supporting Information for:

Decomposition and Growth Pathways for Ammonium Nitrate Clusters and Nanoparticles

Ubaidullah S. Hassan, Miguel A. Amat, and Robert Q. Topper*

Author Affiliations:

Ubaidullah S. Hassan, Department of Chemistry, The Cooper Union for the Advancement of Science and Art, New York NY 10003, United States.

Miguel A. Amat, Department of Chemistry, The Cooper Union for the Advancement of Science and Art, New York NY 10003, United States.

Robert Q. Topper, Department of Chemistry, The Cooper Union for the Advancement of Science and Art, New York NY 10003, United States. Email: topper@cooper.edu; Phone: 212-353-4370.

Table S6: Cartesian Coordinates of p=(1-9) $[(NH_4NO_3)_p\,(NO_3)]^-$: ωB97X-D3/def2-SVPD

p=1 [(NH4NO3)p (NO3)]- ωB97X-D3/def2-SVPD

N	-0.12819597948685	1.16262186011079	-1.98414096465207
H	0.03028240222809	1.32846888569586	-2.97904011955150
H	-0.29879920128960	0.14533837497470	-1.76401938658920
H	-1.00673540704177	1.59664077567647	-1.69651744741854
H	0.71160034137547	1.62283265017191	-1.54134939926875
N	-2.31605485146375	-0.96929935282788	-1.09218984817770
O	-1.12055943625954	-1.26771338833442	-1.37393119771708
O	-3.10326258698380	-1.84761872250284	-0.76826169781034
O	-2.66865428682002	0.22565284302084	-1.15247148838689
N	2.39456539206106	3.03159113125595	-2.50744164197275
O	2.08067423245830	2.57753919848937	-1.37025890007156
O	3.38076294692474	3.74525621609364	-2.62711335994947
O	1.68371043429771	2.73879952817555	-3.48971654843404

p=2 [(NH4NO3)p (NO3)]- ωB97X-D3/def2-SVPD

N	2.07740144294619	0.22016890340747	-2.06475855920044
H	1.59502752557630	0.07972653926409	-1.13910807999229
H	2.14285009118775	-0.68615596321516	-2.59583819249614
H	3.02343679667361	0.54174511488976	-1.87752838485471
H	1.58949416305959	0.95903485905273	-2.63164207841029
N	-0.83841483504240	-0.75611253776366	-2.69697721042294
H	-0.82214558739910	0.28177260768654	-2.87529988947467
H	-0.18015925569030	-1.25909818274907	-3.34586354055913
H	-1.78004811874089	-1.08882591681534	-2.88702442745908
H	-0.61833596894189	-0.97302577733189	-1.69171078414323
N	1.81621273360941	-2.56143449222786	-4.31694909959439
O	2.27223213916556	-3.41327675532704	-5.05658700708118
O	2.47476519358789	-2.15107584679372	-3.32945140582012
O	0.67765720570894	-2.07479253944450	-4.52592722080743
N	0.16375227208989	-0.86294785715620	0.77294418082110
O	-0.46568936215460	-1.49553943304501	-0.11026903744551
O	1.00333552543502	-0.00065254365075	0.41440616266408
O	-0.03487233282575	-1.08150125175240	1.95328663472821
N	-0.13919451449581	2.64693964018615	-3.58541473658163
O	-1.04982159459816	1.91450124946234	-3.12628228189893
O	-0.39324270088152	3.75662979216387	-4.01535657699146
O	1.03829018173025	2.21140539115963	-3.59353446497974

p=3 [(NH4NO3)p (NO3)]- ωB97X-D3/def2-SVPD

N	-1.47285302318091	1.84737605638097	-1.19336089753563
H	-2.19384981571147	1.15859784718871	-0.90552743943398
H	-1.70971301308878	2.77225981495338	-0.79621545552314
H	-1.50750800083076	1.94256393298074	-2.21264157864632
H	-0.50529048032630	1.56842560863662	-0.94957132091455
N	0.30257632034561	4.00384873158460	-4.25572044902925
H	0.57379306251501	3.05311502502722	-3.88731111684828
H	1.10662861208333	4.45473466434171	-4.68000838368508
H	-0.03701329064455	4.55577251851152	-3.43849683932985
H	-0.48906001424147	3.88722312099717	-4.92584781829482
N	-4.78944915553285	4.13708142813220	-2.39487183563417
H	-4.65041046968810	3.38836705809827	-1.66032519186274
H	-4.65024811252232	3.73704708366708	-3.34573834838476
H	-4.06509506196586	4.87234221597044	-2.27260819206119
H	-5.72078212723308	4.53234189176397	-2.31242381293638
N	1.59340001075736	1.93306060053538	-2.10209051990665
O	0.77232991822570	1.66641296671522	-3.02723993626391
O	1.34839763835493	1.51773716366112	-0.95888915696531
O	2.59013878736121	2.60189095223838	-2.34996969555891
N	-4.57922899058579	1.07055266599680	-1.05424980727063
O	-5.63679633100366	0.89251748529183	-1.64451717504400
O	-4.29757492839329	2.21099931753904	-0.58889189175174
O	-3.74709382969403	0.15889369609764	-0.90584276264935
N	-2.81639301255916	3.39560058932597	-4.82322829391170
O	-4.03460248755323	3.52454678209643	-5.01072065370277
O	-2.01293881776984	3.64954651701886	-5.73734307754351
O	-2.39719644257160	3.02479373416513	-3.71254773214970
N	-1.79136274627847	5.15235372196061	-1.66479830701283
O	-2.50746966138688	5.83887256780782	-2.41362494441376
O	-2.30336525173044	4.54808251829900	-0.71018385970932
O	-0.56957828514994	5.05627472301563	-1.87526050602541

p=4 [(NH4NO3)p (NO3)]- ωB97X-D3/def2-SVPD

N	3.54567928252762	-2.09977808638305	-2.90423341981186
H	3.10558242585040	-1.82870672047575	-3.79212588051771
H	4.53163943834023	-1.77386392901343	-2.88491234576372
H	3.45138143228495	-3.12254099496975	-2.77714734630228
H	3.01034136220942	-1.61274461849188	-2.17413470299151
N	3.10351962003562	2.26663670656730	-3.23190443333646
H	2.67697947207703	2.16043379816258	-2.27979639214604
H	4.13578074445164	2.09441986010328	-3.16760036615644
H	2.91631527852531	3.19274956842658	-3.60497985162452
H	2.67789744336751	1.55726388382900	-3.85434968326668
N	-0.62888439719172	-1.59212903646718	-1.71716779784000
H	-0.26093462001316	-1.01818154053306	-0.93893004202841
H	0.00399022966854	-2.39494646000146	-1.84523267866423
H	-0.59203768950082	-1.01275204409492	-2.57334505449785
H	-1.60325652855329	-1.94072709575816	-1.55011919864860
N	-0.88427472170376	-3.39758275839759	-4.39842633816559
H	-1.13836474666759	-3.66633046680944	-5.34557980235367
H	-1.76286714657477	-3.07972250809672	-3.90452898434826
H	-0.17026837791267	-2.63405750914536	-4.45012644573961
H	-0.43621587481087	-4.21348876523626	-3.92596197393323
N	-3.83122260816893	-2.71010465973168	-2.37281347245619
O	-3.25944629814573	-2.58416188777393	-3.49133220894704
O	-5.03229452119207	-2.88642500754244	-2.31508361358177
O	-3.13487710518095	-2.64285095632685	-1.33533474395758
N	1.61711319061305	-4.64286409908397	-2.78536735387191
O	0.78397152419798	-5.34343712675368	-3.38526657822627
O	1.23264365897379	-3.69822698484362	-2.06764717984696
O	2.82805360827024	-4.85795449672344	-2.90861177532542
N	5.50723852204424	0.29529838027011	-3.24780076555544
O	5.69378465250727	1.45048450768944	-2.80789211534017
O	4.82873370721508	0.13255833638583	-4.27038251040131
O	5.96545242206760	-0.68652043171811	-2.63897974303431
N	0.88390396912499	-0.34830689505376	-4.52481537958987
O	-0.22684512446940	-0.02282386839224	-4.08880439628443
O	1.68316812355936	0.48888367062529	-4.93896070400416
O	1.19735995260605	-1.56776131474585	-4.54915179600998
N	1.51761157006911	0.67615210460963	-0.84345515545786
O	0.70971975879984	0.23659591675291	-0.02124062399599
O	1.96196075130775	-0.06334491401448	-1.74779529675557
O	1.88531461939106	1.86200644315621	-0.79423384922088

p=5 [(NH4NO3)p (NO3)]- ωB97X-D3/def2-SVPD

N	-2.20673012280578	3.49354786011974	-4.49566438713811
H	-2.65538363377430	4.28280281196465	-4.95215756634387
H	-2.81663166747261	3.16246399107821	-3.71472152699794
H	-1.27928813139605	3.79860019402304	-4.12207132351978
H	-2.07471285787538	2.72745719009373	-5.19391017992540
N	1.68253151916970	0.29618078835451	-3.31427106685710
H	2.26262037842369	-0.40602083357508	-3.80915465791073
H	0.76849084408013	0.34112614441672	-3.78882509925402

```
H   2.06867707240643     1.25883328885234    -3.30857604488828
H   1.59902439268551    -0.01861115211531    -2.33646237930065
N  -0.61556662311120     0.61986758657055    -0.50534119413813
H  -0.28577553350675     1.16995642470244    -1.31150364215550
H  -0.69026400785017    -0.38460910628199    -0.72403884581100
H  -1.56443724143502     0.96646352719178    -0.27099376363765
H   0.08731288167073     0.71465446388017     0.25083710898489
N  -1.48492163161458    -1.50333141790462    -3.33526162658850
H  -1.76675699482755    -0.59528879757949    -2.93750257012670
H  -1.99220049706893    -2.24702305695908    -2.82091105037850
H  -0.47860647988049    -1.68811325518068    -3.21316993397218
H  -1.67849984008895    -1.46157958892378    -4.35271971417581
N   1.95007624076708    -3.86384676274814    -0.25004377181572
H   0.91291965172515    -3.97115468055784    -0.18816115273839
H   2.24843635508951    -2.98417667218661     0.22822321563168
H   2.24673949127052    -3.84252040941219    -1.25243717789989
H   2.39130147173972    -4.65602620737333     0.20872833672717
N  -1.27423354653765    -3.32586334808252    -0.76211165795725
O  -0.72765199801750    -2.22201008083824    -0.57529772305249
O  -2.27583890765078    -3.41791761007973    -1.48375954027930
O  -0.78763408549025    -4.33666742839142    -0.23031846969445
N   2.06941814097693    -0.66285134409276     0.55518034059004
O   2.61401247281856    -1.58285920100118     1.18708522824406
O   1.59392481616314     0.31160032333947     1.15218313986639
O   1.99043532518339    -0.74577459594215    -0.68530794295774
N   2.31393632532465    -2.79472888468712    -3.36294649501630
O   1.12826399895879    -2.59008697696049    -3.04105088050347
O   2.92833488810675    -3.73772439746023    -2.83752824009578
O   2.87997349741396    -2.05783419808718    -4.18073682832390
N  -3.21888329256112     1.79818425691302    -1.83906664323817
O  -3.78471802133593     2.79306706881520    -2.32019828672910
O  -2.65267299145320     0.99445089602616    -2.60457574215087
O  -3.20123593486774     1.61946733690299    -0.61421196645166
N  -1.48561015745834     0.51664848618732    -5.75718356519627
O  -2.08287245748557     1.42945309903635    -6.35031360828505
O  -1.78164914272632    -0.66649497730709    -5.96830215049555
O  -0.59869805060878     0.80992561626886    -4.93259561036800
N   0.80082755163656     3.32287827566609    -3.12928732462064
O   0.32552699714095     4.30705301667269    -3.71934369361422
O   2.00420020506183     3.05175449763093    -3.23492584707519
O   0.04741633108726     2.60383583902128    -2.44647547836473
```

p=6 [(NH4NO3)p (NO3)]- ωB97X-D3/def2-SVPD

```
N   0.60528871966268    -1.07718424959105    -0.08706433871795
H   1.28999911099528    -1.84702154805458    -0.09959300520301
H   0.77102424719600    -0.50846754936813     0.76769797463606
H  -0.35517865298656    -1.47871138801913    -0.03537028613685
H   0.73789064599835    -0.55618429910736    -0.95083030955349
N   0.50866524409319    -4.85588051329380    -2.32978407418328
H  -0.28308639179318    -4.23483141059249    -2.07182626734793
H   0.75325449798083    -5.48636004028868    -1.54870309316348
H   1.38415009038793    -4.30230289174180    -2.51295423517891
H   0.29411853403935    -5.42036599905661    -3.14627451979110
N   2.98231276360699    -3.57177711701413     4.22923576838248
H   2.57521070413382    -4.28966509824478     4.84323849096852
H   2.61114069763898    -2.62429554915466     4.42076056851701
H   2.68911400746152    -3.84395623156718     3.27384078092840
H   4.01710204887755    -3.62310083931210     4.26653275883731
N   2.88572602506398    -7.14586148155158     1.95125403839779
H   2.62276368421008    -6.86959992639214     0.97526527765328
H   3.35291053748665    -8.04739620681850     1.94009013421148
H   2.02461297870055    -7.18049489802319     2.53939752549404
H   3.52895677356705    -6.43346216706003     2.32538146935054
N  -1.10769940673007    -4.09960202578059     2.50548473633599
H  -0.55081598053689    -4.42963780717903     1.69825986091222
H  -2.07784991922513    -4.06786384173915     2.19088970849665
H  -0.76280821329696    -3.14960918060163     2.69785968568947
H  -0.92421000603776    -4.69966269405056     3.33299312177785
N   4.93024100462680    -4.33918794920515    -0.38925054351948
H   4.10451572571347    -4.02004267206310     0.14188832041591
H   5.74113453312350    -4.31182988094391     0.25238330062392
H   5.00410824022335    -3.68810306536455    -1.18700534666489
H   4.74591875661838    -5.30361438479898    -0.70721057722060
N  -1.97146434120299    -3.21201416122145    -0.45112847168835
O  -1.51534869013414    -3.04691774860186    -1.59882924888717
O  -2.47273025348916    -4.28468705472782    -0.11382588098190
O  -1.89278225125878    -2.27210082242645     0.37109810918288
N   1.28267597351342    -0.94454382938553     3.13091952642059
O   1.94970214661712    -0.91112173520023     4.17134318727513
O   1.14684512636723     0.06998079727572     2.43261971218183
O   0.75489945370363    -2.01893060066905     2.76876240160651
N   5.76477955137654    -4.38762890129911     2.70331860174908
O   5.77351661138073    -3.86306654556833     3.82500109396359
O   4.83967577935976    -5.16757175483978     2.38937642180909
O   6.64922075505670    -4.13332485109293     1.87563146368753
N   0.83504849261550    -6.16683868921130     4.32516101613035
O   0.51368491125080    -6.98948359392373     3.44852689130622
O   0.00807683769201    -5.32987770091339     4.72477193945375
O   1.98800945274268    -6.16988820267374     4.78202981996501
N   1.65086058497813    -3.97982293739726     0.94214514922883
O   1.82339096015483    -4.67386471048774     1.94984374592963
O   0.58514979204478    -4.05506010435915     0.32012719231127
O   2.53059166836549    -3.18893884137284     0.56131070357427
N   3.11159236923740    -2.56684525088069    -2.33543145368195
O   2.12080172895320    -1.83741444568601    -2.42678234913837
O   4.21974741225423    -2.11863710755628    -2.01866225773138
O   2.99774260745629    -3.79858856264938    -2.55767997095727
N   2.89447360945781    -6.86798089425671    -1.47939925696188
O   2.50343910528095    -7.10163945217036    -2.61039721762233
O   4.08545444372601    -6.99729183910822    -1.14879525837958
O   2.06844916169901    -6.47760455361525    -0.59912053469392
```

p=7 [(NH4NO3)p (NO3)]- ωB97X-D3/def2-SVPD

```
N  -1.28237141001520     0.32836496905996    -3.66505428862370
H  -0.65968199825753    -0.06098699611770    -4.41032375846678
H  -0.92673917432723     1.27225752045724    -3.37779507130740
H  -1.21087375764824    -0.31812401064307    -2.85803184610670
H  -2.24574497537664     0.39844525307236    -3.97803201376554
N  -0.64795966163907     2.40621958880276     1.15795096437810
H   0.23151051869711     2.82563485922910     1.49412991763826
H  -1.24435112896573     2.19893875415102     1.97756849225214
H  -0.40709133236265     1.53110213105935     0.66493788801784
H  -1.10158218866573     3.01370900174145     0.46251117367709
N   2.45833351866625     1.57520917793120    -1.61261753184631
H   2.24865385863930     1.00247525177937    -0.78888627254537
H   3.48246546786968     1.74812315736576    -1.60912415060099
H   1.89975998018776     2.44582048215094    -1.55851786037599
H   2.19889623373209     1.04192844400607    -2.45009426855957
N   2.12836712310420     1.52551890667217     5.69483072777933
H   1.10996051290934     1.29961367183104     5.65527419487677
H   2.69245510843582     0.80464756653180     6.19335298698654
H   2.24797686006202     2.43166095173763     6.14019431955469
H   2.50864132441180     1.57677142694392     4.72438495715892
N   4.88801384144398    -0.72936463635601     2.25605868669372
H   5.61171319660660    -0.37440861780074     1.60367945284859
H   5.26019042820062    -1.02462517371491     3.17484029746929
H   4.39525405299805    -1.51352461175151     1.80118942821251
H   4.22648059800374     0.04513741655320     2.43823728724236
N   1.58081874280179    -1.30138550447718     2.56288656622054
H   2.31432650267708    -1.15452649837379     3.27156048411123
H   1.57461584381406    -2.31134325863323     2.29387699092720
H   1.76115517101687    -0.73101491129431     1.72642571667903
H   0.70080073225725    -0.97042061798332     2.96890974256768
N   3.11696528151656    -2.27309692071266    -1.87088756520975
H   3.88392168835343    -1.65207865198529    -1.58183559825666
H   2.27182887093689    -1.90327120657235    -1.39778806883835
H   3.27020400135151    -3.22292648585958    -1.50112560100299
H   2.96860858421965    -2.24281982198859    -2.89472950328073
N   0.07032730620823    -0.73961713560917    -0.92155944410068
O   1.13420041392572    -1.00827045436357    -0.32890592613850
O  -0.46810350529652     0.36772989528517    -0.74730135697486
O  -0.43543156821159    -1.55240295134811    -1.69776045796207
N   2.73068782582755    -3.76372800780164     0.81537342843141
O   2.79307014205111    -4.53600821571195    -0.14361511578418
O   3.53892316092035    -2.81069733530238     0.88814925679411
O   1.86235714801116    -3.90240473603007     1.69239753006725
N   4.26891596432556    -0.79464051996836     5.46034734461265
O   3.23490925921382    -1.09587378700846     4.82834887187811
O   5.38113192996152    -1.10652815576585     5.02386681457551
O   4.15813098985502    -0.16358719044084     6.52530552016561
N  -0.83510389160910     1.34804156614822     4.23016854184209
O  -1.80772279202439     1.90239425893583     3.70893270054436
O  -0.03457767809104     0.70325250965405     3.52111137973411
O  -0.63052724961912     1.44182482565156     5.45103836801075
N   5.38590153233782     0.71748380107936    -0.54810327597061
O   5.25331204345001     1.70308660644069    -1.28669094143542
O   6.32663893010031     0.64620263383290     0.25249805469371
O   4.54311992731982    -0.20604013170700    -0.59079588190105
N   1.47526955951398    -1.09110479493241    -4.47331304386652
O   2.31680536267816    -1.98596642678240    -4.59250862573881
O   1.54867821147338    -0.28650531403936    -3.52220423237381
O   0.53857359847939    -0.98806360449374    -5.28283245467483
N  -0.37697056096446     3.19836373102469    -1.89072014770368
O   0.56209177430080     3.61475208109685    -1.18863791994944
O  -1.53656418188378     3.24407149412413    -1.46913684964646
O  -0.12664674863188     2.71615622334334    -3.01452426712689
N   2.40267065583147     1.92415232939723     2.32865396043909
O   1.80003435183714     2.96168265131145     2.59886985300306
O   2.20403241706780     1.31776789277253     1.27153210020411
O   3.22432825598585     1.44776965439517     3.15339033984678
```

p=8 [(NH4NO3)p (NO3)]- ωB97X-D3/def2-SVPD

```
N   2.13895329385683     5.47333465894713    -1.84084935732320
H   2.21986184365983     5.71949646906690    -2.84627294489874
H   1.14822408195920     5.22334736716084    -1.65775372946360
H   2.36500154688580     6.27170540812524    -1.21757110408277
H   2.75330418058255     4.67570430764813    -1.63487295276072
N  -0.09533499191853    -1.61905347598729    -3.62136522955308
H   0.25257656311309    -0.65896776008409    -3.44836102573972
H   0.00091160993571    -2.17337162811843    -2.74993338877582
H   0.52887761111506    -2.03582880849104    -4.33485837272480
H  -1.07897760045051    -1.53356277152989    -3.88897271546950
N   3.47985134244584     1.70382249212898    -4.35668222321783
H   3.79734420277305     0.86357306505189    -4.87321411671500
H   2.45595987176314     1.64827483105127    -4.28305461634609
H   3.72882250018189     2.60755872180169    -4.79145421716973
H   3.86867780805380     1.66416100722613    -3.39716336990054
N  -0.50330084941443     3.77306213440274    -4.23885718214897
H  -0.93942136950921     4.63456466405746    -3.87127211105401
H   0.08977215225663     3.41229009790598    -3.48020888718539
H   0.11878674762274     3.96261908173623    -5.03893082711104
H  -1.20580336041520     3.04070828421454    -4.45513660356341
N   1.35409575464869     3.26678760156662     0.71897010361917
H   1.05908195666394     3.11483482770614    -0.25660924467265
H   1.38901986876179     4.27688369724231     0.92411556784082
H   2.28672735263209     2.83476894646385     0.80460563085545
H   0.69486351713904     2.77385440660497     1.35315727750903
N   2.77674071573439    -1.01162099204936    -0.59867770412754
H   2.66928082671789    -1.02802632049753    -1.62340489532528
H   1.85954357638586    -1.22505183867289    -0.14675912778093
H   3.11804314163298    -0.07754488946773    -0.29921594389116
H   3.45398177522813    -1.72435881087302    -0.34209215104718
N  -1.93321492238398     0.50568066741835    -0.07847606137663
H  -2.50330379979088     0.95758242507013     0.64848961149988
H  -2.03345569370448     0.99554231688713    -0.99720309855126
H  -2.11974520591189    -0.50200300758368    -0.18878422698684
H  -0.96476642137331     0.58507552510579     0.24253401939808
N  -1.65724657946283     6.14616557040337     1.78875206593016
H  -2.39807693329859     6.59917245528273     2.31675487549642
H  -1.45095692145905     5.22195980810290     2.21745516103328
H  -0.81174875261421     6.76070481956144     1.77334584734926
H  -1.97858451902057     6.00845168891967     0.79779281119197
N   1.33976725079587     7.16255143516929     0.87008089212448
O   2.09876469331543     7.58676361793645    -0.00844734354252
O   0.44826958977689     7.88937325051919     1.33796089683172
O   1.45624762813406     5.99675328245340     1.29578501265966
N  -2.21470592612231     1.07861926308434    -3.42603300504410
O  -2.14179722626865     1.48726977940926    -4.59096097171615
O  -2.44593151943926    -0.11034883036975    -3.17796531562280
O  -2.03650951699869     1.88404870326140    -2.48111688782859
N  -1.52075544105445     5.64524623697869    -1.53384027590497
O  -2.46705337322387     5.98874613962747    -0.80955319326135
O  -0.54379846827463     5.05269920913268    -1.02021794273043
O  -1.54198545024428     5.89205150740153    -2.74073858149832
N  -1.45786888010566     2.62253260892307     2.34193614304772
```

```
O   -1.14246789083317      3.68001581622278      2.91561707763321
O   -0.55737226386805      1.80269727534875      2.05682787001028
O   -2.62969403591183      2.38291651670612      2.04458810918870
N   -0.24963746337396     -2.21828956182366     -0.39529461638364
O   -1.47270117586847     -2.23779283026811     -0.55719592150814
O   0.49757228438300     -2.83161789687070     -1.18520397819820
O   0.25978959809907     -1.56276990231939      0.53415380845789
N   3.97030904908971      2.10881495720691     -0.81812237608771
O   3.96934884816532      3.32983423392555     -0.95961326013810
O   3.57977915336143      1.60230878786958      0.26248411175521
O   4.34084237837682      1.34709021458184     -1.72348246735739
N   0.89206541834299      1.49497751067848     -2.29930334275745
O   0.72665472992182      0.73895854897791     -1.34580922631096
O   1.21179212216273      2.68867796891589     -2.11755185854789
O   0.74884515580042      1.09278355722555     -3.46766419501308
N   2.91507935037881     -1.41665069421198     -4.68617069617182
O   2.15183290435334     -2.28228447193199     -5.13774028530075
O   2.81533814487576     -1.05521113031189     -3.49398390959928
O   3.76325883788637     -0.88476472036567     -5.41551135441295
N   2.45575251297366      4.65815988778528     -5.06660731717524
O   1.84740816761005      5.63377592144989     -4.58818528359787
O   3.64463264609742      4.46054274278798     -4.77417932794775
O   1.85434324666378      3.86375002138738     -5.81032253081087
```

p=9 [(NH4NO3)p (NO3)]- ωB97X-D3/def2-SVPD

```
N   0.64340492307995      3.68543970996748      2.38053611047823
H   0.53213117520102      2.72382605467293      2.01771149547218
H   -0.22466709932326      4.01416857268675      2.83867529412165
H   0.79172607960715      4.34847660196521      1.60119297873314
H   1.36379978097641      3.72551738835765      3.11493049652952
N   -3.59105464008429     -0.14749075592217      2.33460526562016
H   -3.26312991684566      0.81125228877537      2.53750225534381
H   -4.31778592780777     -0.04727062733686      1.60664389580916
H   -3.91610861788664     -0.53764325994808      3.22509621757020
H   -2.76537072826290     -0.66190587393787      1.98988387159324
N   -0.52493169275114      5.75047442901128      6.39851845069454
H   -0.69580106174716      5.60140320302552      5.38535996027281
H   -1.38634046302306      5.46706794765172      6.89425062818888
H   -0.37837049896075      6.75791454557315      6.55122632405753
H   0.25339395356466      5.13932503443130      6.67502107159035
N   -4.04924801056128      7.56066876364124      6.52851548929947
H   -3.17535775487313      8.09682202622060      6.67949476971774
H   -3.78025386102937      6.73055169773722      5.99047814267687
H   -4.70923386091544      8.10438303712920      5.94974187414072
H   -4.42258821178201      7.20710867998509      7.41949319401536
N   -2.51836294003041      3.57296955810257      0.16449083497773
H   -1.97633018284539      2.69714100937584      0.13593346396206
H   -3.52533998334553      3.38767725042652     -0.00177398544258
H   -2.15266014084206      4.28374178160367     -0.48197744767945
H   -2.44180155778148      3.98646181798110      1.10637489949629
N   -2.79740625522124      8.41556002675240      2.15425752682957
H   -2.98474496565577      9.21637273219166      1.55753685543466
H   -2.00396497995858      7.87168581534205      1.74875471329171
H   -3.66424307238367      7.84778740397293      2.21496073077848
H   -2.55553547475221      8.75725750371965      3.11289369940558
N   -1.94771718502131      2.31170871278004      7.29809726486271
H   -2.05082624122982      1.28727840984378      7.18786260683497
H   -2.43413684627432      2.76368712493159      6.50821679115730
H   -0.94654015504665      2.55110796654045      7.21416931926572
H   -2.35655595022876      2.70226583680363      8.16260482363801
N   -5.26601392245159      4.30095306439908      3.26023007904284
H   -5.82884041115421      5.01902101444643      3.75606980503390
H   -4.58065106607739      3.86576405094948      3.89677920219831
H   -4.73470207767768      4.81209884576393      2.54734086085023
H   -5.80903564653961      3.55169769655232      2.80146252644024
N   0.26572186397097     -0.12259265840952      4.22142308145442
H   -0.39077323150313     -0.90615713066482      4.38974690123861
H   0.98253848425299     -0.02556943294440      4.93991875346040
H   -0.28892072565629      0.75316755051858      4.25915773675758
H   0.65742051005448     -0.16741254366120      3.27446589198064
N   -0.61293829559500      0.42091284134520      1.17342476317395
O   -1.36021044441529      0.97185792361380      0.36397186356510
O   -0.90392366632134     -0.69459654863744      1.63825526256749
O   0.44516341727862      0.97060708215368      1.55105840205470
N   -0.79118794677249      6.18501799575049      0.30546113044410
O   -0.05200657568800      5.18643162941123      0.16690323760268
O   -0.52883928029167      7.03442575590508      1.16989694727833
O   -1.79408804609942      6.30027823234216     -0.41112182130837
N   1.36332250269190      3.03550307160486      5.78485250586235
O   0.77515386529826      3.24401191536061      6.87219362967954
O   1.68883703872096      1.89317029933984      5.46156139052676
O   1.61345451252209      4.00006094174092      5.04554837793168
N   -3.55040595316530      4.94774120326812      8.48972903278385
O   -3.04115656429983      5.01257133293094      7.33838662640053
O   -3.28793914817991      3.97855538916860      9.21095776915239
O   -4.30372752595412      5.84531770957133      8.87000253547386
N   -2.29358500329305      5.61756860866851      3.44621554578592
O   -2.90096677700721      6.35090649029262      4.22382451949713
O   -1.20440463225883      5.11811926588272      3.78359038079903
O   -2.75602004477887      5.37253714461069      2.32227262103850
N   -1.33178806076666      8.67238224671317      5.25279556355386
O   -0.40884805601670      8.25259169991049      4.56748214377760
O   -2.27477170162589      9.31248527553876      4.75096291813276
O   -1.36300980380714      8.43455750830720      6.49019966207092
N   -5.40082020178644      2.05660752917230      0.87950651437869
O   -4.92869885724729      1.09284371709082      0.25936048263897
O   -5.98783121435198      1.87770440210877      1.96040967364261
O   -5.26089108749347      3.21294326009009      0.43661739635441
N   -2.28946157453187      2.57752102535268      4.06078823739761
O   -1.20817804120968      2.20857273827939      4.53908728243406
O   -3.11660746025282      3.14187324632183      4.78605181449361
O   -2.53116940589629      2.39591935007077      2.85477728886368
N   -2.51767126437749     -0.61644858806406      5.44878130485346
O   -1.81207854112674     -0.37981850033792      6.44505087500763
O   -3.58094293073223     -0.01408363199374      5.26742518665120
O   -2.12417300934175     -1.45229388547544      4.60630735385221
N   -5.60927600707921      7.47559691053157      3.73638246610219
O   -6.14491215360631      6.62701207053679      4.47928649264786
O   -5.37665043270303      7.19909999115433      2.54707436428170
O   -5.28980155897147      8.57856164521358      4.19658901468482
```
