# Supplementary material for: Decomposition and Growth Pathways for Ammonium Nitrate Clusters and Nanoparticles
Source: J Phys Chem A. 2024 Oct 14;128(42):9184–94. doi: 10.1021/acs.jpca.4c04630 (PMC11514028; doi:10.1021/acs.jpca.4c04630)
Supplement: Supplementary file 2 — jp4c04630_si_002.zip [file jp4c04630_si_002.zip › SI_ammoniumnitrate particle structures_PDF_XYZ/HassanAmatTopper_SuppMats_S12.pdf]

Supporting Information for:

Decomposition and Growth Pathways for Ammonium Nitrate Clusters and Nanoparticles

Ubaidullah S. Hassan, Miguel A. Amat, and Robert Q. Topper*

Author Affiliations:

Ubaidullah S. Hassan, Department of Chemistry, The Cooper Union for the Advancement of Science and Art, New York NY 10003, United States.

Miguel A. Amat, Department of Chemistry, The Cooper Union for the Advancement of Science and Art, New York NY 10003, United States.

Robert Q. Topper, Department of Chemistry, The Cooper Union for the Advancement of Science and Art, New York NY 10003, United States. Email: topper@cooper.edu; Phone: 212-353-4370.

Table S12: Cartesian Coordinates of p=(0-7) $[(NH_4NO_3)_p (NH_4) (HNO_3)]^+$: ωB97X-D3/def2-SVPD

```
p=0 [(NH4NO3)p (NH4)(HNO3)]+   ωB97X-D3/def2-SVPD
 N   1.82703288094127    -1.21455109690397    -2.72666465286370
 H   2.41340294077737    -2.05296696806810    -2.68685403400687
 H   1.89248775159682    -0.70937020882878    -1.83803996879162
 H   0.82398905816957    -1.48475015419729    -2.89563478958272
 H   2.15555056663758    -0.60890746782689    -3.48376754602945
 N   -1.56789452326994    -1.26050595133861    -2.38090760966942
 O   -2.86100714675506    -1.53804289586554    -2.53518300510018
 O   -1.24095552116270    -0.44976616965082    -1.56079231226863
 O   -0.83375017599226    -1.88425881092005    -3.11740508936957
 H   -3.34244560594265    -0.98099163439993    -1.89584609331774

p=1 [(NH4NO3)p (NH4)(HNO3)]+   ωB97X-D3/def2-SVPD
 N   1.48932592271906    -0.05619255719562    -0.16107536337291
 O   0.25782958298807    -0.27753619345162    -0.33666856997060
 O   2.02131632331489    -0.36368352015869     0.91143175688994
 O   2.13490743567639     0.47087559363686    -1.05944789289091
 N   0.58695928470931    -1.12132367098931     3.02974513053801
 H   1.17304789871957    -0.97787872870109     3.85449944019330
 H   -0.22899478772987    -0.49899402828871     3.07063747149064
 H   0.27515797104507    -2.09330932602312     2.99973011478706
 H   1.14050476778356    -0.88224616774933     2.14545234209602
 N   0.12209986388692     1.79504052373760    -2.34330345972470
 H   -0.40608157639879     1.20259773071232    -2.98501574660817
 H   -0.45124128332718     1.97947007080006    -1.50405113138937
 H   0.37251535830307     2.67121575723532    -2.80319771745875
 H   0.98027933513158     1.27606871825994    -2.01788821394061
 N   -1.50937952080926     1.95570744210592     1.27685162097903
 O   -1.16047459178828     0.67512954504047     1.53965706906035
 O   -1.22967945960216     2.37546977401328     0.16961252689476
 O   -2.05017554819504     2.53377585393363     2.15586020953333
 H   -0.65516597642692     0.31720318308209     0.73420541289356

p=2 [(NH4NO3)p (NH4)(HNO3)]+   ωB97X-D3/def2-SVPD
 N   -1.07171915769080    -0.11363955016283     0.87591698200641
 O   -1.64019156164965     0.98690437847191     0.81420731945650
 O   0.16459381158304    -0.16716549006391     0.98793071506090
 O   -1.73269929066551    -1.16231618249275     0.79266758362256
 N   -0.13372724840661    -0.39669190319889    -2.13762295727682
 O   -0.11759272621575    -1.64330404073281    -2.30782930861820
 O   0.93729301538423     0.19064291336800    -1.97128616275460
 O   -1.20515505247514     0.21481278415755    -2.11321907318205
 N   -3.78262668045162    -0.38700576019718    -0.82745938762507
 H   -4.36136756191047    -1.18032828465726    -1.11350717901556
 H   -3.18239359126627    -0.67722994125381    -0.01690704378923
 H   -4.35049244389863     0.42126854901970    -0.57936442604461
 H   -3.11709476098383    -0.15704842615122    -1.57974808498277
 N   0.93743867807416    -2.65007797704909     0.07206066690470
 H   0.92265924797519    -2.43391847065364    -0.94027584132304
 H   1.83541600409655    -3.03415921209263     0.36188190985521
 H   0.17634901840244    -3.31669510066760     0.24205898587705
 H   0.71868137298288    -1.75593170469115     0.57483468080714
 N   0.36134185583383     2.41213442149184    -0.46321197197836
 H   0.64063955289450     1.64917814917906    -1.13111225446731
 H   0.06549356593518     3.23959686951298    -0.97941185109248
 H   1.14737180432809     2.64182741045683     0.14446595981927
 H   -0.42495352411843     2.03436405178461     0.11715828157255
 N   -2.53636043048547    -3.38130646668600    -1.00707782047127
 O   -2.50998071431855    -2.55335585048886    -2.06309154431069
 O   -1.51157602986692    -3.95469886486924    -0.72315110806743
 O   -3.59491004857001    -3.46051341258553    -0.45553849252390
 H   -1.54419210451639    -2.31234788874805    -2.25230357745889

p=3 [(NH4NO3)p (NH4)(HNO3)]+   ωB97X-D3/def2-SVPD
 N   -1.51073054014961     0.23052211114878    -3.79307616191775
 O   -1.56995043727556    -1.00708919728115    -3.63103293166254
 O   -0.90666243756895     0.68866483183432    -4.75649501446939
 O   -2.04851290935954     0.98647348839115    -2.95798137650906
 N   1.26520893026254    -0.52375462566359    -2.02974486861296
 O   1.08828172571772    -0.18861908783892    -0.85312684087868
 O   1.43587879549318     0.35957966169167    -2.89695677988748
 O   1.25559929272264    -1.71457950130622    -2.36717316745016
 N   -1.90015712186817     1.60751233439317     0.32671457608080
 O   -2.49273421891831     0.55040080598223     0.61581555581953
 O   -2.50327356082052     2.50453120635426    -0.28906102446364
 O   -0.71054770726859     1.75205286888233     0.62630787067768
 N   0.91590655158840    -1.45888699895061    -5.08690009267925
 H   0.26670691844954    -0.64445146540058    -5.12728856147030
 H   0.48273205728584    -2.26230126619387    -5.54003973848507
 H   1.09322229606486    -1.66084152006426    -4.06699151858300
 H   1.79653485051793    -1.22590126137899    -5.54376633618597
 N   0.12373607165372     2.57196030237108    -1.90373493910490
 H   -0.78048245978023     2.37066593400631    -2.35677101642053
 H   -0.00564320564484     2.44100906254609    -0.87951427164534
 H   0.42517770566330     3.52081979639271    -2.11463077567269
 H   0.80428786243330     1.85881444089489    -2.25408927121147
 N   -4.47596737878536     1.19970523151673    -1.58014724510996
 H   -3.84953218304904     0.95284101659692    -2.36218039349718
 H   -3.88398241937557     1.80284353438998    -0.95009881944182
 H   -4.70980167215503     0.33049189073851    -1.09107201983344
 H   -5.31264000587162     1.68149679575211    -1.90320447160920
 N   -0.40663976073901    -1.21518385247871     1.20861536777365
 H   -1.20053011303594    -0.53835054012281     1.13127492633700
 H   -0.78773136553021    -2.16088578017522     1.14505593573142
 H   0.08376997667969    -1.07823389828290     2.09023065187696
 H   0.23478327656196    -1.02596323950404     0.40713213047060
 N   -2.98356287773801    -1.96921281377724    -0.70609626643283
 O   -1.76702792380661    -1.93499685494271    -1.25104710438948
 O   -3.90642900421204    -1.55519875347109    -1.37033901018060
 O   -3.03202357280066    -2.41122760290969     0.40576496214439
 H   -1.83265943534110    -1.53238205414064    -2.18071795910716

p=4 [(NH4NO3)p (NH4)(HNO3)]+   ωB97X-D3/def2-SVPD
 N   -0.37277159209271    -3.15807453771598     3.52733191886594
 O   0.40322971764204    -2.20852247052846     3.37076470136538
 O   -0.02411127066771    -4.30384280034144     3.19686532003651
 O   -1.51373471814139    -2.98248234008649     3.99060421560060
 N   -2.58224801257385     0.79553808092460     3.82862485624179
 O   -2.44547753041417     1.13711595666164     2.63893057999238
 O   -3.69159163755973     0.41503835822187     4.23343035450816
 O   -1.60641369871408     0.79703027716576     4.59422724808529
 N   -1.04681166087417    -2.25213746336191     0.59521517392718
 O   -1.24228127221836    -3.47407471513779     0.61806834656615
 O   0.09499437711444    -1.82746651627289     0.28095391771144
 O   -1.92350149075484    -1.44345274613470     0.88889001792049
 N   -4.74412316965292    -3.49083955266716     4.14644345433212
 O   -4.52472252869826    -4.56787805220010     4.72210139064669
 O   -4.76905886582335    -2.42239820651978     4.74601417604459
 O   -4.92208541100757    -3.51803822129702     2.90150135911208
 N   -2.75448551524518    -5.19556662124121     2.36630507749708
 H   -2.29700399712549    -5.26077339018004     3.28806597585987
 H   -3.69445603583654    -4.76763550235798     2.48986919786238
```

```
 H   -2.83987774783054     -6.12682360640546      1.96361355833430
 H   -2.19693171975797     -4.57883354754321      1.75021258128856
 N   1.70744529295275     -3.86919955082635     1.14941914267813
 H   1.18360332561516     -3.08108000350119     0.68977702832639
 H   1.78444485412084     -4.65306513094162     0.50322451008609
 H   1.14357430017760     -4.14364591871823     1.99733424244378
 H   2.63518435049833     -3.56308668890136     1.43837403000064
 N   -4.56453817811432     -0.92743550454957      1.98808972717876
 H   -4.40088442776892     -0.33074992422015      2.83098426168776
 H   -5.24853530924105     -0.48991346758334      1.37451717025671
 H   -4.87815947848666     -1.87016243833093      2.30311914188970
 H   -3.66846125574178     -1.04006227179401      1.49446808041303
 N   0.09182977212212      0.33879203486079     2.15326284237003
 H   0.81822523740049      1.04061659656446     2.27536166203430
 H   -0.86047048494814      0.77580055161182      2.25401301266162
 H   0.17249764210122     -0.39360871615788     2.87625348350246
 H   0.16723469950765     -0.14212690048568     1.24324295853190
 N   -2.40374981108192     -1.40092230231170      6.11044391529674
 H   -1.89364129381664     -2.00625168143658      5.44197465890481
 H   -2.17880637247040     -1.66761886151327      7.06718167632722
 H   -3.40332205935235     -1.54750265315876      5.91778660738242
 H   -2.15058699370567     -0.42090319869409      5.88427296765455
 N   -1.63258403674330     -4.95335050795077      6.01179357182906
 O   -2.68904787666838     -4.28777259199817      6.51271360129312
 O   -1.85792630059640     -5.80139090289372      5.17881160232331
 O   -0.57143392355044     -4.62568022895706      6.44939848051441
 H   -3.47931889197722     -4.50049312109460      5.92463220261372
```

p=5 [(NH4NO3)p (NH4)(HNO3)]+   ωB97X-D3/def2-SVPD

```
 N   -2.16309752440102     -0.61706007469586      3.25571608148528
 O   -1.50071242021596     -1.40006955774307      2.58311043297342
 O   -2.11287038307365      0.60830714735545      3.05032399782263
 O   -2.91934051910429     -1.04110364407483      4.15681230776254
 N   -3.29388256498940      3.31593666084331      2.71029609993092
 O   -2.31186653305665      3.55920958092515      2.02197237321093
 O   -4.36730480127233      2.95850936335187      2.20707338370721
 O   -3.23387385776774      3.40549368753881      3.96887140136608
 N   -0.72215634410977      0.06038321300761     -0.21372373207665
 O   -1.16047421481621      1.21960067653723     -0.24992528232384
 O   0.43683666414149     -0.11785426029704     0.23423716968637
 O   -1.39548047139107     -0.89497582971390     -0.58763463394901
 N   -1.93398178289514     -4.51899825522800      0.72184843247945
 O   -1.50007596701980     -4.62502087240296      1.88166595139640
 O   -1.16053072797106     -4.21273824208602     -0.20442625709532
 O   -3.14021176858886     -4.68040483173620      0.49225110842252
 N   -4.90122651734607     -1.11498863588027      0.89905887606119
 O   -4.89776191940228     -2.33527177267726      1.10183981092621
 O   -4.92522320881905     -0.71473089083723     -0.29529980241082
 O   -4.86337133333585     -0.29998587722612      1.81507597634456
 N   -3.44138525209247     -2.76973209449993     -1.48384575707835
 H   -2.50826203834515     -2.34867674891750     -1.41962291181002
 H   -4.13721490324407     -2.05583093033303     -1.18100979040778
 H   -3.46671553365784     -3.57870488414134     -0.82057734510221
 H   -3.61834838777034     -3.08718835889234     -2.43453696717972
 N   -4.69347945110444      1.08593130207043      4.47968485555551
 H   -5.35011188018025      1.04343473394110      5.25588830683524
 H   -4.00793260770188      0.30096399106603      4.51958032931557
 H   -4.17775790405688      1.99146582702914      4.48643847095396
 H   -5.18433212609275      1.01933865394703      3.58332813345713
 N   -3.86194948861163      1.85058664531399     -0.28084321116198
 H   -4.06701942893205      2.33079081416361      0.62242529815190
 H   -4.09275593698210      2.46353201949175     -1.05939232298236
 H   -4.40602362507743      0.96464049458087     -0.32957901638419
 H   -2.85179870375091      1.60094875582688     -0.31410778925366
 N   0.63849817875437     -2.69591021217054     1.16933955990377
 H   0.14078905943364     -2.65269957476834     2.06242995036407
 H   0.08951392224865     -3.34566325591567     0.55604899673159
 H   1.58457945052656     -3.04521579409881     1.30411316507473
 H   0.63441275450083     -1.73706896726125     0.74876898699173
 N   0.13206663756283      1.94014476582810     2.17594390853393
 H   -0.26831191518452      2.83822777155320      1.89464271484235
 H   0.74331111369884      2.06344765249866     2.98397312087388
 H   0.59471487363261      1.47202354154629     1.38383885775804
 H   -0.67743931282867      1.34315718135498      2.45718333305425
 N   -3.66687894081904     -3.58766870872664      3.27147691388830
 H   -4.23446372089001     -4.20776670129924      3.84409793371814
 H   -4.22941095729925     -3.20087849354879      2.48648923405277
 H   -2.85690227413011     -4.10505562900985      2.86488993363490
 H   -3.32296100680451     -2.77359568182034      3.81411732243995
 N   -0.89250773642656      1.75739303097552      5.32483891625729
 O   -0.81687014132383      2.93655429039565      4.68530909333255
 O   -1.90349011607963      1.50989811180633      5.92618712972455
 O   0.08381262558393      1.06361035953951     5.21204589180638
 H   -1.76691203112073      3.24849450751393      4.52111405838735
```

p=6 [(NH4NO3)p (NH4)(HNO3)]+   ωB97X-D3/def2-SVPD

```
 N   -6.52651754699157      2.58173501816428      1.08288735105368
 O   -6.34585595054786      1.36086491864990      1.17317545229243
 O   -6.78632315377701      3.05206762135708     -0.05270634968014
 O   -6.45018383332370      3.32831990531772      2.05312533104206
 N   -2.98867981716202      6.65920312548946      1.43006981554582
 O   -3.26103345296608      7.26768870138354      0.39186833328889
 O   -3.87225787535317      5.98617307596611      1.99279480401445
 O   -1.83909297625307      6.71427510584023      1.89551922666567
 N   -2.04157885673482      0.08387851245838      0.70878045031868
 O   -0.82740088770731      0.32675120824218      0.79615924566942
 O   -2.64457428226161      0.40251771948880     -0.34427855761075
 O   -2.63600047684680     -0.45153420448103      1.64018901840114
 N   -2.65517344269117      4.30632890141187     -2.45417824921010
 O   -3.41594064330947      5.22486566735779     -2.15429616831304
 O   -1.42247110083037      4.52938076122176     -2.53906159645186
 O   -3.06071032193283      3.16019780227199     -2.67831364646828
 N   -3.36111826907635      1.52538988840458      5.14806257480645
 O   -2.72788161213234      0.47313458002054      5.01201241691256
 O   -2.76266063088267      2.61158437863911      5.25629726225752
 O   -4.60717822575207      1.51179572791191      5.13164903782516
 N   -2.95123235627770      3.21711758291943      1.03454908646334
 O   -3.91984987951541      3.33762334854683      0.29117898786633
 O   -1.83193038406662      3.65079525099102      0.67922415629581
 O   -3.07078752414080      2.67118564931317      2.14403679286367
 N   -0.79027778062199      1.50724188737654      3.34258038937983
 H   -1.50596291673623      2.15657071219578      2.96571076674352
 H   -0.55044669382259      0.86055632654177      2.57459668717978
 H   -1.23956774479027      1.00131759888200      4.12830940106726
 H   0.02549161984368      2.04474605476542     3.63330372410697
 N   -4.61799085479212      4.24274153188503      4.05273254555464
 H   -5.26093823071150      4.68018229120594      4.70936633902098
 H   -5.15033008412063      3.61370491218154      3.43198166969552
 H   -3.90063689888561      3.68779340928755      4.57840002902576
 H   -4.16475752779662      4.95518185556567      3.46150551315855
 N   -5.07596331251496     -0.17278789309994      3.04385405374462
 H   -4.94151000132843      0.29957826952525      3.96647965969325
 H   -4.16433744647561     -0.28149110709835      2.56961925435324
 H   -5.66769786328749      0.43882095821130      2.44484739126813
 H   -5.50731518208770     -1.08542760443153      3.17041839270192
 N   -5.55743600291741      5.50382795064877     -0.29288387640646
 H   -4.82467680095745      5.31489426878971     -0.99923886559177
 H   -6.11359410257385      6.30690194836553     -0.57575629215745
 H   -5.07802348663800      5.71045550863411      0.59966349283685
 H   -6.14788247633935      4.65163997485533     -0.17233056966397
 N   -4.96071030215409      1.49997591593853     -1.53366869089526
 H   -4.36375369266941      1.00091087908939     -0.85404979429029
 H   -4.36921701335190      2.22849451077257     -1.98088050003308
 H   -5.75007186357071      1.96088258836431     -1.03817448830874
 H   -5.31109497943784      0.84720069877293     -2.23092566896635
 N   -0.62143083114555      2.13055685126429     -1.31403523218631
 H   -0.83516622314758      2.70917230484081     -0.48112392105573
 H   -1.33440166360399      1.37947178306633     -1.28989292623900
 H   0.30640774619457      1.72127067406993    -1.23282228678049
 H   -0.72818544900068      2.74229824754993     -2.13524609590388
 N   -0.87767100088116      6.69847408946987     -0.93622205844101
 H   -0.40566794259598      6.29366549082739     -0.11625695787087
 H   -1.07026779742018      5.93880006782644     -1.63270127678803
 H   -0.29737675745234      7.42785141327088     -1.34553826229646
 H   -1.77892745806121      7.09533217011928     -0.60624361801293
 N   0.46580972640830      4.44212928361465     2.01322316952880
 O   -0.42663628818699      4.79508135536204      2.92943636662477
 O   0.60131722612796      5.14094001308286     1.03379624870305
 O   1.08033563520468      3.43892032066215     2.25059146546654
 H   -1.00122978316713      5.52787724085943      2.54480304618449
```

p=7 [(NH4NO3)p (NH4)(HNO3)]+   ωB97X-D3/def2-SVPD

```
 N   -3.32388612825372     -5.74628139248227      4.34468166838684
 O   -4.04155897421672     -5.06850452400566      5.10561364136345
 O   -3.58840456311194     -5.77514924138847      3.13366610467556
 O   -2.36193969989919     -6.36533601562277      4.80943789524272
 N   -0.71121925979378     -2.51954673785586      3.22877701804598
 O   -1.18764123548363     -1.53900149125734      3.81960075494645
 O   0.31526499643911     -2.42113157050416     2.56051234554834
 O   -1.30045121169171     -3.62747614640729      3.31292350255409
 N   -2.56365248437114     -0.93829947740982      0.97014785203012
 O   -2.34398085636294     -2.09145988301622      0.58130786445194
 O   -1.82594004295223     -0.01159590912723      0.62108439408080
 O   -3.52106522700695     -0.69820727009590      1.73634000234035
 N   1.07431038739484     -4.22040938699416     5.42893431764566
 O   0.20454680331958     -4.76162969462750     6.11223050777857
 O   1.75698350719441     -4.89750455875639     4.64580422993295
 O   1.28587079411220     -2.99416512368417     5.50237739699469
 N   0.87738109922938     -4.67805580734038     0.65885405522494
 O   0.35350134055476     -3.86412494629962    -0.09114691915029
 O   0.23849015671983     -5.70004845588129     0.99536199750579
 O   2.03587204718357     -4.51388463571541     1.08055513557955
 N   1.63973057839942      0.22697925984302     2.78695887021532
 O   1.96490249027110      0.07908620120002     3.97270228862679
 O   2.38239325748888     -0.25849976089001     1.90324019497568
 O   0.61970657971246      0.83011914220693     2.46082923329817
 N   -2.69843087800648     -0.60775351328497      6.21092213162860
 O   -2.07220856990441      0.46163320155407      6.02098064898542
 O   -2.21191110367511     -1.44179816423057      6.98574122523799
 O   -3.76213154549892     -0.80395510223428      5.62924291234106
 N   0.55072822094874     -0.42943167478365     6.22747595307558
 H   0.99313715361616     -0.09631430197615     5.34326878775181
 H   0.52715240633265     -1.46010947935056     6.17478617231813
 H   -0.41513299684256     -0.05293179435308      6.30179300383117
 H   1.10716383175910     -0.14113768041594     7.02909127386576
 N   -2.34138253927822     -4.18448579839197      7.08722267478631
 H   -2.48717828387062     -3.15545965323903      7.09761719096146
 H   -2.44507691751990     -4.56828980893788      8.02417937526245
 H   -1.38060449463092     -4.36602877066441      6.74015158351694
 H   -3.02896956632673     -4.63051892609188      6.43992193867465
 N   0.66294219261015     -1.04599626329484    -0.12175737568599
 H   -0.27983099349890     -0.66090958619839      0.09579372575636
 H   1.34110836226952     -0.68073222373371     0.58144864414266
 H   0.94290658212285     -0.76120315082118    -1.05770796861796
 H   0.62781017362033     -2.07864774019873    -0.06689802312974
 N   0.10626624912956     -6.82751281746238     3.61183762827247
 H   0.76419240303669     -6.24137593565071     4.16825956868469
 H   0.39500615546584     -7.80257324941520     3.65955887077317
 H   0.17813833370045     -6.49183858369417     2.63808623604976
 H   -0.86093899959823     -6.70549842938614      3.97271446842502
 N   3.02184033007205     -2.69638945086422     3.15737336921444
 H   2.50881767457585     -3.27415611531764     2.47823899261612
 H   3.93421425043145     -3.12515490218567     3.31045259955502
 H   2.47797244337551     -2.70036721434108     4.03580583538295
 H   3.07897075397446     -1.73373297052512     2.78399486818836
 N   -2.42379341152126     -4.87822022399733      0.79896337053646
 H   -1.43806858025160     -5.20120201535247      0.72276342591734
 H   -2.80948143016538     -5.22616473398498      1.69649138991544
 H   -2.97083292037453     -5.23631543682578      0.01878621576888
 H   -2.43717220233902     -3.84489909171559      0.78629358110517
 N   -3.93697735147309     -2.71263984748895      3.60182181090059
 H   -4.60532398014913     -3.48239522416922      3.65813572681492
 H   -2.97294921684231     -3.10955828982020      3.57955680884217
 H   -4.00132824854913     -2.11521522187283      4.44178352027250
 H   -4.04643945523984     -2.10824398020861      2.77017241837838
 N   -2.06899919345252      1.17669529317849      3.33214298376574
 H   -1.09269745038304      0.93776841984997      3.07900560992995
 H   -2.71119944627580      0.58707405237510      2.77216454103059
 H   -2.24035126835569      2.15717481397322      3.12086402651459
 H   -2.21015767091049      0.98785726721991      4.34498161993395
 N   3.86237597081078     -6.30708405408008     3.28129163674784
 O   2.81791180679668     -6.62831067487809     2.48760012941487
 O   4.41423142591414     -5.25511433684799     3.04907856594979
 O   4.12672687429446     -7.10498760840286     4.12278734262948
 H   2.63303676520092     -5.83279358134968     1.91248761147163
```
