# Supplementary material for: Decomposition and Growth Pathways for Ammonium Nitrate Clusters and Nanoparticles
Source: J Phys Chem A. 2024 Oct 14;128(42):9184–94. doi: 10.1021/acs.jpca.4c04630 (PMC11514028; doi:10.1021/acs.jpca.4c04630)
Supplement: Supplementary file 2 — jp4c04630_si_002.zip [file jp4c04630_si_002.zip › SI_ammoniumnitrate particle structures_PDF_XYZ/HassanAmatTopper_SuppMats_S13.pdf]

Supporting Information for:

Decomposition and Growth Pathways for Ammonium Nitrate Clusters and Nanoparticles

Ubaidullah S. Hassan, Miguel A. Amat, and Robert Q. Topper*

Author Affiliations:

Ubaidullah S. Hassan, Department of Chemistry, The Cooper Union for the Advancement of Science and Art, New York NY 10003, United States.

Miguel A. Amat, Department of Chemistry, The Cooper Union for the Advancement of Science and Art, New York NY 10003, United States.

Robert Q. Topper, Department of Chemistry, The Cooper Union for the Advancement of Science and Art, New York NY 10003, United States. Email: topper@cooper.edu; Phone: 212-353-4370.

Table S13: Cartesian Coordinates of p=(0-7) $[(NH_4NO_3)_p(NH_3)(NH_4)]^+$: ωB97X-D3/def2-SVPD

p=0 [(NH4NO3)p (NH3)(NH4)]+ ωB97X-D3/def2-SVPD

N	-0.62661257755635	0.04389590436330	0.96111500043468
H	-1.46099768147513	0.00976442689079	1.54718442864043
H	0.14633968543437	-0.26757594349675	1.54960199578260
H	-0.75027628490264	-0.67678641560066	0.24970641953974
N	-0.21161823943472	2.48320816448076	-0.09675163906146
H	0.73492261009279	2.55580168011421	-0.47610446625288
H	-0.33045842478190	3.20718719834038	0.61512850383639
H	-0.38430082252903	1.45638874479957	0.34881211790481
H	-0.87660326484740	2.66184824010840	-0.85236136082432

p=1 [(NH4NO3)p (NH3)(NH4)]+ ωB97X-D3/def2-SVPD

N	1.87350243996092	1.62004464290414	-2.21381514947014
H	0.86538099075364	1.45612048234218	-2.21212851166009
H	2.28977823468324	1.05123214678387	-1.35374989335271
H	2.05856735367706	2.61852253504901	-2.11425255362266
H	2.27102043318746	1.28859005093233	-3.09298451838124
N	0.11961536861532	-2.49758551750359	2.53774965750790
H	-0.00468139708002	-2.19963318163927	3.50469179968676
H	0.35580594855071	-3.48919555400845	2.52354458814146
H	0.89151903857360	-1.94034493800425	2.08832932230704
H	-0.76998058353978	-2.30231098633112	1.95434859708844
N	1.82173004002970	-0.34813294459691	0.32188054404207
O	2.06052397916265	-1.03620851449056	1.31455459384994
O	2.76493753636261	0.28586329362930	-0.20926582771244
O	0.68425486214310	-0.26713847157528	-0.16027067139107
N	-1.89497673329913	-1.76101358030123	0.82745744481945
H	-2.29207188088196	-2.46141702148444	0.20553639671608
H	-1.29891594403841	-1.15080854691677	0.26594794369703
H	-2.66924968686070	-1.20139289478895	1.17688323773420

p=2 [(NH4NO3)p (NH3)(NH4)]+ ωB97X-D3/def2-SVPD

N	4.14040029727451	0.84709426832762	2.17061029944354
H	4.83095948089557	1.43542519265606	2.63490647533758
H	4.50910526812153	-0.09148262229301	2.02570076171592
H	3.87043956524030	1.28622450714653	1.25821631786626
H	3.25978236304376	0.79710592943447	2.74722618733637
N	0.31041525611227	3.59289367300475	3.08241032132720
H	1.25780066795882	3.68501919532675	2.64026758339965
H	-0.31024500883869	4.31013656889393	2.70964277841087
H	0.37963571616372	3.67153598390495	4.09591312423083
H	-0.03940624646077	2.63539160679157	2.82443150283814
N	0.32926768065295	0.74996805668376	-0.89022845740446
H	0.61827530414634	0.15678904329539	-0.09067745170563
H	0.41156267145744	1.73173179126325	-0.57145320904060
H	-0.63216723147163	0.53919247211766	-1.14821451634927
H	0.99963359400435	0.63640790143417	-1.70999015467191
N	0.96834254632746	0.56043254108399	2.32499337387708
O	1.65900648738204	0.92659728244825	3.29696534583612
O	-0.15437734357353	1.06335828384550	2.14684213701465
O	1.41865910193971	-0.26617462671161	1.52192766679772
N	2.26667749440713	2.91649154719169	0.63300293438853
O	1.14816601486059	3.24138400783056	0.21803007014508
O	2.96564759077427	2.10403309680806	-0.00264442334473
O	2.68727154536426	3.36979314591422	1.71409984940011
N	2.28002958346086	0.76942419825158	-2.89982883299008
H	2.02480521590313	1.17182445897568	-3.79871248251163
H	2.80607653568990	-0.07843833808371	-3.09687356260671
H	2.91690784916363	1.42309983445781	-2.44642763874069

p=3 [(NH4NO3)p (NH3)(NH4)]+ ωB97X-D3/def2-SVPD

N	0.27359757830201	2.99697391410850	-3.69692772076842
H	0.73235197921531	3.76660947722076	-4.17885495764428
H	0.56703628644389	2.97594303981653	-2.70493915744518
H	0.58025697742013	2.09387474774957	-4.10785662966905
H	-0.78173121010135	3.10402952973591	-3.68585907146987
N	3.04508074927602	0.28828420795373	-0.41339559101594
H	3.14409956478629	0.77996850205766	-1.32566047415112
H	2.19253999487344	0.65911983957940	0.05931444478022
H	3.87217350392887	0.42292458578759	0.16453161143585
H	2.88705543129199	-0.72125452207429	-0.59570731065841
N	-1.83442883836530	-0.81789528740792	0.67098036735339
H	-2.12792296549995	-0.60199062446097	1.62213594301714
H	-1.69921098946073	0.07425703427426	0.13391532061347
H	-2.52685453807078	-1.41107756657173	0.21622187273859
H	-0.90788028620553	-1.32380190254063	0.68926476856295
N	1.15461500625356	-2.59743809363157	-3.86971593666109
H	0.56807252321369	-2.52342191757752	-3.00151029175399
H	1.93313724554290	-1.89003387382423	-3.77553003209370
H	0.60802201359182	-2.38073965936170	-4.70164472127295
H	1.54556149130755	-3.53565324632957	-3.93615359758093
N	2.31978239427827	0.41888149134429	-3.57852511810211
O	2.64954765100375	1.43168746601811	-2.94454547972274
O	2.96462193478047	-0.63289286248007	-3.43762864075026
O	1.32787560769135	0.44371596266772	-4.33028920713392
N	0.81583494955936	-2.18874816289615	-0.65126587761629
O	1.97303953443010	-2.24112571394993	-1.08853848652283
O	0.60439740199543	-2.01754208301745	0.55354732309884
O	-0.14884263473260	-2.28998410358589	-1.44372015458020
N	-0.04943881147346	1.69669076868727	-0.41567279677947
O	0.47217202678903	0.92818965300258	0.41219639795547
O	0.60659924042813	2.60327119808840	-0.92312549393560
O	-1.24923287807306	1.51788260183978	-0.72639994366729
N	-2.45783499166380	3.15651524578627	-3.11628716889724
H	-2.85623307341530	4.07951231771087	-2.96356592814136
H	-2.32950100144321	2.71980240424476	-2.20155779211002
H	-3.15157386789831	2.61489963203562	-3.62471146941148

p=4 [(NH4NO3)p (NH3)(NH4)]+ ωB97X-D3/def2-SVPD

N	0.95994015814398	-0.05609287910117	5.65558322453260
H	0.54573445363650	-1.01522840109187	5.67363209530725
H	0.44958298153203	0.47749900097441	4.94480620422190
H	1.95869221646776	-0.12483169156453	5.35254889867411
H	0.87337967244054	0.38630164353312	6.56788254427171
N	0.51991464660900	2.08561199888981	-1.18531460069329
H	-0.08359596636567	2.08919468493246	-2.00375916035520
H	1.17782903638445	2.91860068765863	-1.16840144986358
H	1.06047164659869	1.19397336400105	-1.13762244103530
H	-0.03628480379875	2.13927916951930	-0.31381419710475
N	3.87388051930014	0.17237875360517	1.40213720568680
H	3.47026629265518	-0.07428732904953	0.48191148397132
H	3.22416880075696	0.85462664948041	1.82811998301320
H	4.80080712107581	0.58066345532823	1.30761148437976
H	3.92322741607610	-0.69025854534219	2.00588113326792
N	1.21631776694628	-3.75519382115596	2.79780143401745
H	1.40930012377508	-3.14568821250832	1.98605089844329
H	1.45194438441282	-4.72109465805665	2.58137960964141
H	0.20775095564896	-3.65017820267337	3.03224214084943
H	1.78231744844831	-3.41481969658334	3.60666852948059
N	-2.06903196683714	-0.22555331974279	1.83773800220336
H	-1.43601805871560	-0.84479699116707	1.31129572739470
H	-2.04786466323517	-0.50737713343415	2.84992466103412

```
 H   -3.01369731094029     -0.28994040401221      1.46629859800825
 H   -1.67788385055458      0.73049854309305      1.74603767836573
 N   0.64053463021883      1.59114988234843      2.01140877661562
 O   1.73040638249688      2.03697477842700      1.62546101386085
 O   0.58247455549835      0.71523107739659      2.87606345360574
 O   -0.41561111638043      2.02476640591329      1.49029243106146
 N   3.28899637413282     -1.58638538801086      4.06262081708409
 O   3.41026026587170     -0.39312750216580      4.39192902354716
 O   2.60597777782448     -2.35361996840331      4.75419945260521
 O   3.82130835375756     -2.00091835817868      3.01841350505332
 N   -1.10341266381687     -2.06495416648668      4.27720862679369
 O   -0.29577963102878     -2.46739011644232      5.12254291754084
 O   -1.66796405793191     -0.96390590078494      4.42705578063519
 O   -1.32607331252749     -2.73729181782345      3.25525830012995
 N   1.21691444385233     -0.97716949107853      0.04003354840907
 O   1.73762295506769     -1.83595662595934      0.75245211483121
 O   1.96268191608950     -0.23167907077046     -0.64634361120838
 O   -0.00533088179337     -0.81272679617746     -0.00636413923993
 N   2.21179953915923      4.25736893117292     -0.66058204929415
 H   1.82673938608509      5.19209771327986     -0.77293645612463
 H   3.16349901358541      4.28696190989113     -1.01702284607909
 H   2.26286260407747      4.06812552152012      0.33984741715977
```

p=5 [(NH4NO3)p (NH3)(NH4)]+   ωB97X-D3/def2-SVPD

```
 N   -0.46305491988628     -5.14784746094904     -0.60603269177297
 H   -0.04734087577343     -5.24847483274047     -1.53783099484567
 H   -0.87516675086016     -4.18912220351073     -0.55547316434231
 H   0.24286289720062     -5.29725957320154      0.11100592852640
 H   -1.21654652095323     -5.86794488949707     -0.53270640018492
 N   -5.01678457132452     -1.46277244529515     -4.17121625172786
 H   -5.85139090984893     -0.92166320788758     -3.96039550877259
 H   -5.23852300115354     -2.44424816613353     -4.44218302103444
 H   -4.40863434972988     -1.52719383581326     -3.33770799106624
 H   -4.46632771901488     -1.00518170691499     -4.93117161509581
 N   -5.00385226237220     -5.99156363632942     -2.30237707147100
 H   -4.34861077455840     -6.69902478522648     -2.68068785548035
 H   -4.44907398738441     -5.16422874585724     -2.04395197090653
 H   -5.43390078135696     -6.41557698290862     -1.43697495513009
 H   -5.66140531453695     -5.71722925894770     -3.03387223145773
 N   -1.24227879372526     -2.46861615739154     -6.99783062870786
 H   -1.26134399934694     -2.37827487735820     -8.01080536495716
 H   -1.06952833309254     -1.53526089279766     -6.55137472092634
 H   -0.52346826691913     -3.14467425207522     -6.67844721216726
 H   -2.14925071363367     -2.80835045395602     -6.65006687800900
 N   -1.15154794085701     -6.83371523962527     -5.02532925554569
 H   -2.11242335509288     -6.43241413451325     -5.03081241077712
 H   -0.49855364011039     -6.06762134587471     -5.28186220270483
 H   -1.09611581659949     -7.61307871097125     -5.67615390078840
 H   -0.94507839038162     -7.16151242425477     -4.05726911500494
 N   -0.56157152566499     -0.62141129608100     -2.57611344180539
 H   -0.21846038927842     -1.36073232656392     -3.22249707460836
 H   -1.34933452917073     -0.12427915748537     -3.05163074982126
 H   0.18749164943770      0.03331033070303     -2.36394694627280
 H   -0.91129299836107     -1.09489552062318     -1.72668632395294
 N   -3.99521771685054     -4.51977159770118     -4.92809222217021
 O   -5.14683590309842     -4.18079866536993     -4.58936846575640
 O   -3.70382441366311     -5.72239050883450     -4.92707316559718
 O   -3.16261059211311     -3.65849071261003     -5.23971818786033
 N   0.06442194101444     -3.83029173768697     -4.42531220747527
 O   -0.11413481694811     -4.47446810356220     -3.39476367174554
 O   0.15929361661675     -4.42623150622300     -5.52725612154623
 O   0.13928982717582     -2.59567115354273     -4.42071734684534
 N   -2.32734153944614     -0.06569360660416     -5.22524726175594
 O   -1.13078064178145     -0.13629747170907     -5.54786018424646
 O   -3.20620265216979     -0.46582182953472     -6.00899329307374
 O   -2.64591482451912      0.36601324790531     -4.10557375599143
 N   -2.64118315525927     -2.83978611556410     -1.51657162205288
 O   -1.59333396809365     -2.63567814178201     -0.85302896200045
 O   -3.19410713544293     -1.87776056236400     -2.05198514163660
 O   -3.07325863228736     -3.98926103249796     -1.61905696666086
 N   -2.10828847515032     -7.47051795250620     -2.00806768933851
 O   -2.45625308768675     -7.06842120951365     -0.87997114103173
 O   -2.95283598401471     -7.86758200703646     -2.81819108657444
 O   -0.90571963017042     -7.43648007620910     -2.32572297903759
 N   -5.53056181056206     -7.35844100134147      0.08719656450898
 H   -6.10769346025544     -8.19536589597106      0.09067041803524
 H   -5.72192210283201     -6.85987226087087      0.95210100396095
 H   -4.55588862571243     -7.65834831588825      0.11747139150391
```

p=6 [(NH4NO3)p (NH3)(NH4)]+   ωB97X-D3/def2-SVPD

```
 N   5.02198216596982     -0.44326656975595     -5.78323648347862
 H   4.51144497517146     -1.11478144050123     -6.40341840868949
 H   5.34022737379817     -0.98642839771196     -4.96125387303771
 H   4.37500775347441      0.29865952352604     -5.41918409595400
 H   5.81049373848000     -0.01921593062817     -6.26513447149103
 N   0.75450761530923     -4.88246877729036     -4.00224945770043
 H   1.35084044317300     -4.50312818151170     -4.76024641285259
 H   1.33724373844893     -5.41918466322006     -3.33001990015415
 H   0.04860305595637     -5.49365172214385     -4.40687332678447
 H   0.31125881694951     -4.08568047468251     -3.49386301785850
 N   6.87285479603337     -0.74250481731995     -1.58648376398439
 H   6.22407144852419      0.03387277904133     -1.42654154391395
 H   7.80260504577573     -0.39062077347499     -1.79887313856970
 H   6.47646903611059     -1.31502235531882     -2.36376705006136
 H   6.86951630211423     -1.33034255330931     -0.71610023597642
 N   5.32001148359823     -4.39858463910335     -5.37300106015981
 H   4.67388149962227     -5.12369319048226     -4.95789840769775
 H   4.81905318288736     -3.89092533161120     -6.13121775539220
 H   6.17122814425450     -4.83764427004669     -5.71423704945394
 H   5.53293447470963     -3.71887366266343     -4.62570351539267
 N   0.71587155388214     -0.29130466243330     -4.11324921008486
 H   0.01206491027243      0.32027741532263     -4.59530357148837
 H   1.30868613073943     -0.75521860026837     -4.81399065600079
 H   0.25676019542669     -1.03054575999594     -3.55299441232752
 H   1.30094895377923      0.28623153038194     -3.49362200943570
 N   2.75129478738875      0.15736188333664      0.08441949404670
 H   3.70664952023622     -0.01472562315079      0.47242407363945
 H   2.26504248789501     -0.72939109948207     -0.12128456895192
 H   2.82353603114314      0.65808625655961     -0.82477942663988
 H   2.19691993791248      0.69759762334740      0.74447066874414
 N   3.52990411962277     -4.33607403145053     -0.01959528080325
 H   3.97798695356326     -3.62987780766732      0.59685457978581
 H   2.64056808744617     -3.95573673960377     -0.39264470387628
 H   3.32626640624631     -5.18999470312839      0.49502617080674
 H   4.12301715292357     -4.57987924273555     -0.83463398077068
 N   0.65936127944565     -2.54187126810696     -1.67371691560943
 O   -0.06578086996214     -2.58587989778426     -2.70069436962758
 O   0.92255003016755     -1.44628230236989     -1.17790808293959
 O   1.08513616777518     -3.59664187099372     -1.20161473125266
 N   3.62707829282964     -5.73422534262975     -2.95022300052359
 O   4.63805907116294     -5.17066750534535     -2.49528985584337
 O   2.65916584514272     -5.97746216458157     -2.21936582070796
 O   3.57013454349130     -6.02935114333413     -4.15868224751910
 N   2.70812165343665     -2.73402651648525     -6.43718631472292
 O   3.86889097999312     -2.61584128084989     -6.92528774152442
 O   2.02931061464482     -1.72564946844515     -6.26437390809100
 O   2.30784959681199     -3.86163304147973     -6.15144787883373
 N   3.58120554018719      1.08276647186809     -3.30255298450421
 O   3.30556288859779      1.20706993926910     -4.51034906959752
 O   4.73204900505117      0.83423967248392     -2.94090968065205
 O   2.65531007637265      1.19188037997809     -2.47368154920465
 N   4.29679377912803     -2.20014005856168     -2.95305034558321
 O   3.32376733155088     -2.43006435813187     -3.65989296341261
 O   4.21306019422701     -2.00958438010059     -1.74861561310868
 O   5.45529180656324     -2.15202795808733     -3.50797416906605
 N   5.32839059250439     -1.60830723879048      0.95971535361368
 O   4.33658638267687     -2.15034275699388      1.46259638323811
 O   5.33714659727203     -0.36958351853674      0.79896267950977
 O   6.29701173940629     -2.28226465454062      0.58369364228740
 N   -1.17808088081151      1.45355368207023     -5.42607502653508
 H   -0.98716690207329      2.42484178148510     -5.18990003960137
 H   -2.15317364946501      1.28171161085736     -5.19178567886121
 H   -1.10474802496767      1.38155019731439     -6.43858926936536
```

p=7 [(NH4NO3)p (NH3)(NH4)]+   ωB97X-D3/def2-SVPD

```
 N   -0.79601571739177     -4.71610219928295     -2.26317716913598
 H   -1.66703002638180     -4.61810680705359     -1.69922906914527
 H   -0.72771792060936     -5.66487334660037     -2.62466978612367
 H   0.02535061399659     -4.51618933441741     -1.65324101573873
 H   -0.80815128147089     -4.03929408574218     -3.05382080814524
 N   -3.19789621383816     -1.03304468654650      2.77997409991594
 H   -2.24021854234700     -1.05566527126019      3.19445238486842
 H   -3.27046971533586     -1.78853969189663      2.07337091631494
 H   -3.33692432429118     -0.10995220144702      2.31820437439275
 H   -3.90267296490273     -1.17099884531814      3.50079571118212
 N   -4.15027896990743     -1.99871794650374     -2.05050988656031
 H   -3.55037376110109     -1.95046328761465     -2.89210058488743
 H   -3.87633148408274     -2.86308159588679     -1.52653906523230
 H   -5.13196788479257     -2.04324001763510     -2.31341544387462
 H   -3.98642212632405     -1.17896154436508     -1.44363230876510
 N   0.39831357929433     -3.53319071461300      2.36272821391921
 H   0.14115129460031     -2.68996627224462      2.92226313457984
 H   0.61464761499526     -4.31971157959568      2.97016535347268
 H   1.22131763255067     -3.30706666914861      1.77146691590895
 H   -0.40228260531743     -3.77589051532719      1.75516050047936
 N   -2.00314228028495      1.04541761579858     -2.94505488956721
 H   -1.77135186080487      0.09094776534975     -2.63245975878670
 H   -2.52940532683992      1.02188841878272     -3.85749631731611
 H   -1.10562775372356      1.53976611843877     -3.03946017258738
 H   -2.55173142942405      1.48710925450820     -2.19007378760706
 N   2.34483307999237      0.03272623126418      0.25978972428433
 H   2.56993385750404     -0.97429553065513      0.36867318095459
 H   2.80438692696985      0.45107540016922     -0.56077776430530
 H   2.55049246024819      0.52817796789050      1.12487035054307
 H   1.32261986641223      0.09454533215329      0.09365776562883
 N   -0.70295861226812      2.72309212879261      0.69547108380953
 H   -0.58052902570244      1.94599752719859      1.37687275273603
 H   -0.63117841309041      3.61231775679923      1.18524246016208
 H   -1.64421581773692      2.64001613345806      0.26540620724077
 H   0.01888399158538      2.66650192101754     -0.05009244354771
 N   1.72585612751036     -0.97432261684012     -3.94648744345751
 H   0.79240112790935     -0.83482733644480     -4.39204834207892
 H   1.63374449430963     -1.71945207116161     -3.24390407887725
 H   2.04648418476713     -0.10448408651168     -3.46241028286255
 H   2.40106086348560     -1.25992077601092     -4.65220811206959
 N   1.83307908080403     -3.14293277319721     -0.51290518307144
 O   1.28455286418440     -4.24928852177623     -0.51377266417791
 O   2.39149603231859     -2.74630077413681      0.53334333712741
 O   1.82383262760981     -2.41459990165650     -1.51021489352009
 N   0.00955859558713     -0.25593256159345      2.95517582351365
 O   1.20382714091965     -0.31363325056209      2.70298465350785
 O   -0.68524843240148      0.72365733987593      2.65612020586456
 O   -0.55123794845459     -1.22632304641132      3.53646413487862
 N   1.51563169387171      1.88492631341988     -2.16442957725884
 O   1.41574583705510      2.49422758266571     -1.09124576070292
 O   0.68228496823901      2.00555610949865     -3.06080719485446
 O   2.49590778590419      1.11910211658917     -2.33033008484230
 N   -2.84140895297773     -3.89714445142548      0.34776116763381
 O   -3.53958098352478     -3.02172918718618      0.86526607758419
 O   -1.91876729202863     -4.44611490776758      0.93969170654894
 O   -3.10404077915223     -4.23437096505058     -0.83827457443699
 N   -1.31899088844001     -1.95669599808675     -4.50536669157593
 O   -2.53808831351791     -2.13033296911557     -4.35860153655303
 O   -0.88955723956114     -0.84722609871836     -4.86348763864425
 O   -0.51852286835195     -2.88071614887910     -4.30050541148757
 N   -0.79790975846371     -1.05728373654069     -0.61579118256594
 O   -1.41693409467331     -1.48706111144837     -1.60620468408511
 O   -0.63140437220593     -1.76007971446961      0.37608933989987
 O   -0.33845805285183      0.10704482832145     -0.64689247714560
 N   -3.49219925909770      1.18026773154777      0.22842305286850
 O   -3.39707701888963      1.41147139616046      1.43700049385377
 O   -3.11937527033654      2.05487688447444     -0.58531621094619
 O   -3.92907343283455      0.09538388250632     -0.17133153368732
 N   -3.37133027902995      0.80588691554370     -5.43567261695548
 H   -2.73765623524392      1.13694639833884     -6.15887961749018
 H   -4.29754359341216      1.15387163512623     -5.66480071430501
 H   -3.38158705530566     -0.21029184464399     -5.50896320799587
```
