# Supplementary material for: Decomposition and Growth Pathways for Ammonium Nitrate Clusters and Nanoparticles
Source: J Phys Chem A. 2024 Oct 14;128(42):9184–94. doi: 10.1021/acs.jpca.4c04630 (PMC11514028; doi:10.1021/acs.jpca.4c04630)
Supplement: Supplementary file 2 — jp4c04630_si_002.zip [file jp4c04630_si_002.zip › SI_ammoniumnitrate particle structures_PDF_XYZ/HassanAmatTopper_SuppMats_S07.pdf]

Supporting Information for:

Decomposition and Growth Pathways for Ammonium Nitrate Clusters and Nanoparticles

Ubaidullah S. Hassan, Miguel A. Amat, and Robert Q. Topper*

Author Affiliations:

Ubaidullah S. Hassan, Department of Chemistry, The Cooper Union for the Advancement of Science and Art, New York NY 10003, United States.

Miguel A. Amat, Department of Chemistry, The Cooper Union for the Advancement of Science and Art, New York NY 10003, United States.

Robert Q. Topper, Department of Chemistry, The Cooper Union for the Advancement of Science and Art, New York NY 10003, United States. Email: topper@cooper.edu; Phone: 212-353-4370.

Table S7: Cartesian Coordinates of p=(0-7) $[(NH_4NO_3)_p(HNO_3)_2(NO_3)]^-$: ωB97X-D3/def2-SVPD

```
p=0 [(NH4NO3)p (HNO3)2(NO3)]-  ωB97X-D3/def2-SVPD
 N   0.78156235026842    -0.46973156671946    -2.67951433824289
 O   -0.45718403169933    -0.48511011541306    -2.88390100222925
 O   1.20235445081907    -0.23122102357994    -1.52042716655893
 O   1.56077273955720    -0.68193278973435    -3.58882598954390
 N   -0.50180560865726     1.11980223940417     1.04280152588049
 O   0.12188483130076     1.54406523834359    -0.05275752817883
 O   -0.62544876653999    -0.07264052674645     1.19546785257607
 O   -0.87849614194092     1.98377221921485     1.79137979855781
 H   0.41849693654912     0.73483774337669    -0.60250768245602
 N   -2.96008255237251    -0.70113558924801    -0.66128075444813
 O   -2.08732072100721    -1.52741495672733    -1.23168465642248
 O   -2.80985058162180     0.48535306866789    -0.83441836521090
 O   -3.83417328395470    -1.23106416612401    -0.02548981458896
 H   -1.42211662070083    -0.97907177471458    -1.78016687913406

p=1 [(NH4NO3)p (HNO3)2(NO3)]-  ωB97X-D3/def2-SVPD
 N   -0.18084720022594     0.98349313952338    -0.80103288837242
 H   -0.77682243630949     1.49806176678633    -1.45766252122877
 H   -0.56656780820621     1.05277056343654     0.14730689012802
 H   0.77103541062141     1.39684062871110    -0.86940068362816
 H   -0.17275241695656    -0.02476738147836    -1.05131590598850
 N   -1.12280053792368    -1.93837157881662     0.04455666140673
 O   -1.11197370862395    -2.86049133026315     0.83599879578064
 O   -2.00723216421070    -1.02141023642517     0.14224067276037
 O   -0.29495519114463    -1.83005637711222    -0.87049954508668
 N   1.81422521117039     2.95185051401814    -2.35259310515845
 O   1.01291802345002     2.26383401398712    -3.07066299811387
 O   2.19768471840598     4.03869437897181    -2.73667219036131
 O   2.16274061699732     2.46400701532537    -1.26758033621044
 N   -1.96763158859820     0.67945892168301     2.81115953124152
 O   -2.66971933413591    -0.37419547447753     2.44624543089374
 O   -1.14111240036280     1.12575096481274     2.03237356571734
 O   -2.20157388737799     1.12280875247835     3.90235803871700
 H   -2.37079551847302    -0.68652142986020     1.48386664896107
 N   -1.88241295009096     3.46948965588529    -3.52884133987124
 O   -0.77606049325411     3.53336504201437    -4.24528583430818
 O   -1.85537461597348     2.86211273029815    -2.47218140399324
 O   -2.84235804852050     4.02745602436297    -3.98610878371684
 H   -0.00014368025696     3.03126069613852    -3.74482969956829

p=2 [(NH4NO3)p (HNO3)2(NO3)]-  ωB97X-D3/def2-SVPD
 N   1.49027581307133     0.85005390075930     0.91456205730073
 H   1.35017419894425     1.87767743443510     0.77547571004915
 H   0.98150993251533     0.28334842981230     0.23712866737216
 H   1.12684119069934     0.59444959169055     1.84573828206720
 H   2.49187722824193     0.60808447365662     0.91989342142582
 N   2.09878848938509     2.17138817260362     4.99138040506367
 H   1.43290973266897     1.43443461842175     5.30900046196343
 H   1.66676894713896     2.61164137556989     4.12971654978391
 H   2.99738685709888     1.72204446575360     4.73261974029541
 H   2.24513967159830     2.88005631448796     5.70313091253722
 N   0.16316094577126    -0.35801633146231     4.30112945111190
 O   0.17822987713649     0.20274947103381     5.40533687246871
 O   -0.81106929212747    -1.06610309866666     3.98888034076101
 O   1.11426020605666    -0.21895609223548     3.51673334809583
 N   1.23441303708891     3.85606042465231     2.04664386695441
 O   1.25986799499749     3.61571007052593     0.82453872056947
 O   0.78477339609908     2.95839521649144     2.81424534658137
 O   1.62585911803666     4.91790373226425     2.50159445870837
 N   4.06355162024613     1.02404675118327     2.78017583899384
 O   4.29222533900301     0.71480446405268     3.95817211506719
 O   3.78762231911898     2.17151311847127     2.45904545345323
 O   4.08768075279601     0.12377638138109     1.88977753199905
 N   -0.80323893986800    -2.13295197866016     0.76477687769423
 O   -0.81074280254852    -2.64958966156954     1.98404255250693
 O   -0.74225858410412    -0.92247173198231     0.66616736495284
 O   -0.87356339308431    -2.91628219396792    -0.13898909074832
 H   -0.74211613118820    -1.89872741031375     2.67159469090003
 N   2.59846894273170    -2.74886642290664     1.94599651876498
 O   3.58847767795026    -2.27039540320110     2.69573054177272
 O   2.14582644728126    -2.02806447927339     1.08111643303656
 O   2.25242590315282    -3.86873005733356     2.19619278677553
 H   3.77288450409135    -1.31115154567399     2.40696577172109

p=3 [(NH4NO3)p (HNO3)2(NO3)]-  ωB97X-D3/def2-SVPD
 N   1.75040796522869    -1.11749196203672    -1.21827598501322
 H   2.51212720927924    -1.38747700429694    -1.83773647052870
 H   1.57551355120902    -0.10688330980028    -1.35461196380093
 H   2.00304891387881    -1.25508578944327    -0.21589588734639
 H   0.87067938333863    -1.64288392441465    -1.42040006818892
 N   -1.68532617326289    -2.28566472281055     2.79448410117252
 H   -2.02918096593704    -2.47828255250642     1.83141072414207
 H   -1.82145087150247    -1.28460153227220     3.01473005288172
 H   -0.66903778883194    -2.49871313930833     2.80672944101010
 H   -2.21095242032297    -2.82516833542689     3.48029005428075
 N   -0.18660308252372     1.33152996789166     1.38180343590123
 H   0.43578902805141     2.05650761551039     0.98531516443993
 H   0.28439953216080     0.79600484132277     2.13435095189520
 H   -0.41124818167214     0.65473032316490     0.64425866761231
 H   -1.07782294819711     1.71291715634255     1.74630165041176
 N   -1.19384932091914    -2.17027409867560    -0.37934302471525
 O   -0.65587744167954    -2.47699115805735    -1.45322849389420
 O   -0.79868903130583    -1.16547085062770     0.24667944311015
 O   -2.10869537192714    -2.85952137682754     0.08767068025684
 N   -3.04770832640610     1.13075707473710     3.14089831063662
 O   -2.12912669985056     0.40864491026846     3.56637106476526
 O   -4.20464495695959     0.99443349521914     3.59450150990466
 O   -2.82567605469417     1.98455671550622     2.28207752821034
 N   1.87922539703139     2.53207847035914    -0.99903530630350
 O   1.13225151149671     1.61939708336077    -1.40140804554332
 O   2.84424561825699     2.89670012547524    -1.70279559453711
 O   1.66939272082804     3.08145017326766     0.08071958237868
 N   1.38203476059904    -1.42480955002600     2.26153380515124
 O   1.07197210555951    -2.62448182448270     2.22307844364353
 O   0.83375849189644    -0.67137574993923     3.08394231050248
 O   2.22353343296045    -0.96305104766764     1.47018139269000
 N   3.48301877377177     0.65578314454639    -4.07412651658671
 O   3.10781980701359     1.92207189876232    -4.02298041414068
 O   3.64506866044066     0.07066870654235    -3.01979757237112
 O   3.63553019977313     0.19239215035216    -5.17065326439669
 H   2.98083612908984     2.20385872353588    -3.03621192153503
 N   -4.31253822437632    -1.73198701913482     5.52200389983808
 O   -4.50550536807125    -0.43248073069204     5.65801078500238
 O   -4.02662542913367    -2.14992740678152     4.41584991353757
 O   -4.44392859054261    -2.39370537355174     6.51506488747258
 H   -4.31846094374791     0.04389788261509     4.75782272805369

p=4 [(NH4NO3)p (HNO3)2(NO3)]-  ωB97X-D3/def2-SVPD
 N   -1.52701863795654    -0.72097531795321    -1.98512832963981
 H   -1.49627358935047    -1.02839457551212    -2.95886005014488
 H   -1.51578307600952    -1.56828062436903    -1.39884838143146
```

```
H   -2.40588682885626     -0.20309854259516     -1.78417591563088
H   -0.70121737162933     -0.12016883049239     -1.76687844349775
N    1.77489654434501      2.00277742318715      2.72291220289685
H    1.12550027616433      2.78036948216114      2.93379750440367
H    1.40623592503035      1.10966808460378      3.10558467304419
H    2.66286156571260      2.23206908193482      3.16922766604797
H    1.87651240185219      1.92082495364976      1.68237770473873
N   -2.58826800091072     -2.52942799829394      2.10917740005641
H   -1.61378117724419     -2.54126907203137      2.48663849774676
H   -3.23032618007160     -2.62135657163385      2.89162698879775
H   -2.74483820670392     -3.28888655517762      1.41008645422907
H   -2.75760493582743     -1.61743183683790      1.65034106920357
N   -1.88171356918559      1.89833193714991      2.42078691015608
H   -2.81729099667483      1.76759247324571      1.99142015677214
H   -1.86367413695280      2.70594248067410      3.06872445959772
H   -1.19598751119831      2.01294740076247      1.66318862317569
H   -1.61065426791194      1.03621650871992      2.92964228115069
N    0.85863960873300      1.23894850385514     -0.40090340775630
O    1.91687599933526      1.75833534845183     -0.01311375026476
O    0.76522291436825      0.80990969145943     -1.55705724924737
O   -0.10988401265194      1.13887594789464      0.37711150340143
N   -0.05385913774547     -1.09160064501296      3.50207272643333
O   -1.14398335387883     -0.49337370668297      3.62999212373799
O   -0.05839732528740     -2.29745740133143      3.21862671015354
O    1.01397409921413     -0.49241385123544      3.66274957329777
N   -0.49939380032676      4.35707150835422      4.29392974690049
O    0.11008703792386      4.18896440105048      3.21843588117841
O   -1.62452754295572      3.88350460587494      4.45064255008917
O    0.03586035755687      5.01091521768740      5.20925965249357
N   -2.36984839859102     -4.16565581999566     -0.80204882308843
O   -1.49538168876801     -3.29266237263898     -0.69541023241539
O   -2.50324585847928     -4.79675727587292     -1.86808808107300
O   -3.11372643537988     -4.42549575516488      0.15182144221021
N   -3.66340454960949      0.62796239801085      0.05909387629316
O   -2.99443392830732     -0.32719963976247      0.50766701734973
O   -3.80276846233077      0.74312376568873     -1.16431930664093
O   -4.15320046880346      1.45762276057285      0.83680183300901
N    3.29731068670075      4.96338604778471      4.54943949532641
O    2.38320501738744      5.89752754578828      4.75890736192268
O    2.94832388005205      3.80137903079200      4.63909005066270
O    4.40188579779687      5.35350539283307      4.29263766563176
H    1.46864619436625      5.44804350689128      4.89872878353147
N   -0.74658797896845     -3.50896116387758     -4.39665651461939
O   -0.71751303504096     -4.60922018171369     -3.66228432338397
O   -1.59798805897872     -2.67843455412137     -4.13380378552656
O    0.07259154889737     -3.43203069443472     -5.26731184308907
H   -1.44666433284955     -4.55336051233694     -2.93859414819039
```

p=5 [(NH4NO3)p (HNO3)2(NO3)]- ωB97X-D3/def2-SVPD

```
N    2.78097705656211      6.18647212950815      0.00967840633954
H    2.07830733911093      5.54244430772994      0.41081649926595
H    3.48472199930286      6.32129735488588      0.74892976111989
H    2.26940300078918      7.05894712213373     -0.20223526776942
H    3.14217757438484      5.70616919661961     -0.82096603109420
N   -0.66952190920682      6.70840830146867      2.78361019557829
H    0.33317459619447      6.99025784153143      2.87268794838412
H   -0.98131631383239      6.87309063337185      1.80042674183407
H   -1.25542710848759      7.22705269737083      3.43011965222819
H   -0.71363764786812      5.69313496830501      2.97765061231887
N    0.83676358992369      0.20739927095585      0.40831072081463
H    1.54039653181689      0.81446338063772     -0.05406038277031
H    1.30304535578212     -0.58821732097602      0.86225375776630
H    0.12041344058883     -0.03348706981518     -0.29707080412592
H    0.39913523183025      0.77750829427895      1.14003392864831
N    3.03331137821618      2.93296557685267      2.90307256190638
H    3.05698331664041      3.06266475159381      1.87647029363852
H    2.79133923132471      3.83053983929941      3.37450180248939
H    3.96282290850327      2.61170895266333      3.18821356615929
H    2.35124100550270      2.19372356304536      3.14108466928438
N   -0.19502778095565      3.77261029565182     -1.51231579571453
H    0.04845801460136      3.40746906734232     -0.57559157242096
H   -1.08051008049780      3.33574643535024     -1.80796849438174
H    0.58090211364848      3.53304568276542     -2.13530506307837
H   -0.26973163972079      4.81201947656065     -1.45740601493186
N    2.85231752264651      3.06016269400423     -0.92999920852841
O    3.48559663125230      3.28377743768256      0.11270471729008
O    2.35013652094828      1.94757837144939     -1.14869142637993
O    2.70746082982420      3.96670735871228     -1.77044252379503
N   -0.16768334843164      7.19227252492098     -0.45215793121464
O    0.65236387261104      7.99091854726291      0.02580150748018
O    0.15100912176624      6.51024198130153     -1.44787707925420
O   -1.28346872451536      7.05407189337356      0.06710718323150
N    0.28863156992073      3.85111560482942      1.76622761464341
O    0.71855455578646      4.92766773968281      1.30949741199425
O   -0.33653511912597      3.83306102651198      2.82185342539694
O    0.49968612421335      2.80588715092653      1.12400394729835
N   -1.92930795569362      1.16895053060185     -1.09685225610492
O   -2.22953622122245      0.91310449974890      0.09507822499118
O   -1.02352201007167      0.51956845299843     -1.63726943749516
O   -2.51053087288698      2.07225373626568     -1.70269591229176
N    2.67970395573451      6.19604326321297      3.15836326338417
O    2.17760475678924      5.39571951685454      3.96754491715276
O    3.76209457485332      5.92422553472558      2.61389387542229
O    2.08580099322838      7.24930418706891      2.87331377986654
N    2.61711282517706     -0.20910514252131      3.00811286262373
O    2.65618892650980     -1.13013803974267      2.19038128150868
O    3.62760247013582      0.07200483407750      3.69785861863224
O    1.59505893866523      0.47668346740568      3.15407369471685
N   -2.99316688412024      3.85381354368432      1.43053533596301
O   -3.52895190220616      2.63956508701839      1.48933018596801
O   -2.15394959657019      4.05822659261002      0.58588608850457
O   -3.40477906278617      4.64856351284630      2.23425872727534
H   -3.02324149214299      2.03732785066985      0.83869334608405
N    6.54270865723859      0.91093125756115      2.64955088228418
O    5.97225191991228     -0.28618713857777      2.75972073307749
O    5.87632407216797      1.88908374156954      2.92171596243634
O    7.68471387120300      0.91070398292659      2.28774321335039
H    5.01623827503457     -0.16381335079514      3.09950828299846
```

p=6 [(NH4NO3)p (HNO3)2(NO3)]- ωB97X-D3/def2-SVPD

```
N    4.22968617540867      0.50284590156296     -1.07537981963677
H    3.24172511454392      0.76732460952402     -0.95829402931320
H    4.73523287110549      0.94326818549850     -0.29122812625515
H    4.61876546518431      0.84816960504408     -1.95896488019542
H    4.33667816747567     -0.52758671271092     -0.99373983004232
N    4.94067045306594     -2.41771065637945      3.71908190638726
H    5.76347524257360     -1.80410249057649      3.89770478797025
H    5.08939440630765     -3.34002601973635      4.12256371421777
H    4.76538266157383     -2.54051642072659      2.70958104878733
H    4.06971455375090     -2.02899213152010      4.14420942008917
N    6.79009096218420      3.15054888161545      2.82435145650701
H    7.33268725933579      3.49797389380897      2.02351372806545
H    6.31293120808320      3.91185070355602      3.31782114334756
H    7.35143480854341      2.55718824300257      3.46502071562036
H    6.03835850215292      2.55550715362531      2.43125560054648
N    3.87172355528757      2.04270941605678      5.58766711022413
H    3.39508698241914      1.32989364940842      6.16326203749909
H    4.86420839668769      1.79465525443432      5.50260425158708
H    3.74552123438123      3.02611264396833      5.89102886947969
H    3.51443988265800      1.94683278658784      4.62601104368506
N    3.03210302934615      4.10793429450594      1.46019369299514
H    2.95181971604378      4.88044061216385      2.14304809259822
H    3.88592374113857      4.23623125916701      0.89289752052263
H    2.19315052982095      3.99954733933359      0.86221517662502
H    3.15195292848471      3.23370922827233      1.99443298303238
N    1.07264616260698     -0.62539169868451      2.22400699505791
H    0.97542869707249     -0.29790644960463      3.20196633102540
H    0.55613559792795     -0.02475898343299      1.55184219429316
H    0.78917269300607     -1.60465162700646      2.16638666642315
H    2.07855734204105     -0.58491188404849      1.99891043479452
N    3.66148948508568      4.97682004661511      4.50620799457953
O    3.40835377047734      4.81106374758858      5.70859703426653
O    2.98608225017994      5.74273257707847      3.81168245817740
O    4.59490200000854      4.32571851176409      3.98301288164296
N    2.25354195594295     -0.67283128021549      5.27195055111070
O    2.43730399120862     -1.67691648222062      4.55291483609542
O    2.98250666734320     -0.47492128269619      6.25231821178266
O    1.35275872668534      0.12718894880458      4.98192871686503
N    7.06312824399402      0.26834377785592      4.26903884606467
O    5.88095186032298      0.67470329638841      4.30219367471636
O    7.99347577868554      1.08566363229421      4.28830677205278
O    7.28887901798159     -0.94551902531664      4.20650065079247
N    4.04524911331056      0.93214843607255      2.09474345639800
O    3.28004255121624      1.54887357996959      2.85283641653980
O    3.91561433837003     -0.27641123002651      1.92115281657984
O    4.92961357531803      1.55787015232489      1.47357912850912
N    0.84779845415527      2.04168875714324      0.28247416619308
O    0.67357799429300      3.25901606861566      0.15325723995092
O   -0.10219553521847      1.28890103246652      0.53262764914225
O    2.00339069770015      1.56982163724909      0.18611591337664
N    3.66502151325655     -2.47176482569839      0.23471005925399
O    4.66410369495149     -2.18584017603743     -0.42480521615111
O    2.55477242802226     -2.03119770382105     -0.04186082108084
O    3.79849204500900     -3.23590329033133      1.23970691319911
N    6.26811178535890      3.66166364672636     -0.24391246559733
O    5.62945647167504      4.44827998600999      0.46228724154637
O    5.70828933708533      3.13238455138341     -1.23820636962345
O    7.43794936052710      3.38295216799190      0.02321672027237
N    6.72437692284968      1.49100557672980     -3.85602131625710
O    7.35656294654856      2.26731519969541     -2.98914537877630
O    5.58471332069019      1.14855837956001     -3.58875768716520
O    7.33543625903506      1.18824369533965     -4.83890773886230
H    6.69859813984506      2.54619447270489     -2.25132482406472
N    1.70853948429389     -4.23043040135959      3.53919381033650
O    1.53200865153142     -3.63606265915311      2.35212089684514
O    2.83754433881727     -4.55665269645616      3.83027772049036
O    0.71781219307775     -4.39305861406886      4.18869322104025
H    2.45331283015128     -3.52003079768066      1.91110158381895
```

p=7 [(NH4NO3)p (HNO3)2(NO3)]- ωB97X-D3/def2-SVPD

```
  N   -0.46413412214705      3.30400333696796      8.20053718272033
H   -0.29849972988144      2.71269604675407      7.36442274995909
H   -1.46367834808625      3.27235779347766      8.40251938236071
H   -0.12514184927071      4.24494081694123      7.93624586794477
H    0.10321251731584      2.89577500915273      8.96485679809451
N   -0.69960954814545     -2.06139987664887      5.21943170815213
H    0.03268540082605     -2.37392694381828      5.87360508057889
H   -1.21220165805903     -2.87291285848050      4.86314042951510
H   -0.25121939081487     -1.61892204028346      4.38806735517824
H   -1.31502223933994     -1.37881381561439      5.67249701993673
N    3.50491956687492     -0.80300340262997      6.60678142936157
H    4.03616535039327     -0.23070130880396      5.93798250094402
H    3.38767278393030     -0.30169822409440      7.50496994939367
H    3.95243153099557     -1.71715520229816      6.75006537456669
H    2.56836342532801     -0.98112068410019      6.22652122358731
N    3.09175594427245      3.48235075392750      5.44942823630777
H    4.05905268990907      3.49463011474630      5.07233160093626
H    2.64283674706316      2.61784612264261      5.10082333255519
H    2.51645010635992      4.28602994822893      5.14012880610935
H    3.09143708259050      3.42492367836215      6.46963943977367
N   -0.18551718560408     -0.68691430374577      9.01937086667981
H   -1.14935059090653     -0.30893346113041      9.00176080781855
H    0.37139877964757     -0.14993169761671      9.69291945446825
H    0.20779777692089     -0.50197259359772      8.08616255787121
H   -0.11887788759963     -1.71290117748885      9.18908087081659
N    3.14175024891291     -0.37976235287468      2.62113675741148
H    3.13838800332822     -1.39829430122130      2.43355344030679
H    2.77886333147765     -0.26125232823829      3.57318154707236
H    2.46527756809424      0.08849135519338      2.00425413539488
H    4.08729061650667      0.04429531441088      2.57895923783622
N   -1.98385708551547      2.83271126707471      4.28710054853051
H   -1.42183427116110      2.09477909286581      3.80825003055943
H   -2.73461869426383      3.14008774124508      3.67615977897413
H   -1.41362268219277      3.64924516408756      4.58383948291670
H   -2.38338930936177      2.41816551862503      5.15738003488842
N    2.13382299988439      1.44259852997004      8.93983213612903
O    1.22673920387189      1.71238611322793      9.76179306143409
O    2.38803616301397      2.22906127928245      8.02320943603449
O    2.73817006321429      0.36753019662114      9.04936526839207
N    2.08457779747670     -3.14939137424517      3.70743341387056
O    2.57762971711325     -2.43935262206097      4.58913015987291
O    1.08369190631912     -3.85380579802303      3.98914589620682
O    2.54569747261214     -3.15869408211700      2.56569281678080
N    0.00345865899199      0.12562430897727      2.69982104346014
O   -0.99333150658712      0.50654395845721      3.34719686772553
```

O	0.57697864993900	0.87480949598483	1.91186776765933
O	0.41651905425372	-1.03563661442471	2.88066735755770
N	-2.54379533087976	1.01068823385618	7.33216588638320
O	-2.87233152898919	2.08706601054953	6.79494945249969
O	-2.44270555556254	-0.02246242889492	6.67207258915450
O	-2.31105836693347	1.00933831206772	8.55874516978017
N	0.33267482548884	5.12446115371143	5.69496178746605
O	0.96747072441222	5.12739051655080	4.63336560993087
O	-0.90196477105594	4.95746434459585	5.68999616685254
O	0.92597522684062	5.26302625347894	6.77975573942320
N	5.23896244660347	1.73898997942765	3.93113684641111
O	5.45719348534700	2.95453294137200	4.04415391648223
O	5.48331419885935	1.14923645789337	2.87023039245978
O	4.73874353137172	1.11861337275550	4.89185225791777
N	0.90440605844219	1.04742092166717	5.68230734971555
O	0.73153253697557	0.04348021165028	6.38509305372956
O	1.78855201465812	1.03817675139259	4.81218696688786
O	0.21868261218050	2.06407309036226	5.84278110007040
N	1.02957228655578	-3.60997529133961	8.22663516775069
O	1.16714747284608	-2.72242683022398	7.36492022784121
O	1.61852272768989	-4.69439143298659	8.10353839000195
O	0.30589344627760	-3.41258908182404	9.21255350522179
N	-1.61620878356551	-4.62195042437186	2.43938516155754
O	-0.34925649461804	-4.67581589713585	2.07087492072489
O	-1.87896423785856	-4.18226269453709	3.54591929106025
O	-2.41608778125797	-5.02311352019519	1.64133443391496
H	0.24897793041669	-4.32632635638865	2.83825381166245
N	4.25987449855546	-4.29411831383583	6.11255949955105
O	3.19846754501349	-5.08024019211420	6.11174763106976
O	4.36954456382018	-3.49858183607823	7.03300033415442
O	5.03235152036286	-4.44223994197915	5.21251362386491
H	2.59449811630686	-4.81119760977298	6.88886074725743
