# Supplementary material for: Decomposition and Growth Pathways for Ammonium Nitrate Clusters and Nanoparticles
Source: J Phys Chem A. 2024 Oct 14;128(42):9184–94. doi: 10.1021/acs.jpca.4c04630 (PMC11514028; doi:10.1021/acs.jpca.4c04630)
Supplement: Supplementary file 2 — jp4c04630_si_002.zip [file jp4c04630_si_002.zip › SI_ammoniumnitrate particle structures_PDF_XYZ/HassanAmatTopper_SuppMats_S16.pdf]

Supporting Information for:

Decomposition and Growth Pathways for Ammonium Nitrate Clusters and Nanoparticles

Ubaidullah S. Hassan, Miguel A. Amat, and Robert Q. Topper*

Author Affiliations:

Ubaidullah S. Hassan, Department of Chemistry, The Cooper Union for the Advancement of Science and Art, New York NY 10003, United States.

Miguel A. Amat, Department of Chemistry, The Cooper Union for the Advancement of Science and Art, New York NY 10003, United States.

Robert Q. Topper, Department of Chemistry, The Cooper Union for the Advancement of Science and Art, New York NY 10003, United States. Email: topper@cooper.edu; Phone: 212-353-4370.

Table S16: Cartesian Coordinates of n=(1-16) Neutral Clusters: ωB97X-D3/6-31G(d)

n=1 (NH4NO3)n wB97X-D3/6-31G(d)

N	0.011765	-0.000957	-1.512181
H	-0.639319	-0.005613	0.005184
H	1.006305	-0.000687	-1.298011
H	-0.188589	0.821963	-2.073137
H	-0.184160	-0.819112	-2.082134
N	0.052697	0.000987	1.780790
O	-0.200773	0.002736	2.957037
O	1.157855	-0.002075	1.262731
O	-1.015780	0.002758	0.959721

n=2 (NH4NO3)n wB97X-D3/6-31G(d)

N	-0.004455	2.373250	-0.295840
H	-0.000574	1.821165	-1.157321
H	-0.010445	3.372962	-0.485754
H	0.860406	2.073959	0.230289
H	-0.865752	2.063438	0.229640
N	0.004365	-2.373162	-0.295882
H	0.004731	-3.372783	-0.485777
H	0.000597	-1.821188	-1.157236
H	0.868683	-2.068499	0.228348
H	-0.857631	-2.068929	0.231418
N	1.856814	0.004559	0.365905
O	2.042255	1.086423	0.967603
O	2.044678	-1.077674	0.966014
O	1.462012	0.004925	-0.818539
N	-1.857123	-0.004615	0.365116
O	-2.041872	-1.086591	0.966519
O	-1.462388	-0.005048	-0.819294
O	-2.044301	1.077807	0.964791

n=3 (NH4NO3)n wB97X-D3/6-31G(d)

N	-2.354137	-0.060410	0.481550
H	-2.355833	-0.362463	-0.543418
H	-3.296360	-0.015553	0.861065
H	-1.907512	0.871268	0.482787
H	-1.744402	-0.701553	1.021599
N	0.937381	-0.216923	-2.177452
H	1.717533	-0.508349	-2.761557
H	0.512343	-1.038851	-1.710078
H	0.184231	0.258880	-2.747725
H	1.268641	0.426522	-1.430582
N	2.225622	-0.075996	2.691586
H	1.513186	0.673314	2.467593
H	3.160589	0.320278	2.626090
H	2.100961	-0.834507	1.961531
H	2.058866	-0.461194	3.618906
N	0.541724	1.527553	0.600696
O	-0.372554	1.714193	-0.215129
O	1.676747	1.166828	0.205598
O	0.335962	1.671059	1.830721
N	-2.021078	0.695027	-2.527930
O	-1.893151	-0.491891	-2.082051
O	-1.159361	1.108784	-3.338722
O	-2.948147	1.399654	-2.144432
N	0.450326	-1.774290	0.703478
O	-0.192823	-2.055371	-0.317515
O	1.704700	-1.850464	0.705105
O	-0.143453	-1.385547	1.738285

n=4 (NH4NO3)n wB97X-D3/6-31G(d)

N	1.163914	1.073066	-2.518718
H	1.561536	1.439100	-3.380831
H	0.841555	1.859191	-1.910888
H	1.881229	0.517277	-2.001157
H	0.348760	0.456291	-2.734028
N	1.073863	-2.727540	0.507130
H	0.165316	-2.602723	1.007443
H	1.749166	-2.006978	0.847772
H	0.918143	-2.588028	-0.516435
H	1.442495	-3.660021	0.679699
N	-2.960788	-0.131836	-0.251141
H	-2.598387	0.721983	-0.731523
H	-2.689243	-0.093836	0.757010
H	-2.525108	-0.975387	-0.686932
H	-3.973509	-0.177877	-0.338614
N	0.723410	1.786912	2.263611
H	-0.110772	2.165285	1.761388
H	1.520579	1.719036	1.591868
H	0.498910	0.830799	2.619414
H	0.971133	2.398316	3.038378
N	-0.650886	-1.608322	-2.038942
O	-1.419593	-2.236830	-1.280005
O	-1.071032	-0.615345	-2.671003
O	0.547521	-1.949349	-2.135728
N	2.665103	0.117789	0.226196
O	2.772120	-0.508003	-0.850300
O	2.596619	1.365724	0.219078
O	2.587414	-0.505930	1.306466
N	-1.047411	-0.965857	2.268823
O	0.078797	-0.888850	2.805108

O	-1.379045	-2.004897	1.658551
O	-1.826563	0.010515	2.309403
N	-0.967342	2.455550	-0.456972
O	-1.594889	2.009702	-1.441553
O	0.200613	2.876966	-0.599176
O	-1.493628	2.444109	0.676608

n=5 (NH4NO3)n wB97X-D3/6-31G(d)

N	0.892788	3.737580	0.498744
H	1.264102	2.978788	1.102081
H	1.635439	4.068442	-0.174517
H	0.541265	4.498440	1.074459
H	0.121892	3.352571	-0.072067
N	0.768818	0.722529	-2.526698
H	0.351889	-0.001113	-3.128089
H	1.315560	0.252420	-1.800721
H	0.026567	1.258917	-2.049072
H	1.394742	1.394116	-3.026764
N	-2.744829	-1.930699	-0.952948
H	-2.121522	-2.555814	-1.510012
H	-2.971428	-2.394649	-0.037221
H	-3.591862	-1.710044	-1.472337
H	-2.215463	-1.063873	-0.738437
N	-0.451891	-0.120835	3.380358
H	0.452147	-0.461856	3.003540
H	-0.292385	0.365766	4.260004
H	-1.122773	-0.916167	3.498454
H	-0.832792	0.553576	2.680260
N	1.193556	-2.751295	0.293439
H	1.522281	-1.876521	0.767307
H	0.189670	-2.861490	0.507489
H	1.701255	-3.553934	0.660213
H	1.323089	-2.682195	-0.739432
N	1.823039	0.574300	1.353476
O	1.857210	-0.594696	1.846778
O	1.880347	0.740477	0.132526
O	1.712275	1.534432	2.136827
N	-0.063053	-2.350473	-2.545495
O	1.169019	-2.163185	-2.405113
O	-0.745836	-1.573448	-3.234184
O	-0.609600	-3.307295	-1.944154
N	-2.271691	-2.484700	2.231519
O	-3.248749	-2.875486	1.561993
O	-1.114486	-2.517811	1.754677
O	-2.443999	-2.040501	3.382234
N	2.068365	3.444784	-2.289481
O	0.822410	3.532161	-2.281018
O	2.745051	4.104218	-1.463363
O	2.637562	2.673886	-3.090036
N	-1.147753	1.248774	0.334852
O	-1.172875	1.799582	1.455053
O	-1.109078	1.952836	-0.693828
O	-1.138272	-0.000515	0.258703

n=6 (NH4NO3)n wB97X-D3/6-31G(d)

N	-0.202716	-1.469378	3.740950
H	-0.905397	-2.117394	3.311565
H	0.644425	-1.422631	3.128152
H	0.062926	-1.813747	4.660407
H	-0.617416	-0.517519	3.815007
N	-3.454560	-1.270255	0.473477
H	-3.367469	-0.947042	1.462482
H	-2.999781	-0.558371	-0.127778
H	-4.433214	-1.391652	0.226527
H	-2.928112	-2.168194	0.376729
N	-0.839738	1.388179	-3.548660
H	-0.343147	0.488266	-3.698310
H	-1.479076	1.588994	-4.313518
H	-0.127519	2.146757	-3.444639
H	-1.345268	1.295373	-2.648501
N	-0.122136	2.684383	1.209269
H	0.587143	1.932808	1.183711
H	0.149458	3.345748	1.933252
H	-1.050974	2.277085	1.454988
H	-0.174796	3.160123	0.286098
N	1.134122	-2.631517	-0.418478
H	0.638490	-3.523400	-0.344574
H	1.564636	-2.428802	-1.343985
H	1.833544	-2.562464	0.332314
H	0.443402	-1.892910	-0.205969
N	3.232024	1.443329	-1.581186
H	2.832236	2.388948	-1.414011
H	2.990209	0.826072	-0.774620
H	4.244067	1.489429	-1.674805
H	2.816572	1.044347	-2.452851
N	1.549284	-0.793496	-3.182662
O	0.370570	-1.197677	-3.256558
O	2.414322	-1.450831	-2.572981
O	1.858518	0.302448	-3.709508
N	-0.719860	0.213871	-0.557996
O	0.318103	0.740752	-0.977848
O	-1.829311	0.490796	-1.087139
O	-0.689747	-0.593774	0.396592
N	-2.251500	0.760318	2.726769
O	-1.264239	1.100934	3.416862
O	-2.891913	-0.267246	3.022389
O	-2.574053	1.439929	1.728192
N	0.820238	3.515695	-1.759288
O	1.708408	3.659181	-0.886715
O	1.144178	3.219919	-2.928238
O	-0.380293	3.635814	-1.445275
N	2.199436	-0.398435	1.459934
O	1.768931	0.658770	1.938448
O	2.853648	-0.433165	0.399155
O	1.988801	-1.492941	2.045950
N	-1.288107	-3.484581	1.410684
O	-0.156882	-3.917990	1.664354
O	-1.716570	-3.475371	0.228918
O	-2.013900	-3.017482	2.322917

n=7 (NH4NO3)n wB97X-D3/6-31G(d)

N	-2.692452	-2.424958	0.986870
H	-2.381867	-2.455929	1.984081
H	-3.380967	-3.154713	0.815503
H	-1.881852	-2.583013	0.373745
H	-3.080229	-1.487161	0.731188
N	-1.203121	3.927730	0.105547
H	-0.364688	3.967143	0.725985
H	-1.649588	4.840569	0.056054
H	-1.884682	3.233015	0.461356
H	-0.905964	3.614782	-0.846806
N	-0.815931	0.249510	3.375499
H	0.070281	0.710309	3.622019
H	-0.975150	-0.656822	3.846100
H	-0.761010	0.020141	2.363930
H	-1.587499	0.905920	3.495266
N	-2.377194	0.613501	-2.544611
H	-1.813326	1.487842	-2.497230
H	-2.822625	0.417028	-1.620285
H	-1.758262	-0.179895	-2.821494
H	-3.103833	0.726545	-3.248475
N	2.874821	1.658482	0.409763
H	2.205608	1.681142	-0.375023
H	2.388830	1.886339	1.297489
H	3.592603	2.356000	0.226431
H	3.304101	0.709815	0.475506
N	2.216628	-0.822064	-3.285970
H	2.432201	-0.964612	-4.269878
H	1.611894	0.020440	-3.184892
H	3.086483	-0.685876	-2.725913
H	1.701606	-1.648635	-2.929211
N	1.443024	-3.041504	0.436502
H	1.000462	-3.241923	-0.476879
H	1.129306	-2.093431	0.694137
H	2.462627	-3.031329	0.299863
H	1.096407	-3.669039	1.176825
N	-0.524021	-2.228538	-2.336288
O	-1.418141	-2.307650	-1.481595
O	0.531968	-2.902796	-2.230879
O	-0.653400	-1.469259	-3.318289
N	0.645524	2.881708	2.466001
O	1.092394	3.662221	1.589813
O	-0.485613	3.033199	2.932794
O	1.376222	1.921084	2.832606
N	-3.308757	0.967782	0.678867
O	-3.284761	2.097489	0.164357
O	-3.515433	-0.029363	-0.086916
O	-3.154269	0.777107	1.883483
N	-0.401966	-2.934759	3.039304
O	-0.477539	-3.893783	2.240026
O	-1.487483	-2.432174	3.478517
O	0.676847	-2.450030	3.374198
N	0.396707	2.297883	-2.520030
O	-0.720945	2.890952	-2.401518
O	1.368166	2.680592	-1.867442
O	0.453921	1.345956	-3.317532
N	3.862479	-1.228574	-0.616447
O	4.126376	-0.277941	-1.395316
O	3.722218	-0.995986	0.604630
O	3.708710	-2.377704	-1.060805
N	0.071646	0.031725	0.042035
O	0.976598	-0.246197	-0.761945
O	-0.488921	1.134042	0.032371

O	-0.265169	-0.832336	0.903007

n=8 (NH4NO3)n wB97X-D3/6-31G(d)

N	-1.695423	0.233549	-3.196833
H	-1.750983	1.168203	-3.644108
H	-2.604700	-0.267931	-3.193843
H	-1.404312	0.394769	-2.224963
H	-0.966475	-0.362886	-3.625757
N	-2.106318	-3.680630	-1.394497
H	-3.009397	-3.216044	-1.144556
H	-1.594003	-3.118129	-2.088162
H	-2.279185	-4.608466	-1.775043
H	-1.495585	-3.735055	-0.545892
N	-2.960170	-0.341046	1.226992
H	-2.116653	-0.326452	0.625187
H	-3.038113	-1.302250	1.581068
H	-3.757919	-0.111831	0.617278
H	-2.805740	0.332134	1.993537
N	-0.035743	4.273473	0.153500
H	0.906308	3.890153	-0.074931
H	0.041856	5.132808	0.692880
H	-0.561561	3.557784	0.697379
H	-0.543196	4.436497	-0.749642
N	0.033108	-0.783509	4.324943
H	-0.545399	0.038594	4.061860
H	1.051328	-0.580375	4.183363
H	-0.222201	-1.590510	3.721781
H	-0.130316	-1.031015	5.299169
N	2.444281	1.709075	1.843828
H	1.568495	2.144602	2.164016
H	3.103426	2.420943	1.481611
H	2.211180	1.053370	1.080236
H	2.826269	1.136702	2.618479
N	2.301530	-2.881041	0.144373
H	2.789451	-2.169668	0.733165
H	1.364777	-3.092028	0.543915
H	2.848955	-3.740038	0.157495
H	2.206061	-2.540367	-0.835793
N	1.831655	1.146669	-2.741584
H	1.808521	0.462951	-3.506187
H	1.022496	1.789779	-2.771819
H	2.702578	1.702870	-2.671876
H	1.719927	0.624245	-1.855651
N	3.420336	3.046451	-0.763792
O	4.026925	3.445336	0.244220
O	4.014277	2.757847	-1.812025
O	2.161011	2.894484	-0.712908
N	-1.099330	1.978533	2.620402
O	-1.751121	1.157506	3.316248
O	-1.544738	2.303228	1.503598
O	-0.033797	2.444856	3.054806
N	-1.208869	3.351124	-2.723187
O	-1.708242	2.990565	-3.799414
O	-0.487267	2.550162	-2.060319
O	-1.408993	4.493824	-2.274058
N	0.251074	-0.291589	0.252985
O	0.082414	-0.377172	1.476870
O	-0.710680	-0.444236	-0.533367
O	1.398343	-0.066340	-0.200477
N	2.874733	-1.247517	2.890682
O	2.399111	-2.355890	3.115641
O	3.520962	-1.006439	1.846565
O	2.692081	-0.295900	3.717883
N	-4.047351	-1.211279	-1.565281
O	-4.164302	-0.002822	-1.302840
O	-4.231216	-2.072212	-0.670501
O	-3.699610	-1.585534	-2.710174
N	1.023287	-1.909257	-2.932586
O	0.232196	-2.722867	-2.431798
O	0.663904	-1.154322	-3.872463
O	2.191558	-1.824286	-2.505292
N	-1.010428	-3.241957	1.747341
O	-0.426638	-3.092547	2.823831
O	-2.229278	-3.051180	1.618959
O	-0.349167	-3.630472	0.735538

n=9 (NH4NO3)n wB97X-D3/6-31G(d)

N	-2.545450	3.622676	-2.637039
H	-2.420820	4.528311	-3.081994
H	-2.973803	3.742437	-1.695784
H	-1.616190	3.197828	-2.454530
H	-3.094610	2.965106	-3.222662
N	-2.001972	1.646876	2.929848
H	-2.509730	1.823656	3.794129
H	-2.378146	0.784077	2.500408
H	-1.004157	1.481200	3.162197
H	-2.046198	2.480129	2.301418
N	-0.299498	-4.155722	0.606479
H	-0.952822	-3.490076	1.046990
H	-0.667790	-5.101100	0.685853
H	0.602383	-4.072907	1.131246
H	-0.127809	-3.918729	-0.398477
N	1.078580	3.010508	0.458467
H	1.213413	3.062034	1.475446
H	0.544713	3.819438	0.110385
H	0.524231	2.160269	0.276601
H	2.002466	2.898759	0.005502
N	-1.640304	-0.863088	-4.893611
H	-1.049329	-1.650292	-4.544558
H	-2.572759	-0.887446	-4.425730
H	-1.746127	-0.898109	-5.904278
H	-1.183446	0.030055	-4.602192
N	-2.370354	-0.868003	-0.569731
H	-3.067071	-0.254809	-1.034115
H	-1.559686	-0.293307	-0.290055
H	-2.058761	-1.598503	-1.224284
H	-2.748388	-1.255970	0.306307
N	2.186493	-1.424619	-1.752606
H	2.128060	-2.185364	-2.432131
H	2.093159	-1.838765	-0.814561
H	1.409211	-0.766906	-1.925388
H	3.078623	-0.893692	-1.787051
N	1.099809	-1.866051	4.215906
H	1.403749	-2.570655	3.507020
H	1.681592	-1.005090	4.124955
H	1.224167	-2.252443	5.149371
H	0.096318	-1.619133	4.078476
N	4.368446	0.415811	1.782140
H	3.859151	0.644428	2.659779
H	4.176123	1.134533	1.048888
H	4.008314	-0.505700	1.483905
H	5.370429	0.348606	1.947494
N	1.743954	1.330886	3.620490
O	2.640050	0.495861	3.947584
O	2.070039	2.377570	3.056762
O	0.563972	1.050992	3.897127
N	0.577527	-0.296269	0.815202
O	1.752666	0.091639	0.883377
O	-0.363286	0.535484	0.907969
O	0.310916	-1.500025	0.635086
N	2.721666	-2.987918	1.582725
O	2.850910	-3.188533	0.374093
O	3.396448	-2.151732	2.199495
O	1.866625	-3.668217	2.238916
N	-2.059033	-1.613325	2.843169
O	-1.737745	-2.774927	2.553983
O	-1.559641	-1.046052	3.836814
O	-2.902212	-0.988148	2.152690
N	-1.850202	4.133262	0.396307
O	-1.111492	4.687819	-0.443026
O	-1.400224	3.887687	1.540870
O	-3.018141	3.809491	0.104379
N	-0.287503	-3.095382	-2.760470
O	0.502348	-3.655084	-1.963965
O	0.160874	-2.703143	-3.857819
O	-1.484292	-2.934262	-2.463068
N	3.490033	1.346510	-1.142914
O	3.695650	2.211877	-0.239779
O	4.228024	0.333769	-1.169589
O	2.571075	1.498943	-1.945385
N	-3.773597	0.671357	-3.081071
O	-3.800593	-0.572479	-3.230703
O	-3.710093	1.425996	-4.070539
O	-3.778364	1.148948	-1.924874
N	-0.442856	1.145426	-2.595014
O	-0.237048	-0.059855	-2.323559
O	-0.455488	2.005133	-1.700240
O	-0.685178	1.486442	-3.773455

n=10 (NH4NO3)n wB97X-D3/6-31G(d)

N	2.866448	0.151974	1.427072
H	3.042034	1.080598	1.836260
H	1.951916	0.202005	0.949045
H	2.832905	-0.554552	2.183229
H	3.553995	-0.130121	0.712683
N	-3.253698	0.274137	-1.103056
H	-4.177343	0.078348	-0.720569
H	-2.545869	-0.074657	-0.422268
H	-3.154130	1.284583	-1.294834
H	-3.102132	-0.194220	-2.017656
N	0.704089	3.846177	-1.411476
H	-0.085884	4.503245	-1.410004
H	1.274940	3.973596	-2.266076

H	0.302647	2.898209	-1.507774
H	1.243866	3.882827	-0.527717
N	2.198491	-4.169781	2.370748
H	2.373258	-3.312916	2.952797
H	1.246552	-4.543592	2.578824
H	2.903402	-4.880349	2.552376
H	2.265152	-3.874921	1.386576
N	-0.560404	1.765066	4.305664
H	-0.066243	2.467103	3.714729
H	-1.546439	1.678682	3.981372
H	-0.536090	2.067453	5.277388
H	-0.057403	0.859574	4.217494
N	0.279014	-3.162952	-1.081257
H	0.260382	-2.535257	-0.260730
H	-0.344664	-2.749958	-1.796626
H	-0.045829	-4.086415	-0.758418
H	1.252537	-3.213099	-1.421989
N	-1.950736	-2.555311	3.445592
H	-2.209607	-3.265850	4.127866
H	-1.977272	-3.014818	2.510639
H	-0.977704	-2.268371	3.658893
H	-2.584562	-1.730716	3.509993
N	2.343396	-0.825298	-3.773173
H	2.702329	-0.915710	-4.721024
H	2.543569	0.138291	-3.432985
H	2.749436	-1.530901	-3.125687
H	1.314635	-0.925852	-3.789761
N	-1.566537	2.576339	0.868150
H	-0.885919	3.162054	1.387793
H	-1.988633	3.147763	0.111998
H	-2.273790	2.212468	1.542906
H	-0.995719	1.808996	0.482904
N	-1.269159	1.957158	-5.022085
H	-1.691778	2.103212	-5.936348
H	-1.445901	2.788948	-4.421281
H	-0.241037	1.816892	-5.127902
H	-1.689372	1.112176	-4.572262
N	-3.392833	0.536494	2.910285
O	-3.260491	-0.177720	3.923552
O	-3.819811	0.093723	1.846741
O	-3.055936	1.757845	3.005495
N	-0.736503	-4.746174	1.487999
O	0.019422	-5.299917	0.665779
O	-0.471045	-4.813249	2.715099
O	-1.728419	-4.097178	1.101937
N	1.621059	3.195216	2.112024
O	2.217638	2.472722	2.920250
O	0.471748	3.640419	2.426456
O	2.125401	3.482715	1.016886
N	0.428180	0.590267	-1.214040
O	0.949576	-0.474707	-1.524688
O	0.664037	1.109785	-0.088450
O	-0.351166	1.198421	-1.987955
N	-2.257298	3.723349	-2.309662
O	-1.628202	4.139817	-3.298446
O	-3.021201	2.747338	-2.405461
O	-2.089460	4.300585	-1.203098
N	1.555610	-1.376465	4.077325
O	2.642465	-1.764307	3.554946
O	0.707852	-2.220850	4.404886
O	1.393777	-0.164016	4.253090
N	1.774032	2.312217	-4.047839
O	1.323597	3.471286	-4.054273
O	1.481696	1.524897	-4.982398
O	2.497031	1.917570	-3.107388
N	-0.586996	-0.924166	1.314599
O	-1.297869	-1.039854	0.292796
O	0.366889	-1.719786	1.469174
O	-0.819485	-0.048116	2.160831
N	3.349387	-2.106382	-0.826781
O	3.953886	-1.036197	-0.972660
O	2.978963	-2.758729	-1.846082
O	3.090079	-2.543861	0.308451
N	-1.500447	-1.065238	-3.566697
O	-2.431584	-0.229907	-3.754898
O	-0.501151	-1.039817	-4.296809
O	-1.643563	-1.890289	-2.651003

n=11 (NH4NO3)n wB97X-D3/6-31G(d)

N	0.966058	-0.634812	-2.727708
H	0.698010	0.277696	-3.122408
H	0.504008	-0.734732	-1.805074
H	1.996196	-0.667597	-2.591347
H	0.616300	-1.393402	-3.328114
N	-4.600651	-3.206135	-0.688012
H	-3.808819	-3.115175	-1.349236
H	-4.273673	-3.724637	0.154163
H	-5.360733	-3.707642	-1.142701
H	-4.928900	-2.254619	-0.412239
N	0.055952	-1.216732	2.360373
H	0.098550	-1.199199	1.329019
H	-0.631249	-1.930131	2.631426
H	-0.226715	-0.291364	2.719572
H	1.004909	-1.429872	2.729717
N	-2.881625	-0.161723	-3.999279
H	-3.498620	-0.169475	-3.163606
H	-3.449596	-0.162329	-4.844049
H	-2.268727	-1.006329	-3.976983
H	-2.293000	0.694341	-3.965094
N	-1.999771	2.682177	-0.808930
H	-1.940087	3.131265	0.113947
H	-1.214812	2.013264	-0.829231
H	-1.881089	3.353885	-1.577130
H	-2.873847	2.139284	-0.898880
N	4.234919	-1.759261	0.810681
H	4.256954	-2.792979	0.961061
H	3.252841	-1.586430	0.567255
H	4.834446	-1.415645	0.051000
H	4.435742	-1.251047	1.685192
N	-4.034967	-0.140269	2.376571
H	-3.930341	-1.146819	2.629539
H	-4.473322	0.380704	3.132155
H	-4.633786	-0.127616	1.518934
H	-3.115124	0.274108	2.131313
N	2.026244	3.724373	-1.779389
H	1.668527	3.354736	-0.882448
H	1.220852	4.038360	-2.348287
H	2.496904	2.949084	-2.254845
H	2.706889	4.458208	-1.521061
N	4.507055	2.176643	1.041734
H	3.493996	2.213013	0.826427
H	4.582501	1.615514	1.907763
H	4.814222	3.146269	1.175631
H	4.976514	1.687689	0.257816
N	1.568521	3.485465	3.220385
H	2.307138	4.103277	2.806851
H	1.905508	2.504732	3.233732
H	0.707954	3.524018	2.648222
H	1.345771	3.766641	4.172793
N	-0.745453	-4.323849	0.090684
H	-1.571987	-4.549860	0.667570
H	0.124300	-4.778107	0.462541
H	-0.591189	-3.303650	0.109192
H	-0.901881	-4.558964	-0.898219
N	-3.149586	-3.485520	2.218930
O	-2.078583	-3.219417	2.768960
O	-4.188785	-2.834655	2.445412
O	-3.219796	-4.441839	1.395415
N	-4.891590	0.077283	-0.856919
O	-4.831824	-0.407660	-1.996433
O	-4.469479	1.204202	-0.577883
O	-5.429441	-0.618722	0.057728
N	3.220978	4.994204	0.826415
O	3.698276	4.691306	1.961772
O	2.043994	5.348514	0.723108
O	3.961051	4.874885	-0.175108
N	1.385515	1.228923	0.166386
O	1.730837	2.344501	0.661625
O	0.504159	1.218974	-0.708189
O	1.938019	0.192375	0.544058
N	3.047596	-0.123269	3.243549
O	2.722315	-1.323702	3.233002
O	4.235382	0.178600	2.950475
O	2.215960	0.751878	3.531862
N	-1.672729	-1.038358	-0.556322
O	-0.506311	-1.536819	-0.462221
O	-1.892648	-0.193409	-1.427739
O	-2.551141	-1.423985	0.229725
N	-0.597946	2.741452	-3.469645
O	0.405776	2.082821	-3.779276
O	-0.480554	3.879500	-2.948620
O	-1.753855	2.299543	-3.637991
N	4.123501	0.277558	-1.593789
O	5.173675	0.279113	-0.899906
O	3.703983	-0.804858	-2.044542
O	3.514343	1.340704	-1.793568
N	-1.135392	1.962490	2.207907
O	-1.808516	1.204242	1.465570
O	-0.986826	3.160744	1.878002
O	-0.616046	1.521846	3.244075
N	-1.500633	-3.067073	-3.114387
O	-2.681779	-2.900716	-2.736438
O	-1.027951	-2.245108	-3.948417
O	-0.804344	-3.999551	-2.711065
N	2.345736	-4.284345	1.021354

O	3.580247	-4.387277	1.156258
O	1.640429	-5.314110	1.027658
O	1.806167	-3.159608	0.870221

n=12 (NH4NO3)n wB97X-D3/6-31G(d)

N	-0.992168	1.826385	2.016685
H	-0.653574	1.470030	2.929897
H	-0.621397	2.767633	1.795000
H	-2.023855	1.820518	2.019915
H	-0.666777	1.160269	1.304025
N	0.710023	5.574915	-3.082647
H	-0.046409	4.907744	-3.343944
H	1.629645	5.189971	-3.407172
H	0.741920	5.690340	-2.048724
H	0.543708	6.479769	-3.517725
N	-4.963548	-0.667059	4.048272
H	-3.923041	-0.682893	4.072485
H	-5.310452	-1.651675	4.029832
H	-5.210504	-0.201213	3.150197
H	-5.329119	-0.145974	4.841805
N	-1.612007	-3.903214	1.933111
H	-1.513411	-3.067971	2.529005
H	-2.517327	-4.365661	2.159582
H	-0.804626	-4.527455	2.065650
H	-1.609556	-3.632907	0.942155
N	-0.228242	-1.146428	-3.006336
H	0.560054	-1.494242	-3.574333
H	-1.067826	-1.733891	-3.127599
H	-0.426302	-0.162413	-3.258798
H	0.055996	-1.194256	-2.018568
N	-3.537696	-1.680681	-0.157193
H	-4.060633	-0.814943	-0.359756
H	-3.366210	-2.230165	-1.020440
H	-4.016751	-2.227294	0.574972
H	-2.611293	-1.415218	0.235009
N	2.116990	-1.319127	3.879985
H	2.447182	-1.548957	4.814678
H	1.220995	-0.782734	3.947360
H	1.932575	-2.191310	3.351847
H	2.830512	-0.753695	3.369328
N	3.335462	-1.611993	-0.035360
H	2.429319	-1.133570	-0.127809
H	3.783152	-1.663274	-0.964856
H	3.146498	-2.542578	0.360752
H	3.890136	-1.063827	0.642652
N	3.021403	1.401863	-3.573834
H	2.211056	1.859888	-4.014844
H	3.785498	2.087927	-3.458920
H	3.275376	0.548748	-4.083549
H	2.719545	1.103867	-2.627781
N	1.355533	-5.052029	-1.428188
H	1.753536	-5.871593	-1.881861
H	1.984134	-4.248492	-1.561211
H	1.213232	-5.232733	-0.410083
H	0.442442	-4.796282	-1.879784
N	-2.268492	2.813726	-1.068231
H	-2.066557	3.664419	-0.517640
H	-2.294957	3.071132	-2.067162
H	-3.120874	2.319350	-0.744093
H	-1.468364	2.160057	-0.960173
N	3.139742	3.934598	0.578834
H	3.325207	4.488058	-0.283842
H	2.141311	4.024456	0.836429
H	3.707131	4.278925	1.350512
H	3.368547	2.928561	0.434350
N	0.846188	1.271502	-1.098263
O	1.101660	2.464183	-1.276178
O	-0.274076	0.909368	-0.651497
O	1.677508	0.378993	-1.387054
N	0.021895	5.006597	0.264691
O	0.326881	4.203741	1.186102
O	0.930308	5.665765	-0.271892
O	-1.156857	5.141503	-0.098161
N	-4.585383	-3.623257	3.006805
O	-5.453665	-3.415395	3.872646
O	-4.130744	-2.647578	2.345454
O	-4.143493	-4.765409	2.804066
N	1.601083	-4.345578	1.710810
O	1.242232	-3.690164	2.702737
O	0.831361	-5.245685	1.280829
O	2.684724	-4.144791	1.137828
N	-0.029447	-1.346048	0.804081
O	0.086856	-2.199482	-0.102253
O	0.966006	-0.813525	1.311604
O	-1.173385	-1.020394	1.212067
N	-1.754059	-3.729927	-2.041041
O	-1.979848	-4.282070	-0.959140
O	-2.491298	-2.792930	-2.451409
O	-0.789562	-4.066513	-2.759951
N	3.667849	4.356766	-2.657217
O	3.096699	4.338682	-3.775356
O	3.408800	5.279179	-1.853434
O	4.459978	3.449925	-2.348892
N	-1.212610	-0.600552	4.022644
O	-1.082007	-1.825835	4.089509
O	-2.316702	-0.050261	3.861382
O	-0.199040	0.151123	4.120883
N	-0.589535	2.593023	-3.801138
O	-1.077700	1.497978	-3.460902
O	0.511876	2.687749	-4.351871
O	-1.258906	3.638567	-3.562070
N	2.880697	-1.813367	-3.179416
O	3.995200	-1.426551	-2.781731
O	2.249708	-2.698467	-2.564114
O	2.358943	-1.284324	-4.187847
N	-4.506052	1.055152	1.051417
O	-5.351952	0.239242	1.457585
O	-3.936323	1.841257	1.823701
O	-4.228541	1.053444	-0.180987
N	3.563070	0.964921	1.788745
O	2.900035	1.716375	2.503677
O	3.848967	1.255538	0.606355
O	3.976767	-0.147873	2.234325

n=13 (NH4NO3)n wB97X-D3/6-31G(d)

N	1.348749	-4.696425	-2.667398
H	1.674211	-5.635762	-2.887080
H	2.149210	-4.029602	-2.715551
H	0.924422	-4.696184	-1.714315
H	0.643049	-4.404760	-3.374961
N	4.050701	2.770019	1.258029
H	3.554294	1.963165	0.836632
H	3.924671	3.588397	0.616740
H	5.026112	2.529500	1.416866
H	3.582364	3.022325	2.138266
N	-2.282898	-3.584366	-0.348243
H	-1.597648	-4.329214	-0.121079
H	-1.760825	-2.692652	-0.457366
H	-3.008190	-3.452476	0.366980
H	-2.726094	-3.778783	-1.248573
N	-3.353359	-0.935341	-3.227861
H	-3.082406	-1.685229	-3.896130
H	-2.595088	-0.898935	-2.519517
H	-4.222755	-1.180086	-2.735899
H	-3.357717	-0.047560	-3.753982
N	2.928347	-1.694169	-0.227219
H	2.618527	-0.717855	-0.316940
H	3.338804	-2.012869	-1.121936
H	2.110542	-2.282186	-0.032884
H	3.580272	-1.787462	0.572081
N	1.243777	-2.759488	3.389442
H	2.223421	-2.779353	3.689115
H	1.191015	-2.127897	2.569518
H	0.643612	-2.345462	4.119057
H	0.891673	-3.677887	3.062026
N	-1.371378	3.203344	4.919678
H	-0.371441	3.512123	4.944727
H	-1.868615	3.570249	5.728678
H	-1.430504	2.166172	4.916522
H	-1.804596	3.580764	4.039517
N	1.596905	1.691877	-3.161545
H	0.983286	1.950497	-3.950104
H	2.280697	2.429415	-2.913169
H	2.076923	0.800359	-3.357175
H	0.987203	1.548691	-2.342295
N	0.079903	4.800226	0.684991
H	0.586708	5.032838	-0.187402
H	0.387375	5.358093	1.493324
H	0.326302	3.816569	0.895894
H	-0.942617	4.847995	0.571426
N	0.080780	-1.056090	-5.882849
H	0.836312	-1.349346	-5.228302
H	0.381956	-1.234358	-6.838986
H	-0.134172	-0.044590	-5.750034
H	-0.768695	-1.620181	-5.679192
N	-3.618488	-0.316511	2.589296
H	-4.137330	0.544017	2.757073
H	-3.392564	-0.796297	3.473953
H	-2.708915	-0.050770	2.154844
H	-4.119438	-0.959273	1.944369
N	1.587346	1.089967	3.742921
H	0.815298	0.583712	4.212718
H	2.443727	0.516252	3.888222
H	1.691375	2.044183	4.134326

H	1.389460	1.151211	2.734078
N	-2.515523	1.425990	-0.767832
H	-3.314795	0.785701	-0.673943
H	-1.707924	1.027128	-0.270218
H	-2.732084	2.346551	-0.333990
H	-2.248361	1.562114	-1.756288
N	2.868364	-1.894457	-3.493399
O	2.183730	-2.240595	-4.468481
O	3.084649	-0.709170	-3.213571
O	3.375812	-2.792892	-2.760585
N	-4.675507	-1.669996	-0.298826
O	-4.758766	-2.352187	-1.333135
O	-4.534439	-2.246467	0.810193
O	-4.717969	-0.423484	-0.346398
N	1.451078	4.448591	3.685112
O	0.567886	5.288539	3.475583
O	1.338992	3.680479	4.697366
O	2.423450	4.324417	2.932064
N	-0.317568	-1.321041	-2.017047
O	0.558787	-2.099867	-1.638023
O	-0.257021	-0.737087	-3.107583
O	-1.335395	-1.119914	-1.275585
N	-1.427106	1.532690	-4.492475
O	-2.388046	0.911916	-4.975510
O	-0.358756	1.610233	-5.164822
O	-1.482131	2.071868	-3.378033
N	-2.703539	3.562033	1.738665
O	-3.252903	2.513902	2.088871
O	-2.172894	4.332198	2.570094
O	-2.643765	3.880813	0.520943
N	-0.454400	-0.751989	1.584382
O	0.801515	-0.713419	1.524542
O	-1.046904	-1.830288	1.667820
O	-1.086647	0.334264	1.599298
N	-1.404798	-3.411300	-4.281102
O	-1.775365	-3.682046	-3.131613
O	-2.093610	-2.633558	-5.003990
O	-0.349373	-3.871556	-4.747819
N	0.600177	-4.638855	0.823315
O	1.714840	-4.101508	0.755676
O	0.032369	-5.046750	-0.233604
O	0.014866	-4.792724	1.905836
N	3.844803	-0.982595	2.825978
O	3.415156	-0.935476	4.008384
O	4.047696	0.066579	2.193156
O	4.020476	-2.100109	2.297938
N	-1.400547	-0.649720	4.762630
O	-1.943140	0.478840	4.782394
O	-0.153320	-0.748104	4.903606
O	-2.086733	-1.660923	4.590747
N	1.252155	1.646340	0.078370
O	2.333471	1.120630	-0.264049
O	0.262551	1.582150	-0.660928
O	1.198283	2.277747	1.159061
N	2.609793	4.227093	-1.282760
O	3.411926	3.472403	-1.865432
O	2.937255	4.776759	-0.202421
O	1.465657	4.417567	-1.745882

n=14 $(NH_4NO_3)_n$ wB97X-D3/6-31G(d)

N	0.424303	-2.683816	-0.167731
H	1.352212	-3.115801	-0.293511
H	-0.116513	-3.200307	0.539210
H	-0.096465	-2.630928	-1.057722
H	0.538769	-1.728717	0.196389
N	1.806481	4.020006	-0.444824
H	1.725540	3.048078	-0.108421
H	2.812917	4.260129	-0.510061
H	1.328624	4.610797	0.260578
H	1.360296	4.088226	-1.375662
N	2.549729	-2.761105	-3.180803
H	2.946214	-3.494090	-2.587118
H	1.609693	-2.992254	-3.555654
H	3.176900	-2.500447	-3.965878
H	2.459654	-1.923452	-2.583500
N	-3.691907	-3.620588	2.252784
H	-3.715021	-3.489151	1.220074
H	-2.795372	-4.022681	2.576940
H	-4.479443	-4.212042	2.510628
H	-3.829144	-2.694448	2.692751
N	-3.542750	-0.779328	-2.412519
H	-3.025137	-1.123853	-3.238627
H	-2.880131	-0.831164	-1.618983
H	-4.371307	-1.356574	-2.181010
H	-3.833332	0.204318	-2.509960
N	-2.966649	2.272142	2.186080
H	-3.698314	2.054130	2.880510
H	-3.403029	2.750104	1.388381
H	-2.649971	1.358158	1.840417
H	-2.165946	2.805735	2.571914
N	2.693558	3.350560	3.263964
H	3.300081	3.146893	2.458372
H	1.921007	3.978310	2.945361
H	3.241071	3.800933	3.994646
H	2.295233	2.452871	3.624312
N	4.182868	0.016625	0.703077
H	4.851433	0.726756	0.358800
H	3.817664	0.286189	1.626049
H	4.615340	-0.919124	0.756276
H	3.394175	-0.033298	0.036806
N	2.815745	-3.350396	2.880731
H	1.856056	-3.644392	2.600857
H	2.741136	-2.444176	3.386929
H	3.441240	-3.258054	2.050117
H	3.178083	-4.047538	3.528744
N	3.832423	2.489281	-3.189075
H	3.081610	2.987384	-3.693860
H	4.422848	3.124076	-2.634938
H	4.361171	1.885253	-3.853642
H	3.339056	1.857356	-2.544876
N	-1.208681	2.061053	-1.166770
H	-0.864090	1.225684	-1.657068
H	-2.246455	2.090026	-1.220345
H	-0.823751	2.890020	-1.648812
H	-0.899548	2.030905	-0.186286
N	0.379413	0.180114	-5.365912
H	0.059728	1.077098	-4.936331
H	0.506873	0.301369	-6.368556
H	-0.338547	-0.545698	-5.178016
H	1.286966	-0.088049	-4.934759
N	-0.569117	-0.866815	3.924200
H	0.000309	-0.097224	4.305946
H	-0.372799	-0.923138	2.915488
H	-1.585087	-0.708991	4.045047
H	-0.261870	-1.752623	4.353988
N	-6.633739	-0.150108	1.210622
H	-6.074867	0.182933	2.025429
H	-6.329075	0.344126	0.340875
H	-7.620866	0.034948	1.374975
H	-6.483372	-1.174367	1.082885
N	-0.288391	4.193432	1.977593
O	0.795354	4.856120	1.963177
O	-0.925146	4.058833	0.924847
O	-0.668578	3.696422	3.048590
N	2.495206	0.093594	4.049245
O	1.627621	0.983148	4.279570
O	3.571797	0.405554	3.520986
O	2.238399	-1.077696	4.374000
N	1.433519	0.212345	-1.791767
O	2.226757	-0.627232	-1.292689
O	0.419229	-0.176827	-2.385130
O	1.674696	1.430308	-1.674378
N	4.096075	-3.003828	-0.365797
O	4.501449	-2.759541	0.808919
O	4.648461	-2.470901	-1.320311
O	3.121687	-3.786801	-0.520610
N	-0.311072	-4.043009	3.414188
O	-1.467854	-4.410892	3.646212
O	0.187781	-4.177241	2.260987
O	0.384625	-3.536614	4.317448
N	-4.824576	1.778512	-0.891861
O	-5.717595	0.956712	-1.170899
O	-4.846679	2.398310	0.179712
O	-3.900930	1.962012	-1.728495
N	-2.200917	-0.958073	0.709424
O	-1.587908	-0.811151	-0.372167
O	-3.230224	-0.289663	0.930808
O	-1.775328	-1.771985	1.551277
N	-5.261992	-2.624356	-0.313203
O	-4.103132	-3.081144	-0.447265
O	-5.872649	-2.789832	0.773068
O	-5.800003	-1.995503	-1.236236
N	0.856881	0.775461	1.403583
O	1.749161	1.623734	1.207995
O	-0.339164	1.113427	1.450069
O	1.175526	-0.427174	1.568633
N	3.960945	-0.330327	-4.744407
O	4.089296	-1.486221	-5.173813
O	2.886469	0.009322	-4.158268
O	4.869346	0.504750	-4.870963
N	-0.873998	-2.357209	-3.813709
O	0.148936	-2.694538	-4.444200
O	-1.132890	-2.805240	-2.692507
O	-1.650299	-1.529276	-4.365859
N	-4.300371	-0.163208	3.653144

O	-3.175396	0.097651	4.116115
O	-5.046266	0.801090	3.326790
O	-4.709843	-1.321496	3.511750
N	0.357774	3.124692	-3.742710
O	1.383291	3.076447	-4.437986
O	-0.614143	2.388653	-4.003903
O	0.268104	3.915669	-2.769246
N	4.837581	3.054399	-0.185488
O	4.490159	3.021470	1.006256
O	5.453830	2.100940	-0.694441
O	4.541265	4.046120	-0.906002

n=15 (NH4NO3)n wB97X-D3/6-31G(d)

N	0.527280	-2.562258	-1.186479
H	0.670585	-2.854154	-0.212987
H	-0.358518	-2.946634	-1.562566
H	1.357100	-2.889745	-1.716955
H	0.513611	-1.535752	-1.224158
N	2.843845	-1.245292	-4.054251
H	3.435268	-0.579447	-4.586434
H	2.442612	-0.731786	-3.242888
H	3.345211	-2.055045	-3.674498
H	2.046595	-1.545037	-4.630960
N	4.699161	-3.770692	1.880095
H	5.398532	-4.353775	2.334235
H	4.759871	-2.792236	2.230706
H	3.746492	-4.115237	2.128351
H	4.816301	-3.774104	0.840886
N	-5.284021	-2.095897	-0.560340
H	-5.498595	-2.526992	0.354439
H	-4.351377	-1.661974	-0.455757
H	-5.931871	-1.332869	-0.796408
H	-5.196315	-2.789620	-1.316839
N	1.099204	-2.994419	5.458376
H	0.415473	-2.282807	5.801413
H	1.973312	-2.525350	5.143878
H	0.706978	-3.535875	4.662584
H	1.334562	-3.632361	6.215904
N	-1.813111	1.954942	-3.082767
H	-2.834187	1.817897	-2.991246
H	-1.383416	1.257136	-3.704446
H	-1.432139	1.844162	-2.134423
H	-1.569386	2.892034	-3.448370
N	-4.379399	1.676691	1.062690
H	-3.433904	1.583628	0.653834
H	-4.614563	0.807210	1.578355
H	-5.053742	1.763700	0.290009
H	-4.378975	2.476378	1.710813
N	-2.812647	-1.496972	-4.185509
H	-2.147562	-0.861490	-4.668059
H	-3.445175	-0.961614	-3.564683
H	-2.284009	-2.190189	-3.620038
H	-3.382579	-2.012240	-4.853625
N	0.070343	3.586046	0.597731
H	0.878156	4.040851	1.050396
H	-0.823102	3.839861	1.064116
H	0.186588	2.567590	0.657999
H	0.057950	3.865362	-0.397043
N	-2.570984	0.671917	4.625336
H	-2.967526	1.596535	4.365185
H	-3.267494	-0.073272	4.512831
H	-2.127155	0.700686	5.557249
H	-1.815909	0.449333	3.958002
N	1.418765	2.903562	-5.892735
H	1.190730	1.887501	-5.902424
H	2.384444	3.028109	-5.517840
H	0.748681	3.414413	-5.274797
H	1.367505	3.270068	-6.840772
N	1.256376	1.888998	4.403107
H	0.693870	1.687628	3.555149
H	1.774269	2.748102	4.210549
H	1.908450	1.101025	4.506339
H	0.652190	1.935949	5.242133
N	3.034572	0.420532	0.727726
H	3.627688	1.259724	0.636511
H	3.495472	-0.257183	1.358567
H	2.808307	0.016853	-0.192558
H	2.146359	0.717677	1.154376
N	-1.810850	-2.944991	1.665141
H	-2.239472	-3.352949	0.822307
H	-1.183100	-3.617750	2.142537
H	-2.535301	-2.607875	2.320324
H	-1.233393	-2.135236	1.397584
N	3.716539	3.834351	-1.867124
H	2.727603	4.053438	-2.075147
H	3.881117	3.749093	-0.844453
H	4.297398	4.561902	-2.278672
H	3.948441	2.934122	-2.331897
N	4.007438	1.795162	-4.386231
O	3.860648	3.025292	-4.587529
O	4.240437	1.384682	-3.226832
O	3.901873	0.996736	-5.332218
N	3.428012	-1.320408	3.585345
O	3.605322	-2.202577	4.439963
O	4.301939	-1.158892	2.686765
O	2.405113	-0.616799	3.581714
N	0.017238	-0.061905	6.417973
O	-0.505173	1.088735	6.425033
O	1.241923	-0.192622	6.480527
O	-0.737435	-1.058883	6.314031
N	-2.145399	-0.033056	-0.623507
O	-1.694523	-0.549162	-1.654109
O	-2.924485	-0.664148	0.128120
O	-1.837670	1.145341	-0.313639
N	1.461897	1.183717	-2.071179
O	1.586695	2.054070	-1.197679
O	1.052403	1.455642	-3.206431
O	1.776130	-0.014992	-1.792212
N	-2.959136	-3.652557	-1.914807
O	-3.983588	-3.504139	-2.609531
O	-3.052962	-3.945981	-0.714091
O	-1.832697	-3.491649	-2.456578
N	3.678445	-3.166433	-1.099969
O	3.068303	-3.305803	-2.195262
O	4.721632	-3.815184	-0.898484
O	3.228282	-2.399859	-0.230507
N	-0.045808	-0.109574	1.938253
O	-0.627756	-0.503843	2.959252
O	0.435735	1.052528	1.895337
O	0.086740	-0.859618	0.958434
N	-5.073128	0.679587	-1.958610
O	-4.892636	-0.286534	-2.729598
O	-4.375813	1.709575	-2.033642
O	-5.958795	0.605118	-1.079143
N	0.206891	4.375767	-3.135909
O	-0.415337	4.352987	-2.063526
O	-0.428711	4.101723	-4.197615
O	1.407539	4.658808	-3.209292
N	0.068317	-0.342440	-5.359909
O	-0.996119	0.338808	-5.317667
O	1.074122	0.120320	-5.919343
O	0.095936	-1.475512	-4.852960
N	1.187295	-4.309108	2.428126
O	1.124122	-4.193175	1.201669
O	0.135497	-4.263089	3.130158
O	2.271363	-4.476482	3.019315
N	-4.682073	-1.669207	2.462207
O	-4.884567	-2.831280	2.064717
O	-3.706226	-1.448631	3.219621
O	-5.416925	-0.736112	2.084255
N	-2.392492	3.348644	2.840601
O	-2.454952	3.918427	1.730878
O	-3.474154	2.968559	3.373906
O	-1.310029	3.130690	3.397365
N	3.218223	3.509354	1.418074
O	2.672248	4.561445	1.038020
O	2.813226	2.890274	2.411894
O	4.183575	3.043778	0.748835

n=16 (NH4NO3)n wB97X-D3/6-31G(d)

N	4.305975	0.122946	-1.364544
H	4.292673	1.048553	-1.835170
H	4.771129	-0.586160	-1.944338
H	3.335445	-0.211341	-1.210053
H	4.779379	0.132135	-0.449241
N	5.097679	-3.825667	0.896461
H	4.225150	-4.387592	1.010360
H	5.116871	-3.057022	1.603188
H	5.133204	-3.418059	-0.064604
H	5.905780	-4.429253	1.034874
N	-0.813572	-3.098160	3.211445
H	-1.728643	-3.502400	3.447642
H	-0.135540	-3.827554	2.917284
H	-0.502562	-2.552221	4.027863
H	-0.974206	-2.430896	2.432873
N	-2.500433	2.042285	-3.185278
H	-2.237346	1.408523	-2.406551
H	-1.940089	1.771605	-4.005798
H	-2.365618	3.031161	-2.898792
H	-3.482815	1.841308	-3.410551
N	0.996272	2.496140	-3.654262
H	0.281224	3.227499	-3.534362
H	1.886738	2.972871	-3.844899
H	0.763793	1.805665	-4.394200

H	1.105169	2.014127	-2.741442
N	-3.455990	-2.832824	-0.620234
H	-3.445847	-3.680692	-1.199901
H	-4.125169	-2.177784	-1.049477
H	-2.512388	-2.408092	-0.698304
H	-3.667438	-3.051048	0.372674
N	-1.718805	4.076630	0.959511
H	-1.918334	4.834967	0.294924
H	-1.343866	3.283794	0.404567
H	-2.571669	3.779393	1.472262
H	-0.962680	4.405616	1.576017
N	1.097606	-2.188170	-3.800048
H	0.935813	-1.866092	-4.762888
H	0.508987	-3.020581	-3.656470
H	0.739723	-1.443416	-3.172054
H	2.105270	-2.357140	-3.613723
N	0.955241	-4.329618	-0.954782
H	0.078636	-4.585499	-1.449498
H	0.810552	-3.462516	-0.403323
H	1.750482	-4.163536	-1.587459
H	1.244818	-5.058104	-0.289653
N	1.931073	6.063391	-0.951548
H	2.210798	5.753207	0.005863
H	0.892597	6.015837	-1.048469
H	2.380634	5.447409	-1.664812
H	2.237784	7.023343	-1.096131
N	2.307889	-1.434024	3.619194
H	2.099526	-0.981041	2.708794
H	1.730853	-1.004885	4.368393
H	3.313244	-1.305579	3.790961
H	2.147817	-2.445089	3.505791
N	-4.458107	0.312312	0.696967
H	-4.769946	0.362751	-0.292341
H	-3.440641	0.508405	0.759148
H	-4.623442	-0.605052	1.135490
H	-4.929378	1.017371	1.277119
N	-3.757792	-0.222413	5.215906
H	-3.719899	-1.172830	4.785039
H	-4.154152	0.458329	4.529963
H	-2.797219	0.076567	5.495550
H	-4.353238	-0.251741	6.041163
N	-0.321461	2.433654	3.794235
H	-0.191965	1.615232	3.169288
H	-0.250540	2.084397	4.758360
H	-1.285797	2.770612	3.664313
H	0.399331	3.153355	3.591247
N	-3.266589	-2.012990	-5.143852
H	-3.785013	-2.337797	-5.957627
H	-3.184164	-2.793657	-4.454660
H	-3.777090	-1.212893	-4.707969
H	-2.315162	-1.704016	-5.443262
N	3.605771	2.400609	1.282117
H	2.617521	2.110501	1.156849
H	4.141174	1.644934	1.751557
H	3.591089	3.259290	1.845602
H	3.974161	2.650845	0.352978
N	-1.907260	-4.290618	-2.993496
O	-1.602027	-4.832776	-1.913247
O	-1.055826	-4.029733	-3.858234
O	-3.113538	-4.001210	-3.192983
N	-1.156141	5.086622	-2.010383
O	-0.854129	5.863460	-1.068815
O	-2.274007	4.538090	-1.987442
O	-0.342894	4.880710	-2.925224
N	-4.826261	-0.103153	-2.796883
O	-4.507916	0.200205	-3.975036
O	-4.945435	-1.296524	-2.477623
O	-5.006350	0.818493	-1.978774
N	3.703331	3.490536	-2.299535
O	3.929922	2.415860	-2.887001
O	3.000066	4.352726	-2.885402
O	4.144204	3.722821	-1.162802
N	0.695866	2.172702	-0.343745
O	-0.386565	2.442199	-0.904779
O	0.817639	2.331625	0.888366
O	1.665214	1.768997	-1.019560
N	1.740971	4.807079	2.215928
O	1.880346	4.004966	3.160186
O	2.754217	5.106336	1.535985
O	0.637657	5.301978	1.935024
N	5.072706	-0.689916	2.213513
O	5.546403	-0.638162	1.067559
O	4.652696	0.325392	2.799759
O	5.001660	-1.794241	2.811075
N	-0.503623	-0.063474	-5.560591
O	-1.494041	0.677219	-5.455076
O	0.655767	0.360480	-5.396329
O	-0.659649	-1.280940	-5.834059
N	4.177337	-2.929559	-2.268591
O	3.567876	-3.971756	-1.979930
O	5.183170	-2.584482	-1.600725
O	3.814940	-2.196515	-3.210410
N	2.029366	-4.808322	2.007465
O	2.584568	-4.163377	2.911029
O	2.713718	-5.274413	1.060822
O	0.800148	-5.007425	2.000683
N	-3.957181	-2.692586	2.882688
O	-4.751916	-1.791219	2.572027
O	-3.579822	-3.550083	2.061633
O	-3.504614	-2.751079	4.054369
N	-1.181321	-0.717938	-1.850902
O	-0.390971	-0.117776	-2.608882
O	-0.960260	-1.904206	-1.530959
O	-2.206198	-0.139439	-1.433375
N	-0.368791	-0.214148	5.568330
O	-1.195151	0.689411	5.854869
O	0.818995	0.110400	5.381995
O	-0.750221	-1.391272	5.471998
N	-1.353087	-0.092234	1.866324
O	-1.856616	-1.159274	1.457637
O	-1.867958	1.000889	1.553097
O	-0.350119	-0.120182	2.609595
N	1.830132	-1.367525	0.317839
O	2.004817	-1.435239	-0.916426
O	1.122324	-2.218144	0.896431
O	2.384982	-0.464284	0.977621
N	-4.001508	2.406877	3.051996
O	-4.081750	3.016754	1.967406
O	-4.817717	1.477560	3.272669
O	-3.140440	2.690714	3.900037
