# Supplementary material for: Decomposition and Growth Pathways for Ammonium Nitrate Clusters and Nanoparticles
Source: J Phys Chem A. 2024 Oct 14;128(42):9184–94. doi: 10.1021/acs.jpca.4c04630 (PMC11514028; doi:10.1021/acs.jpca.4c04630)
Supplement: Supplementary file 2 — jp4c04630_si_002.zip [file jp4c04630_si_002.zip › SI_ammoniumnitrate particle structures_PDF_XYZ/HassanAmatTopper_SuppMats_S14.pdf]

Supporting Information for:

Decomposition and Growth Pathways for Ammonium Nitrate Clusters and Nanoparticles

Ubaidullah S. Hassan, Miguel A. Amat, and Robert Q. Topper*

Author Affiliations:

Ubaidullah S. Hassan, Department of Chemistry, The Cooper Union for the Advancement of Science and Art, New York NY 10003, United States.

Miguel A. Amat, Department of Chemistry, The Cooper Union for the Advancement of Science and Art, New York NY 10003, United States.

Robert Q. Topper, Department of Chemistry, The Cooper Union for the Advancement of Science and Art, New York NY 10003, United States. Email: topper@cooper.edu; Phone: 212-353-4370.

Table S14: Cartesian Coordinates of n=(1-10) Neutral Clusters: ωB97X-D3/def2-SVPD

n=1 (NH4NO3)n wB97X-D3/def2-SVPD

N	0.009321	0.000216	-1.514856
H	-0.617356	0.000206	-0.003107
H	1.008190	0.001037	-1.318359
H	-0.200510	0.821262	-2.076000
H	-0.199283	-0.821792	-2.075048
N	0.051429	-0.000273	1.788964
O	-0.217228	-0.000050	2.954261
O	1.158759	-0.000833	1.293280
O	-0.993323	0.000228	0.950865

n=2 (NH4NO3)n wB97X-D3/def2-SVPD

N	-0.004455	2.397885	-0.269103
H	-0.002747	1.899902	-1.162527
H	-0.007314	3.405360	-0.403836
H	0.858349	2.071097	0.242998
H	-0.865654	2.066294	0.242649
N	0.004770	-2.397787	-0.269127
H	0.007020	-3.405274	-0.403739
H	0.002696	-1.899905	-1.162602
H	0.866334	-2.066475	0.242212
H	-0.857614	-2.070563	0.243352
N	1.871010	0.004115	0.334156
O	2.031280	1.081854	0.935642
O	2.034961	-1.073204	0.935403
O	1.516539	0.003612	-0.854675
N	-1.871372	-0.004247	0.333799
O	-2.031218	-1.081948	0.935475
O	-1.517722	-0.003785	-0.855262
O	-2.034861	1.073069	0.935185

n=3 (NH4NO3)n wB97X-D3/def2-SVPD

N	-2.369822	-0.068964	0.478040
H	-2.340392	-0.361207	-0.547364
H	-3.320594	-0.070896	0.835171
H	-1.965978	0.879966	0.497723
H	-1.740938	-0.687178	1.024620
N	0.921794	-0.198029	-2.213388
H	1.697813	-0.488326	-2.801938
H	0.492287	-1.021512	-1.759724
H	0.171620	0.290801	-2.776995
H	1.259198	0.426122	-1.453091
N	2.250295	-0.084669	2.724612
H	1.566362	0.686750	2.475244
H	3.198984	0.279483	2.675725
H	2.117272	-0.858796	2.011586
H	2.051275	-0.445857	3.654602
N	0.563596	1.563776	0.621429
O	-0.392680	1.772368	-0.123546
O	1.648500	1.157033	0.155517
O	0.457509	1.729096	1.853687
N	-2.055047	0.692134	-2.576412
O	-1.938016	-0.472410	-2.093241
O	-1.175665	1.081407	-3.368942
O	-2.988462	1.401332	-2.245106
N	0.469042	-1.808928	0.731853
O	-0.109561	-2.085295	-0.318578
O	1.708268	-1.923689	0.825439
O	-0.176661	-1.384510	1.713076

n=4 (NH4NO3)n wB97X-D3/def2-SVPD

N	1.162833	1.071312	-2.518649
H	1.560850	1.437251	-3.379619
H	0.863162	1.858469	-1.902095
H	1.873065	0.496256	-2.014200
H	0.334187	0.474764	-2.735169
N	1.074222	-2.725918	0.506003
H	0.154994	-2.610652	0.986769
H	1.739928	-2.008527	0.868951
H	0.942092	-2.578631	-0.518960
H	1.441358	-3.658251	0.677937
N	-2.960500	-0.132600	-0.250257
H	-2.595856	0.707825	-0.750671
H	-2.692681	-0.071349	0.756793
H	-2.529137	-0.987928	-0.664885
H	-3.972366	-0.176957	-0.338280
N	0.722833	1.787084	2.264562
H	-0.124585	2.155070	1.778857
H	1.511386	1.739321	1.582219
H	0.521386	0.825840	2.618230
H	0.970877	2.396715	3.039441
N	-0.659117	-1.628464	-2.065509
O	-1.449221	-2.228977	-1.318056
O	-1.043821	-0.638110	-2.709177
O	0.526776	-1.992364	-2.135913
N	2.697869	0.119576	0.229839
O	2.802889	-0.470048	-0.858615
O	2.623414	1.359385	0.259606
O	2.624064	-0.532436	1.284736
N	-1.060703	-0.976913	2.296356
O	0.051725	-0.866624	2.838466
O	-1.358070	-2.023064	1.695970
O	-1.858584	-0.024820	2.317516

N	-0.977743	2.485920	-0.462382
O	-1.628577	2.030892	-1.417914
O	0.181358	2.896142	-0.640034
O	-1.470305	2.490814	0.678138

n=5 (NH4NO3)n wB97X-D3/def2-SVPD

N	0.939318	3.737760	0.503246
H	1.313049	2.989625	1.117951
H	1.676697	4.057606	-0.181843
H	0.595711	4.513516	1.062701
H	0.161597	3.349142	-0.053299
N	0.797665	0.758191	-2.575394
H	0.366337	0.040453	-3.173222
H	1.349481	0.277786	-1.860911
H	0.063455	1.294602	-2.088882
H	1.417281	1.426775	-3.081784
N	-2.811009	-1.983379	-0.966429
H	-2.134426	-2.603877	-1.463458
H	-3.086364	-2.423679	-0.054405
H	-3.620126	-1.796465	-1.552554
H	-2.315739	-1.101983	-0.740761
N	-0.452569	-0.129331	3.413825
H	0.461089	-0.464976	3.061004
H	-0.323059	0.328099	4.312529
H	-1.138672	-0.918987	3.489499
H	-0.805277	0.564421	2.715658
N	1.209147	-2.724624	0.350526
H	1.557769	-1.882429	0.865366
H	0.214002	-2.846949	0.589422
H	1.721188	-3.553190	0.643161
H	1.308993	-2.592440	-0.680546
N	1.875777	0.563468	1.376866
O	1.882201	-0.578812	1.911664
O	1.891977	0.669583	0.153050
O	1.829863	1.556967	2.110055
N	-0.060157	-2.334876	-2.532785
O	1.156907	-2.145243	-2.336739
O	-0.702399	-1.570802	-3.258492
O	-0.628472	-3.280949	-1.952938
N	-2.319987	-2.512356	2.223479
O	-3.326525	-2.820699	1.569178
O	-1.183061	-2.691399	1.751454
O	-2.439322	-2.009266	3.349886
N	2.034886	3.518641	-2.343547
O	0.796563	3.610387	-2.326656
O	2.710473	4.146528	-1.502185
O	2.590950	2.770790	-3.161670
N	-1.149327	1.197794	0.324815
O	-1.138159	1.716470	1.453814
O	-1.069769	1.917851	-0.677982
O	-1.217957	-0.039744	0.217332

n=6 (NH4NO3)n wB97X-D3/def2-SVPD

N	-0.229173	-1.469389	3.715244
H	-0.917629	-2.099485	3.243618
H	0.635981	-1.385382	3.134986
H	0.009096	-1.856994	4.624342
H	-0.650310	-0.525142	3.827469
N	-3.463365	-1.205992	0.468105
H	-3.396857	-0.935643	1.472585
H	-2.976997	-0.478117	-0.087058
H	-4.437553	-1.286730	0.191910
H	-2.957660	-2.111459	0.333988
N	-0.868401	1.368823	-3.565198
H	-0.430396	0.442005	-3.754463
H	-1.515653	1.633094	-4.301951
H	-0.117456	2.087322	-3.461154
H	-1.351382	1.281638	-2.653019
N	-0.182337	2.662393	1.242543
H	0.544049	1.928855	1.285198
H	0.069753	3.383954	1.913550
H	-1.111604	2.266617	1.506389
H	-0.231291	3.077709	0.288532
N	1.245586	-2.590631	-0.372467
H	0.782872	-3.485749	-0.178890
H	1.540160	-2.433437	-1.356771
H	2.050904	-2.477516	0.250684
H	0.578504	-1.850503	-0.095585
N	3.234775	1.381119	-1.585676
H	2.814516	2.308245	-1.371709
H	3.070024	0.742634	-0.777746
H	4.234875	1.474955	-1.739692
H	2.789503	0.991943	-2.445290
N	1.505747	-0.829152	-3.310695
O	0.322207	-1.168121	-3.470287
O	2.292485	-1.556457	-2.688694
O	1.896719	0.264670	-3.758245
N	-0.642539	0.192998	-0.538664
O	0.396910	0.643418	-1.017241
O	-1.749509	0.501153	-1.034548
O	-0.611145	-0.568346	0.445416
N	-2.334961	0.768281	2.811997
O	-1.360626	1.071099	3.524878
O	-2.980201	-0.261685	3.050108
O	-2.634033	1.485648	1.841780
N	0.812565	3.481738	-1.748140
O	1.718037	3.608832	-0.903407
O	1.098831	3.210211	-2.923457
O	-0.373738	3.591473	-1.399130
N	2.308298	-0.308409	1.524864
O	1.889680	0.783136	1.911939
O	3.013292	-0.408880	0.506826
O	2.032064	-1.353506	2.147970
N	-1.337153	-3.507409	1.320442
O	-0.220780	-3.996228	1.508459
O	-1.810561	-3.437263	0.169490
O	-1.994122	-3.046337	2.275866

n=7 (NH4NO3)n wB97X-D3/def2-SVPD

N	-2.701341	-2.461405	0.952728
H	-2.358936	-2.477032	1.942549
H	-3.448697	-3.140254	0.836216
H	-1.931933	-2.703267	0.316833
H	-3.024904	-1.504895	0.681911
N	-1.132137	3.943957	0.081919
H	-0.330161	4.045997	0.745433
H	-1.574684	4.845410	-0.072494
H	-1.829214	3.282431	0.464625
H	-0.795034	3.546338	-0.822284
N	-0.643466	0.260272	3.393083
H	0.164309	0.855467	3.629405
H	-0.771369	-0.551127	4.004691
H	-0.490103	-0.125238	2.439307
H	-1.485250	0.839013	3.347727
N	-2.286522	0.547213	-2.563763
H	-1.689929	1.400121	-2.532065
H	-2.713862	0.377881	-1.625997
H	-1.711780	-0.275486	-2.852442
H	-3.025959	0.687325	-3.247655
N	2.721153	1.794935	0.519738
H	2.050953	1.967416	-0.243823
H	2.308377	2.060971	1.432689
H	3.549620	2.356047	0.337557
H	2.995089	0.785221	0.538014
N	2.187002	-0.817558	-3.368640
H	2.526986	-1.028941	-4.302798
H	1.561491	0.013347	-3.406990
H	2.985002	-0.601563	-2.733352
H	1.661509	-1.636122	-3.007630
N	1.350493	-3.164580	0.365042
H	1.004413	-3.399030	-0.579447
H	0.983003	-2.220558	0.576141
H	2.374161	-3.117121	0.331630
H	0.968636	-3.786120	1.094746
N	-0.536278	-2.403863	-2.415162
O	-1.403919	-2.606470	-1.563537
O	0.572852	-2.972099	-2.339294
O	-0.743748	-1.622265	-3.357186
N	0.609911	3.097933	2.631949
O	1.016393	3.894283	1.757339
O	-0.528469	3.177349	3.074342
O	1.388231	2.193354	3.015511
N	-3.214171	0.971857	0.748727
O	-3.215090	2.116485	0.290455
O	-3.328851	0.003041	-0.061357
O	-3.112830	0.737484	1.944117
N	-0.417025	-3.000548	3.049478
O	-0.484985	-3.967258	2.266644
O	-1.489578	-2.448799	3.416298
O	0.660013	-2.559835	3.429838
N	0.521029	2.261373	-2.645569
O	-0.598043	2.760972	-2.349475
O	1.523932	2.589540	-2.022380
O	0.561522	1.445130	-3.573333
N	3.664042	-1.140808	-0.584246
O	3.949503	-0.197673	-1.350236
O	3.347970	-0.890764	0.596310
O	3.660888	-2.301337	-0.996228
N	0.037484	0.074820	0.079257
O	0.554928	-0.175338	-1.008154
O	-0.255090	1.227895	0.404357
O	-0.187535	-0.863525	0.888934

n=8 (NH4NO3)n wB97X-D3/def2-SVPD

N	-1.732882	0.215744	-3.249411
H	-1.839197	1.166224	-3.650829
H	-2.616550	-0.328671	-3.256049
H	-1.428325	0.337865	-2.276229
H	-0.983569	-0.317456	-3.718459
N	-2.081935	-3.697848	-1.340376
H	-2.983227	-3.222194	-1.104473
H	-1.571312	-3.168139	-2.059109
H	-2.265259	-4.636285	-1.685353
H	-1.472425	-3.730951	-0.490206
N	-2.990317	-0.372462	1.198221
H	-2.165083	-0.364698	0.572180
H	-3.093790	-1.342875	1.517760
H	-3.796671	-0.093007	0.625661
H	-2.791301	0.263376	1.984638
N	-0.032494	4.276272	0.126523
H	0.924474	3.946906	-0.113880
H	0.010749	5.115126	0.698941
H	-0.525261	3.519887	0.643341
H	-0.550687	4.453316	-0.768258
N	0.064649	-0.813860	4.342032
H	-0.548949	-0.033483	4.039393
H	1.072275	-0.561135	4.222347
H	-0.133003	-1.651230	3.760461
H	-0.114553	-1.041506	5.316692
N	2.489829	1.749932	1.873076
H	1.647169	2.220817	2.226844
H	3.179608	2.428042	1.503066
H	2.195588	1.121305	1.107440
H	2.861871	1.146344	2.626354
N	2.223985	-2.835781	0.152340
H	2.719231	-2.132096	0.745234
H	1.286561	-3.046357	0.548087
H	2.766594	-3.696263	0.169197
H	2.142754	-2.497140	-0.830528
N	1.893462	1.221626	-2.740010
H	1.869325	0.531147	-3.495823
H	1.097743	1.876340	-2.799299
H	2.776888	1.755914	-2.663938
H	1.742072	0.716256	-1.850691
N	3.529372	3.081990	-0.752330
O	4.154518	3.365770	0.274260
O	4.103400	2.755844	-1.792683
O	2.270014	3.085522	-0.730463
N	-1.038647	1.927747	2.596752
O	-1.708901	1.123823	3.278398
O	-1.420602	2.213009	1.452486
O	-0.005752	2.418363	3.063578
N	-1.244197	3.345745	-2.733424
O	-1.817657	2.986609	-3.763164
O	-0.479716	2.547693	-2.133380
O	-1.411616	4.479532	-2.267110
N	0.217712	-0.283376	0.237554
O	0.081115	-0.502030	1.442706
O	-0.732261	-0.467172	-0.545934
O	1.313903	0.110755	-0.199131
N	2.891338	-1.260178	2.926770
O	2.457666	-2.369436	3.193883
O	3.485027	-1.033570	1.856377
O	2.710017	-0.299157	3.726958
N	-4.092074	-1.246041	-1.604015
O	-4.245346	-0.052105	-1.331799
O	-4.244005	-2.115797	-0.720944
O	-3.739789	-1.596639	-2.746415
N	0.990811	-1.898438	-2.985983
O	0.197323	-2.762304	-2.600197
O	0.674068	-1.102471	-3.891872
O	2.115592	-1.807990	-2.467974
N	-1.070238	-3.272579	1.842933
O	-0.517413	-3.149151	2.930749
O	-2.280629	-3.082907	1.698941
O	-0.391070	-3.620064	0.837571

n=9 (NH4NO3)n wB97X-D3/def2-SVPD

N	-2.610305	3.634160	-2.654203
H	-2.432607	4.521076	-3.116579
H	-3.066998	3.799187	-1.734376
H	-1.705664	3.178355	-2.422470
H	-3.162323	2.984822	-3.245462
N	-1.952748	1.757691	3.108025
H	-2.400181	2.034125	3.978213
H	-2.410225	0.893535	2.753092
H	-0.952456	1.558673	3.297652
H	-2.007272	2.546893	2.426223
N	-0.285913	-4.164488	0.742551
H	-0.945026	-3.493811	1.176969
H	-0.665673	-5.104971	0.802205
H	0.623375	-4.109611	1.251352
H	-0.113831	-3.918801	-0.260227
N	0.980659	2.950628	0.392134
H	1.512211	3.071725	1.258015
H	0.399610	3.773044	0.172096
H	0.361476	2.135450	0.526508
H	1.611125	2.720400	-0.391430
N	-1.541937	-0.917321	-4.886094
H	-0.945292	-1.693383	-4.521377
H	-2.478766	-0.943857	-4.430135
H	-1.639534	-0.967953	-5.895989
H	-1.100662	-0.014879	-4.601457
N	-2.440482	-0.906427	-0.561241
H	-3.050758	-0.209445	-1.032748
H	-1.662470	-0.412865	-0.092159
H	-2.065834	-1.567180	-1.255581
H	-2.962384	-1.409377	0.160431
N	2.211954	-1.413882	-1.773617
H	2.197700	-2.036339	-2.582954
H	2.199141	-1.996448	-0.924202
H	1.369605	-0.818972	-1.820553
H	3.049456	-0.795142	-1.746829
N	1.180360	-1.833212	4.012666
H	1.583180	-2.589372	3.416937
H	1.770728	-0.974775	3.965951
H	1.137899	-2.141457	4.981049
H	0.214350	-1.623126	3.698573
N	4.479200	0.346117	1.843159
H	3.910947	0.553664	2.691350
H	4.343122	1.098320	1.128479
H	4.139414	-0.558301	1.481567
H	5.463953	0.263760	2.078894
N	1.806266	1.389105	3.639633
O	2.690432	0.535250	3.928084
O	2.123284	2.432962	3.089532
O	0.632719	1.119635	3.932641
N	0.597166	-0.312627	0.914100
O	1.739114	0.149349	0.965305
O	-0.390145	0.455008	0.984380
O	0.410544	-1.528839	0.782241
N	2.836461	-3.114195	1.487179
O	2.856787	-3.332465	0.281449
O	3.572402	-2.282589	2.021360
O	2.029070	-3.757938	2.219161
N	-2.311557	-1.534913	2.558633
O	-2.309170	-2.594532	1.912254
O	-1.524884	-1.352389	3.497018
O	-3.121999	-0.644027	2.251681
N	-1.962913	4.207455	0.400985
O	-1.167074	4.716617	-0.404491
O	-1.562785	3.927216	1.548869
O	-3.128955	3.962766	0.068499
N	-0.288043	-3.120483	-2.682476
O	0.454454	-3.662088	-1.846496
O	0.195680	-2.765575	-3.772251
O	-1.484444	-2.926322	-2.434936
N	3.551090	1.432631	-1.008489
O	3.891442	2.199195	-0.086423
O	4.202639	0.389447	-1.205854
O	2.558637	1.694659	-1.694043
N	-3.796935	0.663404	-3.140254
O	-3.784251	-0.564026	-3.343411
O	-3.789911	1.460969	-4.086996
O	-3.787228	1.089953	-1.970181
N	-0.459973	1.117757	-2.547034
O	-0.224770	-0.061649	-2.226917
O	-0.573297	1.991109	-1.683322
O	-0.619979	1.413941	-3.743833

n=10 (NH4NO3)n wB97X-D3/def2-SVPD

N	2.850694	0.141157	1.396778
H	3.078385	1.035040	1.851263
H	1.946761	0.271994	0.911382
H	2.770855	-0.595033	2.117580
H	3.535971	-0.148087	0.683833
N	-3.275143	0.222215	-1.173419
H	-4.197435	0.007298	-0.799863
H	-2.568440	-0.109691	-0.487405
H	-3.195560	1.237757	-1.349137
H	-3.110422	-0.233277	-2.089494
N	0.697154	3.902929	-1.416804
H	-0.062868	4.589300	-1.399245
H	1.269827	4.025447	-2.270931
H	0.258349	2.972716	-1.524874
H	1.246527	3.912728	-0.538341

N	2.221707	-4.198060	2.378543
H	2.375304	-3.320162	2.935852
H	1.266930	-4.573158	2.572618
H	2.923735	-4.895771	2.609783
H	2.319911	-3.936798	1.388443
N	-0.478649	1.773770	4.322995
H	-0.023299	2.490414	3.720447
H	-1.462310	1.640648	4.008216
H	-0.458831	2.086827	5.290303
H	0.062955	0.889703	4.243882
N	0.263119	-3.159745	-1.076191
H	0.262410	-2.539749	-0.249873
H	-0.383458	-2.758796	-1.776967
H	-0.039416	-4.090550	-0.755415
H	1.224904	-3.197283	-1.445842
N	-1.943733	-2.572136	3.493496
H	-2.272455	-3.205059	4.219360
H	-1.964564	-3.099476	2.592420
H	-0.965566	-2.319577	3.720946
H	-2.543747	-1.723265	3.467635
N	2.351596	-0.826309	-3.803457
H	2.783410	-0.904892	-4.719891
H	2.514753	0.136213	-3.441339
H	2.723693	-1.533434	-3.139400
H	1.329915	-0.945505	-3.896028
N	-1.600257	2.621676	0.942975
H	-0.888805	3.201527	1.421653
H	-2.028056	3.171809	0.175915
H	-2.294848	2.299646	1.647028
H	-1.069586	1.822567	0.571973
N	-1.239945	1.945186	-4.988202
H	-1.657240	2.103218	-5.901705
H	-1.423778	2.770357	-4.379931
H	-0.213621	1.808336	-5.097606
H	-1.664374	1.093577	-4.558272
N	-3.465865	0.605310	2.841199
O	-3.465688	-0.270539	3.715374
O	-3.876687	0.392754	1.707317
O	-3.017882	1.747803	3.134369
N	-0.757625	-4.813841	1.510683
O	-0.053120	-5.335282	0.636348
O	-0.422401	-4.905481	2.711120
O	-1.770550	-4.167593	1.204101
N	1.654936	3.267351	2.079541
O	2.283835	2.534905	2.842029
O	0.501179	3.652759	2.422019
O	2.125447	3.621860	0.997603
N	0.408855	0.639646	-1.211390
O	0.920247	-0.416298	-1.544244
O	0.643759	1.129999	-0.081144
O	-0.361343	1.263679	-1.970241
N	-2.336388	3.695590	-2.273988
O	-1.694176	4.087764	-3.257288
O	-3.117518	2.741740	-2.370973
O	-2.167346	4.264899	-1.176039
N	1.570609	-1.383640	4.128596
O	2.608543	-1.785379	3.544868
O	0.712113	-2.201400	4.470038
O	1.449234	-0.179320	4.345548
N	1.835929	2.357097	-4.048848
O	1.455136	3.532380	-4.032753
O	1.525739	1.616226	-5.005168
O	2.502987	1.897162	-3.106391
N	-0.602953	-0.917562	1.344427
O	-1.323873	-0.990810	0.335636
O	0.336545	-1.723508	1.468532
O	-0.813266	-0.066469	2.212385
N	3.367327	-2.149465	-0.845644
O	3.986305	-1.091774	-0.959268
O	2.969090	-2.753822	-1.871809
O	3.114139	-2.618242	0.272028
N	-1.480483	-1.150557	-3.644037
O	-2.387083	-0.300328	-3.823126
O	-0.473548	-1.125914	-4.354062
O	-1.636619	-1.989944	-2.753033
