# Supplementary material for: Decomposition and Growth Pathways for Ammonium Nitrate Clusters and Nanoparticles
Source: J Phys Chem A. 2024 Oct 14;128(42):9184–94. doi: 10.1021/acs.jpca.4c04630 (PMC11514028; doi:10.1021/acs.jpca.4c04630)
Supplement: Supplementary file 2 — jp4c04630_si_002.zip [file jp4c04630_si_002.zip › SI_ammoniumnitrate particle structures_PDF_XYZ/HassanAmatTopper_SuppMats_S15.pdf]

Supporting Information for:

Decomposition and Growth Pathways for Ammonium Nitrate Clusters and Nanoparticles

Ubaidullah S. Hassan, Miguel A. Amat, and Robert Q. Topper*

Author Affiliations:

Ubaidullah S. Hassan, Department of Chemistry, The Cooper Union for the Advancement of Science and Art, New York NY 10003, United States.

Miguel A. Amat, Department of Chemistry, The Cooper Union for the Advancement of Science and Art, New York NY 10003, United States.

Robert Q. Topper, Department of Chemistry, The Cooper Union for the Advancement of Science and Art, New York NY 10003, United States. Email: topper@cooper.edu; Phone: 212-353-4370.

Table S7: Cartesian Coordinates of n=(2-40) Neutral Clusters: OPLS-AA

n=2 OPLS/AA

N	-3.2193959414	-0.2103563849	1.5869527190
H	-3.6834808808	-0.8671963879	0.9486561758
H	-3.8870863899	0.1017105629	2.3018221970
H	-2.8815480343	0.6013220868	1.0566015388
H	-2.4254689127	-0.6772624411	2.0407303427
N	-0.5849478503	-1.8239035805	-1.7374538244
H	-0.0620408914	-2.2386560598	-2.5177004463
H	-1.5495338703	-2.1756505703	-1.7465395618
H	-0.5924153826	-0.8018498785	-1.8353618949
H	-0.1358007474	-2.0794582173	-0.8502141546
N	-2.0812733613	0.6596617152	-1.0312588268
O	-2.2959946502	1.4982159917	-0.1403382892
O	-1.0487164307	0.7334709507	-1.7178418829
O	-2.8991096614	-0.2527025312	-1.2355964730
N	-1.0534402599	-2.2176600792	1.1845125023
O	0.0760432125	-2.4122172599	0.7055213750
O	-2.0634926172	-2.5900153932	0.5647031230
O	-1.1728714712	-1.6507471282	2.2833138934

n=3 OPLS/AA

N	-2.3026856198	-0.4628079540	0.1528862500
H	-2.4103163660	-0.7832373862	-0.8166377049
H	-3.2288252481	-0.2894293854	0.5608567712
H	-1.7542814861	0.4051041561	0.1676171491
H	-1.8173194838	-1.1836695125	0.6997078402
N	0.6220017385	-0.3605292071	-2.4607061102
H	1.3450921219	-0.6545933656	-3.1277163543
H	0.3400734226	-1.1630797263	-1.8856723943
H	-0.1927139387	-0.0043208213	-2.9741157666
H	0.9955560524	0.3798767981	-1.8553205751
N	1.9920757770	-0.5527244405	2.7944379409
H	1.7577171008	0.3370113270	2.3387161261
H	2.9606783510	-0.5189328771	3.1334097234
H	1.8923246436	-1.3197950794	2.1192432501
H	1.3575827845	-0.7091802661	3.5863822200
N	0.7821871761	1.3439917481	0.3954606945
O	-0.4546091089	1.3198236494	0.2824617418
O	1.5004562976	1.1994658624	-0.6076443091
O	1.3007147571	1.5126858684	1.5115655494
N	-2.3449089685	0.0776670627	-2.7889534937
O	-2.2156206960	-1.0812347998	-2.3608776515
O	-1.5332208321	0.5292989141	-3.6137013042
O	-3.2858861350	0.7849376431	-2.3922812061
N	0.4056220259	-2.0657540074	0.6348553962
O	0.1182304925	-2.0686646156	-0.5736211431
O	1.5681582437	-2.3108920935	0.9973947346
O	-0.4695233631	-1.8177051133	1.4807932779

n=4 OPLS/AA

N	-0.2800215776	0.5488558633	-3.8051113310
H	0.1768667024	1.1037873595	-4.5382826833
H	-0.8121339687	1.1716215995	-3.1860362738
H	0.4324713540	0.0504945771	-3.2590111801
H	-0.9172899531	-0.1304795422	-4.2371159009
N	-0.4084167746	-2.9103359949	-0.6212028062
H	-1.1681450125	-2.9518039584	0.0682386417
H	0.2650481016	-2.1861015507	-0.3452241550
H	-0.7960898507	-2.6832445664	-1.5444441578
H	0.0655189234	-3.8201939443	-0.6633808823
N	-4.8611589007	-0.4452467833	-1.5977399709
H	-4.5531446091	0.5224420927	-1.7492045971
H	-4.5740421749	-0.7532041197	-0.6612780388
H	-4.4337247411	-1.0550815432	-2.3045901169
H	-5.8837237778	-0.4951426207	-1.6758872782
N	-0.7336533023	1.5530275670	0.7571438815
H	-1.4408506359	2.1582153458	0.3237205533
H	0.0523386804	1.4264629981	0.1087360636
H	-1.1502984984	0.6396104885	0.9723450557
H	-0.3958034442	1.9878220251	1.6237734314
N	-2.4018947129	-1.7235997379	-3.5564676448
O	-3.6041576506	-1.8828752732	-3.2877427979
O	-2.0712912830	-1.1153825512	-4.5878701545
O	-1.5302345033	-2.1725417507	-2.7937893681
N	1.1880155183	-0.1818827952	-1.0911035479
O	1.3281970326	-0.7038636337	-2.2095413236
O	1.1130547357	1.0536862794	-0.9873352781
O	1.1227947340	-0.8954716059	-0.0764332250
N	-2.7729262716	-1.5604205317	0.8857702867
O	-1.9050835678	-0.6952534855	1.0891244962
O	-2.4674026196	-2.7642106507	0.9094886005
O	-3.9462935720	-1.2217971863	0.6586975807
N	-2.7613457153	2.1894066523	-1.5251419666
O	-3.8987553156	1.8916125506	-1.9259361597
O	-1.7644278922	1.8767286000	-2.1970089180
O	-2.6208538252	2.7998792977	-0.4524799585

n=5 OPLS/AA

N	-2.0832397464	-1.2737816169	-0.7900371257
H	-1.3417255992	-0.9071664196	-0.1817722457
H	-2.3515011075	-0.5559429300	-1.4733951190
H	-2.8994589886	-1.5262430584	-0.2205769486
H	-1.7402725682	-2.1057737027	-1.2844035969
N	0.6735658234	-1.5517505509	-3.5273518854
H	1.4430237817	-1.9727322949	-4.0611542077

H	1.0560087665	-1.0239369482	-2.7340140750
H	0.0535598895	-2.2922359211	-3.1787696388
H	0.1416716051	-0.9180974496	-4.1354701403
N	3.5231812548	-5.1832949099	-2.0676079554
H	4.0501312829	-4.4521286585	-2.5594978601
H	4.0007841299	-5.4172532728	-1.1893177621
H	3.4654854931	-6.0204134271	-2.6593336616
H	2.5763246268	-4.8433835691	-1.8622830169
N	1.5241472900	-2.8925137677	2.1056845673
H	1.7867452180	-1.9475664642	1.8017881714
H	1.1138740835	-2.8480230942	3.0458607005
H	2.3587287720	-3.4903090464	2.1243949620
H	0.8372413424	-3.2841555458	1.4506941392
N	4.2073191831	-0.9702101226	-0.8316641010
H	3.3902166247	-0.6128380736	-0.3228765959
H	4.4340215171	-1.9135742342	-0.4956508720
H	5.0086371517	-0.3483502956	-0.6722420424
H	3.9964006428	-1.0060775389	-1.8358863981
N	1.1563034539	-0.6113129258	0.0742736241
O	2.1518852198	-0.6715297562	0.8146848555
O	1.3015283636	-0.5449208250	-1.1576028817
O	0.0154958598	-0.6174882012	0.5657392941
N	3.6468564091	-2.4370177075	-3.5266149508
O	3.5428493642	-1.2179390544	-3.3120277121
O	2.7818885226	-3.0258407200	-4.1960445036
O	4.6158321207	-3.0672738556	-3.0717722706
N	4.1615368610	-4.3395291127	0.8479720646
O	4.4344689474	-5.4335802469	0.3268405046
O	4.4089249512	-3.2838892038	0.2418025944
O	3.6412162654	-4.3011178564	1.9752740022
N	-1.9287119660	-0.2289257118	-3.5392019334
O	-2.1942554942	-1.4406856860	-3.6035627174
O	-2.4840054958	0.4841859761	-2.6870641938
O	-1.1078742471	0.2697229759	-4.3269795232
N	0.2166833694	-3.6495325882	-0.9297333594
O	-0.1081968442	-3.6407454329	0.2691801252
O	-0.5509371760	-3.1775233363	-1.7847093096
O	1.3091850079	-4.1303293823	-1.2736711708

n=6 OPLS/AA

N	1.3674100053	-4.3542377339	4.0062273734
H	0.7869292110	-5.0576287197	3.5345078952
H	2.2541637232	-4.2468767564	3.4998913685
H	1.5573877828	-4.6569463829	4.9687820170
H	0.8711587390	-3.4554997615	4.0217277534
N	-1.7767824099	-4.1928113439	0.8967747196
H	-1.7097956876	-3.8371045730	1.8576189160
H	-1.2203082464	-3.5977063275	0.2719377726
H	-2.7582683787	-4.1819308502	0.5954403007
H	-1.4187572616	-5.1545032785	0.8621028248
N	0.7976924226	-1.6394541666	-2.9321372357
H	1.2477248639	-2.4786737237	-3.3160870168
H	0.0690730840	-1.3155050110	-3.5789799441
H	1.4997589261	-0.9003301514	-2.8095246795
H	0.3742132548	-1.8633085977	-2.0239576762
N	1.4969990804	-0.2701698784	1.7132551061
H	2.2508729889	-0.9597303942	1.8153164725
H	1.6584369798	0.5115326103	2.3590985154
H	0.5964202067	-0.7130091446	1.9302495779
H	1.4822668802	0.0805267435	0.7483559581
N	2.8821608460	-5.4318119678	0.1896638575
H	2.4925583117	-6.3442081820	0.4541995094
H	3.0541859146	-5.4135538007	-0.8224175131
H	3.7657381639	-5.2789427945	0.6898214960
H	2.2161606145	-4.6905439825	0.4370521953
N	4.7193988347	-1.6359694085	-1.0251659326
H	4.2070019105	-0.7701740821	-0.8200222211
H	4.6804873017	-2.2581846029	-0.2093419136
H	5.6984775998	-1.4119059878	-1.2383228686
H	4.2916280278	-2.1036121181	-1.8329765274
N	2.9394848230	-3.9371950190	-2.9295094826
O	1.8498853388	-3.9109723105	-3.5254148817
O	3.2764733986	-4.9509142105	-2.2956088503
O	3.6920963373	-2.9496977411	-2.9675047464
N	0.9302886963	-2.9277705158	-0.0731359389
O	1.9445592245	-2.3150579871	-0.4457705639
O	-0.1541037126	-2.7427516697	-0.6500926453
O	1.0004106334	-3.7255025327	0.8764561570
N	-0.6221582339	-2.1681361776	3.0700271653
O	0.3291428399	-1.9736245297	3.8447589877
O	-1.3555844381	-3.1588667072	3.2235098412
O	-0.8400332791	-1.3719166550	2.1418119198
N	2.3897094868	0.4597164908	-1.1934514546
O	3.3841641985	0.5190862021	-0.4514588841
O	2.5304965329	0.2087292349	-2.4018397680
O	1.2544668150	0.6513341897	-0.7270553363
N	3.8628991541	-3.0793579651	1.9287069351
O	3.4545936314	-1.9300271188	2.1639581132
O	4.6113806046	-3.2862774618	0.9591824377
O	3.5227229525	-4.0217700734	2.6629808456
N	0.2223163724	-6.6162251010	1.6135874811
O	1.2927786089	-7.1610275342	1.2968560177
O	-0.6957100548	-6.5205875797	0.7822639253
O	0.0698804404	-6.1670598275	2.7616434245

n=7 OPLS/AA

N	-1.8388612523	-5.5367478429	1.8766236819
H	-1.3274468188	-5.9749074954	2.6516769018
H	-2.7441165892	-6.0058347435	1.7553597533
H	-1.2904725963	-5.6226028050	1.0128330062
H	-1.9934085071	-4.5436467546	2.0866258212
N	-0.0152886629	0.4699698279	1.1656916369
H	0.7900517593	0.4520594267	1.8023564777
H	-0.3996788053	1.4212639476	1.1267249628
H	-0.7360179701	-0.1766700157	1.5072201167
H	0.2844911489	0.1832259356	0.2264656104
N	0.1460011185	-3.1112993931	4.2416658196
H	0.5419531511	-2.2114528888	4.5378656748
H	0.3368825395	-3.8216306117	4.9580655184
H	0.5737806411	-3.3976228434	3.3532632724
H	-0.8686114720	-3.0144903520	4.1174691012
N	-0.9768011728	-2.5483272630	-1.4776246780
H	-0.2998850094	-1.7845717013	-1.3649390662
H	-1.4917200013	-2.6816431687	-0.5993763334
H	-0.4793665961	-3.4139217645	-1.7175007397
H	-1.6362324252	-2.3131716738	-2.2286824629
N	4.0900559411	-1.2972536718	1.2675711081
H	3.5056370505	-1.0677207306	0.4551640139
H	3.4898117301	-1.4796636700	2.0803860718
H	4.7147561725	-0.5088486135	1.4734900461
H	4.6500182422	-2.1327814497	1.0612435096
N	3.0174683968	-4.1986505666	-2.1978086656
H	3.1286502008	-4.4171822862	-3.1948636248
H	2.6164331944	-3.2589438273	-2.0960408119
H	3.9339032942	-4.2315226555	-1.7359623398
H	2.3908870061	-4.8869537101	-1.7643688572
N	2.6728535216	-6.0664374613	1.5327193978
H	2.3024169167	-6.5654835638	0.7154276464
H	2.2404825435	-5.1369870115	1.5911307338
H	3.6903860734	-5.9653350096	1.4397606741
H	2.4581281920	-6.5979447463	2.3845577410
N	0.4257849939	-5.5471192391	-1.0682186587
O	-0.4981747367	-5.8081610732	-0.2800730580
O	1.5356345897	-6.0862060431	-0.9245621123
O	0.2398949791	-4.7469899567	-2.0000215560
N	1.8635769845	-0.6525310248	3.4304912341
O	2.0730694433	0.3095446327	2.6731661719
O	0.9210720572	-0.6130462266	4.2386640644
O	2.5965900431	-1.6540922869	3.3796434250
N	-2.1429266737	-2.3763836560	1.7602140967
O	-1.9517876571	-1.1497513749	1.8033074164
O	-2.3675521675	-2.9321249724	0.6722283947
O	-2.1094401696	-3.0472751608	2.8051073201
N	0.6107117032	-6.2844878244	4.2392370076
O	1.5928745474	-6.8668617368	3.7501412249
O	-0.5293431409	-6.5171266011	3.8042978591
O	0.7686038303	-5.4694744791	5.1632726827
N	1.6830546159	-0.9091420086	-1.3825677447
O	0.5625387315	-0.3871931158	-1.2599650651
O	2.6469603581	-0.4704634297	-0.7333645905
O	1.8396648842	-1.8697702536	-2.1543741999
N	5.2886382911	-4.4066022407	0.2339411210
O	5.2456774771	-4.4300334372	-1.0072773362
O	5.5421667667	-3.3448197831	0.8266967500
O	5.0780704599	-5.4449543377	0.8824044712
N	1.2701256176	-3.2659609221	1.0961013174
O	2.3035270173	-3.4737545931	0.4388941596
O	0.6069652039	-2.2338994313	0.9009468768
O	0.8998843336	-4.0902294055	1.9484636021

n=8 OPLS/AA

N	-1.0424737713	-0.8008087445	-3.6917183344
H	-1.2770475251	0.1175301680	-4.0865156159
H	-1.8716515719	-1.4058636236	-3.7163649077
H	-0.7311682948	-0.6862156596	-2.7200224718
H	-0.2900279217	-1.2286849682	-4.2439707266
N	-1.4050367180	-4.8000263350	-1.7788651440
H	-2.2845056474	-4.2916855250	-1.6293321355
H	-0.8216008217	-4.2849276167	-2.4485744472
H	-1.6091334547	-5.7349457175	-2.1510288100
H	-0.9049078046	-4.8885459857	-0.8865250376
N	-1.9892715725	-1.1972505072	0.4432839322
H	-1.1123542723	-0.9670204440	-0.0386354222

H	-1.9618978011	-2.1751278600	0.7551192172
H	-2.7783467539	-1.0651995227	-0.2002674797
H	-2.1044866088	-0.5816539778	1.2569189441
N	0.3395889639	3.1134817633	-0.6295539688
H	1.3149404351	2.8524626141	-0.8160870997
H	0.3132014794	4.0276594974	-0.1628447910
H	-0.0938125798	2.4071085783	-0.0233893464
H	-0.1759725294	3.1666961093	-1.5158948198
N	0.7850159076	-2.0303189215	3.6000155508
H	0.2540049765	-1.2453606816	3.2049154997
H	1.7905327721	-1.8472040532	3.5018347702
H	0.5437823176	-2.8934163090	3.0989119897
H	0.5517430469	-2.1352938778	4.5943995587
N	3.3433084058	0.5508148571	1.2948000123
H	2.5431296935	1.0092198181	1.7462587787
H	4.0141800996	1.2626724225	0.9826707722
H	3.0162138193	0.0104811779	0.4853037673
H	3.7997092315	-0.0791135436	1.9649671709
N	2.7011210421	-3.9395405690	-0.2339339033
H	3.1400312266	-3.2311789341	0.3659141957
H	1.7735816399	-4.1756640309	0.1377528648
H	3.2869734719	-4.7825419752	-0.2529361042
H	2.6038982574	-3.5687766457	-1.1864659854
N	2.6800091923	0.3291218678	-3.1462147430
H	2.7689957904	-0.3717448064	-3.8912673505
H	1.8766303905	0.9375286361	-3.3428321135
H	3.5357696109	0.8949668479	-3.1046324318
H	2.5386410642	-0.1442638890	-2.2461278018
N	4.0106606217	2.2298223399	-1.1150609313
O	4.7397931824	2.2401051807	-0.1094377996
O	4.4926669781	1.9657322115	-2.2290376759
O	2.7995207296	2.4836298319	-1.0067072313
N	-0.0928792289	0.8686300805	1.9325161099
O	-0.5998816767	-0.0394717255	2.6117197162
O	-0.7156537440	1.3454981619	0.9693096693
O	1.0368986436	1.2998641523	2.2165191729
N	-0.6046824944	2.2025333362	-3.4910992527
O	-1.3898101471	1.6908490348	-4.3064345552
O	0.5236651757	1.7110334643	-3.3229406302
O	-0.9479027880	3.2057183171	-2.8439220516
N	1.0423412399	-1.0137727215	-0.4916518836
O	0.8732740502	-1.6555464143	0.5583770319
O	0.0796057156	-0.7880523283	-1.2434553987
O	2.1741448651	-0.5977190869	-0.7898775242
N	3.4770073824	-2.3646673968	2.2945528756
O	3.0800794432	-3.5063119023	2.5811081847
O	4.0309010288	-2.1555550643	1.2025401597
O	3.3200415488	-1.4321344731	3.1000109309
N	-3.2431383031	-2.3619812652	-2.1740082719
O	-3.3571923563	-1.1893158198	-1.7804789375
O	-3.4827809169	-3.3031132674	-1.3995020531
O	-2.8894413515	-2.5935148947	-3.3420447655
N	1.3441634209	-2.7922410060	-3.3075310608
O	0.2989616718	-3.4471652641	-3.1604071989
O	1.3834159425	-1.8614470783	-4.1291756401
O	2.3501134582	-3.0681108976	-2.6330098004
N	-0.4083158839	-4.2033746490	1.1766732239
O	0.0420636504	-4.1226899605	2.3315178745
O	-1.5131078900	-3.7049243922	0.9046440426
O	0.2460971149	-4.7825100605	0.2938570438

n=9 OPLS/AA

N	-4.6363514246	3.3784793970	-5.0488112870
H	-4.2584968989	4.1257611636	-5.6429762610
H	-5.0274153853	3.7872532927	-4.1919521584
H	-3.8848117901	2.7233503600	-4.8033939960
H	-5.3746812561	2.8775534994	-5.5569233112
N	-4.0194800119	1.4876236250	0.5559790438
H	-4.6602689830	1.6746538692	1.3361362586
H	-4.3035170838	0.6236829351	0.0793616799
H	-3.0637576793	1.3815741387	0.9159566175
H	-4.0503769255	2.2705837393	-0.1075376209
N	-2.2342468293	-4.1660889191	-1.9504270789
H	-2.9848630235	-3.5785334530	-1.5688645012
H	-2.4741471139	-5.1557855390	-1.8193426046
H	-1.3555116109	-3.9592343513	-1.4612735980
H	-2.1224662998	-3.9708017610	-2.9522272402
N	-1.1628708133	2.6834429290	-2.1247089297
H	-0.6484784200	2.8315294158	-1.2485200484
H	-1.7367462381	3.5100858080	-2.3285795570
H	-1.7695039019	1.8604221989	-2.0305351661
H	-0.4967541922	2.5317344373	-2.8912000938
N	-3.7174513775	-0.9217189842	-7.4254846910
H	-3.3418630064	-1.7535822004	-6.9551942886
H	-4.7159979701	-0.8262360868	-7.2063445439
H	-3.5976044988	-1.0190433560	-8.4405710009
H	-3.2143396688	-0.0880151038	-7.0998284728
N	-4.4585081462	-0.7731629183	-3.1444204241
H	-5.1123251347	-0.2288314874	-3.7192793080
H	-3.5791374295	-0.2533062510	-3.0410237440
H	-4.2717522645	-1.6746154272	-3.5991083820
H	-4.8708183930	-0.9358979776	-2.2182708223
N	0.0252230270	-1.6089654604	-4.3340584480
H	0.1748208756	-2.1784369641	-5.1752235850
H	0.0164396644	-2.2166237119	-3.5064661647
H	-0.8738206777	-1.1185940148	-4.4081650359
H	0.7834523915	-0.9222077053	-4.2463798255
N	-0.9151777493	-1.6032641899	1.3882706181
H	-0.5446243672	-2.2603560544	0.6917388025
H	-0.4443941588	-0.6963570378	1.2876877475
H	-0.7472984030	-1.9733176664	2.3311982260
H	-1.9243937073	-1.4830266408	1.2424570182
N	2.1787863720	0.2456591341	-0.5203187709
H	1.5641144734	0.5609491190	0.2392908527
H	2.0398767497	0.8434543733	-1.3434710377
H	1.9536681685	-0.7266439939	-0.7615322567
H	3.1574854975	0.3048773451	-0.2155619019
N	-0.4519961454	1.4647922241	1.0679956673
O	0.4554646886	0.6844660003	1.4005971256
O	-0.1986119769	2.4365335172	0.3368778702
O	-1.6128420825	1.2733770007	1.4665123269
N	-1.6190579534	-0.4187558371	-1.6966377275
O	-0.4928611021	-0.2524865141	-1.1995933884
O	-2.4798431583	0.4711730377	-1.5961721118
O	-1.8844698137	-1.4749548850	-2.2941481634
N	0.6263317424	-2.9801166264	-1.1066905050
O	0.5525245513	-3.2207199373	-2.3231116723
O	1.5530816139	-2.2793488280	-0.6672851350
O	-0.2266116248	-3.4402814845	-0.3296740821
N	-4.1207516730	-1.6655103121	-0.0768429662
O	-4.0151420123	-2.7514746531	-0.6705987751
O	-3.4436977313	-1.4458347704	0.9411739921
O	-4.9034159056	-0.7992208155	-0.5011044571
N	-4.0700436787	4.0545292685	-2.1240554442
O	-3.0254355111	4.4282461668	-2.6827569452
O	-4.0153371266	3.4872563393	-1.0203224476
O	-5.1693592832	4.2480854553	-2.6690873787
N	-2.7683689826	-3.2365147918	-5.0459268166
O	-1.8272190802	-3.8269154129	-4.4903353491
O	-2.6883133857	-2.9331977436	-6.2478451918
O	-3.7895753041	-2.9494309879	-4.3995993887
N	1.3113123093	1.2216000507	-3.4392128461
O	1.7699704681	1.8539056119	-2.4733762753
O	1.8334107372	0.1482587257	-3.7832615964
O	0.3305549329	1.6626361695	-4.0610011671
N	-6.0657937241	0.7509252508	-5.9656313841
O	-6.0773374474	-0.2465736554	-6.7058209445
O	-6.2210601689	1.8847034992	-6.4488053959
O	-5.8989834216	0.6146457991	-4.7422668270
N	-2.6257236185	0.8800204837	-5.1501143191
O	-2.1249545070	-0.2390933229	-4.9505335191
O	-2.9166581059	1.6107017892	-4.1886155594
O	-2.8355584117	1.2684532975	-6.3111948134

n=10 OPLS/AA

N	5.8892151280	-4.0215886707	5.2289469600
H	5.9657451571	-3.0300624894	5.4843977066
H	5.0321662401	-4.1692354007	4.6831426615
H	5.8548263601	-4.5929572461	6.0813499678
H	6.7041228293	-4.2940985810	4.6668977528
N	0.3449112237	-3.9647740204	2.8923265679
H	-0.6085144326	-4.1968610814	3.1945701975
H	1.0203849355	-4.3597649862	3.5571213385
H	0.4544444470	-2.9444281976	2.8586754312
H	0.5133290162	-4.3580420424	1.9589395988
N	4.0916139493	0.0925780227	2.0031940966
H	3.2840174641	0.7232257215	1.9375625901
H	4.7871010566	0.3521633893	1.2938649723
H	3.7847296991	-0.8742131133	1.8439066822
H	4.5106067910	0.1691367076	2.9374420779
N	5.5541100537	-7.9087161381	6.3974768033
H	5.5001533045	-7.0226306233	6.9134004676
H	4.6331794949	-8.3627165246	6.3997660114
H	6.2408266684	-8.5254047817	6.8473111416
H	5.8422806944	-7.7241117597	5.4294300950
N	3.1797221417	-2.5622128479	7.8326195525
H	3.6441704552	-1.6903864622	7.5525434647
H	2.2275242035	-2.3542672916	8.1555938818
H	3.7102184942	-3.0036815350	8.5928264601
H	3.1369758665	-3.2005152538	7.0295141306
N	3.5194778739	-7.3764934515	2.8080540499
H	3.5581944035	-6.7600557113	3.6282623408
H	2.8368442916	-7.0083963250	2.1351866162
H	3.2369345411	-8.3190396029	3.1013312934

H	4.4459382970	-7.4184815663	2.3674367480
N	1.3663755900	-6.6841503631	7.3542539918
H	0.8415835935	-7.1473478993	8.1054380574
H	1.3480388361	-7.2708750175	6.5118451666
H	2.3388001784	-6.5436900279	7.6524097200
H	0.9370792410	-5.7746889589	7.1473237548
N	5.5214033283	-4.3942526839	0.7349033431
H	4.7310967327	-4.6351826945	0.1253097796
H	6.0553707202	-3.6244061081	0.3148441096
H	6.1307623033	-5.2132647951	0.8450683349
H	5.1683827873	-4.1041573725	1.6543905545
N	1.4233770645	-0.8512983274	5.0322010426
H	2.0908782214	-0.3854024115	5.6579959760
H	1.0423520195	-0.1673698720	4.3678995535
H	0.6580960215	-1.2533201580	5.5862397569
H	1.9021826458	-1.5991004144	4.5166694935
N	2.1411299204	-2.2105971675	-0.9205841783
H	1.6889957499	-2.1260274483	-1.8385486395
H	1.8022392136	-1.4643615972	-0.3021019652
H	3.1585406192	-2.1277484570	-1.0312507282
H	1.9147436585	-3.1242510852	-0.5104362742
N	0.2064455847	-3.2282533461	6.6358991690
O	0.2441281245	-4.3686011263	7.1270253006
O	-0.2409294618	-3.0602936071	5.4893116212
O	0.6161384214	-2.2558645219	7.2913611128
N	2.5488798295	-8.9158657355	5.3993852108
O	3.0044080980	-9.3991057714	4.3496276315
O	3.2156128169	-8.9651083690	6.4463141469
O	1.4266176701	-8.3833826374	5.4022138561
N	4.5866798251	-0.7038106845	5.6111147425
O	5.3947264683	-1.5830025154	5.9533138917
O	3.6536042629	-0.3891373187	6.3683257958
O	4.7117088446	-0.1392917649	4.5117036551
N	3.5459317784	-2.9347885010	2.7770468155
O	4.3525756293	-3.8471175629	3.0219943433
O	2.7688060183	-2.5387775220	3.6615064067
O	3.5164136638	-2.4184700022	1.6476387872
N	1.0494468837	-0.4528316834	1.9449160935
O	1.3504900595	-0.4270296671	0.7400405094
O	0.3668482120	-1.3881431442	2.3946598380
O	1.4310026867	0.4566784932	2.7000485410
N	4.6730921047	-5.4037458598	8.1935619490
O	5.6499784846	-5.4767736280	7.4297701863
O	3.8105182562	-6.2975722137	8.1858993237
O	4.5587794814	-4.4368909593	8.9650169582
N	5.5734224576	-1.4009951814	-0.4649447475
O	5.5725739761	-0.2115978296	-0.1066834872
O	4.6539723010	-1.8354318450	-1.1783128893
O	6.4937218364	-2.1559564773	-0.1098375800
N	2.9259675250	-5.2064414082	5.1493322946
O	1.8836245327	-5.3258606964	4.4842924229
O	3.8480322091	-6.0273291318	5.0116443337
O	3.0462459301	-4.2661336394	5.9520607734
N	6.4783750538	-6.4348860073	3.0993863847
O	7.0609331194	-5.3462418871	3.2353937032
O	5.9825108286	-6.7411804243	2.0024268088
O	6.3916811437	-7.2172363403	4.0603394158
N	2.1032898078	-5.0961135909	0.4324320435
O	1.1533787294	-4.2992932135	0.3565101080
O	3.1284885841	-4.9065262312	-0.2428767844
O	2.0280020493	-6.0825221220	1.1836634117

n=11 OPLS/AA

N	3.3606407553	-5.4796352058	-7.3335744863
H	3.1593431958	-4.5719280564	-7.7692155326
H	2.9675298826	-5.4988179359	-6.3852440850
H	4.3768477205	-5.6184381811	-7.2856927374
H	2.9388420261	-6.2293557656	-7.8941460146
N	-2.0551294084	-7.6760428428	-5.6826540298
H	-1.4942885311	-7.6623018547	-6.5425986753
H	-1.4836209716	-8.0308640699	-4.9069513232
H	-2.8696740585	-8.2869734533	-5.8150032881
H	-2.3729335260	-6.7240319800	-5.4660636700
N	2.7385719752	-6.0694650978	-2.4446045095
H	2.9791275105	-6.0886276862	-3.4426034711
H	2.0963759676	-6.8419990668	-2.2324483405
H	2.2856694790	-5.1760832604	-2.2188087040
H	3.5931151778	-6.1711503963	-1.8845584946
N	-0.4935444723	-5.0468798599	-8.9066444170
H	-1.2183106288	-5.0044950531	-8.1805909451
H	-0.9309478185	-5.2352351546	-9.8162791136
H	0.1703683308	-5.7978242311	-8.6840733826
H	0.0047115215	-4.1499649595	-8.9456335198
N	0.3018442452	-2.4115241651	-5.5877975664
H	0.6508609800	-2.0890309558	-4.6776209097
H	0.7017592130	-3.3329046913	-5.8007779173
H	0.5767835727	-1.7423712432	-6.3164114954
H	-0.7220264450	-2.4817894558	-5.5563790568
N	6.4652948979	-6.3811962739	-4.1519059184
H	6.4661990270	-7.4077446746	-4.1310813877
H	5.5072493093	-6.0427125405	-4.2995951123
H	7.0657966229	-6.0538794587	-4.9177367776
H	6.8219346331	-6.0204494215	-3.2592103758
N	-2.0248270054	-4.9552545884	-2.7081144778
H	-1.6612044196	-5.8872210340	-2.4769148733
H	-2.7257831222	-4.6786659573	-2.0106949935
H	-2.4583184386	-4.9781551932	-3.6385962152
H	-1.2540016872	-4.2769770770	-2.7062516038
N	4.3300709454	-1.4423612773	-6.6437425671
H	3.7625896121	-2.0143512492	-6.0073240679
H	3.7525633227	-1.1359421655	-7.4354666094
H	5.1200478601	-1.9985314337	-6.9913727663
H	4.6850824338	-0.6206208178	-6.1408602049
N	7.2569192225	-2.6853325864	-3.8643891801
H	6.2360638908	-2.7756860899	-3.9270528358
H	7.5883198525	-3.1370871005	-3.0039670937
H	7.5121970432	-1.6909318129	-3.8490314398
H	7.6910951093	-3.1376254301	-4.6775054123
N	3.7633021128	-1.5479849108	-1.7826075314
H	4.2813597562	-0.7930521350	-2.2472890135
H	4.3828137987	-2.3556127519	-1.6477845852
H	2.9638264645	-1.8233591610	-2.3650534381
H	3.4252089364	-1.2199148601	-0.8703035415
N	1.8423267634	-9.0324795390	-4.9461612106
H	1.0485475805	-9.2767877720	-4.3424525114
H	2.6650745396	-9.5802187272	-4.6681278242
H	2.0494591116	-8.0313637277	-4.8507733345
H	1.6062250490	-9.2415481670	-5.9232905845
N	-0.3342027485	-7.9962908153	-2.8295540976
O	0.8270992144	-7.8863399321	-2.4025804859
O	-1.2578357056	-7.3681372447	-2.2860992575
O	-0.5718719457	-8.7343958632	-3.7999833307
N	-2.4899106621	-4.5115198218	-5.9232674805
O	-2.3056391713	-4.8917121283	-7.0913923620
O	-2.1520138334	-3.3658114990	-5.5823920149
O	-3.0120794020	-5.2770364544	-5.0960173988
N	5.8029225595	0.1691903652	-4.0060961081
O	5.1089249308	0.2528294754	-2.9792613105
O	5.2913323123	0.3719787508	-5.1197254003
O	7.0085114059	-0.1172373612	-3.9193015435
N	3.9742914701	-3.7499533954	-4.6486033769
O	4.7095756845	-2.9051901617	-4.1112486392
O	3.0047503051	-3.3837633018	-5.3333753503
O	4.2085486094	-4.9609076975	-4.5011860226
N	5.8556839909	-4.5856058217	-1.6541358608
O	5.1315610875	-5.5801703673	-1.4823648029
O	6.9891802296	-4.7198006026	-2.1442337577
O	5.4463103260	-3.4568455866	-1.3358087655
N	0.8022366703	-5.6911137153	-5.3785264534
O	1.8133314021	-6.3840005666	-5.1769993694
O	0.7813007907	-4.8823461423	-6.3211157663
O	-0.1879229789	-5.8069945302	-4.6374636280
N	1.7649325675	-2.4814619330	-8.3151425988
O	2.7921728152	-3.1727342173	-8.4148321843
O	1.7756398614	-1.4490902443	-7.6244096828
O	0.7269841904	-2.8225616119	-8.9061864051
N	6.5467384001	-4.3705303683	-6.5574953595
O	7.5114160372	-4.4925204269	-5.7844925179
O	5.9363423007	-5.3776118146	-6.9527439817
O	6.1924565772	-3.2414579546	-6.9352498829
N	1.0323605463	-2.9452152020	-2.6329367998
O	-0.1784108907	-3.2001267434	-2.7428028287
O	1.5079743423	-1.9395180750	-3.1855512375
O	1.7675187791	-3.6960013920	-1.9704558000
N	0.6423419533	-7.8042936786	-7.8910265649
O	-0.5835289603	-7.6281392512	-7.7949639196
O	1.3498205742	-6.9531188952	-8.4549356000
O	1.1607346632	-8.8316237166	-7.4231797985
N	4.7856092254	-8.9423926183	-4.1521391660
O	6.0275949128	-8.9365251477	-4.1308075929
O	4.1723830248	-10.0091510315	-4.3224272326
O	4.1568492325	-7.8815008217	-4.0031825525

n=12 OPLS/AA

N	1.2948795533	-1.9091976253	-0.1148501885
H	1.4454639976	-2.2303631264	0.8486917896
H	1.7452871121	-0.9957978439	-0.2454968718
H	0.2874503465	-1.8226731415	-0.2932750498
H	1.7013169037	-2.5879567021	-0.7693196836
N	2.5670701704	1.5320358518	-5.1558448990
H	1.8603631810	0.8385060832	-5.4275391916
H	3.4924091559	1.2201309752	-5.4731740581
H	2.5719023497	1.6335482069	-4.1341270241
H	2.3436053067	2.4359574668	-5.5885395868

N	-3.0708752084	-4.3342627892	1.7780334490
H	-2.1178609972	-4.2301518060	2.1456758215
H	-3.2955131540	-5.3320067320	1.6870416718
H	-3.1336628921	-3.8839627645	0.8574232609
H	-3.7364628620	-3.8909297530	2.4219934000
N	0.2726310791	-7.4405546519	-0.2804516939
H	0.4298188226	-6.5492545739	0.2044320878
H	-0.5365690814	-7.9176253652	0.1340761342
H	1.1053788713	-8.0335721354	-0.1850740088
H	0.0918958569	-7.2617656652	-1.2752405166
N	1.5324080002	-4.7496473333	-5.2431682578
H	2.3660746584	-4.9039881925	-5.8223184108
H	0.8219545339	-5.4550129395	-5.4710935072
H	1.1559110035	-3.8124788006	-5.4281025081
H	1.7856926168	-4.8271095509	-4.2511591692
N	-1.9225730298	-5.1499570833	-2.4016899791
H	-2.4304898276	-4.2694072524	-2.5462189362
H	-1.8335290553	-5.6446308435	-3.2970132319
H	-2.4383997694	-5.7373104314	-1.7359765699
H	-0.9878739614	-4.9484789482	-2.0275513192
N	3.5640358141	-4.8810742145	1.6012080860
H	3.9686572265	-5.0474247331	2.5301026522
H	2.6918510865	-4.3480569537	1.6982703653
H	3.3665901361	-5.7808730891	1.1477627049
H	4.2290452014	-4.3479422442	1.0286975262
N	4.9723372967	-4.7742350618	-2.3698332521
H	4.0886708593	-4.2994359358	-2.5887750589
H	5.5357845530	-4.8595551175	-3.2239306488
H	4.7746487500	-5.7096117203	-1.9953666055
H	5.4902441641	-4.2283370114	-1.6712609085
N	4.7267920069	-2.2468611987	-5.8288688643
H	3.9877035072	-1.8227427732	-6.4016758547
H	5.4706219462	-1.5582481034	-5.6652726795
H	5.1156377799	-3.0606145974	-6.3196232783
H	4.3332040743	-2.5458389079	-4.9289042023
N	3.0187622331	-8.3482500942	-3.7626998350
H	3.3278597180	-9.2784964098	-4.0682102848
H	3.6838981059	-7.6402656894	-4.0952461307
H	2.9750049911	-8.3218165963	-2.7372135611
H	2.0882864186	-8.1524225875	-4.1501296612
N	-0.4984258546	-0.8752102174	-3.4265794988
H	-0.3876545284	4.454271E-40	-2.9019910857
H	-0.5402499994	-0.6691554032	-4.4315804142
H	-1.3663415398	-1.3406729391	-3.1362308425
H	0.3005427569	-1.4914571014	-3.2365151419
N	4.7650864096	0.3660150015	-1.6588773567
H	5.0105149555	0.7385615109	-2.5836530185
H	3.7540656946	0.4628102832	-1.5082039594
H	5.2707291184	0.8887566421	-0.9341000445
H	5.0250361091	-0.6260680675	-1.6095533050
N	2.7242313262	-2.0488107343	-3.3971008830
O	3.1583509572	-0.9085636977	-3.6303277091
O	1.6164200468	-2.1925412029	-2.8538572612
O	3.3979235168	-3.0453281046	-3.7071179283
N	1.6895782952	1.1336341811	-1.8850792890
O	2.1676642123	0.4538606209	-0.9618460175
O	2.4336747462	1.8377241819	-2.5876429184
O	0.4673949432	1.1093177209	-2.1057491088
N	-2.7538968627	-7.1948290432	0.6143621466
O	-3.4513319420	-6.8702859518	1.5896949118
O	-2.8007794770	-6.5274420214	-0.4322583646
O	-2.0095785698	-8.1867599549	0.6856499500
N	3.3985333918	-7.5529571238	-0.7048931842
O	3.1940770191	-7.1324975999	0.4459453324
O	2.6600644523	-8.4278327129	-1.1868415631
O	4.3414594632	-7.0985406928	-1.3737838604
N	1.6919240738	-4.8214663695	-1.7631065676
O	2.1294138765	-5.4248153337	-2.7568825036
O	2.4768170451	-4.2505818124	-0.9878312894
O	0.4695403158	-4.7890019362	-1.5446057341
N	-0.0645891264	-7.0789282967	-4.0669295655
O	-0.2056771685	-7.4402681891	-2.8868678902
O	-0.7866709942	-6.1816905946	-4.5323113366
O	0.7985814783	-7.6148265380	-4.7816100449
N	5.5468956975	0.5312225138	-4.9334431312
O	4.8696400750	0.7078669295	-5.9596689753
O	5.4259003854	1.3043299032	-3.9687239960
O	6.3451472745	-0.4185300560	-4.8719363727
N	0.4387390251	-4.3143952244	1.9422553732
O	0.5796707030	-5.4099290709	1.3739581233
O	-0.6520781290	-4.0175292943	2.4570431613
O	1.3886252660	-3.5157266650	1.9957648780
N	1.2279024450	-1.3281513689	-6.0294720164
O	0.6257951471	-2.3694886186	-5.7194696856
O	2.3286265451	-1.3939711574	-6.6013916976
O	0.7292852414	-0.2209934394	-5.7675544551
N	4.6913661966	-5.4227663776	-5.3472697484
O	5.6655922442	-4.7583329647	-4.9568472095
O	4.4556299361	-6.5338263762	-4.8442866414
O	3.9528758149	-4.9761394324	-6.2406761137
N	-2.1637197403	-2.4510677593	-1.2017952508
O	-2.9249167427	-3.0955245155	-0.4613409822
O	-1.2358571313	-1.7864898335	-0.7114636586
O	-2.3303854811	-2.4711889451	-2.4325821024
N	4.9561616467	-2.5437534832	-0.2786573208
O	4.1546473149	-1.8528415332	0.3719065609
O	5.4140180430	-2.1184162463	-1.3521905836
O	5.2998198588	-3.6600035688	0.1443124006

n=13 OPLS/AA

N	4.3049867032	1.7415774216	0.8383742987
H	4.7463956605	0.8181257839	0.7569414866
H	4.9932232821	2.4706260787	0.6168948358
H	3.9627469268	1.8739657026	1.7973220567
H	3.5175813733	1.8035912218	0.1823387362
N	6.9483814021	9.0590069969	4.6015678352
H	6.8262877795	8.0413252029	4.6620079805
H	6.5988760772	9.3924343276	3.6955286923
H	7.9448989633	9.2900121099	4.6900263676
H	6.4234626694	9.5122553560	5.3587083592
N	0.5814432105	2.6554226910	2.8435193651
H	1.1191278446	1.8220379872	3.1092283547
H	1.0207644336	3.4899574912	3.2494543545
H	-0.3794309056	2.5692083701	3.1949785349
H	0.5653119929	2.7404861040	1.8204164750
N	-0.5370058637	5.0551928358	-0.2879162183
H	-0.2835742281	4.2243298295	-0.8353421662
H	0.1523588774	5.1956832842	0.4599296034
H	-1.4676224564	4.9222207144	0.1250083469
H	-0.5491854010	5.8785367057	-0.9012611903
N	5.4631616909	4.8058710738	2.7303434756
H	5.4312435049	5.8310698371	2.7770855629
H	5.9463278076	4.5172942683	1.8715602571
H	4.5063171541	4.4334895657	2.7263348687
H	5.9687582660	4.4416316225	3.5463932593
N	4.0186947639	3.4700093294	6.3488623253
H	5.0054138845	3.4855527738	6.6323757746
H	3.8707508365	4.1426928269	5.5873873145
H	3.4284651810	3.7242579517	7.1496258370
H	3.7701501144	2.5275337804	6.0260606511
N	1.5139632144	9.7365445127	8.2628822208
H	2.5005110329	10.0196486991	8.2343950500
H	1.0605934373	10.1575961552	9.0822759265
H	1.4531510212	8.7135354714	8.3260478976
H	1.0415983268	10.0553980007	7.4088099813
N	4.2849675276	7.9283799577	0.1073541021
H	3.5919481335	7.8813177610	-0.6487843552
H	4.7832006075	8.8248862548	0.0596577414
H	4.9560174013	7.1580203781	0.0050129610
H	3.8087032930	7.8492953912	1.0135293249
N	2.9199530053	10.7102451936	4.1284030754
H	3.3569945696	11.1877114900	3.3313731485
H	3.1242648792	11.2277478597	4.9913520097
H	3.2946984589	9.7573035615	4.2039253561
H	1.9038509390	10.6682183282	3.9869610112
N	2.9331613501	5.0184980115	-2.5667228383
H	3.8301110322	4.7274989288	-2.1604755007
H	2.9980703102	4.9959836541	-3.5911810444
H	2.7113948268	5.9725360165	-2.2587185880
H	2.1930701049	4.3779731631	-2.2565158244
N	-0.6629334055	6.0758760006	5.4884701923
H	-1.0988988279	7.0054476269	5.4802946470
H	-0.5611443882	5.7541070504	6.4581806770
H	0.2622391037	6.1278524739	5.0462148379
H	-1.2539299343	5.4160977564	4.9691905994
N	4.2336820569	7.1672447398	6.8605222608
H	3.4161870885	6.6446200558	7.1963816271
H	5.0733294967	6.5829007791	6.9486641803
H	4.3492858364	8.0193616913	7.4215641409
H	4.0959250098	7.4220959239	5.8754794222
N	0.5364255951	7.8363226447	2.3543403813
H	-0.2675155159	7.2018674367	2.4276982487
H	1.3526095593	7.4083231922	2.8069935494
H	0.3145555352	8.7249890575	2.8183244408
H	0.7460520187	8.0101102745	1.3643453579
N	5.6415371690	4.6064489652	-0.4285057690
O	5.1840780693	4.3289341037	-1.5495475098
O	5.7917481712	5.7936981622	-0.0954983969
O	5.9487855140	3.6967138972	0.3595292342
N	-1.7016592821	4.9230014369	2.6991114313
O	-2.1110887188	4.3437605585	1.6793734983
O	-1.5851973435	4.2959886076	3.7650895923
O	-1.4086915482	6.1292561158	2.6528711661
N	4.4035656423	10.2765742803	7.0209841435
O	3.5795899098	11.1308202615	6.6544521438

O	4.1467594681	9.5407538259	7.9882673277
O	5.4843484191	10.1581486583	6.4202324752
N	2.3718850888	4.8962039876	1.2478076338
O	3.0511176684	4.0890338142	1.9036561871
O	2.8436828919	5.4166027652	0.2233029455
O	1.2208537795	5.1829756144	1.6164640655
N	1.3345474725	7.4899819946	-1.2541574934
O	0.3927497461	6.9246226039	-1.8341606939
O	2.4639162520	7.5037283588	-1.7712214239
O	1.1469762685	8.0415954653	-0.1570894792
N	0.0266089533	9.6357851051	5.0530195490
O	-0.7616655666	8.6919267257	5.2284406190
O	0.4471297506	10.2828871832	6.0263843679
O	0.3943629720	9.9325416453	3.9042327355
N	2.2715435129	5.4625321025	4.5750678559
O	3.5055722096	5.4022111870	4.7037242733
O	1.6005319794	4.4183813491	4.5249615980
O	1.7085258964	6.5670046606	4.4965176333
N	1.3880443606	2.6513063967	-0.7832968489
O	0.6757833321	2.5519803370	0.2295390075
O	1.0592153765	3.4214890564	-1.7007433492
O	2.4291352113	1.9804492568	-0.8786862818
N	3.4585108035	1.5195269738	3.9114798119
O	4.4632382327	2.1523433792	3.5466728754
O	2.6768231106	1.0539238262	3.0657872137
O	3.2354708875	1.3523135815	5.1219803212
N	6.4982480195	5.4180203288	5.7529868182
O	6.0519647013	5.3086222980	6.9070583851
O	6.8319736521	6.5331454664	5.3192345381
O	6.6108057959	4.4122924122	5.0326669515
N	1.5141962011	5.9780208921	7.8798770686
O	1.6719953048	7.1841880066	8.1314287446
O	2.5020497169	5.2463388181	7.7015383885
O	0.3685426593	5.5035354696	7.8066640138
N	4.1917872095	7.9421176411	3.3214876333
O	5.2280350117	7.3635070856	2.9548567177
O	3.2521138201	8.0978545435	2.5241308440
O	4.0952127188	8.3649916348	4.4854762754
N	5.1900965732	10.6538671125	1.7141545172
O	5.2876737300	10.2695569363	0.5369534733
O	6.0268888425	10.2908911927	2.5573894137
O	4.2557263950	11.4011538100	2.0481209335

n=14 OPLS/AA

N	1.2548395604	0.3760800489	2.6246823564
H	2.1670034114	-0.0311214417	2.3872373047
H	0.8111370102	-0.1845623396	3.3615967643
H	0.6552293390	0.3819047151	1.7912141927
H	1.3859893694	1.3380988652	2.9586809324
N	2.6512459994	7.0032441419	2.2109311373
H	2.5987516653	6.0035327318	2.4390923500
H	3.6336632861	7.2749931447	2.0874668116
H	2.2380946636	7.5508183238	2.9749363815
H	2.1344743314	7.1836313937	1.3422292283
N	3.8508411591	0.5091987642	-0.3838726822
H	4.1584473028	-0.3439020085	0.0976183661
H	2.9819641375	0.3184383501	-0.8966083559
H	4.5778876808	0.8112943026	-1.0429456411
H	3.6850658151	1.2509635818	0.3064453711
N	-2.8730774561	-0.4930690242	5.2594243130
H	-2.8583893992	-0.3234912378	4.2468712364
H	-1.9732296776	-0.8910546808	5.5528797238
H	-3.6282689463	-1.1510092556	5.4853357758
H	-3.0324217870	0.3932792427	5.7526095300
N	-2.3751167307	1.9388212603	0.4565637142
H	-1.8812488452	1.6246355336	-0.3870107865
H	-1.7306113770	1.9247073415	1.2557184751
H	-3.1661226495	1.3115020901	0.6436888572
H	-2.7224835702	2.8944397702	0.3138574896
N	-1.8116507013	5.2012533453	4.9638002899
H	-2.4537860063	4.7599129000	5.6324683384
H	-2.2979137908	5.3547593721	4.0726107517
H	-1.0022818556	4.5882429217	4.8109087719
H	-1.4926217780	6.1020977574	5.3392139488
N	3.4834363101	6.1011133653	5.7736313300
H	4.1619930793	5.9808594656	5.0124902076
H	2.6841785117	6.6501609471	5.4360392535
H	3.9305108430	6.5930454440	6.5561674097
H	3.1570634673	5.1803874874	6.0898277077
N	4.8076293844	3.0795022618	3.1559794641
H	5.4089016896	3.8266808167	2.7893298772
H	4.8032491902	3.1162251951	4.1820725036
H	5.1671959482	2.1685273122	2.8476419032
H	3.8511712950	3.2065764508	2.8048732156
N	3.5784524948	-0.6532578115	5.9479003737
H	2.6316049928	-0.9096490430	5.6446192467
H	3.5668196308	0.2951361477	6.3411538035
H	4.2152523702	-0.6822222076	5.1429888838
H	3.9001320633	-1.3162963929	6.6628392654
N	4.9632928554	5.6541922603	-0.6538729119
H	4.3135638127	6.2401833725	-1.1911835159
H	5.6306266846	6.2530901201	-0.1536513616
H	5.4731471563	5.0334441757	-1.2933695354
H	4.4358331351	5.0900519434	0.0227122420
N	-0.4018850730	5.4563874571	1.2055814736
H	0.1827721715	4.6141538333	1.1503136983
H	-1.3855597962	5.2003012046	1.0605347376
H	-0.1112745345	6.1229290563	0.4806653850
H	-0.2934775634	5.8881649140	2.1308120199
N	1.3461268415	3.4208973080	-2.4000619895
H	1.0991595052	4.3490476919	-2.0370467041
H	1.1969899767	3.3998378400	-3.4157141838
H	0.7555204488	2.7101222272	-1.9526042849
H	2.3328371946	3.2245823771	-2.1948824316
N	0.4424344542	2.2123505744	6.6402075599
H	1.2299863509	2.8453380286	6.8227588961
H	0.2796605053	2.1515500814	5.6282576466
H	-0.4008689399	2.5733970100	7.1014237574
H	0.6609606675	1.2791177939	7.0083901174
N	-5.2071231871	2.9173373792	4.1153132835
H	-4.5841419467	3.1653660888	4.8928814355
H	-4.9459313696	3.4571967008	3.2819073602
H	-6.1776222102	3.1326132277	4.3722636273
H	-5.1207966150	1.9141737411	3.9142014682
N	0.4988274682	7.2370567350	4.4913933386
O	1.6809702603	7.5966935963	4.6187419068
O	0.0766213566	6.8726546364	3.3814514388
O	-0.2611098241	7.2418219759	5.4739874611
N	3.5165762760	2.9631545390	6.4320887929
O	2.6606178393	3.7782177065	6.8142210982
O	4.4662583798	3.3441097163	5.7278196927
O	3.4228525333	1.7671352316	6.7542258471
N	2.5211934640	3.5038510644	0.8724761403
O	2.9576379251	2.3818483909	1.1784894473
O	1.3402771456	3.6331131270	0.5095079890
O	3.2656659207	4.4965924744	0.9294310306
N	4.6212293140	-0.1666488403	2.7046972978
O	4.9489925201	-0.5933252070	3.8243117269
O	5.2916259439	0.7256822450	2.1594161742
O	3.6230686743	-0.6323039338	2.1303635300
N	0.4863925197	-0.9656683260	5.9543747852
O	-0.6466267400	-1.4733910799	5.9931416947
O	1.1822246581	-1.0770246456	4.9314205750
O	0.9235799929	-0.3465887540	6.9385628782
N	-3.4178066273	4.7479489967	1.7981963337
O	-4.5281214551	4.2380313636	2.0222563737
O	-2.8954431921	5.4945181239	2.6424645912
O	-2.8298547615	4.5112973121	0.7298671760
N	-1.3278083630	2.1219904021	3.5509298304
O	-0.7810809320	2.3396632802	2.4569803136
O	-2.4395588039	2.6173935609	3.7991325263
O	-0.7627848982	1.4089137912	4.3966773321
N	-4.1370777560	0.3043548524	2.5091950740
O	-3.0314083221	-0.2388783731	2.6686023405
O	-4.9099156418	0.4343852405	3.4729541783
O	-4.4699095719	0.7175580225	1.3860277988
N	1.6107205822	3.8107913143	4.0089186589
O	2.3298889540	4.7082732029	3.5395136911
O	0.5476235390	4.1024682200	4.5814281963
O	1.9546495306	2.6216315628	3.9058140063
N	5.0746903652	2.8393485517	-2.0413003363
O	5.4809082789	1.6657690187	-2.0145422123
O	3.8584194400	3.0776486248	-1.9581377094
O	5.8847440288	3.7746287646	-2.1512211757
N	0.3873120050	0.8869047352	-0.8135875096
O	1.4642534898	0.6117832615	-1.3681351848
O	0.0988170771	0.3652311907	0.2762038927
O	-0.4011351865	1.6837003947	-1.3488316675
N	-3.0320138776	2.8393581979	6.6170438498
O	-1.9378032614	3.1102703258	7.1388940749
O	-3.7357143873	3.7466739037	6.1431222174
O	-3.4225242984	1.6611294158	6.5691152185
N	1.6203096622	6.5484757590	-1.0044698223
O	2.7425198985	6.6933761346	-1.5169793125
O	0.7957179561	7.7774147068	-1.5226698639
O	1.3226908923	7.1746369395	0.0262405392
N	5.4678054148	6.0917595256	2.5185145279
O	5.0692748140	6.1258133881	3.6945381695
O	6.0899477839	5.1005555571	2.1020154566
O	5.2441934666	7.0489104021	1.7589893461

n=15 OPLS/AA

N	1.9001499226	-2.3569429068	2.6631417861
H	2.8127677398	-2.8208636443	2.5847685334

H	1.4256894414	-2.6768342241	3.5156624500
H	1.3263373250	-2.5899028064	1.8441763949
H	2.0358060731	-1.3401714042	2.7079596899
N	-0.8905565250	-0.7693560555	0.7452442715
H	-1.3160295000	-0.1701373449	0.0282046757
H	-0.0609593317	-0.3040648130	1.1319059349
H	-0.6157907962	-1.6645831482	0.3241649787
H	-1.5694468865	-0.9386383325	1.4967007985
N	4.6573304459	-3.3826554968	-1.2091224269
H	5.0415744228	-3.8486888176	-2.0394269540
H	4.9044971074	-2.3863239754	-1.2307285914
H	5.0478736568	-3.8138993104	-0.3631080667
H	3.6353769707	-3.4817103378	-1.2032269041
N	2.6000007159	-1.6025730455	8.1924279022
H	3.4919460346	-1.8727695680	8.6233147613
H	2.7748108225	-1.2160001621	7.2574212081
H	2.1392165630	-0.8938091690	8.7751518985
H	1.9940303124	-2.4277135462	8.1138241604
N	8.3332412126	-2.6106653865	1.9299654365
H	8.6474289058	-1.8539934837	2.5488109927
H	8.0085163136	-2.2130202414	1.0407709898
H	7.5634511952	-3.1216803094	2.3777629375
H	9.1135687416	-3.2539667747	1.7525174287
N	-0.2084563793	2.2387036957	5.0034238992
H	-0.0363154311	2.3391848919	6.0106513416
H	-1.0357398507	1.6490152769	4.8547487092
H	0.6074849760	1.8020744044	4.5586429524
H	-0.3692550440	3.1645403077	4.5896535746
N	3.7223875509	2.1349532016	7.2847878689
H	3.2658348550	1.8607310970	6.4069507707
H	4.4139977273	1.4230923774	7.5477787742
H	3.0169571167	2.2171881723	8.0263004016
H	4.1927600598	3.0388008927	7.1581206739
N	-1.0592408893	-1.5121088451	5.8199195837
H	-1.4949939285	-0.9324630930	5.0930315673
H	-0.5949221300	-0.9071530416	6.5074304934
H	-0.3664995242	-2.1363954960	5.3902556421
H	-1.7805483988	-2.0724231854	6.2889599241
N	3.4599997563	3.8157111894	3.2496268175
H	3.7768695925	4.3721947000	2.4470337033
H	4.0410673951	4.0332532297	4.0677163880
H	3.5422822722	2.8166971431	3.0272846333
H	2.4797800742	4.0407002268	3.4564717638
N	7.4652254862	0.7543365157	5.7397065125
H	7.2433447804	1.6358284646	6.2171758770
H	7.4819512255	-0.0108423780	6.4241451114
H	8.3848380395	0.8310064963	5.2895176744
H	6.7507676834	0.5613543385	5.0279878523
N	-2.5406802780	3.1650089827	1.6033283474
H	-2.6701814108	2.1881573905	1.8918184184
H	-2.0868999024	3.1927054690	0.6827030378
H	-1.9510281054	3.6494453009	2.2902543209
H	-3.4546118197	3.6297268190	1.5485378937
N	7.1439704320	2.1037239618	1.9778054484
H	6.7685573077	2.3191779195	2.9088697189
H	6.7685491796	2.7717590463	1.2944148613
H	6.8705825679	1.1507655565	1.7106633934
H	8.1681923073	2.1731935347	1.9972747268
N	3.6241135034	0.6905125599	0.1127474324
H	3.5378081465	1.5900228210	-0.3747654117
H	4.4299983769	0.1750451592	-0.2601836804
H	2.7694244701	0.1399681900	-0.0309295568
H	3.7592229359	0.8570149454	1.1168679037
N	4.9289956855	-2.3667114986	4.9041885121
H	4.0981537622	-2.8376261223	5.2812524500
H	5.4255424096	-3.0039603480	4.2704772685
H	5.5505184086	-2.0988008035	5.6763082490
H	4.6417673523	-1.5264591794	4.3887164480
N	1.3475594968	4.2530984638	-0.3294603030
H	0.9132751194	4.5332196610	0.5577630578
H	2.3583902993	4.1349571525	-0.1934514620
H	1.1798298791	4.9783666912	-1.0366295174
H	0.9387422666	3.3658506232	-0.6455224264
N	-0.8820249636	2.1063055580	-0.8779917911
O	-1.3346657378	3.2460985504	-0.6804919807
O	0.3257080357	1.9516553174	-1.1239231157
O	-1.6371177965	1.1211620133	-0.8295602378
N	6.3829126774	-0.9966773177	-0.1162736726
O	7.2364801742	-1.8845864224	-0.2776981476
O	5.4231512949	-0.9079538308	-0.8998578369
O	6.4891066486	-0.1974910566	0.8287357274
N	9.1527064303	0.1223995924	3.2253033822
O	9.2189808111	1.2045361387	3.8316086786
O	9.2872826664	0.0928898182	1.9907847226
O	8.9518556516	-0.9302280270	3.8535172510
N	2.2386476290	0.2097634781	5.2198793240
O	1.2618228240	-0.3254296940	4.6699707134
O	3.0229355527	-0.4756281644	5.8967461203
O	2.4311846651	1.4303492753	5.0929210359
N	1.0103895448	1.4087230171	1.8742380610
O	1.8497531445	2.2982309833	1.6568392650
O	-0.1391730230	1.7137430796	2.2326883016
O	1.3205887627	0.2141940268	1.7331865029
N	1.4660458736	-3.2948292482	5.8633248290
O	0.9588615160	-3.2725288005	6.9970287712
O	2.6963365313	-3.4090635823	5.7354447085
O	0.7429389912	-3.2028952878	4.8575001977
N	1.6745000039	-2.6503788188	-0.3936013995
O	0.6868411033	-2.8322801107	0.3374661261
O	2.1038515949	-3.5778389226	-1.0996416803
O	2.2328077630	-1.5410165301	-0.4186286644
N	4.7072095186	0.3278209541	3.1761672874
O	5.6610116419	0.2890920869	3.9710128669
O	4.4325870522	1.3861464559	2.5866356562
O	4.0280293148	-0.6917765013	2.9708531737
N	0.8094598496	1.0080570986	7.9245068030
O	0.1071787056	0.0506527529	7.5595464976
O	0.5981339127	2.1433607747	7.4668553828
O	1.7230676661	0.8301576250	8.7471191907
N	0.0235876439	4.5837960159	3.0160268288
O	1.0213335026	4.4784143622	3.7484309655
O	-1.1007882211	4.2850043462	3.4513661945
O	0.1502177522	4.9879696647	1.8482823864
N	-2.3760855008	0.1075263273	3.3855889476
O	-2.1772848991	0.5928977589	4.5116044026
O	-2.9335029564	0.7855094729	2.5065880685
O	-2.0174683583	-1.0558291866	3.1385741727
N	5.3439668260	-3.8388298266	2.1214390161
O	4.1435356308	-3.6313520083	2.3642060063
O	6.1958137581	-3.6673687245	3.0091190293
O	5.6925513697	-4.2177690520	0.9909911028
N	5.6351086346	-1.0090147817	7.7774632591
O	5.1383604504	-1.8034299280	8.5930389623
O	6.3941995153	-1.4264647553	6.8872192109
O	5.3727657271	0.2028513139	7.8521316642
N	5.7936904609	3.3878561792	5.4274623532
O	4.7257039119	4.0168500392	5.5098599638
O	6.3603556953	2.9884139756	6.4581681061
O	6.2950121791	3.1583043381	4.3143580937
N	4.4647437978	3.8218461525	0.1847257115
O	3.9094616325	4.7330139920	0.8206917453
O	5.6521492858	3.5384267435	0.4144201965
O	3.8326199660	3.1940972166	-0.6809355042

n=16 OPLS/AA

N	-5.2619894830	0.3085116058	-2.4982901768
H	-4.3644836633	0.2318650688	-2.9910829422
H	-6.0283481947	0.0968761102	-3.1480127540
H	-5.3735253809	1.2630322517	-2.1367855377
H	-5.2815998189	-0.3577270822	-1.7172799531
N	-9.0748514579	-0.7290034877	-0.1427672336
H	-9.5892402499	0.1217448087	0.1138796139
H	-8.2350783390	-0.8108157155	0.4423161395
H	-8.7999968909	-0.6778388374	-1.1307311346
H	-9.6750908530	-1.5491033782	0.0034666966
N	-8.3189540914	5.1933410521	1.8795094805
H	-8.6601412174	6.1583917122	1.7988579703
H	-9.0858160102	4.5411913212	1.6773753381
H	-7.9738253392	5.0308881917	2.8327831750
H	-7.5560341311	5.0428939231	1.2090213600
N	-3.3388380226	6.8501030234	-3.9086162428
H	-3.9097746837	6.3195418624	-3.2402059788
H	-3.5003646889	6.4914281625	-4.8570339596
H	-2.3459738744	6.7469367546	-3.6681728434
H	-3.5992393994	7.8425047975	-3.8690515383
N	-3.0070077637	3.2123447768	-4.4940024733
H	-2.1734186524	3.8080143279	-4.5614048242
H	-2.7281536684	2.2257874391	-4.5503912887
H	-3.6476464850	3.4315825583	-5.2658524251
H	-3.4788114371	3.3839953619	-3.5983614211
N	-8.1523177578	7.5034378971	-1.6258453472
H	-9.0037586715	7.2636784921	-2.1471896631
H	-7.6920265968	8.3053338922	-2.0723087227
H	-7.5138385375	6.6993367889	-1.6247421167
H	-8.3996480547	7.7454021819	-0.6591413939
N	-1.4639762881	6.0784974465	-0.2579149704
H	-0.9259029686	6.3397627092	-1.0924529642
H	-2.3242500651	5.5957000575	-0.5426436047
H	-1.7054589786	6.9240277348	0.2721795172
H	-0.9002926159	5.4544995391	0.3312563572
N	-7.2259132277	3.2373025352	-4.6896961441
H	-6.6458046066	3.3598661855	-5.5279611600
H	-8.1021983071	3.7608617725	-4.8003526857
H	-6.7190873174	3.5879525188	-3.8684729042
H	-7.4365621149	2.2405297835	-4.5619986430

N	-9.4639583304	3.5544034991	-1.7625477958
H	-9.7709275298	4.3911468262	-2.2723189334
H	-8.4812609998	3.6635807029	-1.4857428460
H	-9.5595231402	2.7305830841	-2.3678764792
H	-10.0441219505	3.4323041982	-0.9242534211
N	0.6703810179	2.4305743004	-1.9694577477
H	0.4026865538	2.2925321091	-0.9878671381
H	0.6090354335	3.4283319713	-2.2038741185
H	0.0361234856	1.8969983304	-2.5754689613
H	1.6336783380	2.1044346563	-2.1106198169
N	-6.4947925355	2.0245625939	2.4112001747
H	-6.2061051262	2.4445379843	1.5198437092
H	-6.0993735913	2.5661921282	3.1887064394
H	-6.1542217984	1.0571580351	2.4599394484
H	-7.5194693449	2.0303626369	2.4763102338
N	-4.8251524897	8.5095293985	-0.4412146495
H	-4.7544741591	8.9127907623	-1.3828201634
H	-4.9281378166	7.4904168234	-0.5121954016
H	-5.6425230241	8.9034777930	0.0393533437
H	-3.9754748903	8.7314326082	0.0908027061
N	-5.5156486024	8.0321045740	4.1478063353
H	-6.3916340150	8.0992538296	3.6164151122
H	-4.7347866217	8.3506586786	3.5621336153
H	-5.3584808606	7.0565401552	4.4267469918
H	-5.5776937655	8.6219656980	4.9859291045
N	-3.1213237426	4.4855416781	2.6717321692
H	-3.7876725748	4.4775094787	1.8906106581
H	-3.6148410973	4.2657807893	3.5448761767
H	-2.6920938093	5.4153253841	2.7458904359
H	-2.3906881380	3.7835510525	2.5055506454
N	-7.3349422898	7.5664859923	-6.1107476241
H	-7.6542103500	8.0658090186	-6.9491869593
H	-8.1398830164	7.3614997067	-5.5071867450
H	-6.6636078550	8.1510924593	-5.5991023568
H	-6.8820682487	6.6875432711	-6.3875152519
N	-2.9880374366	0.9733983385	0.3528914902
H	-3.4338426803	1.8363154733	0.0199233223
H	-3.6827911320	0.3951573068	0.8399123291
H	-2.2256842609	1.2093058603	0.9989571221
H	-2.6098321075	0.4528155540	-0.4472271370
N	-9.5239321431	5.8999424546	-3.9943716462
O	-10.0201451292	5.8733716614	-2.8559145428
O	-9.3118165855	4.8388417298	-4.6043635748
O	-9.2398344859	6.9876148483	-4.5228372466
N	-0.5021480890	5.4353181850	-3.2144267531
O	0.3754156846	4.8968962577	-2.5194436149
O	-0.9180873611	6.5685219287	-2.9214050676
O	-0.9637729621	4.8405358899	-4.2024323722
N	-5.5705227005	9.0987790733	-3.5255378112
O	-5.6381608102	8.8896963911	-4.7481282697
O	-6.6107018122	9.2319025046	-2.8597123997
O	-4.4627045874	9.1747383854	-2.9687723161
N	-2.0575244604	0.5648560201	-3.0510084026
O	-3.0177337950	0.4926549098	-3.8357395313
O	-1.0247985699	1.1678544618	-3.3869965355
O	-2.1300410746	0.0340582615	-1.9302882388
N	-3.2452964318	3.6528141185	-1.3520892541
O	-2.9864384256	4.6964178006	-1.9741035613
O	-3.0789478042	3.6054379266	-0.1220073294
O	-3.6705034078	2.6565858263	-1.9601573612
N	-0.6617408244	2.9368746269	1.2092954644
O	-1.4672595130	2.5406759418	2.0678897547
O	-0.1395031897	2.1260013761	0.4264989806
O	-0.3784595424	4.1439475344	1.1334975970
N	-5.7672833806	-0.6470806104	0.9109483862
O	-5.4214289554	-0.9972082766	-0.2295836146
O	-4.9299549566	-0.1704404671	1.6949848618
O	-6.9504671824	-0.7735931895	1.2674441984
N	-5.0687471924	4.8815557552	-6.3237769971
O	-4.0983427970	5.6245405785	-6.1017257136
O	-4.9450384756	3.6500590368	-6.2182786707
O	-6.1628611855	5.3700680436	-6.6513268707
N	-8.3242365003	0.5334260170	-3.3116432099
O	-9.2227778148	1.3864253069	-3.2220126989
O	-8.3295164645	-0.4555761577	-2.5600683757
O	-7.4204144941	0.6694290111	-4.1528492323
N	-9.8189672910	2.4202079618	1.1769686500
O	-9.0942916791	2.2051428088	2.1626715202
O	-10.1982138094	1.4743133158	0.4667058056
O	-10.1643966626	3.5811686952	0.9015284023
N	-7.8110311479	8.2950701693	1.4778669412
O	-7.0632629002	9.0325092957	0.8145087473
O	-8.7657115917	7.7217963056	0.9274487015
O	-7.6041187854	8.1309047743	2.6916443519
N	-5.9801968644	5.4548423260	-2.7858308838
O	-5.3108973463	4.6900602915	-3.5000888943
O	-7.1645393329	5.1847223300	-2.5262427177
O	-5.4651534994	6.4897451898	-2.3311606733
N	-5.7849657649	4.6067992618	4.3180272961
O	-5.0906206072	5.5745130379	4.6707775765
O	-5.2663154177	3.4859592321	4.1848456303
O	-6.9979622463	4.7599256387	4.0984585045
N	-5.3802146768	5.5891762885	0.7191771896
O	-6.3958539072	6.1285786996	0.2495604271
O	-4.2551127902	6.0385924206	0.4449714353
O	-5.4896774213	4.6003569493	1.4630003054
N	-6.5949818864	2.6078023955	-0.7197422382
O	-6.7171826450	2.4945022918	-1.9506963283
O	-7.3970201606	3.3059697832	-0.0776247906
O	-5.6707421096	2.0229346408	-0.1309051216
N	-2.7898565083	7.9787365877	1.9823012839
O	-2.4273972837	8.2819503909	0.8335181839
O	-3.6273922250	8.6793069712	2.5745452978
O	-2.3147796339	6.9749515929	2.5388408181

n=17 OPLS/AA

N	-7.0081441783	-1.1365940876	-4.0543812257
H	-7.5455999384	-1.3215061086	-3.1992876939
H	-7.5026265138	-1.5334848406	-4.8619700044
H	-6.0799691283	-1.5684257226	-3.9753366839
H	-6.9043816561	-0.1229598588	-4.1809296877
N	-1.0298467706	5.1562707471	-4.5085013204
H	-0.6759811600	4.7880308799	-5.3992395690
H	-1.6822468103	4.4805231429	-4.0938037811
H	-1.5170694712	6.0446362762	-4.6748030066
H	-0.2440892962	5.3118923307	-3.8661597926
N	-3.7024544435	1.5077126593	-4.4345109776
H	-2.7780052993	1.6645001322	-4.8528978390
H	-4.1997894308	0.7828911688	-4.9651015945
H	-4.2419606965	2.3809526625	-4.4594192893
H	-3.5900614470	1.2025068266	-3.4606255952
N	-3.7070788889	-2.5332249282	-1.3670388516
H	-4.7323149715	-2.5889574855	-1.3716164673
H	-3.3134177517	-3.4494748779	-1.6114803050
H	-3.3833388873	-2.2601320730	-0.4317056341
H	-3.3992449436	-1.8343353306	-2.0533530046
N	0.3671693693	1.2128629132	-0.8710473144
H	0.5911330034	2.2023321791	-0.7128481992
H	-0.0421936876	0.8139939641	-0.0180759581
H	1.2249585531	0.7018112792	-1.1103368204
H	-0.3052201736	1.1333151940	-1.6429281258
N	-3.6445397257	1.2160136273	0.7032211899
H	-4.2971781195	0.5696785978	0.2443636367
H	-4.1719710743	1.8872766762	1.2737093820
H	-3.1057133387	1.7217160992	-0.0096369016
H	-3.0032970061	0.6853825067	1.3044481956
N	-6.5584106776	2.3284894674	-1.4030297263
H	-6.9220259536	1.3734536483	-1.3033973369
H	-7.0574872767	2.8072926069	-2.1619136352
H	-5.5559977501	2.2919207257	-1.6222706305
H	-6.6981320841	2.8412899584	-0.5245372055
N	-0.2835828522	-2.3871116260	-5.2434201155
H	-1.2158195588	-1.9595409250	-5.1949008809
H	-0.0584976071	-2.6073376460	-6.2206978744
H	-0.2734372903	-3.2500056931	-4.6870511547
H	0.4134221393	-1.7315618234	-4.8710305048
N	-3.8172424649	-1.0995230649	-8.4121988850
H	-3.1119075880	-1.7080496020	-8.8439792257
H	-3.3829286441	-0.5527631539	-7.6594307998
H	-4.5743127988	-1.6746471215	-8.0244975141
H	-4.1998201417	-0.4626329749	-9.1208884211
N	-5.5556126221	5.5845314624	-3.0895061555
H	-5.4234716258	5.3156007142	-2.1074414390
H	-5.1656778945	6.5218615083	-3.2431224912
H	-6.5582216861	5.5904466314	-3.3108104699
H	-5.0750791533	4.9102167335	-3.6966492654
N	-2.3055670623	4.3731602821	-8.6840894159
H	-2.6198132361	4.6092674922	-7.7355437790
H	-2.1406543566	5.2365000843	-9.2148274595
H	-3.0273146056	3.8147027686	-9.1546648225
H	-1.4344863568	3.8321710133	-8.6313206786
N	0.2409462054	-3.5776272167	-1.2233823921
H	0.8574009734	-3.1083607522	-1.8971839963
H	-0.5847464207	-3.9433459085	-1.7119683222
H	-0.0556651546	-2.9061009261	-0.5055316652
H	0.7467960241	-4.3527008232	-0.7788462410
N	1.0998004447	1.7339851835	-4.6934416212
H	0.0785982923	1.6920156378	-4.7915273935
H	1.3431768473	2.3480623662	-3.9073679441
H	1.5116894489	2.1048718134	-5.5577483260
H	1.4657361958	0.7909908756	-4.5171229166
N	-0.3811378922	0.3321913983	-9.2766825297
H	-1.1634513821	0.9332720707	-9.5611534881
H	0.4741211590	0.6324254027	-9.7589958960
H	-0.5900987845	-0.6419278735	-9.5250019085

H	-0.2451233230	0.4049965786	-8.2615791030
N	-3.9356171619	-4.4699464602	-6.3248406253
H	-4.7306436192	-3.8293583339	-6.4335415096
H	-3.6339052620	-4.4775389263	-5.3434401110
H	-3.1593985199	-4.1560093758	-6.9191163617
H	-4.2185220209	-5.4168785810	-6.6032646247
N	-6.3216911137	1.8091216369	-7.7541693084
H	-6.3350516315	0.7827686345	-7.7285351795
H	-5.3821688488	2.1453211567	-7.5122964449
H	-7.0014261810	2.1772371753	-7.0783833120
H	-6.5681178066	2.1311585817	-8.6974622720
N	-1.6312001647	5.5369735992	-0.4313487708
H	-1.9747704871	5.9446338909	-1.3088501200
H	-1.2045432531	6.2727167192	0.1438734702
H	-0.9304096766	4.8161806767	-0.6401208448
H	-2.4150775765	5.1143635069	0.0797015567
N	-4.0036693275	4.0509182378	-5.9959078279
O	-3.2228902980	4.9572887192	-6.3304142830
O	-4.4760816191	4.0305905938	-4.8472428095
O	-4.3120363136	3.1648746870	-6.8100670467
N	1.8056473198	-1.1410330183	-3.0942512598
O	1.5878779909	-0.7725105229	-4.2603497122
O	2.0310134965	-0.2995103153	-2.2087753777
O	1.7980504660	-2.3510791908	-2.8136284635
N	-1.7931345497	-0.3857232803	-2.6768789546
O	-3.0055145752	-0.2028348818	-2.4776192789
O	-1.0597861871	0.5738420017	-2.9674861804
O	-1.3141025010	-1.5281778805	-2.5855313310
N	-2.8436614288	2.9245953392	-1.8837457792
O	-2.1462618412	2.6658225938	-0.8889149431
O	-2.3665095719	3.5837168076	-2.8223100584
O	-4.0182138188	2.5242462938	-1.9400123814
N	-1.4377697820	-1.0828040887	0.7556555110
O	-1.1535944339	0.1052699699	0.9809160574
O	-2.6251520949	-1.4476799548	0.7530562227
O	-0.5345620901	-1.9060029439	0.5329940737
N	-1.9657758672	-4.6237458139	-3.2461335111
O	-2.1341496485	-4.5319611629	-2.0188422830
O	-2.9305326746	-4.8705596500	-3.9886597017
O	-0.8326443662	-4.4687165039	-3.7308989389
N	-3.7819921862	1.8173251389	-9.8881508933
O	-4.1860773159	2.9896921335	-9.8154271087
O	-2.5651551467	1.5841998630	-9.9774713050
O	-4.5947447503	0.8780826641	-9.8715542529
N	-4.8470097235	4.2420622879	0.3446483563
O	-5.4017095742	4.8251761223	-0.6015568237
O	-3.7429001891	4.6359658387	0.7555011762
O	-5.3964198495	3.2650441161	0.8800011474
N	-7.1810467371	2.5412511396	-4.5181635476
O	-7.5741132176	2.7398136024	-5.6796667122
O	-7.3239280003	3.4228994201	-3.6548564383
O	-6.6450985620	1.4610395267	-4.2199672524
N	-1.2570024595	-2.7973088005	-8.3606297875
O	-0.1390562039	-2.8076061366	-7.8192352499
O	-1.4700557589	-2.0523802780	-9.3315611439
O	-2.1618961442	-3.5319405784	-7.9310926229
N	-6.3685597817	-0.7990965865	-1.0940255864
O	-7.3555653717	-0.1454568679	-1.4703140321
O	-6.2590097220	-1.9955684310	-1.4094171219
O	-5.4911035449	-0.2562640237	-0.4023450483
N	0.4387495732	3.3099502994	-7.4653036439
O	-0.0712038155	3.0432511480	-8.5661383340
O	1.4561841740	2.7046830393	-7.0891437208
O	-0.0687320475	4.1819174127	-6.7406282937
N	-6.5839914971	-1.9064486982	-7.1437120724
O	-7.5200010060	-2.1820957018	-6.3749970150
O	-6.5589197594	-0.8078321318	-7.7228760898
O	-5.6730529925	-2.7294189235	-7.3332632652
N	-3.0569965910	7.2788608561	-3.4939954529
O	-4.2125975174	7.7306424023	-3.5531380294
O	-2.2734021928	7.4513525109	-4.4422804096
O	-2.6849897634	6.6545871525	-2.4865671087
N	-4.0283529220	-1.5920892660	-5.0219590014
O	-4.6024271205	-2.2645200082	-4.1494375848
O	-4.6029467026	-0.6160227737	-5.5320120386
O	-2.8796840181	-1.8957252605	-5.3844276725
N	-1.4401746380	0.8806096935	-6.4139208478
O	-2.4974414134	0.6520011387	-7.0246114448
O	-1.4027519031	1.7711627144	-5.5487429662
O	-0.4203297764	0.2186646947	-6.6684083374
N	0.7824433543	4.1503397500	-1.9371683135
O	0.5034124211	3.8934057991	-0.7543138871
O	1.3270456991	3.2894022017	-2.6479514977
O	0.5168717290	5.2682121491	-2.4092399358

n=18 OPLS/AA

N	2.6148761467	-7.5328212979	2.9818980905
H	3.5657244152	-7.2899013920	2.6800478176
H	2.3998816269	-8.4948461932	2.6946348961
H	1.9431630100	-6.8881472490	2.5489635035
H	2.5507364608	-7.4583901211	4.0039458510
N	3.1726248534	-2.0628699395	2.6720325847
H	3.0281749922	-1.2998496421	2.0003404224
H	2.5896744517	-2.8659094952	2.4083452951
H	2.9116670460	-1.7447208215	3.6127332202
H	4.1609827828	-2.3409990561	2.6667107471
N	-1.8091192179	-10.3470644233	-0.6174813146
H	-1.5161519657	-10.1296295620	0.3422730122
H	-1.6513495126	-9.5287150260	-1.2171876341
H	-2.8069333457	-10.5890500909	-0.6242212759
H	-1.2620417622	-11.1408628023	-0.9707884260
N	-1.3376842273	-3.8332159352	5.0692225901
H	-1.1755141216	-3.5626269188	4.0921225945
H	-1.2352678002	-3.0097506859	5.6739130431
H	-0.6533205925	-4.5473596382	5.3446871267
H	-2.2866342369	-4.2131262344	5.1661636443
N	2.6924067052	-4.0484634964	6.0175703365
H	3.3078308346	-4.3946205360	5.2721412368
H	2.4344356096	-3.0737922778	5.8234012997
H	3.1831824483	-4.0997352851	6.9179846288
H	1.8441785279	-4.6257062239	6.0567534546
N	-3.9937627508	-5.6328043718	-3.6938978769
H	-4.3404350536	-6.5797526111	-3.5006533578
H	-4.7880874479	-4.9869976990	-3.7727439449
H	-3.4655514227	-5.6347582572	-4.5743662178
H	-3.3809774166	-5.3297098422	-2.9278277989
N	0.3387715509	-5.2114216418	-0.4997466284
H	-0.4480408513	-5.8235985284	-0.7455144064
H	1.0111259504	-5.7324222839	0.0753469100
H	-0.0089461002	-4.4025526641	0.0285136636
H	0.8009464385	-4.8871136869	-1.3573329201
N	-0.4716702446	-1.0410519657	-2.3283588698
H	-1.0411189632	-0.4302559508	-1.7309540802
H	-0.6051471443	-0.7787251290	-3.3120271236
H	-0.7591552520	-2.0173301759	-2.1924590875
H	0.5187398264	-0.9378960122	-2.0779946063
N	-4.3844708306	-2.3943312342	0.9846112256
H	-3.6600943615	-2.6426210300	0.3006031803
H	-5.2975538869	-2.3540068079	0.5167565918
H	-4.1688740922	-1.4777142616	1.3939605824
H	-4.4113602761	-3.1029830792	1.7271238816
N	2.4592023823	-11.0193795607	-0.6153685464
H	1.6507625533	-11.2236923718	-0.0162713791
H	2.1613381762	-11.0015731733	-1.5978116912
H	3.1726593584	-11.7465516204	-0.4871676768
H	2.8520486538	-10.1057012764	-0.3602228550
N	-4.1568484068	-6.3133434541	0.1595544150
H	-4.9642410560	-5.7935938360	-0.2040573864
H	-4.1508156577	-6.2626662840	1.1850445982
H	-3.2910666855	-5.9020485624	-0.2085407456
H	-4.2212710143	-7.2950646276	-0.1342291603
N	-4.7456106551	-1.1054198509	-2.9192720386
H	-4.4929447682	-0.7147928808	-2.0039542700
H	-5.4921007148	-0.5389327727	-3.3388905158
H	-5.0742974696	-2.0708630945	-2.8004450826
H	-3.9230994216	-1.0970902750	-3.5337973945
N	-2.6908903125	-10.3132587187	-4.6091115792
H	-1.7103948396	-10.5413728695	-4.4070505503
H	-3.1074245004	-11.0630567915	-5.1735022291
H	-2.7364918049	-9.4285391366	-5.1281754375
H	-3.2092491502	-10.2200662994	-3.7277179030
N	-0.3849050001	-0.6302333787	1.7524519180
H	-0.5218732266	-1.6050064673	1.4604018738
H	-0.1875354186	-0.5992056000	2.7595850823
H	-1.2355046604	-0.0906288484	1.5535984726
H	0.4052931718	-0.2260935485	1.2362219587
N	4.0372938605	-4.6254278212	-0.2424976950
H	4.9407159810	-4.6627556020	0.2439909550
H	3.6926028233	-3.6582886326	-0.2505328732
H	3.3613342458	-5.2256482966	0.2443796040
H	4.1545232718	-4.9550187902	-1.2078279921
N	1.2379732634	-8.2538314413	-3.8650666035
H	0.3310313492	-8.1151137313	-4.3259894373
H	1.1239802133	-8.1571113829	-2.8492489524
H	1.9048947924	-7.5501687473	-4.2031642393
H	1.5919858155	-9.1929317684	-4.0818642339
N	-0.2146832363	-4.7173185892	-4.5563530697
H	-0.8152459228	-5.2746017399	-5.1752198140
H	-0.4284752774	-4.9430394286	-3.5777940519
H	-0.3852944531	-3.7185335918	-4.7223526651
H	0.7702821233	-4.9331001394	-4.7500463506
N	-1.4123888398	-7.2542638118	2.8220310271
H	-1.0197649981	-7.3243432623	3.7681660529
H	-1.2923685323	-8.1498721876	2.3344703145
H	-0.9251760361	-6.5141801595	2.3032484179

H	-2.4122454104	-7.0286597060	2.8822402444
N	-1.1895553586	-7.4682310793	-1.9589115227
O	-1.4772199037	-7.2901106187	-0.7636960100
O	-0.7837425798	-6.5179670203	-2.6483548617
O	-1.3077036874	-8.5966165073	-2.4646841037
N	-2.1427498489	-2.1005104916	-4.6026355312
O	-2.5688710539	-0.9781888130	-4.2835232011
O	-0.9401638078	-2.2544606450	-4.8730131838
O	-2.9192153099	-3.0688827964	-4.6513702481
N	0.4279357457	-11.1832903967	-3.0863337037
O	1.6260446009	-10.8553570095	-3.0827594262
O	-0.0755918544	-11.7072682302	-2.0788987942
O	-0.2666460687	-10.9872457927	-4.0973437046
N	-5.8670027637	-3.7034209521	-1.6102979708
O	-5.6136200524	-3.6327747385	-2.8243095955
O	-6.1206943276	-2.6710996824	-0.9676065420
O	-5.8666939110	-4.8063893232	-1.0389143148
N	2.6185858130	-5.3693262843	-3.1414477084
O	1.8229743812	-4.6365454326	-2.5306674010
O	2.2947528014	-5.8627308214	-4.2344719782
O	3.7380311576	-5.6087027916	-2.6592033578
N	2.4201255637	-7.8280188180	-0.2956022965
O	3.2250820533	-8.6773398133	0.1211911866
O	2.3757542148	-6.7035680154	0.2303820064
O	1.6595398106	-8.1031488467	-1.2383808416
N	-2.2749103956	-3.6687938099	-1.6046772855
O	-1.1699225675	-3.5332702276	-2.1557246239
O	-2.8558236506	-2.6804553786	-1.1264045115
O	-2.7989853907	-4.7926567283	-1.5319026625
N	1.0405650508	-1.4632683248	5.0833979055
O	2.2739904838	-1.6102447958	5.0922029626
O	0.3495489036	-1.9588169188	5.9889067051
O	0.4981553283	-0.8207427424	4.1690833128
N	-1.9384396593	-7.2754855148	-5.6349347248
O	-0.7759692662	-7.6682212724	-5.4414428107
O	-2.1948408082	-6.0602140764	-5.6151434946
O	-2.8445096329	-8.0980218578	-5.8482180408
N	1.2786008208	-4.6502132662	2.5374516726
O	0.7480467991	-5.7616070718	2.3751736713
O	1.0875499433	-4.0184842043	3.5897994599
O	2.0002063009	-4.1705481362	1.6473811699
N	-4.2294994170	-8.9611456698	-1.9424609414
O	-3.9763084946	-10.0931214689	-2.3869191036
O	-4.2209899198	-8.7588916006	-0.7168840198
O	-4.4912000473	-8.0314231914	-2.7235803297
N	-1.3863906087	-3.6414399642	1.5754640180
O	-1.7681756807	-3.0445524956	2.5957499678
O	-0.6075646690	-3.0838712612	0.7845372148
O	-1.7834317961	-4.7958970653	1.3461046866
N	-2.9092938576	0.1087873974	-0.0646087231
O	-3.9508489069	0.0539254632	-0.7392724603
O	-2.9531292766	-0.0816849943	1.1621009553
O	-1.8239025156	0.3541219208	-0.6166551086
N	-4.0019970411	-5.2402579601	3.2897924249
O	-4.5813092055	-4.3392592301	2.6608118086
O	-3.5634979023	-5.0182139298	4.4305960298
O	-3.8611839022	-6.3633016244	2.7779690243
N	4.6671794065	-4.8331904247	2.8832737495
O	5.1207370008	-3.8575070017	2.2625118967
O	4.2095186917	-4.6827210616	4.0282294481
O	4.6712825304	-5.9593441173	2.3590794816
N	0.7912446830	-6.5038949972	5.5045994118
O	-0.0883033112	-7.2306251495	5.0134003456
O	0.4757199935	-5.4487205856	6.0791157670
O	1.9863183286	-6.8323395209	5.4212820558
N	0.3026943667	-10.0995297160	1.6238624835
O	0.4144278036	-11.1346739744	0.9463597792
O	1.2865243125	-9.6456424459	2.2313844127
O	-0.7928698981	-9.5182722597	1.6938433148
N	2.4047873529	-1.2147571572	-0.2669993239
O	1.8848713744	-0.9427366486	-1.3618555379
O	2.0880715429	-0.5720949196	0.7477391917
O	3.2414198148	-2.1294406397	-0.1868815611

n=19 OPLS/AA

N	-5.7284469142	5.5760375350	-1.8044040101
H	-6.4225392757	4.9816764145	-1.3362088054
H	-6.1969542141	6.1626252480	-2.5048684634
H	-5.0276371915	4.9848327503	-2.2665491814
H	-5.2666576514	6.1750151481	-1.1099891343
N	-1.8492410361	0.6571078735	-2.2865250241
H	-2.0892145460	0.3032014637	-1.3530374423
H	-2.1797919183	1.6245868210	-2.3811573968
H	-2.2974800200	0.0709063572	-3.0004473725
H	-0.8304778939	0.6297365076	-2.4114569754
N	-9.4452898886	-1.7301016975	-4.5859450468
H	-8.8140223435	-1.8573235445	-3.7862251047
H	-10.2238911996	-2.3958466379	-4.5166748635
H	-8.9299923057	-1.8956892403	-5.4584613450
H	-9.8132530909	-0.7715474911	-4.5824180950
N	0.5917654677	4.9673788909	-5.3307319966
H	0.7411147634	4.6172234660	-6.2843157968
H	-0.3785831059	4.7867204073	-5.0478524403
H	0.7734148908	5.9776077924	-5.3047208863
H	1.2311154680	4.4879635570	-4.6860397919
N	-5.3106938487	-1.6022297826	-4.1387689012
H	-5.5403249328	-2.2345325358	-3.3630763776
H	-5.8896347390	-0.7567330781	-4.0739811432
H	-5.4941366973	-2.0750576476	-5.0315275783
H	-4.3186792495	-1.3425964849	-4.0864897503
N	-0.6945040396	1.8039320959	-6.8786700018
H	-1.3094444811	2.6124447090	-6.7290314189
H	-1.2520427695	0.9419205462	-6.8607440933
H	0.0127571396	1.7696108018	-6.1351384307
H	-0.2292866462	1.8917531141	-7.7897659187
N	-0.9059465240	7.2568793491	-9.1621487366
H	-1.3279434110	7.7731524324	-9.9429283652
H	-0.4023812377	7.9110730313	-8.5516675215
H	-0.2500740584	6.5573139062	-9.5291296953
H	-1.6433878000	6.7859785294	-8.6248701249
N	-2.6872141104	7.3039513490	-4.0060473475
H	-2.4530914468	6.3163293635	-4.1610489272
H	-2.2016903658	7.8838493912	-4.7004677098
H	-2.3927607330	7.5814655267	-3.0623744095
H	-3.7013136681	7.4341601528	-4.1002984944
N	-4.3318945876	-0.2614649574	-8.1341961667
H	-4.3441285406	0.7059853953	-7.7905011812
H	-5.2512485007	-0.6914840857	-7.9789331089
H	-3.6147470721	-0.7950906503	-7.6290441253
H	-4.1174542488	-0.2652695466	-9.1383059169
N	-5.3096808961	0.0223576264	0.4358184248
H	-4.3124417620	0.2167880596	0.2876731800
H	-5.4419995711	-0.4006900847	1.3619701877
H	-5.6431193684	-0.6258106064	-0.2873221226
H	-5.8411619117	0.8991433263	0.3809523099
N	-3.6010679378	3.8464285814	0.7589253034
H	-4.5184057219	4.1806377200	1.0767802063
H	-3.7173771264	3.2871231620	-0.0942360564
H	-3.1747951580	3.2676755506	1.4921183003
H	-2.9936946385	4.6502782186	0.5610390731
N	-8.2482266939	1.9236031076	-2.3744907465
H	-8.2939436010	1.0088541704	-1.9103997599
H	-9.1413271790	2.1143063165	-2.8437864902
H	-7.4913819096	1.9179342512	-3.0683130298
H	-8.0662541302	2.6533168016	-1.6754632542
N	-6.0841945328	3.9914126632	-11.6637051984
H	-5.5659774880	4.8518457494	-11.4507683624
H	-6.5092843758	4.0695108378	-12.5950665601
H	-6.8207075888	3.8528915248	-10.9618534072
H	-5.4408081741	3.1914033788	-11.6471322564
N	-8.4063414567	4.4240473513	-5.5970007603
H	-9.3322960791	4.2093679191	-5.2087265322
H	-7.7837014204	3.6187873538	-5.4624258476
H	-8.0153363270	5.2430296642	-5.1167732269
H	-8.4940329021	4.6250042590	-6.6000770564
N	0.1442376146	3.7469412602	-0.7657825155
H	-0.5198521085	4.5285869636	-0.7183385319
H	0.9627346266	3.9573673714	-0.1826717382
H	0.4430480907	3.6102771666	-1.7385470034
H	-0.3089807969	2.8915343006	-0.4235727422
N	-7.7969942961	1.5987408803	-8.0666832386
H	-7.5389021862	0.6160137798	-8.2145751124
H	-8.5898520289	1.6502295445	-7.4163293207
H	-8.0622580093	2.0216858553	-8.9638946042
H	-6.9969647085	2.1070333846	-7.6719340609
N	-2.3401820058	3.7085209088	-10.4943571255
H	-2.6932172041	3.0293825749	-11.1787352153
H	-2.1840871170	4.6115095564	-10.9574775996
H	-1.4551829965	3.3685877986	-10.1000758070
H	-3.0282410494	3.8246030439	-9.7411405467
N	-5.2882705646	6.4896892439	-8.3162234466
H	-5.5726738339	6.7954550647	-7.3782159089
H	-6.1217252292	6.2459544090	-8.8641115170
H	-4.6778069128	5.6678437558	-8.2378675428
H	-4.7808765594	7.2495040439	-8.7846979040
N	-4.3931779032	3.1196816823	-5.0815711429
H	-5.0768702816	2.8668617343	-5.8046775688
H	-3.9992795577	4.0443871604	-5.2913294744
H	-3.6395668164	2.4225370942	-5.0643875140
H	-4.8569956231	3.1449404942	-4.1658907187
N	-10.0066922891	1.7170924225	-5.0194882832
O	-10.3832783072	0.6240515742	-4.5651067411
O	-9.9809335109	2.7190766708	-4.2857431215
O	-9.6558647667	1.8081490957	-6.2076159436
N	-6.8152227346	3.3987428720	0.1671208758

O	-6.7870729301	2.1775073693	0.3925301620
O	-6.1558564955	4.1766140227	0.8764660291
O	-7.5027393316	3.8421075810	-0.7676343163
N	-4.8743715326	1.9204242305	-2.1715018660
O	-4.7743666191	0.7891865133	-2.6748090837
O	-5.5678876407	2.7883459417	-2.7271683326
O	-4.2808598601	2.1837404486	-1.1125273293
N	-4.5379560157	1.3196470084	-10.8739313825
O	-4.1827936754	2.1908764672	-11.6850028054
O	-3.7602493995	0.3999374870	-10.5700815407
O	-5.6708258841	1.3681271099	-10.3667093932
N	-2.2209906375	1.1685626659	0.7731705763
O	-2.6242383058	0.1508624664	0.1860628070
O	-2.7958025681	1.5608137279	1.8021255120
O	-1.2429302512	1.7940123070	0.3313230543
N	-0.1926846624	8.1472146153	-6.1992027496
O	-1.3809242922	8.3957093656	-5.9358496554
O	0.5085505891	7.5288439371	-5.3813367843
O	0.2943201078	8.5170908408	-7.2804226796
N	-6.6834529300	1.0930058158	-5.0968036875
O	-7.0617521550	2.2742025976	-5.0283802278
O	-5.7756800171	0.7761356697	-5.8832892627
O	-7.2129270441	0.2286784842	-4.3787409939
N	-7.9534502559	4.5620568393	-9.2289986016
O	-8.0084676512	3.5361229318	-9.9271752373
O	-8.5309984910	4.5970017279	-8.1298012572
O	-7.3208841165	5.5530466561	-9.6300196332
N	-4.7226982715	3.1630397702	-8.1272486070
O	-4.5389978629	3.9715378815	-9.0522366474
O	-5.7693540757	3.2100718349	-7.4599274525
O	-3.8597421811	2.3075089054	-7.8695815138
N	-2.4814844371	6.3901490493	-0.6724697379
O	-3.6769462784	6.4106539359	-0.3356189600
O	-2.0464669644	7.2368430703	-1.4705217062
O	-1.7210394563	5.5229494435	-0.2112681761
N	0.8537822649	2.2963928879	-3.5411649044
O	0.7645198443	2.0402822819	-4.7533767211
O	0.6006085269	1.4177089534	-2.7004298019
O	1.1962186993	3.4311883419	-3.1696878910
N	-6.2170114428	6.7554126703	-4.7066142661
O	-7.0861383718	6.1106935236	-4.0967179519
O	-6.2727599149	6.8572826551	-5.9433569738
O	-5.2921352971	7.2982622691	-4.0797673680
N	-2.5884486753	0.0186366738	-5.2238911213
O	-2.6410539838	1.2308799836	-4.9579649092
O	-2.8209026289	-0.8219246567	-4.3393348935
O	-2.3033891838	-0.3530456047	-6.3743744873
N	-2.6313336133	5.0493237756	-6.8441413323
O	-1.8585006933	4.1223045327	-6.5502323126
O	-3.5282657911	5.3846830035	-6.0528987855
O	-2.5072342558	5.6409842669	-7.9292937722
N	-2.3799351198	4.2740551523	-3.0701317986
O	-1.5645852327	4.8612607851	-3.8004824112
O	-3.5402093528	4.7045219963	-2.9629874494
O	-2.0350104963	3.2563818562	-2.4469250335
N	-7.2839766836	-1.4199887723	-1.7939342809
O	-7.9240606111	-0.4556831899	-1.3429074297
O	-6.1398712098	-1.6576993615	-1.3725861453
O	-7.7879986358	-2.1465843504	-2.6663099701
N	0.3237543261	4.0010515138	-8.8721251316
O	0.8712854371	4.0980285768	-7.7613492574
O	0.2753627491	4.9850232748	-9.6287463973
O	-0.1753856097	2.9201018196	-9.2262800251
N	-3.5120233226	6.5953759144	-10.8889009641
O	-4.5561924161	6.0366883650	-11.2638636786
O	-3.5688864207	7.5527507651	-10.0994594563
O	-2.4109902447	6.1966882922	-11.3033800910
N	-6.9625478488	-1.7500596741	-7.0915803306
O	-8.0534932178	-2.1589765724	-6.6607171468
O	-5.9026417326	-2.1946482871	-6.6204562484
O	-6.9315085710	-0.8965534758	-7.9935683229

n=20 OPLS/AA

N	-0.3517348589	5.2403733911	4.3012991896
H	-0.5563215311	5.8758731934	5.0813763603
H	0.1697639279	5.7460364160	3.5756418283
H	-1.2318606916	4.8876984582	3.9073011764
H	0.2114786600	4.4518861155	4.6408781531
N	-4.1636394095	0.4634639327	4.2457542697
H	-3.8160168655	0.6703109833	3.3020334624
H	-3.4121149300	0.0390620412	4.8019275131
H	-4.9539973276	-0.1890337971	4.1839756479
H	-4.4724281765	1.3335167049	4.6950795363
N	2.0853871724	-4.6802029814	-0.6501287265
H	1.1435239936	-4.6652929849	-0.2415855316
H	2.4323642944	-5.6462349861	-0.6751032738
H	2.7153985329	-4.1049181393	-0.0788419682
H	2.0502609512	-4.3043658006	-1.6049837345
N	-3.1891479331	-3.8519227229	3.7378453097
H	-4.1919015813	-4.0355414821	3.8603417168
H	-2.7608864692	-3.6667117742	4.6524620100
H	-3.0626888685	-3.0349084850	3.1289681759
H	-2.7411157899	-4.6705293292	3.3096094554
N	2.2002462057	-1.5089260279	-4.0047984793
H	1.9763746809	-2.4396199336	-4.3761830399
H	3.0354643758	-1.1437038632	-4.4773003991
H	2.3798905170	-1.5770399633	-2.9961737040
H	1.4092550311	-0.8753412578	-4.1695371358
N	-4.8502188516	-1.4549633740	0.3459515276
H	-5.6032815659	-1.8549839021	0.9178922789
H	-4.9870385497	-0.4409468257	0.2605964129
H	-4.8656472467	-1.8821796635	-0.5875814020
H	-3.9449087775	-1.6417434941	0.7928993775
N	3.2819154861	1.6590073374	-6.3692299716
H	2.2726074186	1.5407956469	-6.5160608907
H	3.6496834954	0.8509800226	-5.8534070995
H	3.4511838892	2.5157035554	-5.8291787500
H	3.7541861581	1.7285500096	-7.2782732891
N	4.8998849628	-0.1653341291	-0.7984303890
H	5.0634595714	-0.8980988885	-0.0980481702
H	5.1456160003	-0.5210519061	-1.7297283036
H	5.4809618070	0.6525593175	-0.5801747238
H	3.9095026321	0.1052542471	-0.7857696762
N	5.6499709084	3.2499227680	-2.8296521055
H	5.5873449117	2.4518571218	-3.4726181284
H	6.4470411853	3.8407007445	-3.0940288095
H	5.7810958378	2.9073397203	-1.8706533400
H	4.7844016379	3.7997927080	-2.8813087705
N	0.8860398355	-2.7556058784	2.8494278713
H	1.9037639341	-2.8083435158	2.9746975740
H	0.4952637378	-3.7046204529	2.8194268020
H	0.6741731104	-2.2702658981	1.9697727580
H	0.4709595511	-2.2391936979	3.6338144733
N	1.4681983104	4.7350424849	-1.3924719168
H	1.5878587070	5.5039523981	-0.7226248951
H	0.5166690398	4.7613905791	-1.7773517818
H	1.6196361480	3.8386584899	-0.9151901980
H	2.1486294633	4.8361692211	-2.1547201397
N	-0.2049605774	2.6255579385	-4.4950374233
H	-1.1109671518	3.0465746359	-4.2581131205
H	-0.2229800844	2.3000011567	-5.4686506754
H	-0.0236288398	1.8302155402	-3.8715113256
H	0.5377328839	3.3254408311	-4.3818743410
N	-1.9157933231	-3.3633562920	-1.4884647499
H	-2.8574073716	-3.2344060584	-1.8770145131
H	-1.3926102603	-4.0266321835	-2.0720564394
H	-1.9874235001	-3.7311470367	-0.5325177797
H	-1.4257330773	-2.4612397637	-1.4722706460
N	3.5753229633	4.7025636570	2.0544629111
H	3.3691921294	5.7047243474	2.1406030730
H	4.4473082708	4.5765857118	1.5272002950
H	2.8058653683	4.2361561917	1.5598574018
H	3.6789258842	4.2927893532	2.9901909586
N	-2.2364833002	4.7033877276	0.8877965555
H	-1.5578256590	5.4738649176	0.8828162745
H	-2.9475432781	4.8782652936	1.6075508401
H	-1.7518056060	3.8216978848	1.0926039720
H	-2.6887579970	4.6397235647	-0.0317848695
N	-3.9188291400	1.6480283256	-2.4581829334
H	-4.1653129787	1.6801539317	-1.4619652061
H	-4.5072573828	0.9541624632	-2.9341420215
H	-2.9312389274	1.3855119166	-2.5581140650
H	-4.0715075110	2.5722850221	-2.8785094708
N	3.4756227647	0.4958741888	2.5847212824
H	3.0899854933	0.8678399317	3.4605988301
H	3.8142715807	-0.4610728180	2.7390070267
H	4.2544792357	1.0887305071	2.2746575668
H	2.7437543737	0.4879994968	1.8646225589
N	-0.4023969190	1.0468267035	4.2634496808
H	0.5471202347	1.2726108492	4.5823145299
H	-1.0204269105	1.8488473850	4.4338505071
H	-0.7513911079	0.2302365935	4.7788322305
H	-0.3848889677	0.8356122063	3.2588017665
N	-0.3001473719	0.7254623165	-0.1948130712
H	-0.5452837238	-0.2231737894	-0.5017883231
H	0.2766846277	1.1792055596	-0.9128775523
H	0.2260341249	0.6750869553	0.6854317126
H	-1.1580247552	1.2707296166	-0.0500184211
N	-1.5226276124	-2.1697422925	-5.7687080507
H	-1.9208678662	-2.7322659710	-6.5297651504
H	-1.2072363948	-1.2659307552	-6.1400404763
H	-0.7248472165	-2.6675713558	-5.3564603245
H	-2.2375593599	-2.0132016360	-5.0485669927
N	5.5585808571	2.7588522700	0.4353815997
O	6.1059475661	2.1541343377	-0.5014875582

O	5.4590225703	3.9965833459	0.4017982353
O	5.1107720745	2.1258386169	1.4058349034
N	-0.8004549785	-0.2146438348	-2.9900886760
O	-0.7374336860	-1.1295956392	-2.1522883279
O	-1.5242252461	0.7724754342	-2.7784993109
O	-0.1397054713	-0.2868113574	-4.0394792339
N	-3.0316274648	3.5711302901	4.1862496196
O	-2.8541345701	4.6025875192	3.5172128734
O	-4.1824313185	3.1426407867	4.3735065199
O	-2.0583157223	2.9681620789	4.6680298534
N	3.9839028101	-2.5123791492	1.6989046581
O	3.4611368568	-2.1967110559	2.7806104055
O	3.6116095551	-3.5379142377	1.1050246826
O	4.8789627389	-1.8025115823	1.2110784935
N	-3.4377322941	1.4961419593	0.7165691590
O	-4.4415228792	1.1515807902	0.0710461362
O	-3.3829337188	1.2846887770	1.9393948124
O	-2.4887395203	2.0521567583	0.1392660635
N	0.7940765630	2.7579689944	1.6704243288
O	-0.2423930017	3.3202092674	2.0611091478
O	1.0056209038	1.5702063505	1.9662060127
O	1.6190024510	3.3834918688	0.9839572733
N	1.9354673961	-1.3322515273	-0.3058674390
O	2.3512851144	-0.2863996719	0.2197820950
O	2.3982769277	-1.7058390482	-1.3963983247
O	1.0568394389	-2.0045164030	0.2590143673
N	-1.1180731456	-4.6113552917	1.3238438338
O	-0.8635113667	-4.7371442135	2.5331384045
O	-0.2183112184	-4.7620155456	0.4807851923
O	-2.2723977810	-4.3349058934	0.9576076098
N	-1.4033596806	-1.7186338433	5.3329538869
O	-2.1111085587	-0.7004145693	5.4060219828
O	-0.2382770485	-1.6361637344	4.9101075171
O	-1.8606938028	-2.8193241123	5.6827324425
N	-2.1615617787	4.2012174237	-2.3772785959
O	-2.3813752131	3.6765370076	-3.4815477778
O	-1.0095624648	4.5550029623	-2.0760290576
O	-3.0937484087	4.3721124389	-1.5742583059
N	-1.9685737018	-1.2306656803	1.6566683694
O	-0.9509921265	-0.7332027378	2.1666451035
O	-2.0799848919	-1.2866602954	0.4207597722
O	-2.8747448166	-1.6721343630	2.3826008169
N	0.9547911912	6.3675149592	1.4704956269
O	1.8011901576	6.3908591678	0.5616052328
O	-0.2556359068	6.3533688013	1.1917770634
O	1.3188196159	6.3583169012	2.6581055407
N	2.9739587328	4.2878561591	-4.1584825659
O	3.6979230806	3.8421904725	-5.0641732581
O	1.7404787458	4.3101084932	-4.3035682855
O	3.4834747823	4.7112698526	-3.1077053084
N	-4.1497095210	-1.6741913896	-3.0654179322
O	-3.3054380032	-2.0052350603	-3.9143193418
O	-4.8273657283	-0.6464405310	-3.2313340203
O	-4.3163249656	-2.3708991383	-2.0505996174
N	0.4094820674	0.3668831665	-6.7762247272
O	1.3133945648	-0.4735011824	-6.6358172703
O	-0.7663012440	-0.0047616457	-6.9259967182
O	0.6813531001	1.5789133032	-6.7668601856
N	0.6260825769	-4.1234130720	-3.5972007926
O	-0.2805613845	-4.6997213534	-2.9735956266
O	0.3673976490	-3.5168162506	-4.6498832306
O	1.7914124042	-4.1537016363	-3.1681231751
N	2.2611531584	1.7221610765	-2.4516050277
O	2.3102410238	1.3044939086	-3.6204345402
O	1.1638530118	1.9809796369	-1.9301040672
O	3.3093662837	1.8810098120	-1.8042759546
N	2.2516195826	2.7226500497	4.6207045926
O	3.2675820877	3.3057518414	4.2073882493
O	2.2168827640	1.4813152599	4.6506913213
O	1.2703931062	3.3808835778	5.0040345158
N	5.0614087220	0.0365993732	-4.2441819570
O	4.2960444919	-0.3935941439	-5.1229134541
O	5.3405356306	-0.6716314174	-3.2625938422
O	5.5476464351	1.1750245974	-4.3470386575
N	-6.0195552392	-2.4907472918	3.2189672863
O	-5.8526333602	-1.4359396638	3.8534244284
O	-6.4532672927	-2.4537496003	2.0555486443
O	-5.7527648501	-3.5825534902	3.7479292120

n=21 OPLS/AA

N	-1.4855431086	3.2367222415	-4.1842779019
H	-2.2563894847	2.8733593648	-3.6115655989
H	-0.8310249311	2.4755575838	-4.3998446080
H	-0.9936264593	3.9749995512	-3.6673638393
H	-1.8611323102	3.6229721121	-5.0583370035
N	0.5448337386	-0.9024750893	-8.8269946127
H	0.9217858954	-0.4058292257	-9.6427678994
H	0.9279436481	-1.8546682891	-8.7988949146
H	-0.4785999648	-0.9472409702	-8.8963847755
H	0.8082057429	-0.4021613884	-7.9699316557
N	3.4796319388	-4.7944281569	0.5847964016
H	3.4509448238	-5.8009785745	0.3841240223
H	2.8841745164	-4.5900396055	1.3959008731
H	3.1376871071	-4.2729359805	-0.2308966572
H	4.4457212801	-4.5137594473	0.7900571728
N	3.6293005255	1.7550257073	-9.2539640696
H	3.8183036755	2.7550663151	-9.3897311113
H	4.5060737007	1.2269020734	-9.3351361827
H	2.9674674127	1.4316055375	-9.9692345420
H	3.2253574971	1.6065298772	-8.3217545748
N	-0.3317187616	-3.3581509203	-5.8152972402
H	-0.1040257828	-2.5418283631	-5.2356339866
H	0.4951967621	-3.6340394950	-6.3578199554
H	-1.0976794072	-3.1198812198	-6.4562085938
H	-0.6203663966	-4.1368538084	-5.2115258605
N	2.1021643931	-0.3177740108	-3.9746020234
H	1.1659208644	0.0590778803	-3.7857454515
H	2.3825332735	-0.9386890290	-3.2064272278
H	2.7731783003	0.4555300338	-4.0519112401
H	2.0870242223	-0.8470145614	-4.8543239902
N	0.1746511456	-5.9697853661	-1.9043671049
H	-0.1770265144	-6.9100690417	-1.6889012597
H	0.1851357598	-5.4036998163	-1.0478206038
H	-0.4369464073	-5.5267088230	-2.5999726932
H	1.1274414016	-6.0386646991	-2.2807736531
N	5.0214824586	3.1513352033	-5.9745205129
H	5.6613609377	3.3839108962	-5.2059502971
H	4.6259883782	4.0151737361	-6.3638816653
H	4.2629235407	2.5568890283	-5.6203205167
H	5.5356576008	2.6493673791	-6.7079288238
N	-3.8342447842	-1.2623396057	-4.6615866316
H	-3.5022126840	-0.8426830067	-5.5378737540
H	-3.4104471832	-2.1901759975	-4.5443266256
H	-4.8564259989	-1.3543211974	-4.6919259649
H	-3.5678929471	-0.6621778124	-3.8722210351
N	-1.8796962857	1.6971836622	-7.8357824073
H	-2.2562699974	2.6044477971	-7.5369511391
H	-2.6299077284	0.9962055014	-7.8289198494
H	-1.1343703434	1.4071294694	-7.1918931862
H	-1.4982374404	1.7809527647	-8.7853651635
N	0.3939146807	5.1992024841	-9.9934601414
H	0.7064705536	4.3386266351	-10.4581687943
H	1.1310318507	5.5343511951	-9.3621374785
H	-0.4578065195	5.0065609553	-9.4533714797
H	0.1959631426	5.9172703129	-10.7001632657
N	6.5065119479	-0.6080459187	-4.9934740371
H	6.8881738087	-1.4865071678	-4.6235062608
H	7.0152624758	-0.3460114041	-5.8459677315
H	6.6111866406	0.1333534217	-4.2909032215
H	5.5114252383	-0.7330193801	-5.2135185742
N	4.0445208393	4.5217233310	-1.4214833860
H	3.5406574390	4.4620915911	-2.3141209536
H	4.9588815162	4.0637470215	-1.5133361920
H	3.4990816331	4.0530693003	-0.6886157336
H	4.1794622781	5.5079853529	-1.1698615343
N	0.3341108028	2.5180351110	-0.8975747715
H	0.2259950283	3.5345578957	-0.8015096631
H	0.9085344289	2.3114512022	-1.7231626056
H	-0.5901562011	2.0843237497	-1.0064806240
H	0.7920698498	2.1418085864	-0.0591460998
N	2.3702744151	-1.2688150536	0.5985978838
H	2.1040709743	-1.4258804158	-0.3805355584
H	1.7989306785	-1.8653036385	1.2085178689
H	2.2156340379	-0.2832453166	0.8414438666
H	3.3624617103	-1.5008309963	0.7249644046
N	-1.4089922021	-1.8530885118	-1.3544967162
H	-0.5546264352	-1.6469352025	-1.8853413705
H	-2.0842692963	-1.0904056794	-1.4831451859
H	-1.1794192881	-1.9424489288	-0.3577286805
H	-1.8176529567	-2.7325640355	-1.6917721451
N	5.8411143992	0.3102684162	-0.5620325919
H	5.2389223866	-0.1595127503	-1.2482588907
H	6.3635644406	1.0637105875	-1.0242073792
H	6.5002100243	-0.3687056426	-0.1635069232
H	5.2617601588	0.7055810124	0.1878421573
N	1.5097301137	5.3322953867	-5.4583844757
H	1.4113741353	4.3122679652	-5.3943043813
H	2.3508778869	5.5616302831	-6.0007041364
H	1.5925221720	5.7281138651	-4.5146108104
H	0.6841461648	5.7271684399	-5.9239185123
N	3.5327534911	-4.5941176881	-4.7529978553
H	3.5380396804	-3.6708078226	-4.3038797793
H	2.9735787620	-4.5531150878	-5.6131589417
H	3.1242975543	-5.2842597936	-4.1118245979
H	4.4950979728	-4.8682871490	-4.9831276648

N	5.4183946721	-2.4984059535	-8.5969527640
H	4.4857409723	-2.9274010802	-8.6158301343
H	5.9838718347	-2.8747455504	-9.3669130045
H	5.8745912865	-2.7104328154	-7.7018746764
H	5.3293736866	-1.4810447859	-8.7031932593
N	6.5932132201	-4.3194845562	-2.0895553514
H	7.3578048355	-4.6123013600	-2.7091472160
H	6.3150983153	-3.3583097310	-2.3198372233
H	6.9091576915	-4.3598129728	-1.1134466311
H	5.7907927830	-4.9475144460	-2.2157909387
N	-2.7195481541	0.9894213556	-2.0499560307
O	-3.1808843988	-0.1211482574	-2.3611182311
O	-3.1355184199	2.0149718604	-2.6140773040
O	-1.8422409375	1.0744405322	-1.1746718524
N	4.5756669142	-1.7085091499	-2.6490414215
O	3.9239103951	-0.9034322715	-1.9634157433
O	4.0164175604	-2.3474059725	-3.5557215863
O	5.7866737619	-1.8746893394	-2.4279867569
N	3.2129480670	5.1163599815	-8.0739741269
O	3.6757304779	4.3483235402	-8.9336079884
O	2.2254659441	5.8219839380	-8.3385337608
O	3.7376482014	5.1787725166	-6.9497797265
N	-0.8866572074	0.1009280783	-4.4308064405
O	-0.2247467353	0.8536269861	-3.6970920600
O	-0.7118630636	-1.1278228135	-4.3794628515
O	-1.7233624967	0.5769804456	-5.2158650420
N	3.3399130394	-6.3344752763	-2.0507078940
O	4.4743152457	-5.8391116732	-2.1544597423
O	2.5597882214	-6.3078053093	-3.0169955801
O	2.9856353658	-6.8565092666	-0.9806674983
N	3.0006936642	2.5961111660	-3.5760809188
O	3.2002883591	3.7997621539	-3.8093279298
O	3.6213671273	1.7288052829	-4.2129190994
O	2.1804248460	2.2597657905	-2.7059950266
N	-2.6264804444	-1.2551088557	-7.6279980292
O	-2.3925598500	-2.3167689894	-7.0270181403
O	-2.0104892310	-0.9836520574	-8.6719730972
O	-3.4763929365	-0.4649048841	-7.1850024936
N	0.9761569773	5.3345452933	-2.3019997613
O	1.7561903928	6.0767188811	-2.9214706219
O	1.1970557571	5.0563683614	-1.1116891991
O	-0.0247760237	4.8705482638	-2.8728399224
N	1.5760322956	-2.9454907652	-2.1277288249
O	1.2220723222	-1.8012219085	-1.7985191171
O	1.1553119497	-3.4460083579	-3.1838985276
O	2.3507132384	-3.5892425475	-1.4007682448
N	2.1069282146	-3.8755018623	-7.9075305670
O	1.9568375417	-4.3933691133	-6.7884665781
O	3.2317160107	-3.8494015870	-8.4340180790
O	1.1322303068	-3.3837344907	-8.5001075208
N	5.8813745787	-2.6787744274	0.5755248511
O	6.8675340257	-1.9539163252	0.3631850582
O	5.9948111921	-3.9144128145	0.5176644861
O	4.7817776330	-2.1679937312	0.8457252267
N	0.4710245993	-3.6111178174	0.9683646923
O	1.4487198717	-3.5898375043	1.7343131327
O	-0.2401828390	-2.6007007966	0.8408525967
O	0.2045365506	-4.6428159820	0.3299278335
N	-1.5143264309	4.7233497071	-7.2797164301
O	-1.5976668849	4.5970309305	-8.5126462117
O	-2.2097035983	4.0136052482	-6.5342425478
O	-0.7356081827	5.5594136156	-6.7922601384
N	1.2897981205	1.9856557350	-6.5530275405
O	2.4787329448	2.0869324104	-6.8982737273
O	0.8014311412	2.8069008971	-5.7592568002
O	0.5892297115	1.0631331549	-7.0015524551
N	2.9469746073	1.8125161166	0.7522324858
O	3.9776889346	1.1651555684	1.0003659319
O	1.8336223302	1.2715923728	0.8564731761
O	3.0296126236	3.0008013651	0.3998580657
N	-2.0431556617	-4.2748671704	-3.4161346744
O	-2.7846730598	-3.5443403051	-4.0939992318
O	-2.0792201913	-4.2166900269	-2.1758392738
O	-1.2655731080	-5.0635718142	-3.9785659702
N	6.3462687808	2.4843021327	-2.9011315139
O	6.3814255261	3.3561749438	-3.7852241083
O	6.6046976572	1.3025852659	-3.1835809312
O	6.0526829226	2.7941464380	-1.7345885632
N	3.1478604682	-0.9991740093	-6.8019981951
O	4.1305663781	-0.8327124587	-6.0606497226
O	2.0739278483	-1.4053015986	-6.3279232852
O	3.2390872515	-0.7595077777	-8.0174225559
N	6.3234401154	0.5420848680	-8.1498668298
O	7.0901474108	-0.1972410659	-7.5106681145
O	5.7486891598	0.1128155584	-9.1639709220
O	6.1314836212	1.7106810522	-7.7749611511
N	0.7599065874	1.8919863799	-10.4788689420
O	1.1861743936	2.9643647471	-10.9385626507
O	1.4684421358	0.8725898106	-10.5215997519
O	-0.3748976806	1.8390045393	-9.9764440190
N	6.6375101131	-3.7196018723	-5.2234153787
O	6.1511930964	-4.8018947693	-4.8557800221
O	6.3658474593	-3.2665806729	-6.3476880797
O	7.3954903937	-3.0903296682	-4.4667774251

n=22 OPLS/AA

N	6.4810696318	-0.6955202703	5.2261572903
H	6.1082196990	-0.2268764464	4.3921357129
H	5.9078504418	-1.5199671476	5.4404976556
H	6.4588633015	-0.0469756815	6.0218534127
H	7.4493447219	-0.9882613494	5.0501415675
N	0.8372866461	-4.0395356243	5.3872394496
H	0.7869941267	-3.2805049104	6.0768619082
H	0.2180396655	-4.8059314539	5.6760391981
H	1.8027344223	-4.3837965998	5.3269858503
H	0.5413783209	-3.6879087937	4.4690715133
N	-2.9846342958	5.3551358482	6.2452980577
H	-2.8720948010	5.3103756544	7.2648898803
H	-3.9763511832	5.4899059154	6.0160180328
H	-2.6522890342	4.4790380351	5.8254948026
H	-2.4378020553	6.1412237444	5.8747905082
N	-6.2343720782	1.3162364403	3.3583917522
H	-6.1713820703	1.4298812991	2.3398863754
H	-5.2966503072	1.3914284073	3.7698024153
H	-6.6306977673	0.3943975842	3.5760488117
H	-6.8387581066	2.0492385815	3.7478284144
N	3.2398668547	7.4971108597	7.8337271122
H	2.3593371316	7.5960275666	7.3149603155
H	3.0646126596	6.9971840746	8.7132689600
H	3.6223939372	8.4275240749	8.0392503573
H	3.9131228329	6.9677078191	7.2674283108
N	-1.4070630632	-2.9897734692	8.7227322355
H	-1.4113679193	-2.4342516086	7.8592433657
H	-1.1641121835	-3.9627662913	8.5025175137
H	-2.3382137380	-2.9587818485	9.1542799899
H	-0.7145584161	-2.6032935875	9.3748872316
N	2.6292586143	-1.0451936243	3.2447902182
H	3.3396578215	-0.7588501299	2.5609945856
H	1.7246984036	-1.1571585828	2.7720647600
H	2.5475919269	-0.3274245713	3.9744296241
H	2.9050869970	-1.9373409345	3.6716712371
N	-5.3013033374	-3.4272215161	6.8610541758
H	-5.1857619448	-2.5547487121	7.3898996075
H	-6.1099844994	-3.9440725876	7.2259300911
H	-4.4556790511	-4.0013087465	6.9589262470
H	-5.4537877418	-3.2087551684	5.8694612728
N	-2.7304694694	0.0144945324	1.8667108072
H	-2.1368196277	0.5631977651	2.4997516633
H	-2.1887294387	-0.7683402551	1.4821043590
H	-3.0581744958	0.6133970432	1.0997944348
H	-3.5381537374	-0.3502758891	2.3851933884
N	2.5576096880	1.4228095420	-0.1521662521
H	3.2041582109	0.8283215343	0.3796185859
H	2.6522517937	1.2204048372	-1.1543188815
H	2.7803039093	2.4102145869	0.0200954245
H	1.5937254677	1.2322966306	0.1459403809
N	-4.3968840348	1.4026610228	7.0524594695
H	-5.2141649482	1.8971025109	6.6758753206
H	-4.6997953928	0.5247330592	7.4903200470
H	-3.9330411280	1.9924534732	7.7533375954
H	-3.7405354663	1.1963555297	6.2903045482
N	-1.7707914322	3.9730695836	1.1097663093
H	-0.8394818697	4.1146526501	0.7012900778
H	-1.6884619210	3.4043223143	1.9606382625
H	-2.3719515387	3.4899116211	0.4319754612
H	-2.1832694924	4.8833918868	1.3451610379
N	-0.0567827778	1.5160213305	5.1206398453
H	0.5309005903	0.6849716882	5.2556244692
H	-0.9397090424	1.3974247142	5.6311403245
H	-0.2534952645	1.6372726599	4.1202210147
H	0.4351731779	2.3444154501	5.4755737045
N	-5.5108767253	5.4090245264	2.4450742276
H	-6.0411349081	4.8387558084	3.1142946296
H	-4.7697065961	5.9179726737	2.9409303642
H	-6.1421173093	6.0798145869	1.9914096555
H	-5.0905486044	4.7995544812	1.7336629128
N	3.5646204909	3.3344561167	3.3073853514
H	2.8807102946	3.9491973358	2.8506620494
H	4.1818282422	2.9223212147	2.5978544502
H	3.0713100224	2.5860979035	3.8082249371
H	4.1246327383	3.8802086114	3.9727995242
N	-2.7581080600	-3.2260761051	3.8134150380
H	-2.4464109045	-2.3941835898	4.3282313389
H	-3.7840063966	-3.2394475863	3.7735591481
H	-2.4231665353	-4.0703281079	4.2922555698

H	-2.3788481000	-3.2003443263	2.8596145963
N	2.7241333684	1.7065637291	9.3813689128
H	2.2894345260	1.8292592829	8.4592958055
H	2.1748089983	1.0365174506	9.9322912364
H	2.7520454781	2.6094587579	9.8694641956
H	3.6802440476	1.3510195444	9.2644235156
N	-1.0298847703	0.8882878003	9.2052922574
H	-0.4441115476	0.5132781366	9.9605892727
H	-1.9268099544	0.3888389832	9.1876633415
H	-0.5505540016	0.7628324935	8.3059933615
H	-1.1980630073	1.8882012228	9.3669237897
N	5.3950713734	3.4332038638	7.5036674178
H	5.6445910295	4.0884284490	6.7535629040
H	5.1796220218	3.9583876245	8.3592363980
H	6.1831416461	2.7992485227	7.6805328890
H	4.5729310393	2.8867514971	7.2213367498
N	0.8265511231	5.9732373058	4.3902897343
H	-0.1693270195	6.0836924237	4.1660967601
H	1.3644510475	5.8771178296	3.5210030134
H	0.9563777057	5.1341723161	4.9676548923
H	1.1547017887	6.7979667613	4.9064040530
N	3.7733281120	-1.9662973405	8.1271090410
H	3.5263662998	-1.2511391317	7.4329947153
H	4.7489585917	-1.8367870436	8.4196956703
H	3.6613053809	-2.8988721051	7.7123913777
H	3.1566819352	-1.8783903852	8.9433537249
N	0.3241581844	5.4437285393	9.6474300305
H	-0.0178199893	6.3738102837	9.3786643796
H	-0.4746058689	4.8252156527	9.8308761583
H	0.8982275772	5.5219315757	10.4951116579
H	0.8908306854	5.0539575509	8.8850676644
N	-2.7062177311	6.3206610731	3.1850214982
O	-2.7779116944	6.1999878344	1.9507946730
O	-1.6379463868	6.0802852441	3.7715653642
O	-3.7027959144	6.6817104314	3.8327049788
N	-0.3205843125	-2.2679293144	2.2288525441
O	0.4933629876	-1.4064026010	1.8569931278
O	-0.0063187577	-3.0766649838	3.1177854376
O	-1.4487980757	-2.3207204010	1.7117786508
N	1.1632254648	-1.0347619900	10.3774293464
O	1.0516549200	0.1713480766	10.6528715941
O	2.2845428467	-1.5190762097	10.1513652481
O	0.1534778148	-1.7565584178	10.3280511573
N	0.1858728088	1.5817561841	1.9310701772
O	-0.8285322415	1.7779550800	2.6206398242
O	1.3081648019	1.5807567393	2.4634963169
O	0.0779857792	1.3865565758	0.7090734066
N	-3.2446019062	2.6248818091	4.2955598040
O	-2.5036830306	2.9657844719	5.2324923017
O	-3.8520070865	1.5425206042	4.3462656232
O	-3.3781157089	3.3663409479	3.3079206921
N	3.3739599586	0.9823425240	5.8911536234
O	3.7482830670	-0.1602808778	6.2031021497
O	2.8979241980	1.1937261575	4.7634458487
O	3.4756726927	1.9135830420	6.7069135285
N	4.5195526766	5.5303528791	5.4510083552
O	3.5590198861	5.9902421652	4.8115581900
O	4.9125896335	6.1044778445	6.4800469286
O	5.0870489670	4.4963377952	5.0614196335
N	-2.2725665980	3.7874866168	9.0618166278
O	-3.0488073250	2.9441273658	8.5830542459
O	-2.3933832721	4.9858978815	8.7581199323
O	-1.3755084747	3.4324343173	9.8442763350
N	-6.1581201757	3.7889202894	5.1788364364
O	-5.4788392875	4.7529442635	5.5690327499
O	-6.2277656226	2.7583888918	5.8688889424
O	-6.7677561080	3.8554277665	4.0985867471
N	-4.0527524655	-1.3077227657	8.7511223812
O	-3.2637312581	-0.4307043303	9.1400980993
O	-4.9567364409	-1.0186874037	7.9496927569
O	-3.9377896049	-2.4737775017	9.1635766195
N	4.8879548023	0.7100969080	2.0493737219
O	5.4699394072	0.2604727328	3.0504481105
O	5.0370689674	1.9002994859	1.7265723896
O	4.1568554436	-0.0304820909	1.3711001196
N	-4.5747528547	2.5427370387	0.6205675772
O	-5.5513059435	1.8484448128	0.9481653195
O	-3.5011046269	1.9988138444	0.3132486016
O	-4.6718480717	3.7809534557	0.6002887943
N	-2.0651324705	-0.8264037948	5.8118649942
O	-1.9850543591	0.2221430690	6.4730418853
O	-2.0382191943	-0.7816640699	4.5707800078
O	-2.1721239442	-1.9196912637	6.3917735563
N	1.7129565132	4.3991609169	1.0311202124
O	1.7468743349	4.8914044442	2.1711043361
O	0.6573369941	4.4200990757	0.3767144244
O	2.7346590330	3.8859788177	0.5455414860
N	3.4001600037	4.9758163543	10.0110633172
O	4.5205533329	4.7067253864	9.5470301829
O	3.0055289096	6.1533382098	10.0380168001
O	2.6743971843	4.0673847354	10.4481433206
N	-5.5388154190	-1.7436348980	3.8595398711
O	-5.3089546589	-2.8688406000	4.3329256230
O	-6.5687182104	-1.1308601483	4.1863812048
O	-4.7387727436	-1.2312035331	3.0593121414
N	1.2218838289	3.8253927200	7.0029855204
O	1.7552550956	4.8164351699	7.5286925351
O	1.0876581894	3.7736416691	5.7691608905
O	0.8227378803	2.8861005648	7.7111037058
N	5.9628626820	0.3423189575	8.1519454355
O	6.1534692600	-0.8824456919	8.2334230214
O	6.5639424721	1.0086492334	7.2930362667
O	5.1711756766	0.9007537805	8.9293776444
N	3.9225547363	-3.3457603860	5.4035880684
O	3.4167023734	-3.9409586415	6.3694401971
O	5.0325366355	-2.8008301917	5.5219484402
O	3.3184247135	-3.2954922844	4.3193746950
N	0.1803441536	7.4710489047	7.0790017505
O	0.2538218791	7.6857574073	8.3002794396
O	-0.8381984324	6.9445305625	6.6011237885
O	1.1254097751	7.7828589955	6.3356014251
N	0.8963757747	-1.0525463305	6.7511930549
O	0.8431588792	-0.0627337437	7.4998297598
O	0.9822874431	-0.8974796696	5.5217251103
O	0.8636809756	-2.1974264999	7.2320246816
N	-1.9841819932	-5.0342346345	6.4165375992
O	-1.7821147991	-5.1558300869	5.1969469687
O	-1.0360760763	-5.1187209108	7.2146448290
O	-3.1343560301	-4.8281527398	6.8380213391

n=23 OPLS/AA

N	4.2761875332	-10.7214788497	11.8748013489
H	4.7364024484	-10.5037946846	12.7664581964
H	4.7837414815	-11.4796319356	11.4038156024
H	4.2760132080	-9.8867041055	11.2769831322
H	3.3085934433	-11.0157844611	12.0519493330
N	4.2019267455	-2.5336664589	13.3977604788
H	3.6144900094	-3.3662573349	13.2714805842
H	3.9702865605	-2.0836568695	14.2911074884
H	4.0320404703	-1.8750714568	12.6285876758
H	5.1908893696	-2.8096809852	13.3998660438
N	1.3981836027	-7.5109053765	1.5693004368
H	0.6018644553	-7.7826897422	2.1577264488
H	2.0524752442	-8.2985774231	1.4937366873
H	1.8774492647	-6.7071983337	1.9919053808
H	1.0609446710	-7.2551562716	0.6338338033
N	0.9551468185	-3.9876735823	4.6919807797
H	0.4821099126	-4.5286091776	3.9585907982
H	1.5334704533	-4.6162651698	5.2617606275
H	0.2537032726	-3.5333167265	5.2884473115
H	1.5513031749	-3.2725037821	4.2591236674
N	-2.2114429336	-5.5140087509	7.1251696541
H	-2.0482707024	-6.2202911993	7.8523395267
H	-1.7005319196	-5.7750955368	6.2736778389
H	-1.8811022026	-4.5997753202	7.4557566469
H	-3.2158667510	-5.4608736351	6.9189053122
N	4.8373161771	-10.9770033657	7.8322412146
H	5.2463355009	-11.4046036126	8.6713462850
H	3.9689911044	-11.4670101152	7.5870073690
H	5.5022166929	-11.0481518235	7.0530873626
H	4.6317218085	-9.9882483278	8.0175246591
N	0.5701928848	-8.4660018189	6.0013895827
H	0.6939417127	-7.8082302320	6.7800158503
H	0.6434685798	-9.4284475191	6.3514804739
H	1.2979843784	-8.3020829238	5.2959211839
H	-0.3546230111	-8.3252459601	5.5781415811
N	6.6734450019	-3.3761278619	5.3354554831
H	7.1074568435	-2.7067390461	5.9818210394
H	7.3827073326	-3.7316023046	4.6836736544
H	5.9276101706	-2.9078114332	4.8075865117
H	6.2760060838	-4.1583580119	5.8687413567
N	3.9639882709	-6.3669334228	7.5747533289
H	3.5364087123	-6.1166714622	8.4740757237
H	4.1672626327	-7.3732715562	7.5606898805
H	4.8365178244	-5.8397757815	7.4521461366
H	3.3157634978	-6.1380146475	6.8121024508
N	9.6278447754	-4.1266413307	10.1350226309
H	8.6924755694	-4.4169206379	9.8267070948
H	10.2975306410	-4.8841454235	9.9562963704
H	9.6074557236	-3.9179064165	11.1401343392
H	9.9139162567	-3.2875931276	9.6169524189
N	5.3586114704	-5.1872047728	2.4041433695
H	6.3286668786	-5.2493534226	2.0734378389
H	5.0609984727	-4.2045366581	2.4090997300
H	5.2962317074	-5.5685050632	3.3554342702

H	4.7485497676	-5.7264240076	1.7786013169
N	6.2570777912	-1.9775515136	9.5114750853
H	6.8495614746	-1.8613871261	8.6809902766
H	5.7873648756	-2.8897138880	9.4719242936
H	5.5528691175	-1.2307646996	9.5366767194
H	6.8385162741	-1.9283402277	10.3563082428
N	0.4338233634	-11.4130564291	9.3868042064
H	-0.0817419385	-10.5629228491	9.1304813373
H	1.1727199435	-11.5867859144	8.6953675895
H	-0.2111965507	-12.2116806296	9.4064057592
H	0.8555114969	-11.2908354953	10.3149618899
N	6.6833454017	-5.9046345001	11.9591257693
H	5.9722081089	-5.2511021728	11.6106787208
H	6.2975266488	-6.4443609602	12.7427544592
H	7.5051536231	-5.3781150388	12.2779423754
H	6.9584925334	-6.5449591920	11.2051271824
N	8.1262208880	-8.9503764316	7.6064661324
H	9.0612483730	-8.5839337707	7.8201937071
H	7.9188342843	-9.7419266756	8.2266868134
H	8.0967898691	-9.2643472766	6.6293317263
H	7.4280119364	-8.2112976464	7.7496524910
N	8.1238272217	-8.5339379602	2.7495974962
H	7.1471017345	-8.3671358567	2.4804869639
H	8.6049609264	-7.6344735400	2.8666820828
H	8.5922580866	-9.0798230405	2.0169191711
H	8.1509871881	-9.0543192412	3.6343015047
N	8.9754071427	-9.5278823440	11.8236338927
H	9.2523441389	-8.9765219797	11.0029367827
H	8.4673814831	-10.3659426073	11.5173666732
H	8.3683997213	-8.9613269335	12.4276144233
H	9.8135034971	-9.8077373186	12.3466168925
N	2.7504364685	-1.9845150515	7.5767191065
H	3.1388576950	-1.2361710817	8.1626767426
H	3.1853854215	-1.9520410065	6.6472033561
H	1.7359967165	-1.8554352441	7.4846000695
H	2.9415064194	-2.8944121449	8.0123968284
N	0.2654366709	-2.7567663901	10.4276410890
H	0.2174721032	-2.9181912966	11.4404971980
H	0.5017132890	-3.6354156621	9.9518433358
H	0.9866915557	-2.0541274460	10.2268291634
H	-0.6441303109	-2.4193313129	10.0913956454
N	4.3037893436	-9.9051771530	4.1352727972
H	3.8913268291	-9.6002654852	3.2458124853
H	5.1609192041	-10.4391918879	3.9498237463
H	3.6331333849	-10.4981778038	4.6380713031
H	4.5297775544	-9.0830731383	4.7073827880
N	9.5636553246	-5.9255718443	5.9889759957
H	8.6167825497	-6.3220670829	5.9675383783
H	10.1478098066	-6.4692325911	6.6350651683
H	9.5191147538	-4.9500608793	6.3061983449
H	9.9709132662	-5.9609272100	5.0471020703
N	0.0299191546	-7.3677482449	10.7826632996
H	0.1420674211	-6.5768161198	11.4277099937
H	-0.1212931171	-8.2292190055	11.3204715024
H	0.8769300979	-7.4656206511	10.2106239845
H	-0.7780276743	-7.1993364328	10.1718483462
N	3.1809218485	-7.0916042211	12.3345016955
H	4.0603426299	-7.2536352154	12.8390818613
H	2.5549614629	-7.8938932365	12.4713980678
H	3.3769297582	-6.9773903567	11.3331168461
H	2.7314543996	-6.2414982335	12.6944104982
N	10.4386840812	-7.3468094103	9.0739054948
O	10.8801174162	-6.2706380595	9.5098065676
O	10.0184915906	-8.2104418143	9.8616841773
O	10.4174432196	-7.5593485283	7.8502247543
N	-0.7497603947	-2.7127787295	7.4042867764
O	-1.4968466505	-3.1385226026	8.3007365475
O	-0.8957562533	-3.1062143811	6.2351368680
O	0.1433224384	-1.8935985455	7.6769871333
N	1.1520918858	-10.0886533093	12.1018617795
O	1.6080205498	-11.2194291288	11.8641791965
O	1.8525587177	-9.2473160064	12.6888163528
O	-0.0043045411	-9.7992145597	11.7525895081
N	-1.5791034420	-8.3273076875	8.2782127653
O	-1.9731449582	-8.3738287711	7.1011040264
O	-1.8848731087	-7.3620910668	8.9978513529
O	-0.8792916956	-9.2460039643	8.7356832848
N	7.2197567845	-10.5273976366	4.9589646035
O	8.2844146991	-9.8983590525	5.0765736819
O	6.6130375697	-10.9161804204	5.9707737023
O	6.7618177160	-10.7676536304	3.8295455172
N	6.1612454811	-8.4610004794	13.5920392221
O	5.7056431725	-9.6060240705	13.7481334985
O	7.3788329865	-8.2966257327	13.4090651246
O	5.3992596709	-7.4803508454	13.6189190648
N	0.9754625851	-5.3121725356	8.1313589119
O	0.5992678509	-5.0407598575	9.2836742294
O	0.6277453392	-6.3796415557	7.5997382588
O	1.6993751480	-4.5161155526	7.5106637478
N	1.4491209271	-4.4828172790	12.8342083715
O	2.6185326401	-4.6222805335	13.2292326201
O	0.8183148344	-5.4621020611	12.4028119184
O	0.9105148732	-3.3640683416	12.8705806052
N	4.2087462735	-4.4639145319	10.2002260818
O	4.9175178138	-4.1480594458	11.1702242154
O	3.9920805674	-3.6410764074	9.2952336490
O	3.7166400432	-5.6026086592	10.1352203287
N	7.6363459779	-3.1190173988	12.4906447649
O	6.7167591594	-3.0926152709	13.3253189061
O	8.4698761936	-4.0397293510	12.5137703209
O	7.7224026499	-2.2247068545	11.6328443773
N	8.5688890880	-5.4859815429	3.0288815112
O	9.4721580343	-6.2829693829	3.3320868946
O	7.8673006246	-5.6948934317	2.0253141933
O	8.3672084428	-4.4800810042	3.7292440096
N	-0.5916008347	-6.5431051405	3.7853084932
O	-1.0551244027	-6.2767911227	4.9065759265
O	-0.4835975811	-7.7263318797	3.4229168658
O	-0.2360802342	-5.6261916809	3.0264320766
N	5.8925569961	-8.2392390922	9.9098869307
O	7.0253157219	-7.8647363307	10.2557528899
O	5.6393923860	-8.4234189445	8.7078039996
O	5.0129621722	-8.4295621546	10.7661045918
N	6.1325730039	-7.0561874746	5.2359398417
O	7.3626435601	-7.2240146356	5.2781107052
O	5.3681187390	-7.9822478577	5.5538073528
O	5.6669563377	-5.9622990498	4.8759011772
N	4.4734591876	-8.0745864938	1.5808324451
O	3.6294722211	-8.9729852822	1.7344017392
O	4.1844082939	-7.0550669164	0.9327218657
O	5.6064979600	-8.1957073802	2.0753741284
N	6.9409451847	-11.4627224182	10.1824621940
O	5.7932110314	-11.9230034261	10.0647098349
O	7.5466345020	-11.5728799127	11.2613614888
O	7.4829904570	-10.8922834566	9.2213144846
N	3.4036449037	-0.8189284061	10.3443051111
O	4.0833352127	-0.5173752692	9.3492650615
O	3.9371642403	-0.8847623707	11.4641447950
O	2.1904342814	-1.0546477682	10.2195053764
N	6.9908814639	-5.2328999670	8.2950196515
O	7.6717686289	-6.2123005635	7.9483424947
O	6.0907561465	-4.8079088388	7.5519349558
O	7.2101197928	-4.6784900523	9.3847823813
N	2.6634885602	-8.9407114923	8.9907174509
O	2.2279281985	-7.9461246824	9.5941262342
O	2.8429101940	-10.0078740380	9.6006271896
O	2.9196274943	-8.8681356981	7.7773979522
N	3.8761845210	-2.4783087733	4.1680664317
O	2.7236269215	-2.4099813651	3.7098522283
O	4.8180796716	-2.8164696447	3.4322005055
O	4.0868471395	-2.2084750927	5.3621475226
N	2.6208613602	-6.5851239853	4.2815675003
O	2.7835131146	-5.9715816818	3.2137996605
O	2.6294892170	-5.9683940080	5.3598010585
O	2.4495816112	-7.8153972564	4.2711017734
N	8.8149656993	-2.5543339981	7.5354431547
O	9.1899426571	-3.5000042505	6.8226192173
O	7.7207230339	-2.0082346030	7.3176672356
O	9.5342319859	-2.1547628191	8.4660437605
N	1.9920082223	-11.4468259965	6.6129202602
O	2.3840684352	-12.0850510974	7.6038669898
O	0.8413969616	-10.9792585625	6.5907316994
O	2.7505598808	-11.2761681922	5.6441613115

n=24 OPLS/AA

N	-2.2628452796	-1.7725203556	5.2521464798
H	-2.8114331071	-2.0178609865	6.0846700338
H	-1.8024723562	-0.8667955984	5.4003157110
H	-2.8853958050	-1.7108811543	4.4379800495
H	-1.5520803842	-2.4945439222	5.0856209358
N	2.3670967161	-2.8895526370	2.6993725735
H	2.0036649616	-3.0213538814	1.7481723373
H	3.3584940550	-3.1552806701	2.7268428162
H	1.8368677426	-3.4777601657	3.3529050233
H	2.2693597512	-1.9038159593	2.9695691907
N	-1.4738723608	0.9228270686	0.2797953910
H	-0.9811245359	0.2773355520	-0.3485169362
H	-1.0836366954	0.8438933752	1.2262203923
H	-2.4724695805	0.6848884068	0.3003941753
H	-1.3582581513	1.8851903120	-0.0589166791
N	-1.9708279935	-6.4800298600	5.9636684028
H	-1.2909349747	-6.9307014000	6.5872682293
H	-2.1643663630	-5.5289016871	6.2985307385
H	-2.8415661612	-7.0240852939	5.9563027994
H	-1.5864438129	-6.4364314978	5.0125724515

N	4.1653803676	6.4294445352	3.1761034077
H	3.7869453530	5.5098686123	3.4318438215
H	3.3928903249	7.0655576148	2.9462034961
H	4.7819447842	6.3311626958	2.3609810093
H	4.6997406396	6.8111883223	3.9653855528
N	1.8266642545	-7.0175987025	3.8166071788
H	1.1896356765	-6.4440403535	3.2513979178
H	1.3576482067	-7.2930162037	4.6874710215
H	2.6696852588	-6.4751559981	4.0386590263
H	2.0896872555	-7.8581816959	3.2889001994
N	1.4495910147	5.3563449634	0.8699421007
H	1.1944552703	4.5342970814	1.4297448599
H	1.4401887611	6.1924924377	1.4657639568
H	0.7739381054	5.4730735225	0.1056757115
H	2.3897816735	5.2255160113	0.4785844198
N	4.4189489846	3.3546636834	7.8572519512
H	3.5503259101	3.0926250218	7.3765589183
H	4.6602019698	4.3249050596	7.6234499957
H	5.1776855559	2.7314541472	7.5569783870
H	4.2875816566	3.2696702497	8.8720200358
N	-0.1492917064	4.9068952978	6.2421397802
H	-0.1200921516	5.5034718329	7.0772924138
H	-0.9350508676	5.1914153586	5.6455950953
H	-0.2716043577	3.9275397670	6.5252261341
H	0.7295805796	5.0051548136	5.7204462912
N	1.4984474282	-0.1105674138	11.2263912934
H	0.6611257740	-0.1824872226	11.8162693198
H	1.2264640013	0.1805289283	10.2800707238
H	1.9656736377	-1.0238096116	11.1825292223
H	2.1405254844	0.5834981808	11.6266964823
N	1.2241358309	0.9294637444	5.4852941354
H	1.6049436927	0.3934656649	4.6966703159
H	1.9892142156	1.4079738133	5.9751082477
H	0.7480426331	0.2919757430	6.1342778326
H	0.5543431530	1.6244392343	5.1351193775
N	6.3823613231	-2.1067006500	4.7324050637
H	5.6875916462	-1.6740281572	4.1124649797
H	7.2615391997	-2.2419283696	4.2195752570
H	6.0322954364	-3.0156594121	5.0571870951
H	6.5480183334	-1.4951862398	5.5403923192
N	-2.7312675742	2.3060593658	3.5522113696
H	-3.2062612068	1.5016975653	3.1260432801
H	-3.3503701367	2.7406818527	4.2465095739
H	-2.4990348558	2.9916280017	2.8239949635
H	-1.8694045602	1.9902292602	4.0122972459
N	-2.2174980217	3.4658479887	8.9433211772
H	-1.5519285879	4.1982926694	9.2167772358
H	-1.7695656962	2.8288924854	8.2740835482
H	-3.0407805459	3.8971136261	8.5069138453
H	-2.5077166085	2.9390938870	9.7755103458
N	0.0169725199	-4.7015107364	9.0853869580
H	0.8272936859	-4.4440564563	9.6610022803
H	-0.1140668400	-5.7194532085	9.1146528971
H	0.1779039724	-4.4048892421	8.1156693286
H	-0.8232399494	-4.2376437880	9.4502238867
N	2.6669200966	1.4150683046	1.6585519518
H	3.0730821806	1.1077507213	2.5500811785
H	2.3829744562	0.5943311265	1.1108293548
H	3.3649519655	1.9532169125	1.1318802229
H	1.8466721797	2.0049741589	1.8414179192
N	4.3160912658	-0.6682100540	8.2915026316
H	4.2572818158	0.0670549705	9.0057595111
H	5.2062466903	-0.5845407901	7.7866685403
H	3.5369328104	-0.5664497685	7.6305998628
H	4.2639036892	-1.5889039118	8.7429833080
N	-1.9751958478	6.6178900782	2.8516456517
H	-2.2401028362	5.9734365082	3.6057941658
H	-1.1505736735	7.1587349813	3.1375123094
H	-2.7544676715	7.2581015605	2.6590315124
H	-1.7556394678	6.0812866350	2.0042453538
N	3.1475486215	-4.4525516332	7.2441771005
H	3.5090496945	-4.0859777189	8.1325332437
H	2.4246287814	-3.8199000033	6.8817222684
H	2.7421526879	-5.3833236695	7.3976472199
H	3.9143636743	-4.5210047842	6.5648065352
N	-1.8646010198	-0.6722511346	9.4073882610
H	-0.9026291777	-0.9720852642	9.2100414407
H	-2.1615213679	0.0117333355	8.7015283337
H	-1.9047653258	-0.2421824740	10.3388727213
H	-2.4894872708	-1.4864704278	9.3791103560
N	5.7370437490	2.8615272024	4.1274732500
H	5.9239604482	3.2378143299	3.1906134724
H	4.8064080437	2.4280326590	4.1427817518
H	5.7712149672	3.6231051370	4.8152718408
H	6.4465917191	2.1571570504	4.3612250225
N	-1.4134609337	-3.2085656708	1.2868696128
H	-1.6885458110	-4.1762825482	1.4920243182
H	-1.0684990025	-2.7616745137	2.1444967143
H	-2.2257463741	-2.6894733661	0.9333571426
H	-0.6710528154	-3.2068331978	0.5776004761
N	2.6895502572	7.7216447314	6.9477133691
H	1.8557107497	7.3727552349	7.4347697238
H	2.4788609117	7.8391709023	5.9496991987
H	3.4572215709	7.0488633949	7.0585355308
H	2.9664069843	8.6257890536	7.3478494975
N	1.3230520172	3.9774751766	11.0414492387
H	1.3330334254	3.6918969009	10.0552537624
H	1.2011142783	4.9951776717	11.1018438133
H	2.2110195065	3.7123336954	11.4835456727
H	0.5470408681	3.5104921601	11.5251527460
N	4.6857161325	4.1659087272	1.0961431669
O	4.3909045672	3.0332205845	0.6800643005
O	5.6110831520	4.3061760724	1.9128676382
O	4.0551601705	5.1583303238	0.6954972396
N	0.4000303358	6.1922160896	9.1493896257
O	1.1262937400	6.4705489883	10.1179401800
O	-0.5086856768	5.3546619828	9.2749134780
O	0.5824830911	6.7514377480	8.0553143383
N	0.1664441396	-0.5808865245	2.9856998323
O	1.3709330448	-0.4792183529	3.2718605755
O	-0.4633953272	0.4130099922	2.5875919688
O	-0.4082057615	-1.6764520948	3.0976470428
N	-0.8890502531	-6.0275277029	2.5947765809
O	-1.9020134708	-5.6240119801	1.9997148261
O	0.2393423788	-5.6941577858	2.1965147604
O	-1.0044797602	-6.7644139358	3.5881009559
N	-3.4279509090	-0.8129344975	1.9676892554
O	-3.6211593401	0.4102296454	2.0654537293
O	-3.1612388984	-1.3132136446	0.8624275718
O	-3.5014545475	-1.5358200754	2.9751872762
N	0.9627541895	-1.4621315268	0.0500689590
O	1.8517108279	-0.6369899118	0.3182392802
O	-0.0554953658	-1.1009249590	-0.5628874582
O	1.0920472105	-2.6484806646	0.3948553326
N	0.7975949411	-7.1424759991	7.1911818870
O	-0.2191529065	-7.0971941411	7.9033555165
O	1.8723112789	-6.6841995538	7.6130650507
O	0.7396264043	-7.6460347079	6.0571241807
N	0.3272258816	-4.1468485209	5.3687323912
O	0.4533037717	-4.2564087626	6.5996340241
O	-0.7927173587	-3.9332118388	4.8756761900
O	1.3210920320	-4.2509250452	4.6308863654
N	-2.6785007637	-3.3876276054	8.0937087211
O	-2.5644546390	-4.4096816603	7.3969900539
O	-3.2088638305	-2.3690891836	7.6200924083
O	-2.2621834865	-3.3841119694	9.2640446431
N	1.4604993228	1.9387589011	8.5393027227
O	1.4196354676	3.1802681583	8.5409821696
O	0.6102202857	1.2884907138	9.1695366114
O	2.3516429326	1.3475173552	7.9073888784
N	4.5904562947	-4.6682332627	4.4024594529
O	4.6945627031	-3.9863110579	3.3694241808
O	5.1110134739	-4.2767487233	5.4601824741
O	3.9657922042	-5.7416408710	4.3777716839
N	3.0585809144	-3.1424371821	10.1305569461
O	3.9994251560	-3.0629602997	9.3233931158
O	2.8763090708	-2.2385784899	10.9629236623
O	2.3000079056	-4.1257735485	10.1053540398
N	0.2874038508	3.5998946487	3.2158653747
O	-0.0438340789	2.8227514756	4.1265507393
O	0.8234495596	3.1576411634	2.1862611965
O	0.0825959069	4.8192922888	3.3347842839
N	-1.6830278311	1.4312932001	6.6180762817
O	-1.0121443832	2.4675722589	6.7561306999
O	-2.4014916335	1.0280753485	7.5477230101
O	-1.6354474383	0.7982314831	5.5503742754
N	-1.3323253558	1.5260501943	11.5340844362
O	-2.3739800194	1.7869185836	10.9096477651
O	-0.5785805649	2.4446820212	11.8960267903
O	-1.0444152514	0.3465490286	11.7965789645
N	5.1690955079	5.9584086289	6.1509497674
O	5.1869689609	7.0050209315	5.4821440119
O	4.6370521186	5.9475944926	7.2733716121
O	5.6832658581	4.9226096286	5.6973333131
N	3.7356809271	2.0935296722	10.7922276241
O	3.9853415809	3.2479593452	10.4075464586
O	4.1973082866	1.1196056605	10.1746597241
O	3.0243923411	1.9130238658	11.7944774966
N	1.3307879330	7.7894132038	3.5222934213
O	0.1022067965	7.8998369996	3.6686248502
O	2.0959926437	8.0768013164	4.4576477576
O	1.7941647320	7.3916009751	2.4406067854
N	-1.5523452585	4.2879046116	0.6509508184
O	-1.0955901192	3.4704942982	-0.1652801742
O	-2.3060750363	3.9027393976	1.5601026709
O	-1.2553703808	5.4904811070	0.5580298837

N	-3.2574548093	4.5989097794	5.7412694667
O	-4.0061810344	3.7195170473	5.2839999907
O	-3.2629694162	4.8481544974	6.9581772572
O	-2.5032133702	5.2290583008	4.9816305406
N	4.0283066966	-0.1274747478	4.3074476202
O	3.7922095064	1.0464747038	3.9771164036
O	3.8651733833	-0.4859805140	5.4855304053
O	4.4275375216	-0.9429190897	3.4596953692
N	0.8755658920	-1.5417427039	7.7775458020
O	2.0005573232	-1.9372972292	7.4297688504
O	0.4946650847	-1.7042693999	8.9486638827
O	0.1314746691	-0.9836610332	6.9542040100
N	2.7817934566	4.1598931793	4.9721490219
O	2.8040124263	3.1675582231	5.7190061454
O	3.2413799096	4.0811212506	3.8208049438
O	2.2999876458	5.2310009266	5.3766363023
N	6.5345586079	0.6280888453	6.2630447489
O	6.9907751195	0.7532179212	5.1144686218
O	6.0958481547	1.6200071645	6.8685603121
O	6.5170525353	-0.4889594491	6.8061057500

n=25 OPLS/AA

N	9.1853996083	4.5349935905	-11.6233759614
H	8.7228773255	3.9609988399	-12.3381061634
H	9.7067496315	3.9270522755	-10.9808526573
H	8.4784220160	5.0623283425	-11.0976990002
H	9.8335490097	5.1895943452	-12.0768467207
N	9.7531267449	0.1162478011	-10.6230094902
H	8.8740506498	0.2243523582	-10.1036110675
H	10.5182490377	0.5527417360	-10.0954721860
H	9.6669814826	0.5702888771	-11.5398852829
H	9.9532249532	-0.8823916614	-10.7530689185
N	2.0258293783	-4.8385803102	-8.2722076054
H	1.7765741632	-5.8223904055	-8.4278534593
H	2.6237216981	-4.7644023411	-7.4407888852
H	1.1713378831	-4.2874624084	-8.1295581509
H	2.5316835260	-4.4800670442	-9.0906300778
N	6.2450460268	-2.4402804333	-8.5196912589
H	6.7276960508	-2.0481166840	-7.7026890277
H	5.6238550044	-3.2001490518	-8.2181019992
H	6.9377032069	-2.8072685005	-9.1828513553
H	5.6909303154	-1.7055871148	-8.9751218575
N	12.2373642114	-2.8596102638	-5.4497114913
H	11.5589656437	-3.1733095525	-4.7457198457
H	12.7864780229	-2.0786801397	-5.0717474073
H	11.7382954266	-2.5508995551	-6.2922455087
H	12.8657170918	-3.6355521133	-5.6891325179
N	8.0304931327	-3.7331391699	-5.2553668829
H	8.5372768836	-4.5902879799	-5.0049627197
H	8.4049696768	-3.3587395304	-6.1350412051
H	8.1467633526	-3.0366576198	-4.5099608120
H	7.0329631112	-3.9468723843	-5.3715025507
N	4.4950696079	-7.5201206804	-5.5926917937
H	4.7915480294	-8.4116926020	-5.1786433041
H	5.1719586620	-7.2338687209	-6.3097104193
H	4.4476164888	-6.8017681512	-4.8606034320
H	3.5691555400	-7.6331541159	-6.0218096161
N	2.8097268627	-2.6892903263	-4.4006186624
H	3.5057309801	-2.2161139420	-4.9887670073
H	1.9859589767	-2.9240011575	-4.9667917641
H	2.5312131185	-2.0674999201	-3.6324765472
H	3.2160050535	-3.5495458245	-4.0144399039
N	7.4587845977	1.0748277856	-6.8437771325
H	7.4784568594	0.3312439457	-6.1360115127
H	7.6767816324	1.9750041310	-6.4006129130
H	6.5246295027	1.1193152556	-7.2675820966
H	8.1552704156	0.8737470861	-7.5709013186
N	7.5969622472	-7.2810050302	-2.8759546965
H	7.7208934093	-7.4470129439	-1.8703113516
H	8.2958776513	-6.6019499439	-3.1994297853
H	6.6548473858	-6.9108852188	-3.0481941727
H	7.7162306632	-8.1641721759	-3.3858824970
N	5.8317916639	-2.0710149341	-1.8993661448
H	4.8947667917	-1.9008648518	-1.5156091436
H	5.9066175187	-1.6347415471	-2.8258116686
H	5.9887191123	-3.0822122673	-1.9835638491
H	6.5370623203	-1.6662409046	-1.2724795440
N	5.0996523732	3.4080158498	-13.1058508495
H	4.2357918277	3.1225232718	-12.6299569081
H	5.7221484402	2.5970200324	-13.2008198773
H	5.5687325401	4.1369297632	-12.5555020258
H	4.8719358433	3.7755900536	-14.0371241235
N	6.4661634742	-9.6929302541	-8.6393312266
H	7.0128434229	-9.0167238994	-9.1853368100
H	5.4996174827	-9.3584191684	-8.5491876446
H	6.8815629073	-9.7947292882	-7.7058882494
H	6.4706306162	-10.6018480019	-9.1169127343
N	6.2047135941	1.7050183913	-3.2255291691
H	6.7244369536	2.3866796618	-2.6603197924
H	5.7783498637	2.1809653110	-4.0292226333
H	5.4690523918	1.2752645150	-2.6525110844
H	6.8470156735	0.9771647412	-3.5600626158
N	5.7491180150	-0.5718924314	-11.9558223904
H	5.9373584825	0.1330357580	-12.6782335420
H	4.9333306895	-1.1323932318	-12.2288875433
H	5.5590086534	-0.1036739505	-11.0620296802
H	6.5667744180	-1.1845376145	-11.8541394998
N	10.3781936790	2.1874495707	-1.8646324512
H	10.8513324206	2.9077266389	-1.3064418263
H	9.6494100104	2.6247443596	-2.4407247333
H	9.9501458228	1.4954481719	-1.2384179345
H	11.0618869229	1.7218798138	-2.4729447672
N	11.1436722228	0.9273316694	-6.1677729016
H	10.5168448662	0.1453028059	-5.9447159150
H	12.0917507870	0.7106995575	-5.8384520101
H	10.8065810607	1.7791440860	-5.7040696780
H	11.1595115667	1.0741794667	-7.1838537860
N	3.9663616316	3.4833587451	-9.4467568844
H	3.1571078690	3.4736665585	-10.0786196122
H	3.6551866569	3.2831020117	-8.4889980101
H	4.4120196114	4.4079099164	-9.4755600220
H	4.6411316009	2.7687564843	-9.7438505085
N	5.9170791595	-5.7802062084	-10.0268975297
H	5.7761606070	-4.9325560647	-10.5889182527
H	5.0086603424	-6.2186562403	-9.8351385462
H	6.3710042694	-5.5313875214	-9.1401750367
H	6.5124912820	-6.4382241816	-10.5433588306
N	9.8890733351	-8.0566433968	-6.2707871438
H	9.7481086379	-8.3673845431	-7.2391904646
H	9.6957155486	-8.8352065153	-5.6299385483
H	9.2523365291	-7.2778606462	-6.0651194716
H	10.8601324875	-7.7461221852	-6.1489010338
N	1.8227227116	-0.8257581271	-10.3322621361
H	1.1590938685	-1.4088800166	-9.8090014761
H	1.3058765896	-0.1923897565	-10.9535102981
H	2.4389776345	-1.4267281952	-10.8919945464
H	2.3869421074	-0.2750351079	-9.6745417140
N	7.8797621531	4.7548451198	-7.7148416226
H	8.6048473076	4.0363731831	-7.8256824657
H	6.9987556538	4.3109315077	-7.4302314706
H	7.7437486104	5.2443412488	-8.6071024477
H	8.1716977467	5.4277338399	-6.9963502144
N	10.3046616799	-2.1981841048	-1.6085883772
H	10.1074518560	-1.6441637409	-0.7669199037
H	10.3612681422	-3.1923895473	-1.3584165721
H	11.1976622916	-1.8962928447	-2.0155860414
H	9.5522642378	-2.0598897469	-2.2934301719
N	2.7932359028	0.7334352313	-5.9729048538
H	2.9296174491	1.7119785143	-6.2523493291
H	2.6243331189	0.6867703647	-4.9612087902
H	3.6335084501	0.1909295690	-6.2049935799
H	1.9854847261	0.3440634303	-6.4730679883
N	9.9815520969	-4.7845140331	-9.0017555270
H	10.0157842569	-5.6268219042	-9.5879139587
H	10.2084240976	-3.9613103955	-9.5719305454
H	10.6630706894	-4.8706646295	-8.2386395417
H	9.0389293771	-4.6792600238	-8.6085386329
N	6.9193977605	1.8865463797	-10.0504225181
O	7.3648775826	0.7691414184	-9.7406293748
O	7.5921701553	2.9068350792	-9.8281474963
O	5.8011446434	1.9836627196	-10.5824911116
N	8.4058196390	0.0791162816	-0.6902616291
O	7.8323052459	1.1117825836	-1.0745785043
O	9.6177799679	0.1059619888	-0.4192461681
O	7.7673731892	-0.9803965806	-0.5769601236
N	8.5198655446	-2.6795828384	-10.7913231098
O	9.7480287115	-2.6047111697	-10.6209488756
O	7.8673017037	-3.5712193952	-10.2237294213
O	7.9442657553	-1.8628172929	-11.5292916265
N	7.2813790859	-9.5129558526	-5.1094598764
O	7.3977452354	-9.9511994585	-6.2659283680
O	8.2940611907	-9.2758089964	-4.4303028739
O	6.1523299226	-9.3118589410	-4.6321480031
N	6.4172542842	5.5634050433	-10.4991697286
O	7.5433224397	5.8736979158	-10.0764325132
O	6.2699613407	5.2289918780	-11.6863893943
O	5.4384782841	5.5875253555	-9.7346866629
N	4.7393390692	3.1367112964	-6.4088803576
O	5.0081472777	2.5954068212	-5.3236386688
O	3.5697884508	3.1421703573	-6.8273770248
O	5.6400822044	3.6725571421	-7.0756259160
N	8.9132865476	3.1079971343	-4.4861919697
O	8.5290757770	3.3102663494	-3.3223676018
O	8.1044642696	3.1701417790	-5.4269164122
O	10.1063205567	2.8435830616	-4.7092920748

N	0.9753343436	-2.0141194111	-7.3801139642
O	0.7029754387	-2.4390411070	-8.5151382328
O	1.1317382421	-2.8105392657	-6.4397570391
O	1.0912894434	-0.7927768773	-7.1854464641
N	9.6883780604	-1.7320545635	-7.6226635787
O	8.8775357805	-2.6616639070	-7.4764473808
O	10.9056548375	-1.9437032551	-7.4943670848
O	9.2819432361	-0.5907956096	-7.8971764914
N	2.5861822558	1.8241237269	-12.0228223849
O	2.8071722835	3.0257780371	-11.7987504384
O	3.4759921850	1.1100529793	-12.5141055703
O	1.4753814046	1.3365397717	-11.7556109309
N	3.3560190333	-0.2392783097	-2.5263002418
O	2.6307298002	0.1102156118	-3.4722534320
O	3.2351076692	-1.3748517485	-2.0375556069
O	4.2022203116	0.5468018404	-2.0690913184
N	10.5122358508	2.5827849612	-8.8681530271
O	9.8241276027	2.9998072598	-7.9217824061
O	11.1304049950	1.5106365120	-8.7614641201
O	10.5821750110	3.2379116393	-9.9212134029
N	12.2901630709	-5.8467141652	-6.2912159985
O	13.2874908695	-5.1771507061	-5.9749250766
O	12.2422364184	-7.0572438996	-6.0167370209
O	11.3407611606	-5.3057474545	-6.8819863736
N	3.4546908181	-7.7396575707	-8.4740048898
O	2.6318759448	-7.4370395422	-7.5939958420
O	3.6559592483	-6.9692868766	-9.4274396928
O	4.0762377615	-8.8126471570	-8.4005790753
N	7.2419945667	-6.2787107623	-7.1166033219
O	7.5844927130	-5.3802914223	-7.9030863924
O	6.2895917176	-7.0236160952	-7.4013185636
O	7.8518997606	-6.4322248929	-6.0454041473
N	3.9518497985	-3.1357176162	-11.0614417815
O	3.5933518522	-2.1728517396	-11.7595724550
O	3.1406978652	-3.6854605003	-10.2980066413
O	5.1215006198	-3.5488409411	-11.1267463008
N	12.4743178803	-0.2775486994	-3.6100230116
O	12.9874843785	-0.4038861586	-4.7341746484
O	12.3582472371	-1.2652395115	-2.8657029350
O	12.0772217057	0.8364804686	-3.2301911457
N	5.5493131858	-1.2805430610	-5.4660674290
O	4.4474520882	-1.4144741628	-6.0237254716
O	6.5964498831	-1.3905514171	-6.1251734975
O	5.6040376300	-1.0366034068	-4.2493023384
N	8.7581949247	-7.8056557365	-9.6996676790
O	9.5868072722	-7.0222626652	-10.1923286013
O	7.6207878855	-7.8962310072	-10.1907066371
O	9.0669898651	-8.4984740948	-8.7159670067
N	4.6057096358	-4.5007153046	-6.5038518998
O	4.7589706321	-4.3685788622	-7.7294411208
O	3.5123888492	-4.8794063927	-6.0518998798
O	5.5457701828	-4.2541604605	-5.7302140761
N	7.8728115169	1.9908321107	-13.1700006120
O	8.8560869318	1.3913192290	-12.7044131616
O	7.9519901365	3.2018509975	-13.4349193029
O	6.8103566269	1.3793256132	-13.3706695330
N	8.8080988384	-0.8341291922	-4.1682077032
O	8.4353562951	-1.7678810690	-3.4386953044
O	9.8264943912	-0.9611632456	-4.8680296507
O	8.1624453091	0.2266575919	-4.1978981784
N	4.3216392577	0.3073828482	-8.6995341847
O	4.6804663002	-0.6115902016	-9.4543517774
O	5.0895710163	0.7199000813	-7.8145888432
O	3.1948795495	0.8138390726	-8.8296620382
N	9.9895993237	-4.8116836733	-3.1678995168
O	10.5804133785	-3.9078002182	-3.7818631664
O	9.3676372743	-5.6848899162	-3.7953483015
O	10.0207473434	-4.8423609104	-1.9264860830
N	5.2292163036	-5.0977360085	-2.9606131047
O	5.2220433458	-6.2777855180	-3.3484914551
O	4.1892989547	-4.4203635248	-3.0131139303
O	6.2763074532	-4.5950585781	-2.5202335741

n=26 OPLS/AA

N	-1.4615603811	3.2497398452	-2.9021542654
H	-2.3148892121	3.3808850808	-3.4579122442
H	-1.7117124120	3.1338428535	-1.9131009129
H	-0.8547436297	4.0712420062	-3.0077154028
H	-0.9648971017	2.4129895680	-3.2298890432
N	4.4086168394	7.4583587003	3.3959961089
H	4.4831387434	8.3221878363	3.9459795698
H	4.1271500741	6.6849706919	4.0099033772
H	5.3181469201	7.2439822912	2.9705160135
H	3.7060316925	7.5822948231	2.6575860109
N	6.8119508495	4.2920388211	-0.9588218005
H	6.3663737307	3.4805895391	-0.5146966025
H	7.3230303591	4.8320363893	-0.2507010152
H	6.0922637989	4.8853434688	-1.3880952197
H	7.4661350752	3.9701850971	-1.6817939323
N	5.5275887249	9.4855522394	-5.5820003754
H	4.6698696348	8.9212385600	-5.5717958954
H	6.1433607437	9.1587127328	-6.3358143068
H	6.0101702088	9.3910837088	-4.6806534210
H	5.2869534768	10.4711734064	-5.7397378684
N	-3.0509136198	5.0702720881	-6.4264629775
H	-3.5753266116	4.3693763242	-5.8898325276
H	-2.3265950862	5.4866672965	-5.8296276756
H	-3.6921705345	5.8043572078	-6.7491766749
H	-2.6095627578	4.6206868412	-7.2372145094
N	8.2082406502	6.3964720746	-4.3783179444
H	8.6277368640	6.5637255016	-5.3004271112
H	7.1881583864	6.4933758090	-4.4437131033
H	8.5746761557	7.0809088758	-3.7063753802
H	8.4423916033	5.4478782749	-4.0627570811
N	1.3480254316	2.1006923967	0.2055669882
H	1.1719647297	1.8327069715	-0.7698419203
H	1.9773565604	1.4157946420	0.6404141813
H	0.4596253429	2.1239490882	0.7198042221
H	1.7831549218	3.0303186240	0.2318905196
N	5.2003689201	9.1348274877	-9.4096655965
H	6.0935725585	8.6304648763	-9.4548509272
H	4.5955487241	8.6973034538	-8.7046833728
H	4.7395982946	9.0977038636	-10.3264795314
H	5.3727569731	10.1138372661	-9.1526485985
N	4.7794906462	1.2555625733	-4.9064460631
H	4.8373129746	1.5760719571	-5.8801844319
H	5.4027118077	1.8243176127	-4.3213411371
H	5.0642645288	0.2705402570	-4.8528699842
H	3.8136733298	1.3513207786	-4.5713896476
N	-3.1825119917	6.0024667784	-0.8279001345
H	-3.1322590982	5.6252438270	0.1257316546
H	-2.2324071799	6.1517221932	-1.1874253119
H	-3.6883534059	6.8959018054	-0.8163312015
H	-3.6770282339	5.3369989205	-1.4335747504
N	3.2167153653	3.9575567552	-2.5767908261
H	3.5862584618	3.9504984566	-3.5347178472
H	2.2074742345	4.1448989526	-2.6006639595
H	3.6880732623	4.6902908285	-2.0335063381
H	3.3850558624	3.0445387764	-2.1382760925
N	5.9773517079	4.7601014789	-7.1301163325
H	5.6310909155	4.0430554760	-7.7783328018
H	6.5910660327	4.3231651901	-6.4324945923
H	5.1828146924	5.2052934170	-6.6560376541
H	6.5044348536	5.4688911341	-7.6536009132
N	3.1610982864	11.4119524119	-0.7240329319
H	2.5735780483	10.6852013591	-0.2987224868
H	3.9118429423	11.6697197111	-0.0727471523
H	2.5877904989	12.2385014132	-0.9298737763
H	3.5711810839	11.0543864563	-1.5947878978
N	1.7921585306	11.8738452063	-9.7233936346
H	1.6831073284	12.6533345360	-10.3827420386
H	2.3868552677	12.1687997795	-8.9400850899
H	2.2283663606	11.0779430467	-10.2034957127
H	0.8703050596	11.5953042222	-9.3672523394
N	-2.0956899098	10.0334026771	-4.5582471255
H	-3.0249051326	10.1169410131	-4.1295088444
H	-2.1717477323	10.1810068563	-5.5714908767
H	-1.7178246787	9.0961962270	-4.3763200492
H	-1.4682830006	10.7394666933	-4.1556683142
N	-0.6484699371	8.2927154457	-8.5262277543
H	-1.3097108088	8.9485731940	-8.0939694736
H	-1.1076080657	7.3847014682	-8.6638412751
H	0.1666465051	8.1746457387	-7.9131370155
H	-0.3432080232	8.6629420206	-9.4339628319
N	-0.3255694205	9.0443947334	-1.2676313499
H	-0.0038829394	8.6517259815	-0.3751263602
H	0.0903575887	8.5161890828	-2.0436714257
H	-0.0397259438	10.0284980159	-1.3314260238
H	-1.3490260741	8.9811654711	-1.3203007209
N	1.4634931071	6.9808574483	-4.7296222085
H	0.6167722360	7.3408512561	-4.2738763118
H	2.2788853988	7.1695524716	-4.1348413377
H	1.5872208250	7.4447262230	-5.6372304587
H	1.3710931441	5.9683001930	-4.8725402820
N	4.8758545191	3.0211175859	2.9957607204
H	4.0117801019	3.4826075267	3.3033944859
H	5.2925210232	2.5136111706	3.7851028188
H	5.5384916882	3.7275194863	2.6549618631
H	4.6606244215	2.3607326094	2.2395840136
N	7.8928666373	8.5789462554	0.6837761915
H	8.8753333506	8.8435126857	0.5459362163
H	7.4515789593	9.2321676805	1.3416528063
H	7.8450274108	7.6261970399	1.0635338311
H	7.3995277855	8.6139078730	-0.2160182221
N	0.5400479585	5.6060063765	2.0280814741

H	0.8644807403	5.6171360954	3.0021736654
H	1.2251048345	5.1113090245	1.4448094175
H	0.4362726930	6.5702441506	1.6908697702
H	-0.3656661177	5.1253362463	1.9744739921
N	2.0751597288	11.8864653347	-5.5529653499
H	2.2701111109	10.8833568381	-5.4529483503
H	2.8800302240	12.3495235088	-5.9911380314
H	1.9069131711	12.2964386879	-4.6267631745
H	1.2435845991	12.0165413272	-6.1410117459
N	-5.7320368830	7.5049456671	-4.0311785276
H	-5.4249544581	7.7589657095	-3.0849173058
H	-5.5104011631	6.5184042733	-4.2096396480
H	-5.2472447853	8.0943235768	-4.7180888752
H	-6.7455468263	7.6480893560	-4.1120673598
N	0.7370418823	2.5039865398	-6.7435876919
H	1.1012668428	3.3112464781	-6.2240613039
H	-0.0696212999	2.7957691428	-7.3078501384
H	0.4462036255	1.7743337366	-6.0823347757
H	1.4703187156	2.1345975879	-7.3601040438
N	2.7389364215	5.6324665770	-9.1188986981
H	2.9456906630	5.9526452510	-8.1654972098
H	2.8454513884	6.4160884569	-9.7737467695
H	1.7753054921	5.2804181941	-9.1602857994
H	3.3892983438	4.8807147179	-9.3760640852
N	3.9671073579	7.8948156792	-0.9600627469
H	3.1851553689	8.1398599393	-1.5787229890
H	4.0039847814	6.8754876547	-0.8423974613
H	3.8320660600	8.3403197176	-0.0448985410
H	4.8472224597	8.2235956441	-1.3742325990
N	5.0383738115	6.3672028762	-3.7466786805
O	5.6420592231	5.8168357540	-2.8109020695
O	3.8466492295	6.6907519781	-3.6120443233
O	5.6264134555	6.5940210791	-4.8170905103
N	7.2564904985	8.5064472308	-2.4043043177
O	6.4354788805	8.6519140085	-1.4835452556
O	8.3484773673	7.9577319862	-2.1818314661
O	6.9855150297	8.9096960223	-3.5475371518
N	5.4754407640	10.3789890601	1.4117057364
O	6.5420874555	10.3103682342	2.0446148364
O	4.8494442122	9.3422862400	1.1353120121
O	5.0347902696	11.4843135959	1.0551900737
N	-0.7308542797	4.7060647446	-8.4800999155
O	-1.3453000942	5.7757556079	-8.6258246869
O	-1.2745196990	3.7515339390	-7.9001716751
O	0.4272578864	4.5909045941	-8.9143037340
N	4.0295984698	2.5472893823	-8.0972522547
O	4.2901008119	3.4971671609	-8.8541463335
O	2.8882926897	2.0570737545	-8.0859533290
O	4.9104026166	2.0876268615	-7.3516565015
N	3.0627549685	4.5663583814	-5.7024849082
O	3.9122534498	3.8976976294	-5.0907144938
O	1.8580043025	4.4420656545	-5.4265409616
O	3.4180074392	5.3593124986	-6.5901999838
N	2.5080765527	8.6359692683	-7.4648888487
O	1.5661895022	7.8777343699	-7.1803778051
O	3.6621763973	8.1856641859	-7.5559755774
O	2.2958635876	9.8445102222	-7.6583133193
N	-1.5416495194	3.6773353108	0.7493148042
O	-1.9265994638	3.2799545054	-0.3628542237
O	-1.9616463412	4.7545232568	1.2035027714
O	-0.7367021050	2.9975276231	1.4072963946
N	1.6230780956	8.4046949247	1.0314809901
O	1.9484650422	9.3989028213	0.3616363033
O	0.4393323552	8.0286389282	1.0500772368
O	2.4814375805	7.7865425271	1.6827299544
N	0.5212174694	5.7718078402	-1.8183390211
O	0.7271788665	4.7611559836	-2.5105677685
O	-0.6177795040	6.0026063147	-1.3796638912
O	1.4542537969	6.5516618500	-1.5647851996
N	2.9723666227	9.5635507550	-3.4526793635
O	3.9046461337	10.2029359376	-2.9378401533
O	2.8731318735	9.4971882979	-4.6891122973
O	2.1393211902	8.9905275681	-2.7310850591
N	7.2933728683	3.4471599870	-3.9884813012
O	7.3143788977	3.6816727254	-5.2081453669
O	8.1273335126	3.9815095741	-3.2388159936
O	6.4384055063	2.6782970427	-3.5184821647
N	7.1043466661	5.7591705238	1.8508079224
O	6.7579612433	4.6253722341	2.2216670440
O	7.8081732630	5.8926740281	0.8360060468
O	6.7469052043	6.7594661145	2.4947511949
N	-1.6346126963	6.7977379772	-4.0573473077
O	-2.8198293891	6.8275498491	-3.6866849892
O	-1.1950960807	5.8003281671	-4.6531801083
O	-0.8889120188	7.7653366943	-3.8321766442
N	-3.9272632922	8.8449511364	-1.9022462491
O	-4.8297987180	8.0351859273	-1.6325649142
O	-4.0838970798	9.6663737698	-2.8208041541
O	-2.8680932262	8.8332937026	-1.2533691568
N	3.4987359336	5.0134333006	0.5133931041
O	4.1335565366	5.3005453563	-0.5149975709
O	3.9675021967	5.2896291011	1.6300810556
O	2.3951481790	4.4501249909	0.4250957564
N	4.4364541956	12.0034243686	-7.5813550119
O	3.4367888628	12.6738361886	-7.8883324129
O	4.7127203692	11.8121532800	-6.3854828269
O	5.1598539372	11.5242832513	-8.4702505115
N	1.1775745332	1.5937225723	-3.5934083737
O	0.1601024162	1.5746409390	-4.3057315400
O	1.0733965301	1.7518948424	-2.3657494685
O	2.2992255564	1.4546318236	-4.1087445275
N	-4.3707011606	4.2957416450	-3.7189215165
O	-5.2031093133	4.9847104432	-4.3316503092
O	-4.2929856393	4.3703935446	-2.4814218994
O	-3.6160079218	3.5321203324	-4.3436928440
N	2.6730881718	4.9471263304	4.2667866519
O	1.7282674345	5.7517251842	4.3211223659
O	2.5014413492	3.8199879929	3.7737202797
O	3.7895566304	5.2696660737	4.7055176634
N	0.6723598692	11.8054892633	-2.6851198285
O	-0.1332928918	11.4250595826	-3.5506917768
O	1.6657751877	12.4753649571	-3.0128551954
O	0.4845971605	11.5160430172	-1.4918115526
N	7.3103163211	7.4339448435	-7.6704550199
O	7.1455650146	8.6398939774	-7.4223389094
O	8.0973907345	6.7606389227	-6.9847503342
O	6.6879927133	6.9013012014	-8.6042765677
N	-0.6184867926	11.2798581234	-7.7075079525
O	-1.3603938682	10.5448086523	-7.0349798288
O	0.0688550571	12.1491474769	-7.1463349733
O	-0.5639215228	11.1456181330	-8.9412100485
N	2.3492567335	8.8150002738	-10.6348000836
O	2.9741021517	7.7439475098	-10.5610973630
O	2.9540434652	9.8710101854	-10.8839867343
O	1.1196235937	8.8300431384	-10.4593160123
N	4.4570124688	1.9183331390	0.0505906151
O	3.9172550315	1.9462164708	-1.0678468394
O	3.8321918675	1.4808992301	1.0310337304
O	5.6215914451	2.3278840459	0.1885850653
N	-3.8903675576	8.0195484739	-6.4143290238
O	-4.3933976624	8.9479926464	-5.7601348301
O	-4.6051041773	7.0896103855	-6.8234556878
O	-2.6725998528	8.0210423909	-6.6593967508

n=27 OPLS/AA

N	3.9282128477	-1.4323247682	1.2625984797
H	3.3131460641	-1.8249987523	1.9849114947
H	4.8844382220	-1.7795277634	1.4015869648
H	3.9231293251	-0.4077124785	1.3287750442
H	3.5921371803	-1.7170604610	0.3351211187
N	4.6795301589	-6.1553686954	9.7236292935
H	5.2945338550	-5.8250120883	10.4765376966
H	4.0380414812	-5.4017328970	9.4502064613
H	4.1379939539	-6.9629064591	10.0535638673
H	5.2475519447	-6.4318230157	8.9142098819
N	8.1349444592	-0.6946197546	7.5824582222
H	8.6757390129	-1.1087539910	6.8141677937
H	7.1412811278	-0.6667182793	7.3253796794
H	8.4692582473	0.2596713514	7.7607790012
H	8.2534999752	-1.2626785028	8.4295056661
N	12.2464697103	-3.9992751585	3.5971560345
H	12.0833818910	-4.9446571679	3.9630676593
H	12.6318841903	-4.0616064911	2.6475218054
H	12.9117698395	-3.5070975422	4.2049162307
H	11.3588427614	-3.4837403536	3.5731187990
N	6.2178118715	2.8391865885	7.7246432902
H	6.5972576926	2.1560866639	7.0585866413
H	6.4456373906	3.7889484170	7.4079688717
H	6.6301984222	2.6792156891	8.6512403807
H	5.1981543500	2.7324949186	7.7807766183
N	7.2581633031	-2.1627691700	3.9199737961
H	8.2122480870	-1.8932643393	4.1870420814
H	6.8047587196	-2.6415135649	4.7070333704
H	7.2945762921	-2.7936518123	3.1107169086
H	6.7210710430	-1.3226467011	3.6751030842
N	8.4343338076	-1.6661901078	12.5985685184
H	8.9462133343	-2.2658307450	11.9408097830
H	9.0870559796	-1.2855234546	13.2937529190
H	7.7116198691	-2.2189711421	13.0743344433
H	7.9924465461	-0.8944356737	12.0853762879
N	13.6928712203	2.2719913013	11.6868266916
H	13.2821772760	3.0495624130	11.1567797617
H	14.3314732150	2.6434241927	12.3998903529
H	14.2126339401	1.6589959926	11.0478282639
H	12.9452000501	1.7359833642	12.1428078718

N	9.0460288184	-6.0689718157	10.9649244457
H	8.7449512621	-5.6658955757	11.8599763701
H	9.0524196168	-5.3370637695	10.2448519112
H	8.3960203807	-6.8144982185	10.6893977904
H	9.9907237210	-6.4584293068	11.0654725828
N	3.1623800300	-0.4246175947	6.8780508653
H	4.0854794887	-0.6781541554	6.5067791328
H	2.4534353260	-0.5470255381	6.1454879754
H	3.1736270829	0.5557295664	7.1830565561
H	2.9369791213	-1.0290204988	7.6768794351
N	8.8700873970	-4.7079067405	1.3855106014
H	9.3806657531	-3.9664375048	1.8792404996
H	8.6868276104	-4.4123616422	0.4194341909
H	7.9791715565	-4.8866893564	1.8635704988
H	9.4336851653	-5.5661377364	1.3797976971
N	11.0130958873	0.3463783027	4.9006604405
H	11.5143697071	1.2366840455	4.7990910571
H	10.0033015593	0.5102006180	4.8128404563
H	11.2154439998	-0.0570465500	5.8229072370
H	11.3192687713	-0.3043240355	4.1678029125
N	11.1621607139	5.3208681018	7.3360514376
H	11.3091143790	5.8108032461	8.2263346916
H	10.2878773291	5.6458463379	6.9068051277
H	11.1044139587	4.3101046587	7.5071014261
H	11.9472373318	5.5167186415	6.7039653722
N	3.3895315865	-3.5102028801	4.9438047865
H	4.0651570927	-2.7395785670	5.0062903135
H	2.6648578357	-3.2750601625	4.2554803407
H	2.9542612119	-3.6590306336	5.8617511492
H	3.8738508639	-4.3671414067	4.6516974034
N	7.3338865021	1.0301857016	1.4094362207
H	8.2523256457	1.4580385019	1.5757119508
H	6.6690399481	1.7527170453	1.1091660053
H	6.9973985498	0.5943596668	2.2760774668
H	7.4167827594	0.3156280090	0.6767896220
N	13.2043800770	-2.8214554620	7.6398518670
H	13.4990150195	-2.8114702735	6.6563242140
H	12.2361392742	-2.4871576396	7.7104875462
H	13.8190986108	-2.2079355544	8.1875287528
H	13.2632676904	-3.7792583709	8.0050659972
N	9.7651529509	3.8021121680	3.8063668142
H	9.6123353082	3.2084215042	4.6300258829
H	9.0411982865	4.5292765222	3.7695384122
H	9.7168057115	3.2276826851	2.9567028585
H	10.6902723487	4.2430673822	3.8692009056
N	10.5237751387	-5.9220282828	7.3020238272
H	10.8619639434	-6.1844498265	6.3687503607
H	9.8376665323	-6.6144272058	7.6246279146
H	10.0812911747	-4.9965530304	7.2579894916
H	11.3141792338	-5.8926833243	7.9567266330
N	12.3725868547	-2.2304802497	11.4065857514
H	11.8582484437	-2.0033339589	12.2657112714
H	11.7898817522	-2.0100813272	10.5904278231
H	12.6050340478	-3.2305672642	11.4011277427
H	13.2371826743	-1.6779382270	11.3690770052
N	10.0118844824	-1.0961016784	-0.9360452003
H	10.2648560515	-1.0225345613	-1.9284308925
H	10.3607604724	-0.2724489015	-0.4319456742
H	10.4316648203	-1.9442920060	-0.5378076329
H	8.9902568317	-1.1451311729	-0.8459975680
N	1.6850339139	2.0952988124	3.5594795570
H	2.4884477141	2.4477441284	3.0260522775
H	1.4775572580	1.1324152038	3.2695773682
H	0.8676635306	2.6903167455	3.3803292291
H	1.9064679352	2.1107195152	4.5619588337
N	7.1689162072	-5.9671086733	5.6693142972
H	7.9090065691	-6.5065659966	5.2051072850
H	6.3786305404	-5.8456073901	5.0251709216
H	7.5346769557	-5.0466087125	5.9397480620
H	6.8533514846	-6.4696531194	6.5072304683
N	5.3037475143	-2.1240624678	10.0763648278
H	4.2822333145	-2.1350564907	9.9732921892
H	5.5562327405	-2.4466922998	11.0178508274
H	5.6514826230	-1.1680153054	9.9374776738
H	5.7250403841	-2.7464857861	9.3768385204
N	13.3989909750	1.5936564187	7.4525928020
H	13.5913486859	2.4841453816	6.9790275843
H	12.4327492241	1.5924398380	7.7998824278
H	14.0462920192	1.4786215163	8.2412650112
H	13.5255741582	0.8194198060	6.7901957236
N	13.5143676823	0.0078020283	1.5594570712
H	13.8597649514	-0.3907791175	2.4404053638
H	12.7552305568	0.6700655311	1.7578318758
H	13.1624586182	-0.7459007715	0.9575205096
H	14.2800169392	0.4978220831	1.0820713934
N	9.7055749521	2.0838138729	10.8402949557
H	8.7588764555	2.2125627363	10.4642340773
H	10.2658212725	1.5359526063	10.1768141509
H	9.6529742610	1.5858774968	11.7366922208
H	10.1446268973	3.0008627778	10.9834390078
N	5.3989635550	3.8615467832	4.0568110084
H	6.0422766238	3.0615389805	4.0760959443
H	4.8392622547	3.8299962095	3.1965937677
H	5.9381205158	4.7349802414	4.0824876502
H	4.7761954523	3.8196709224	4.8720666901
N	2.8379179856	-3.2898295420	8.5126243117
O	3.0921953076	-4.1996693630	9.3191926315
O	2.5127760922	-3.5667846981	7.3461782128
O	2.9087826141	-2.1030336094	8.8725023805
N	10.1752059352	-1.7225991253	2.5956048312
O	9.8734046866	-2.8818322019	2.9244096235
O	11.2937613306	-1.2662611039	2.8847541845
O	9.3584511309	-1.0197035042	1.9776501881
N	6.8599043583	0.8512090513	10.2296363890
O	7.1654677530	2.0460608302	10.0813860964
O	6.0683671705	0.3099619344	9.4399919439
O	7.3458785428	0.1976038630	11.1675318818
N	9.7701977969	-3.0987785137	5.9457106146
O	10.9766866123	-3.3281238877	6.1322671714
O	9.3908976769	-1.9460987868	5.6802322219
O	8.9430084357	-4.0221136099	6.0246325142
N	3.2049210127	2.6664550617	6.5611428219
O	3.6924527606	2.1003087593	7.5535182901
O	3.7908679870	3.6340326099	6.0478419916
O	2.1314414262	2.2650234926	6.0820677982
N	12.3259914890	-5.3866577987	9.8915873231
O	12.6786917374	-5.1437274424	8.7255661880
O	12.7759825667	-4.7091931053	10.8305055997
O	11.5232995166	-6.3070535894	10.1186903645
N	10.3482729556	-6.4715558732	4.0851676511
O	9.3144896714	-6.9313863180	4.5978722388
O	10.4036580899	-6.2864109543	2.8581094794
O	11.3266718933	-6.1968701261	4.7995218103
N	11.2845405915	-0.4529115839	8.6982182763
O	11.2906100732	-1.6280535330	9.1007384270
O	11.2284280884	-0.2137574030	7.4805670225
O	11.3345836533	0.4830769377	9.5133500354
N	7.2475949203	-1.9672274222	0.1231542913
O	6.4936875370	-2.0624713710	1.1057885867
O	7.8113167679	-2.9782485222	-0.3274807326
O	7.4377806092	-0.8609614828	-0.4088454084
N	11.8953420522	4.6921962444	10.3827216110
O	11.5477388459	5.7560343068	9.8437981482
O	11.1614557398	4.1561774380	11.2295466404
O	12.9768324417	4.1643765634	10.0748197965
N	7.3532007227	-7.2977547528	8.5943767065
O	7.2287429735	-7.5392421683	9.8064872658
O	8.4819604722	-7.2870204209	8.0759134841
O	6.3488979140	-7.0670014834	7.9007288114
N	4.8645721443	-0.1197208920	4.0584372373
O	5.7315223111	-0.1280861875	3.1688613040
O	4.2458917382	0.9269341263	4.3129283742
O	4.6163021838	-1.1580114506	4.6935225450
N	1.6616385711	-1.1757839264	3.5489816718
O	1.6529971755	-1.0231432594	4.7817201128
O	2.1249584349	-2.2172576221	3.0553369047
O	1.2069597368	-0.2869501823	2.8098874028
N	12.1621352912	-2.9442969901	0.4061332858
O	12.6078970579	-1.8536305783	0.0127363661
O	11.0145421714	-3.2974583273	0.0878173856
O	12.8639672092	-3.6818026585	1.1178466786
N	7.5773945206	1.2518207712	4.8333647801
O	6.9327757096	1.1639023284	5.8915494742
O	7.3743794779	2.2020976497	4.0595581780
O	8.4250290568	0.3894616411	4.5489864592
N	13.9086120946	-1.2985292192	4.9103175350
O	13.6351694857	-0.4878086745	5.8108613978
O	13.7936519528	-2.5180600244	5.1165860299
O	14.2970151581	-0.8897186297	3.8035042862
N	5.8715009527	-2.9648712370	7.0686900181
O	5.6666364521	-1.7539404522	7.2549561762
O	6.1022036323	-3.3867870162	5.9233598213
O	5.8456627529	-3.7538868779	8.0277548290
N	4.2891847498	2.3375143801	1.4891169691
O	3.6177141570	3.1156071162	2.1867679191
O	5.4053512726	2.6854698115	1.0694537446
O	3.8444884618	1.2114653061	1.2111290198
N	8.9938996294	-3.2292308395	9.8569513571
O	9.6750113684	-3.1855151557	10.8948307571
O	8.2148083316	-2.3005265467	9.5857632356
O	9.0918792669	-4.2016515989	9.0902594615
N	14.4943985466	-0.2069969505	9.9394891963
O	14.7194360526	0.9750028357	9.6308590984
O	14.4386296896	-1.0794996078	9.0570795077
O	14.3251297613	-0.5164943284	11.1305299415
N	12.6109389152	3.7080333919	5.2422935473

O	13.3821435978	3.8971379046	6.1975425890
O	12.0725496681	4.6747975961	4.6779160999
O	12.3781232923	2.5521637444	4.8514216382
N	10.6614542419	1.6656346600	1.3591110806
O	10.7287455216	1.0630434654	0.2749645744
O	9.6151029624	2.2537711604	1.6788959578
O	11.6405150298	1.6800893658	2.1234733248
N	7.8231274090	5.3360941362	5.6364744168
O	7.6344019035	5.3333646991	4.4087150166
O	6.9173946308	4.9725092675	6.4048962336
O	8.9175865739	5.7024087370	6.0958123700
N	6.7206150359	-4.2649359973	12.2124795491
O	7.8062466868	-4.4998910836	12.7685532285
O	6.1631825471	-5.1482384870	11.5401227666
O	6.1924154487	-3.1466775210	12.3287627458
N	10.9140344562	0.1271925364	13.0638814779
O	9.8018115106	0.6798551232	13.0405985375
O	11.0008409467	-1.0828506842	13.3308607812
O	11.9394517367	0.7845736993	12.8201849189
N	5.5722615633	-4.6774126611	3.1740876129
O	5.0978044752	-5.4015559465	4.0648877503
O	6.7752511853	-4.7653331520	2.8772646564
O	4.8437284428	-3.8653482312	2.5801099539
N	9.8591269433	2.3904970017	7.3395280421
O	9.7026333941	2.6245387485	6.1296717941
O	8.9994729522	1.7469537047	7.9639197071
O	10.8752753017	2.7999988815	7.9249930963

n=28 OPLS/AA

N	3.4162137910	-5.9503234993	3.7566189567
H	3.4614391682	-5.0671741723	3.2348546143
H	4.2837295810	-6.4795102241	3.6096072229
H	2.6174476421	-6.5034770159	3.4246108987
H	3.3022388166	-5.7511317249	4.7574025828
N	-0.1052333787	-4.8643238225	2.0296003770
H	-0.7334837938	-5.6617962475	1.8760580544
H	-0.2384280199	-4.5019140046	2.9809956666
H	-0.3175159561	-4.1253980864	1.3490414979
H	0.8684936430	-5.1681877281	1.9123061398
N	1.2018240018	-9.7630515696	-0.3225361667
H	1.3066801906	-9.3585933504	-1.2604353223
H	1.8037623858	-9.2570338209	0.3376538857
H	1.4739439320	-10.7528564277	-0.3442757761
H	0.2229096008	-9.6837222856	-0.0230883676
N	1.1939335020	-5.5924365912	-1.6237633161
H	1.3406431680	-6.0910831451	-2.5092371925
H	0.5854058251	-6.1505105081	-1.0134500726
H	0.7513517492	-4.6851151835	-1.8111793749
H	2.0983334088	-5.4430380139	-1.1611874868
N	2.4546060973	0.2900930061	1.6476568193
H	2.4314433138	0.1558054381	2.6653337975
H	2.0443604073	-0.5295123460	1.1848607239
H	3.4272495516	0.4058574017	1.3397626171
H	1.9153710942	1.1282214000	1.4006711297
N	9.7379862190	-5.4038049154	7.3910789531
H	9.1927400229	-5.4603238892	6.5228921708
H	9.2849933724	-4.7472621420	8.0375955833
H	10.6870561103	-5.0747738470	7.1783809947
H	9.7871548392	-6.3328598385	7.8254462179
N	11.9248613823	-5.2035161324	3.8276965036
H	10.9213534186	-5.1046384462	4.0211676059
H	12.1218170906	-4.8868102328	2.8710667385
H	12.1966737729	-6.1888725256	3.9247857758
H	12.4596002700	-4.6337432287	4.4937660828
N	5.1564451121	-10.1771398211	-0.0082312005
H	5.1492083268	-9.5165523934	0.7777764183
H	4.7863983567	-11.0830452924	0.3026310863
H	6.1188728243	-10.2979099805	-0.3449551105
H	4.5713009335	-9.8110509748	-0.7683764307
N	1.4020999471	-11.8300361954	4.1981324299
H	1.5288417037	-12.4323266870	3.3762937665
H	0.4449225199	-11.9345213268	4.5546838505
H	1.5637605331	-10.8512643122	3.9333552035
H	2.0708751554	-12.1020330424	4.9281960984
N	9.1489845599	-1.4359014554	0.5583099475
H	8.3522635511	-2.0653142585	0.7109901853
H	9.9649527269	-1.9829059226	0.2596182189
H	9.3683731248	-0.9433339820	1.4320842123
H	8.9103480608	-0.7520522714	-0.1694526780
N	-1.0419802754	-8.5149715894	3.8101067796
H	-0.1128650490	-8.7632560927	3.4504739123
H	-1.3778542292	-9.2588414178	4.4330682961
H	-1.6947002113	-8.4052068464	3.0251584307
H	-0.9825007070	-7.6325822427	4.3317261291
N	5.7125420605	-6.0620394044	9.3180007053
H	6.0540202077	-5.1044319807	9.1744152088
H	5.5507412038	-6.2211636335	10.3193670958
H	6.4122348399	-6.7301001929	8.9739653564
H	4.8331723233	-6.1924608776	8.8042550206
N	6.5103963292	-0.2192447484	2.9998271171
H	5.6799877218	0.2793707793	3.3404738517
H	6.6779320047	0.0264076211	2.0170649912
H	6.3579412649	-1.2315807928	3.0783682578
H	7.3257235165	0.0488238842	3.5634016994
N	1.7339145872	-2.6072142841	5.4941230825
H	2.3548787686	-2.8825848184	4.7241811029
H	2.1431541563	-2.9055533016	6.3872924344
H	0.8176183376	-3.0539267645	5.3713034306
H	1.6200076912	-1.5867925202	5.4937146121
N	6.7291322339	-4.8373727476	2.7478042361
H	6.3578450372	-4.7433425162	3.7004532957
H	5.9516226996	-4.9539059092	2.0874000933
H	7.3447899228	-5.6576779571	2.6998407350
H	7.2622709144	-3.9945645164	2.5035237481
N	6.0096205150	-8.1200342176	5.8703227397
H	6.6300917020	-7.3454196746	6.1334271171
H	6.5463495837	-8.9949673490	5.8448549577
H	5.6085435389	-7.9374370943	4.9429441364
H	5.2534978397	-8.2023119982	6.5600650037
N	10.5088278906	-9.3801567584	6.0488382079
H	11.4241140534	-9.2869856196	5.5929802512
H	10.5843141509	-9.0894733994	7.0306934390
H	10.2023683767	-10.3591030411	6.0043155625
H	9.8245158728	-8.7850648827	5.5673631350
N	6.4856219170	-11.3940768300	8.2429533670
H	7.2582504085	-10.7340619626	8.0957742697
H	5.6812937902	-10.8959724782	8.6419308157
H	6.7854141702	-12.1332922568	8.8894212176
H	6.2175300518	-11.8129799796	7.3446870217
N	12.6473521273	-1.1710595687	3.6908365344
H	13.4982245110	-0.6031158663	3.6031311456
H	11.8851266156	-0.5908011831	4.0603594170
H	12.8229684321	-1.9539707367	4.3314959919
H	12.3830897791	-1.5363499355	2.7683594976
N	10.1667094741	-6.0127848191	0.6510553200
H	10.7963258184	-6.5949618235	1.2157581553
H	9.6034654326	-5.4179174944	1.2700251910
H	9.5462483974	-6.6161100898	0.0985499407
H	10.7207988611	-5.4221504357	0.0198885430
N	9.3133509105	-1.6736411355	6.2907165278
H	9.1566485765	-0.7782697102	5.8132330340
H	10.3023524626	-1.7519587670	6.5552481213
H	9.0654356363	-2.4455104923	5.6606441217
H	8.7289668139	-1.7188247005	7.1337403692
N	8.8046901928	-9.4987494287	2.2609200487
H	8.3010563962	-10.1869523779	2.8327292802
H	8.4910454912	-8.5529241926	2.5084605692
H	8.6134726286	-9.6736769509	1.2674052128
H	9.8131857648	-9.5814448637	2.4350856896
N	4.3052392165	-2.3862502226	-0.6449572977
H	4.6572626856	-1.4970110406	-1.0185469818
H	3.3686961522	-2.5697943700	-1.0236832147
H	4.2556062574	-2.3315370887	0.3791416069
H	4.9393921135	-3.1466575249	-0.9167409651
N	1.2687320862	-7.3965650444	7.1558603615
H	2.0044030050	-7.5548753470	6.4573174172
H	0.8978960793	-6.4446910528	7.0526554893
H	1.6609299304	-7.5108150772	8.0978599927
H	0.5117000468	-8.0758788547	7.0156078664
N	1.8298219493	-11.3129573411	8.6726355172
H	1.0348275415	-11.3080957000	8.0228691334
H	2.1681939420	-10.3528634491	8.8066459591
H	2.5848605435	-11.8910198140	8.2853423171
H	1.5314049961	-11.6998503965	9.5756840263
N	5.2771700456	-11.8916614070	3.7459198856
H	4.9307340440	-12.2595779850	2.8521326704
H	5.1427610454	-12.5957789574	4.4810303407
H	4.7586594510	-11.0395160690	3.9892932826
H	6.2765253046	-11.6717729748	3.6612223781
N	5.3158680018	-2.2811046906	6.5655284331
H	4.6524697187	-2.8855201634	7.0643477910
H	6.1126575899	-2.0692173189	7.1774637069
H	4.8480613446	-1.4071999593	6.2977916663
H	5.6502827077	-2.7624819094	5.7225110538
N	5.6502354660	-6.6664346558	-1.2509553262
H	4.7846107121	-6.7585138793	-1.7954287412
H	5.9991616967	-5.7034826982	-1.3231266385
H	6.3564144262	-7.3148049512	-1.6186002095
H	5.4607541858	-6.8889371841	-0.2666662460
N	3.0723394328	-12.3588614243	1.1609110175
O	2.5872172508	-11.9130989394	0.1078344835
O	2.3323689993	-12.8683066582	2.0187730239
O	4.2974330347	-12.2951786240	1.3561257023
N	11.7465036368	-3.2096254483	0.9500310928
O	12.3050298725	-4.1925824838	1.4646783712

O	10.9694314846	-3.3669468793	-0.0062255404
O	11.9650497292	-2.0693460638	1.3916408031
N	-0.2590265126	-5.1379478664	5.3208017746
O	-0.2018110738	-4.1515652336	4.5679448120
O	0.1427059813	-5.0488472965	6.4928469968
O	-0.7179748147	-6.2134319349	4.9016131777
N	4.2697252234	-2.5826519098	3.0873402189
O	3.3012356938	-3.2230378422	3.5288736825
O	5.3023019534	-2.4698210128	3.7685688122
O	4.2056379715	-2.0550964495	1.9645772582
N	3.3844180488	-0.0025584938	4.6554141718
O	4.2085206810	0.6037163732	3.9509260256
O	2.2260168063	-0.1793943128	4.2432812254
O	3.7187169281	-0.4319978873	5.7720361634
N	5.7515736730	0.2470278229	0.0183628150
O	6.9245104385	0.2225430934	0.4265756921
O	5.4868487698	-0.1342706445	-1.1338307951
O	4.8433610794	0.6528113466	0.7623441471
N	-0.5721646356	-10.4133100020	6.3325683925
O	-0.2400902146	-11.2558231304	7.1828131328
O	-0.9174465265	-10.7796888057	5.1969782821
O	-0.5589571552	-9.2044170966	6.6179139925
N	7.9981449996	-11.0871094382	5.0876593539
O	7.1271443132	-10.4664007247	5.7194001300
O	7.8405279204	-11.3197466058	3.8776780666
O	9.0267635933	-11.4751812964	5.6659003305
N	3.1092970794	-9.3532954687	5.4100464665
O	2.3389821910	-9.9837142282	6.1531514869
O	3.8403984221	-9.9632343111	4.6122474028
O	3.1485106565	-8.1129368683	5.4647405538
N	12.2951448972	-3.2546567910	6.1588071312
O	11.8421691480	-2.1954712576	6.6235187485
O	11.9493846010	-4.3442153778	6.6449447808
O	13.0938815857	-3.2242837132	5.2079570988
N	7.9976799240	-8.8696071227	-0.7184878425
O	7.3038518768	-8.5769452574	-1.7063995716
O	8.8625093472	-8.0769472178	-0.3101029080
O	7.8266784103	-9.9549297666	-0.1389605815
N	6.8770878210	-4.8887419345	5.9149289637
O	7.6517530256	-3.9178048155	5.9010421855
O	7.1339521361	-5.8781918502	6.6206395976
O	5.8455574708	-4.8702291229	5.2231045509
N	9.3004852219	-3.2217141000	3.2337924881
O	8.2617103985	-2.5410984682	3.2068269718
O	9.4441264573	-4.1674289514	2.4413225984
O	10.1956195304	-2.9566146668	4.0532285538
N	1.0703067865	-2.6298287508	-0.3119893309
O	0.0737727838	-3.3381333616	-0.0923476437
O	1.3512104202	-1.6968711651	0.4585307551
O	1.7859377316	-2.8544819065	-1.3021519011
N	2.9403721973	-8.0981172990	-2.2726993365
O	3.4654143197	-7.0459255867	-2.6730008244
O	1.7202347730	-8.2785764285	-2.4200664954
O	3.6354680586	-8.9698505836	-1.7250302489
N	8.5841061913	-8.4263029519	8.4040587920
O	9.6860230877	-7.9100906012	8.1544152993
O	7.6423366368	-7.7193480459	8.7994102533
O	8.4239587206	-9.6494711933	8.2583507061
N	6.9989049477	-4.0604807021	-0.2115762850
O	7.0923790536	-3.0411502358	0.4921608129
O	6.2405381652	-4.0686200725	-1.1953605524
O	7.6637981595	-5.0716726121	0.0684711101
N	8.8929215387	-6.9484509610	4.1845434415
O	8.7146803911	-7.9885351983	4.8398851718
O	9.4865977536	-5.9853189752	4.6973233559
O	8.4774861370	-6.8714986476	3.0164208563
N	-0.9582381637	-7.6446829208	0.7892050115
O	-0.2874654318	-7.0753347977	-0.0876784288
O	-0.9924020052	-8.8854647139	0.8372808332
O	-1.5948475667	-6.9732487102	1.6180132971
N	3.9074792861	-9.0678733831	8.7510582342
O	4.5685520381	-8.2701215057	8.0657899148
O	4.4409144267	-10.1005184860	9.1893605878
O	2.7129704319	-8.8329799684	8.9980243989
N	9.6106211113	0.1810195949	3.9022176692
O	9.5826944839	0.0578575722	2.6664712832
O	10.6999760747	0.2546413994	4.4945887561
O	8.5491919209	0.2305598530	4.5455934863
N	11.6175517866	-8.2778493483	3.2966175225
O	12.1395016868	-7.8305993222	4.3312931367
O	11.5062203764	-7.5531827797	2.2938804720
O	11.2069329660	-9.4497668864	3.2646789331
N	7.5053338654	-3.0460390236	8.6402218496
O	6.4654223901	-3.6314417973	8.9850488020
O	7.4394763061	-1.9297354066	8.0993457754
O	8.6111037901	-3.5769402941	8.8362711293
N	3.1907168110	-4.8108683786	7.3013882695
O	2.9449870844	-5.2603170352	6.1697387135
O	3.2607420014	-3.5836690271	7.4805422120
O	3.3664214887	-5.5886196997	8.2538846497
N	4.1238840427	-12.7961744148	6.5985882492
O	3.7884699579	-12.7324011256	7.7929283474
O	5.3272543102	-12.7949827843	6.2905030495
O	3.2559271612	-12.8611393868	5.7123326372
N	5.6971249450	-8.1474076197	2.3759566031
O	5.5286766772	-7.4045878298	3.3572118460
O	5.5913205442	-7.6905446310	1.2256959591
O	5.9713778345	-9.3470913640	2.5449621403
N	1.7775082450	-8.4829176672	2.4522691282
O	1.2807471333	-9.5051651875	2.9535238701
O	1.4455663655	-7.3615499808	2.8710375743
O	2.6062119032	-8.5820379131	1.5322451997
N	3.4589816776	-5.0733580745	0.7343854448
O	4.5285115370	-4.9906349544	1.3607085852
O	2.3801399178	-5.1064921995	1.3492191603
O	3.4682935857	-5.1229471096	-0.5067724104

n=29 OPLS/AA

N	0.1589331261	7.6634725193	3.2927311754
H	0.1907062211	6.7261834905	3.7107214199
H	0.7865313356	8.2902978526	3.8098821925
H	-0.8005830520	8.0261081955	3.3381139308
H	0.4590780307	7.6112996259	2.3122075655
N	5.9626872399	2.0326084390	7.4020651689
H	6.5339189733	2.4926133105	6.6835069441
H	4.9872834028	2.3406146719	7.3128788448
H	6.3168436835	2.2827383594	8.3327873404
H	6.0127034565	1.0144678620	7.2790868466
N	-4.4564795703	3.7590273632	3.3462777991
H	-5.4466664130	3.6102051520	3.1190817086
H	-4.2363232666	3.2967980995	4.2362834143
H	-4.2717594539	4.7656777474	3.4285517734
H	-3.8711701119	3.3634283092	2.6011940786
N	4.4064875512	4.1847703877	4.4636953246
H	4.7283064268	3.5093842849	3.7604725142
H	3.5562208235	4.6511442315	4.1263874311
H	5.1394852238	4.8851594818	4.6261916410
H	4.2019380441	3.6933928948	5.3417290271
N	2.3097208778	0.1596695530	9.8863774211
H	2.8093010522	-0.7372635115	9.8727846580
H	2.1765531963	0.4625526234	10.8583634508
H	1.3946661779	0.0486317806	9.4340686172
H	2.8583635711	0.8647164460	9.3802929452
N	5.3192529660	3.3633466403	14.0666007718
H	5.4471894409	3.5373045488	15.0703970669
H	6.1910881650	2.9940679207	13.6693964181
H	5.0737395508	4.2420143559	13.5955363040
H	4.5649948320	2.6799999054	13.9310742757
N	3.9333238979	-1.9663733538	2.6017859608
H	3.8362104904	-1.6927867401	1.6169227985
H	4.7714567762	-2.5487266215	2.7141809576
H	3.1049963947	-2.4990103040	2.8923147043
H	4.0206318357	-1.1249694830	3.1837244235
N	3.1177675968	7.9801948448	9.6547236038
H	2.4347388114	7.3260799320	9.2549218318
H	2.6267253193	8.7980316166	10.0345330058
H	3.7710423553	8.2827300831	8.9226443433
H	3.6385632359	7.5139371104	10.4067948448
N	10.2893375777	2.6041363271	5.7756931716
H	10.5593175792	1.9880196810	4.9999695257
H	9.6956984892	2.0857658139	6.4338111922
H	9.7700016043	3.4099265578	5.4079598782
H	11.1323329009	2.9328326559	6.2610313346
N	4.2270922262	8.3214421243	5.4993489733
H	4.1635500408	7.3625985630	5.8610360110
H	4.8097927019	8.3304468304	4.6540006686
H	4.6484342326	8.9288172092	6.2119512073
H	3.2865918675	8.6639049606	5.2704083586
N	-2.4174793583	6.5302534931	6.4613812847
H	-2.1494591152	6.9378427775	5.5579034365
H	-1.5724664326	6.3047829419	6.9992947270
H	-2.9608884307	5.6736558263	6.3026912054
H	-2.9871031936	7.2047328239	6.9856348900
N	-1.0937454316	2.6240055213	8.2304478625
H	-0.7441142375	1.6776089711	8.4210461191
H	-0.7851792574	3.2593920653	8.9756361215
H	-2.1198306458	2.6093608133	8.1962398230
H	-0.7258572454	2.9496593139	7.3288695720
N	5.2471913507	-0.7834352149	12.2127622448
H	4.3023711545	-0.8520586688	12.6087947133
H	5.9229266161	-0.6136980431	12.9669544147
H	5.2813818536	-0.0072466489	11.5414963931
H	5.4820848583	-1.6607375655	11.7338038439
N	9.0944730583	5.2752671037	1.9783702878
H	9.3058113369	4.3495306802	2.3689906969

H	8.5317855900	5.1669122380	1.1263862964
H	9.9710911629	5.7587930737	1.7503815619
H	8.5692043492	5.8258315212	2.6677229766
N	6.7780192852	5.4691529328	10.3349674782
H	6.6919426569	5.6108072452	11.3482596214
H	7.7073624280	5.0890882274	10.1202424722
H	6.6555593762	6.3670471306	9.8522407395
H	6.0572125960	4.8096692659	10.0191280665
N	7.6463525757	-0.7899283068	4.0047258049
H	6.8146504342	-0.1890565050	3.9665063957
H	8.0495340038	-0.7566717970	4.9484277843
H	8.3399606518	-0.4579791390	3.3243192273
H	7.3812644029	-1.7560052010	3.7796497750
N	7.2508220392	-2.8293156204	7.8551119108
H	7.6628009791	-1.9003993585	8.0021630212
H	7.1752018362	-3.0146594521	6.8480548447
H	6.3166029236	-2.8605426804	8.2799589877
H	7.8486828193	-3.5416600858	8.2902709327
N	1.5402027036	5.2946446604	6.4444782334
H	1.4318564019	4.2937791521	6.6463826928
H	0.7977466319	5.8218290823	6.9188939291
H	1.4753310814	5.4484010139	5.4313710424
H	2.4558765938	5.6145684185	6.7812654659
N	6.8470989367	2.1482035648	1.0185718490
H	7.1705002738	2.9285814357	0.4349126579
H	6.4079332191	2.5169079136	1.8702908526
H	7.6471039469	1.5575326366	1.2741796783
H	6.1628586221	1.5897930333	0.4949036389
N	2.8036721076	-3.0139218371	6.6209521762
H	3.2710966079	-2.6924563591	5.7651423956
H	2.0945133998	-2.3267938348	6.9023481976
H	3.4968321097	-3.1172621819	7.3713424137
H	2.3522467682	-3.9191746597	6.4449748642
N	8.9798685730	1.5364446888	10.1443192951
H	8.1143577781	2.0830186841	10.0644191743
H	8.9134394384	0.7015021733	9.5504398029
H	9.1157847923	1.2515624308	11.1213578841
H	9.7758914402	2.1096959996	9.8410602413
N	3.1414883003	3.8615188546	0.3062742395
H	2.4594465117	4.3964445757	-0.2440979703
H	4.0862150152	4.2183921650	0.1208893429
H	3.0921681385	2.8704173518	0.0426061585
H	2.9281228713	3.9608218469	1.3056988907
N	-1.7867603734	1.0432973498	4.4394546002
H	-2.6933462013	1.2593198182	4.8703519517
H	-1.9382305874	0.5381276411	3.5584920442
H	-1.2346486235	0.4591796440	5.0783713752
H	-1.2808169645	1.9165625065	4.2506034493
N	2.0229091146	0.6340528868	5.2867470462
H	2.5807269041	0.5457366306	4.4292648888
H	1.1754644756	0.0602168405	5.2043792055
H	1.7588328750	1.6167949477	5.4235582502
H	2.5766127469	0.3134630425	6.0897850050
N	-0.7417954724	4.5959732158	1.1791070069
H	-0.0713104272	4.3083828459	1.9015872356
H	-0.2432977698	5.0783310467	0.4220960103
H	-1.4365977257	5.2304715708	1.5900780407
H	-1.2159753141	3.7667071199	0.8026674446
N	0.7130336262	0.3813419322	1.1097351960
H	-0.1487780938	0.5952211878	0.5942040904
H	0.7517560506	0.9511000418	1.9630293750
H	1.5262564185	0.5929448625	0.5197111167
H	0.7228992903	-0.6138981549	1.3619956998
N	2.5204115433	4.1869431358	10.9239853980
H	3.2164397605	4.2400002403	10.1710141089
H	2.4302922374	3.2138047072	11.2388104206
H	1.6135085198	4.5193945034	10.5757921783
H	2.8214063334	4.7745731440	11.7103241506
N	4.7170671469	7.8560853916	0.7728492344
H	3.9234317736	7.5318760681	0.2078117963
H	5.5235787289	7.2405280620	0.6151694087
H	4.4603825674	7.8360480871	1.7668044936
H	4.9608747446	8.8158890334	0.5016106888
N	7.9346651686	6.1389916931	6.5524763583
H	7.7997072415	7.1309066548	6.7807900990
H	8.8068862396	5.8051825429	6.9791337617
H	7.9872727675	6.0264717746	5.5332574257
H	7.1447942942	5.5934067661	6.9167243690
N	4.5428778463	1.2348939473	3.0014738246
O	5.6060109750	0.6622310273	3.2927177804
O	3.4594102976	0.6453368981	3.1482337226
O	4.5632122827	2.3971148520	2.5634696183
N	0.2262870803	5.4081656531	9.1161685877
O	1.2001237943	6.1482846274	9.3326954202
O	0.0334292787	4.4104890018	9.8306369767
O	-0.5546924609	5.6657235373	8.1851726167
N	1.5052575362	6.5753094488	-0.0091952097
O	1.1652189011	5.4632484055	-0.4459009157

O	2.5572135665	7.1033185893	-0.4061999560
O	0.7933395677	7.1593618217	0.8245159137
N	9.6170160477	1.5398762545	2.7020771162
O	10.2902829123	1.1223220206	3.6588310562
O	8.8036269620	0.7879522695	2.1399148381
O	9.7571383815	2.7093554150	2.3074851368
N	6.5761653465	7.0483190470	3.8544959952
O	7.7774920297	6.8786312349	3.5879661513
O	5.9862598745	6.2483747363	4.5995587743
O	5.9647436431	8.0179519505	3.3759626748
N	3.0627028520	6.5839832253	3.1208712664
O	3.1033342450	7.8058138563	2.9006511267
O	3.5806921864	5.7905489260	2.3176484000
O	2.5040816748	6.1555865486	4.1443150965
N	4.6486171122	-2.5714942497	9.5823211066
O	5.6820987565	-2.5526561008	10.2712178522
O	4.6570118068	-3.1134404123	8.4646262480
O	3.6067399346	-2.0483858149	10.0111195648
N	8.6614185547	-0.0020637293	7.3687317103
O	8.7679952186	-0.7085079822	6.3525644784
O	8.5000065745	-0.5329334214	8.4801008900
O	8.7162539151	1.2352512117	7.2735296859
N	7.6890552310	1.1926649090	13.1476239119
O	8.6415529415	0.6690991336	12.5462547230
O	7.5331681003	2.4244884975	13.1111854688
O	6.8924440100	0.4844065256	13.7854320574
N	0.9914945718	-1.7499660461	3.4480135763
O	1.7603711681	-2.3937459962	4.1810852416
O	0.2197311630	-0.9111190225	3.9417086469
O	0.9943813865	-1.9450332767	2.2212458528
N	2.7937080252	1.1387549980	12.7956836310
O	2.1723415897	1.8454405565	11.9848092973
O	2.7743399231	-0.0982385904	12.6839265286
O	3.4344430786	1.6690634546	13.7183158099
N	1.3209755695	2.9659454793	3.3335623529
O	1.4611974161	3.7636958706	2.3917820930
O	0.6416970211	1.9373013771	3.1802982814
O	1.8600327052	3.1968393762	4.4286075658
N	3.7656109255	0.4738557743	0.0212447315
O	3.5069760459	-0.6954547300	0.3511520738
O	2.8438158908	1.2870464861	-0.1577320580
O	4.9460417902	0.8299758536	-0.1296859428
N	5.9974805645	-3.4186532308	4.5519605353
O	6.9071891962	-3.4135048251	5.3977865282
O	4.8742623312	-3.8571132637	4.8505430381
O	6.2109903380	-2.9853412548	3.4075511184
N	-2.9289000822	6.7333825217	3.2859339947
O	-2.3966766233	7.6085563349	3.9886621764
O	-4.0317266321	6.2623777182	3.6098876747
O	-2.3582965318	6.3292131866	2.2592513064
N	4.6828910556	5.3825423669	7.8194011330
O	5.8279105440	4.9663653765	7.5770274656
O	4.1800227347	5.1999244211	8.9404685032
O	4.0407393711	5.9813377853	6.9407067228
N	6.0903428819	8.3261122887	8.0207551080
O	4.9768690015	8.8757792978	8.0533128646
O	6.4760127823	7.6465800768	8.9864220339
O	6.8181474479	8.4559775961	7.0225296218
N	9.8354489596	4.1447782241	8.4921408583
O	10.5287426089	3.1268007730	8.6536454579
O	9.9875802048	4.8453276050	7.4776929493
O	8.9900233845	4.4622065500	9.3450848544
N	-3.7571408584	3.1409515598	6.3902498333
O	-3.8180159522	4.3565802928	6.1421391373
O	-3.5635194924	2.7536145021	7.5545086882
O	-3.8898872376	2.3126592178	5.4741009369
N	5.6661319014	2.3864473901	10.5588101667
O	5.2348246418	1.2244117751	10.6404507698
O	5.1218702055	3.2977225468	11.2040811997
O	6.6417016423	2.6372080503	9.8318979455
N	7.6143706540	3.6996181251	4.4057001117
O	7.0732340237	3.1411763459	5.3743754120
O	7.0719696992	3.6690469700	3.2886125951
O	8.6979091114	4.2886315336	4.5541124473
N	0.2036917287	-0.2525451633	7.5200097026
O	-0.4378821992	0.1616715427	6.5403029473
O	0.0073015908	0.2388083452	8.6438515751
O	1.0416564692	-1.1581161068	7.3758744694
N	4.6695056586	-0.5612595839	6.3668888440
O	4.3291352144	-1.0926722400	5.2969512474
O	5.8719165566	-0.4914582528	6.6707863883
O	3.8074645108	-0.0996478872	7.1329295129
N	-0.9609608985	4.2399488060	4.7192074042
O	-1.6249742632	3.6797289733	3.8313685104
O	-0.4448557513	5.3488582775	4.5024744532
O	-0.8130525619	3.6912587256	5.8237801384
N	-2.1754574639	1.7756091657	1.2626053968
O	-2.1339347404	0.6779416050	1.8426223798

O	-3.0952209343	2.5735250665	1.5083355023
O	-1.2972160101	2.0753610670	0.4368576436
N	1.1907002159	8.3138035079	6.1952491239
O	1.6022408750	8.9297372917	5.1981143432
O	1.9133662859	8.2043661293	7.1996370581
O	0.0564925737	7.8073066950	6.1879959645
N	2.7960395421	2.6816064326	7.5862999275
O	3.6078796808	2.8969350398	6.6711130116
O	3.2024634602	2.3423056586	8.7100047351
O	1.5777745047	2.8055786991	7.3777818681
N	4.6093257184	5.9956372871	12.4566306289
O	3.6924764803	5.3525681063	12.9940945013
O	5.7887314704	5.7914529653	12.7887883412
O	4.3467689933	6.8428914717	11.5870083440
N	6.6346834815	5.1978147683	0.0647508200
O	6.8098928611	6.3908211661	0.3631721982
O	5.4879539793	4.7750961215	-0.1573714555
O	7.6062043863	4.4275263972	-0.0115483441

n=30 OPLS/AA

N	2.7507303181	2.1541926997	3.7744855216
H	2.1031844100	1.7182147976	3.1075195079
H	2.5332481416	3.1540974546	3.8589051251
H	3.7147028930	2.0406828849	3.4396603121
H	2.6517851972	1.7037752371	4.6918564915
N	-1.0416666205	8.7129796068	6.6566380871
H	-0.3257717929	8.0456304230	6.9670825432
H	-1.3774612040	8.4454583496	5.7239489320
H	-0.6366758532	9.6556691893	6.6172127981
H	-1.8267569348	8.7051598153	7.3183083776
N	1.1876228789	7.3192062371	2.7453213698
H	0.3990392124	7.6370954676	3.3209239544
H	1.5411435241	8.1040922285	2.1856680669
H	0.8740386494	6.5672063203	2.1205041322
H	1.9362693619	6.9684312414	3.3541898860
N	1.7289733081	1.3775853580	14.0327517583
H	1.3623074531	1.5365010435	13.0869515399
H	2.4766292644	0.6747899748	13.9963662409
H	0.9729530513	1.0418374247	14.6409805153
H	2.1040031065	2.2572131436	14.4067078162
N	6.2334982215	3.2694635245	13.5930027153
H	6.7544756543	3.8714467149	12.9445932378
H	5.7575962453	2.5286627817	13.0648301546
H	5.5376362839	3.8312355534	14.0974071913
H	6.8842852101	2.8465096342	14.2651796461
N	4.7545444750	6.5318328004	1.1737254041
H	5.1450289956	6.7838196811	0.2581597053
H	4.7336274099	7.3622763462	1.7771826760
H	5.3393200089	5.8080183120	1.6077365622
H	3.8002018658	6.1732171077	1.0518217811
N	-2.8241519262	5.1613776889	11.0043545688
H	-3.5052662796	4.4018316417	11.1201523988
H	-2.5598583698	5.5276146491	11.9264468041
H	-3.2422442190	5.9135776533	10.4443281655
H	-1.9892394999	4.8024860719	10.5264910195
N	4.8632958960	-0.6512788856	5.7568073516
H	4.8312466066	-0.7612491027	4.7364566806
H	4.4418717041	0.2479769073	6.0175281890
H	5.8397669424	-0.6761878234	6.0732237932
H	4.3402982997	-1.4156556305	6.2000197501
N	1.2300056665	-1.9577936939	4.9363105210
H	0.2840369356	-2.3570163146	4.9370567265
H	1.1719205013	-0.9396956006	5.0560480320
H	1.6910725983	-2.1717317642	4.0441875976
H	1.7729917093	-2.3627314848	5.7079497287
N	2.6542201802	11.2265555146	5.2214107241
H	2.1316262946	10.3462949498	5.3006134163
H	3.4027423536	11.2446014038	5.9239942333
H	2.0184099319	12.0173545246	5.3783112597
H	3.0641016316	11.2979703228	4.2827240645
N	0.8955474753	6.9423531695	13.2899991130
H	-0.0186768732	6.4771548911	13.2450491396
H	1.3452448555	6.9031023577	12.3677918014
H	1.4881724141	6.4664073275	13.9802938047
H	0.7674486143	7.9227476486	13.5668616623
N	1.8430739243	4.2298500771	11.1709525837
H	1.2004698559	4.1034351922	11.9617214410
H	2.7149816409	4.6580842207	11.5035591642
H	1.4073347682	4.8407589608	10.4701264656
H	2.0495088064	3.3171218116	10.7484040340
N	-2.6850289812	-0.7671771395	3.3874609504
H	-1.7019154493	-0.7770974790	3.0914449579
H	-2.9780849127	0.2000000591	3.5689028779
H	-2.7900943770	-1.3230903252	4.2442907565
H	-3.2700202283	-1.1685208224	2.6452049211
N	-3.0984702861	0.8721579366	7.2165656818
H	-3.6365711359	1.5865136635	6.7122052268
H	-2.0965352933	1.0098305639	7.0393449062
H	-3.3786012377	-0.0605185382	6.8911785778
H	-3.2821740017	0.9528067530	8.2235335250
N	-0.0468817176	-0.8581218133	9.0639794502
H	-0.6173689768	-1.3645398585	8.3767232039
H	0.9369450865	-1.1334884951	8.9615455904
H	-0.3694198188	-1.0868135066	10.0115578410
H	-0.1376837167	0.1523541138	8.9060904963
N	3.5781938632	5.7708442672	7.9423184817
H	3.7121158288	4.9744165427	7.3082807190
H	4.2944690287	5.7471720168	8.6775899944
H	2.6465164896	5.7147883607	8.3701859040
H	3.6596742362	6.6469993727	7.4132166920
N	-1.7019211008	1.3591755286	11.7996203643
H	-1.9042856097	2.1328312266	12.4436246842
H	-0.9389181811	1.6301120381	11.1682293303
H	-2.5414203706	1.1435651569	11.2491763003
H	-1.4230604389	0.5301944464	12.3374517697
N	5.0733373343	0.7155024041	9.7187382109
H	5.9418464217	1.0720588433	9.3030513477
H	4.7112778426	-0.0559126898	9.1459674341
H	4.3773899510	1.4694570470	9.7567534760
H	5.2628359676	0.3764067633	10.6691801810
N	4.6759334695	7.1331618235	13.1309396020
H	4.6709846044	6.5776673984	13.9944426709
H	4.1398551686	7.9966952633	13.2764206625
H	4.2493502550	6.5893095211	12.3716722193
H	5.6435438451	7.3689745700	12.8812236962
N	6.4295049873	3.6683652407	4.8766098245
H	7.1153583677	4.3988650266	4.6525310415
H	5.5918825400	4.0991196315	5.2853566605
H	6.8386494055	3.0095949431	5.5495571213
H	6.1721303041	3.1658820729	4.0189942564
N	5.4215205708	8.3579398807	5.0313882838
H	6.0305824802	7.5314072008	5.0202789566
H	5.2783234920	8.6904973725	4.0705892430
H	5.8615190808	9.0998470376	5.5883538176
H	4.5156578233	8.1100071070	5.4463311070
N	3.0995726658	10.8593910446	9.6190924269
H	2.9768946697	11.1579548529	10.5937953864
H	2.5784682163	11.4909174112	8.9995286397
H	2.7462527959	9.9018283213	9.5074043588
H	4.0966748618	10.8868638838	9.3756422721
N	6.6121164486	8.0846519605	9.3755298615
H	6.7965831768	9.0810447151	9.2099714051
H	6.6492745616	7.5769310346	8.4838609647
H	5.6810357013	7.9738619558	9.7939193221
H	7.3215725345	7.7067711070	10.0143675926
N	3.1631254119	1.3841082662	0.2928915208
H	4.0077795200	1.9536394091	0.4210703510
H	2.3627927142	1.8555684373	0.7304158808
H	3.3001693998	0.4653819408	0.7303802731
H	2.9817608362	1.2618438324	-0.7103002968
N	-3.3278048153	4.9531502141	7.1409316436
H	-3.7489372922	5.5695202082	7.8458937453
H	-2.9446934195	5.5202265867	6.3755005681
H	-4.0439128843	4.3173408878	6.7705652597
H	-2.5736760755	4.4055137738	7.5717676879
N	7.5450779081	4.0628836923	8.9400673002
H	6.5666490290	4.2802394821	9.1629338225
H	8.1561838597	4.4497901233	9.6688248834
H	7.7896120504	4.4770421593	8.0329226913
H	7.6678657403	3.0444632163	8.8955880209
N	2.8872657961	-2.4927218661	11.4333464238
H	3.1524510224	-3.4762141048	11.5624042008
H	2.0961724901	-2.2680482289	12.0481047693
H	2.6158018258	-2.3369311786	10.4554547638
H	3.6846381046	-1.8896949100	11.6674220868
N	-1.0738622032	3.3088368407	2.7787565230
H	-0.5562544007	3.0248529806	3.6187990383
H	-0.5778267439	4.0790821111	2.3151772612
H	-2.0153916728	3.6195549595	3.0456123501
H	-1.1459754911	2.5118570349	2.1354382604
N	-0.5329400133	9.1305998252	10.5829383606
H	-1.5505368088	9.0123859402	10.6519190588
H	-0.1708823319	9.5033829657	11.4684786764
H	-0.3151558070	9.7844371695	9.8218192563
H	-0.0951860964	8.2221931104	10.3895365179
N	0.5072893307	4.5782391360	6.4385241800
H	0.2020220952	4.6738830979	5.4628704274
H	-0.2199224181	4.0951066757	6.9788766425
H	0.6695402439	5.5095501121	6.8392360853
H	1.3775171044	4.0344167513	6.4731126147
N	0.6736934666	11.2254208433	7.6375282686
O	0.1657572346	11.0447987780	6.5184239996
O	1.6429360753	11.9920859049	7.7632714981
O	0.2123867184	10.6393773754	8.6308901076
N	-0.9621433285	2.9004448237	9.0517668216

O	-0.7025671200	1.6859659135	9.0778259761
O	-1.2824244536	3.4442929093	7.9818756254
O	-0.9014383632	3.5710761882	10.0955997036
N	3.4648391478	4.6889592202	14.6887723311
O	2.6252367462	5.6008960747	14.6084175460
O	4.6727556656	4.9632740094	14.7820836026
O	3.0965247351	3.5027066214	14.6758158342
N	-3.1741492959	7.7832847688	9.3659740079
O	-2.9174449822	8.0198881273	10.5580896303
O	-3.7520126172	6.7293547414	9.0524084006
O	-2.8529900296	8.6006120958	8.4874232855
N	0.2183316920	1.2164289392	5.5517984882
O	0.0486476333	2.2678171472	4.9124174851
O	-0.6784404314	0.7926759367	6.2996293931
O	1.2847887328	0.5887932283	5.4433484990
N	5.4995010018	10.4515193065	7.5766167200
O	6.3842222164	9.8440029592	6.9511487582
O	4.5409069476	10.9535098822	6.9665996612
O	5.5733739009	10.5570451629	8.8121027353
N	-3.6212698173	2.7029685674	4.6117157035
O	-3.3879438155	1.5893643382	4.1132555663
O	-3.3518424260	3.7362738190	3.9771236702
O	-4.1240236152	2.7832676097	5.7447687861
N	3.5428151687	-1.1316966801	2.5418166025
O	2.9798210626	-2.1281817858	3.0245929293
O	3.5104616084	-0.9330458603	1.3160478601
O	4.1381633142	-0.3338617520	3.2848096163
N	3.8474712556	2.7610288630	6.8996298176
O	3.9933252942	1.7060524294	6.2602791823
O	4.8042091178	3.2440099549	7.5276424280
O	2.7448784673	3.3330246651	6.9109678517
N	1.5636007642	4.6487501851	1.2212918829
O	0.4281572480	5.0684067377	1.4999929959
O	1.8057753513	3.4314840155	1.2725993161
O	2.4568704123	5.4463604441	0.8912830711
N	2.0262549098	1.1823386157	10.7002806644
O	2.7646973867	2.0252762194	10.1643918555
O	1.1280291101	1.5475526871	11.4767000136
O	2.1860383613	-0.0258140320	10.4597499305
N	5.0587892919	4.4836064400	10.7898164743
O	4.3220541713	4.8401604249	11.7242192402
O	5.2729115770	5.2538286905	9.8390655907
O	5.5814025480	3.3568292977	10.8061646052
N	-1.9558420981	-1.9894565770	6.1508992347
O	-1.3168501199	-2.4903333502	5.2107771040
O	-1.4875699916	-2.0108738980	7.3012385080
O	-3.0631070742	-1.4671620624	5.9406819231
N	-3.9203409158	2.3293849481	9.8955776354
O	-4.1799730712	2.9679539100	8.8622148067
O	-3.5338920103	1.1514509846	9.8171765377
O	-4.0471577679	2.8687503838	11.0073424569
N	2.0923816462	7.9740518012	6.0967156404
O	1.7218081262	8.8162884091	5.2622513371
O	3.2732647389	7.9591585260	6.4818276016
O	1.2820714213	7.1467078024	6.5460683443
N	2.1046210621	9.8211510835	12.7325915492
O	0.8905348652	9.5601978967	12.7628578959
O	2.5080091787	10.8313150723	12.1327043481
O	2.9153197951	9.0719396782	13.3022128620
N	3.8261912335	9.4612091321	2.3916570472
O	3.7095684436	10.5544403226	2.9698115248
O	4.8773355551	8.8087406519	2.5030051883
O	2.8916689496	9.0204460671	1.7021538732
N	3.1198096145	-1.9664718639	8.0442269697
O	2.2407589678	-2.3038366421	8.8544571085
O	3.0036887506	-2.2614983318	6.8431885693
O	4.1149819264	-1.3340801087	8.4350355458
N	3.4022428233	5.4788778130	4.4321129362
O	3.4830747606	6.5547603119	3.8165148876
O	4.2723678223	5.1723701111	5.2639503155
O	2.4512851214	4.7095023967	4.2158734313
N	2.9443477770	7.7634725184	10.4138155085
O	2.5580860050	6.9428646892	11.2625925964
O	2.1156920128	8.4335788774	9.7756109936
O	4.1592662914	7.9139741098	10.2032427660
N	0.0195505692	0.3722987164	2.2227787473
O	-0.1491449345	-0.7019679940	2.8232042405
O	-0.9445119531	0.9404706023	1.6835366198
O	1.1523095071	0.8783939484	2.1615953324
N	-0.9312534331	4.3041865879	13.2224871296
O	-1.8144550091	3.4346629042	13.3055620983
O	-1.2449694284	5.5048254274	13.1671105783
O	0.2656651017	3.9730711657	13.1947886899
N	7.6082946262	6.1389062018	11.6540971910
O	7.0534731921	7.1672307325	12.0756891107
O	8.1890442504	6.1521358963	10.5561114169
O	7.5823664154	5.0973511382	12.3304915899
N	7.2919729517	1.1979688895	7.4081882951
O	7.0072942251	0.0460231898	7.0407688638
O	7.5303912198	2.0779068848	6.5644562495
O	7.3382334475	1.4699768128	8.6193407469
N	0.1204411665	6.1556360279	9.2634199128
O	-0.1206166416	6.4833884975	10.4370839885
O	-0.6928166424	6.4247881798	8.3638718227
O	1.1747576324	5.5587309260	8.9893037064
N	7.1879353426	6.1255289526	6.3626493998
O	7.0635075394	6.3730525015	5.1517539767
O	8.0118625038	5.2766753895	6.7416079362
O	6.4884354214	6.7268594509	7.1945869561
N	-1.2371901533	6.5553685929	4.5951964310
O	-0.5602564546	5.5757020958	4.2415974590
O	-2.1635893245	6.4088005482	5.4096412080
O	-0.9877244800	7.6816040414	4.1343502550
N	4.5681760937	-0.0075020275	12.7424116358
O	5.1314936983	1.0990586210	12.7075588243
O	3.7010830582	-0.2362313363	13.6019757590
O	4.8719517691	-0.8853340739	11.9176996600
N	-0.0574845264	-1.1446891985	12.6389875481
O	-0.4096442033	-0.2818523975	13.4602756387
O	0.8819634383	-1.9115355948	12.9080638143
O	-0.6447732869	-1.2406796803	11.5486223136
N	5.3985467816	3.3770581445	1.8910547785
O	5.9611025944	4.4590384012	2.1274180425
O	5.1736761194	2.5788822795	2.8159149759
O	5.0608613591	3.0932535244	0.7298303823

n=31 OPLS/AA

N	-1.0818005130	12.2967791094	2.8072453901
H	-0.4687377520	11.7088770999	3.3841001170
H	-2.0609470627	12.0356632100	2.9725487679
H	-0.8536809161	13.1594061349	1.8156176175
H	-0.9438357240	13.2831694204	3.0567156199
N	-4.1948060163	9.9342091044	0.8701145261
H	-3.6582455808	9.0827269631	1.0733807546
H	-3.5484314508	10.7062785248	0.6692578194
H	-4.7743887548	10.1756339768	1.6825389252
H	-4.7981577562	9.7721961237	0.0552808031
N	4.0839762791	0.8801702487	4.3920757977
H	3.7383945724	0.6502880188	3.4529466575
H	5.1061226011	0.9741354005	4.3671332229
H	3.8249610916	0.1312027256	5.0449113865
H	3.6664265147	1.7650546262	4.7033110092
N	3.7391815514	5.6284994032	-0.9752433641
H	3.7149973523	4.8377734663	-0.3207177994
H	4.1377025154	6.4497813493	-0.5052309262
H	2.7851429242	5.8428171011	-1.2884745028
H	4.3188833900	5.3836249262	-1.7865495904
N	-5.5559064173	7.9731789968	4.5835935364
H	-5.3954057285	7.7368401417	3.5973787666
H	-6.2956112465	7.3710989114	4.9638184988
H	-5.8429257481	8.9562224233	4.6576276457
H	-4.6896827899	7.8285542805	5.1155482739
N	-0.7826386242	-0.2012867625	8.0433287691
H	0.1926174460	-0.4949126560	7.9133459632
H	-1.2758232750	-0.2443572581	7.1438019390
H	-0.8056188414	0.7610683993	8.4005121622
H	-1.2417288765	-0.8269458211	8.7156548855
N	-0.7171936423	-0.0196156417	-0.3461080286
H	-0.4380535661	-0.1329936598	-1.3276692037
H	-1.6347729853	-0.4561134534	-0.1986207573
H	-0.7734557077	0.9802639583	-0.1195889574
H	-0.0224920384	-0.4696195223	0.2614458480
N	-1.3933509499	5.6189346238	0.4713727390
H	-0.6271593079	5.4319646403	1.1288137558
H	-1.9405764706	4.7616047663	0.3307867138
H	-1.0052548837	5.9284893564	-0.4274002218
H	-2.0004123911	6.3536795499	0.8532913483
N	-1.5926060843	9.2919639374	5.4669838967
H	-0.8616020159	10.0089561254	5.3908829641
H	-1.5330498524	8.8387059017	6.3863559072
H	-1.4609886183	8.5894180748	4.7298667570
H	-2.5147831388	9.7307763461	5.3608298843
N	-6.9143418798	0.1012203561	-1.4920496561
H	-6.4506763273	0.6006597648	-2.2600409516
H	-7.7238162861	-0.4136246269	-1.8580011755
H	-7.2290951746	0.7763902106	-0.7854298466
H	-6.2537792797	-0.5585434378	-1.0647273986
N	3.0269033277	7.8280542063	-4.0869021978
H	3.5850247439	6.9681899788	-4.0288496992
H	3.4324183880	8.4521984656	-4.7941759531
H	3.0274794671	8.2987190411	-3.1743728528
H	2.0626912552	7.5931085024	-4.3502102296
N	-5.0254901192	3.8506906658	3.1928949594
H	-4.6938581986	4.6921682293	2.7069197781
H	-4.2791542919	3.1456498020	3.2045095563

H	-5.8452253888	3.4733021973	2.7031526234
H	-5.2837222743	4.0916432539	4.1569974064
N	1.1545351199	9.8149550347	-0.0388655266
H	2.1554117875	9.9399413901	0.1531251108
H	0.6866295170	10.7287948549	-0.0248721264
H	0.7415421790	9.2079583402	0.6789278752
H	1.0345579710	9.3831256754	-0.9626427791
N	3.2125458837	1.6221256954	7.8608655388
H	4.1800568812	1.6042406731	7.5175891292
H	3.1413120720	2.2754333986	8.6497568333
H	2.9416647223	0.6812629003	8.1701164420
H	2.5871508017	1.9275657923	7.1059994164
N	-2.9057311711	0.5961159533	-3.2459137113
H	-3.3877660099	-0.2873385327	-3.0424765390
H	-1.9739164614	0.3973426914	-3.6285953317
H	-2.8113570138	1.1415309136	-2.3811295975
H	-3.4498856688	1.1329278803	-3.9314531790
N	3.2462434025	1.6878221022	-1.7332782016
H	4.1836897373	1.9396894566	-2.0679322060
H	2.6776555603	1.3598267517	-2.5228107223
H	2.8015054660	2.5106575338	-1.3097388949
H	3.3221237592	0.9411149120	-1.0326313093
N	-0.5084285919	5.3089673843	4.3667872442
H	-1.1873053232	5.2749529464	5.1363361376
H	-0.5141940238	6.2457312942	3.9464602022
H	-0.7620834401	4.6131702940	3.6556183815
H	0.4298677581	5.1020149693	4.7287350049
N	-3.5087378400	2.4970496731	6.1948443773
H	-3.4111173465	2.2587621739	7.1887887443
H	-4.2394138337	3.2098234145	6.0839350845
H	-3.7674709796	1.6563782148	5.6651612779
H	-2.6169491051	2.8632346571	5.8414933707
N	-4.4124926689	0.1120500441	2.1408239274
H	-3.9680869962	0.9186913829	1.6868752010
H	-4.5742693772	-0.6258249174	1.4454078482
H	-5.3094657111	0.4002763109	2.5489911784
H	-3.7981481584	-0.2449418142	2.8820210398
N	-5.8246138922	4.7440666399	-0.5031077476
H	-6.0071491713	5.7410819676	-0.6670510481
H	-4.8544259384	4.6206327667	-0.1904832144
H	-5.9726108743	4.2212517341	-1.3743120816
H	-6.4642697625	4.3933010622	0.2194151940
N	0.6562436601	4.3248318140	8.1380790800
H	0.8648192997	4.3626246583	7.1334378500
H	0.1493279550	5.1738472746	8.4145639820
H	1.5336571869	4.2623323257	8.6676778348
H	0.0771704020	3.5005230342	8.3366356749
N	0.9272041990	4.0757518720	-4.1298834772
H	0.9841102516	4.2824858160	-3.1257625307
H	0.4159130919	4.8282859242	-4.6058126627
H	0.4340642659	3.1859332445	-4.2687047312
H	1.8747292420	4.0063027048	-4.5192530062
N	-2.6574571530	4.8090140707	-3.0906578657
H	-3.2980155312	4.0940312252	-3.4549662413
H	-2.0265137403	4.3839115405	-2.4011414161
H	-3.1990914923	5.5577150112	-2.6430925987
H	-2.1062084722	5.2003978093	-3.8634315616
N	-3.4096626854	6.1454892024	8.7011742067
H	-4.3012390127	5.9084846294	8.2504452607
H	-3.5916916789	6.5626187959	9.6215562106
H	-2.8982673179	6.8167599390	8.1162760102
H	-2.8474536007	5.2940932144	8.8164189062
N	5.9721278043	4.6577049486	3.8874999957
H	5.8904701544	4.6348958654	2.8642464243
H	5.0425838975	4.7929748981	4.3020899757
H	6.5864996995	5.4322891837	4.1646316635
H	6.3689573865	3.7706598251	4.2190309229
N	0.0191124062	-0.6552848395	3.7790592628
H	-0.9839591927	-0.8673975357	3.7234564810
H	0.1479482811	0.3514282969	3.9345091383
H	0.4756319103	-0.9271096483	2.9004599573
H	0.4368276490	-1.1780606774	4.5578114207
N	1.3303563488	2.8146202358	2.1384689635
H	1.1884879558	3.8315035989	2.1460638653
H	0.8678442974	2.4106625791	1.3155865300
H	0.9293517425	2.4067292501	2.9911446191
H	2.3357412613	2.6095865054	2.1010808470
N	6.4326532317	5.7262319270	7.9751713339
H	5.7878794042	5.4752629273	8.7337999453
H	7.3261715724	6.0370854453	8.3742073605
H	6.0249067123	6.4850352153	7.4164264281
H	6.5916546101	4.9075438759	7.3762523405
N	2.6348663196	7.3855809441	5.9536389561
H	2.7460662143	6.6145719059	5.2847475269
H	3.4878412309	7.4745835190	6.5182158133
H	1.8358416213	7.1923996750	6.5688430304
H	2.4697163201	8.2607679256	5.4427488024
N	-2.0483114139	8.8373067559	-2.6226604090
H	-3.0559850882	8.7728419741	-2.4364504318
H	-1.8817756267	8.7660180130	-3.6333128875
H	-1.5615940137	8.0723582593	-2.1407937326
H	-1.6938919084	9.7380087145	-2.2800844028
N	3.6696455579	8.7123436995	2.5413259094
H	4.3927024127	8.4699169186	3.2288243737
H	3.2058000927	7.8560236542	2.2160597879
H	4.1031071436	9.1911137782	1.7431243124
H	2.9769732870	9.3323202108	2.9772958333
N	-0.4718606594	6.8997989827	-4.9396203063
O	0.7599429304	6.9980412647	-5.0662152550
O	-0.9993834364	5.7789249829	-4.8480828432
O	-1.1761420392	7.9224315239	-4.9045627923
N	1.2958896177	10.3026659840	3.9897252878
O	0.3225491252	10.8653774976	4.5179127063
O	2.0146166935	9.5442614699	4.6614930263
O	1.5505032394	10.4983591421	2.7897691649
N	-4.1447066177	-1.2120812476	-0.7232643448
O	-4.2263215805	-1.2447618556	-1.9623320938
O	-3.0314843329	-1.1506840633	-0.1755559905
O	-5.1763147701	-1.2407978472	-0.0319043935
N	-3.1335079756	6.1753895693	5.5268037664
O	-3.5151375497	7.2366302920	6.0475195310
O	-3.4876296816	5.8879870919	4.3713750389
O	-2.3977561033	5.4015507010	6.1615172401
N	-2.8975316070	-0.3208908686	4.8862024740
O	-4.0062125685	0.2281005603	4.9977948010
O	-2.0830349340	-0.2717259098	5.8227898364
O	-2.6033470816	-0.9190477379	3.8380219408
N	0.1536989284	2.1528134014	5.6000588440
O	0.8138149974	1.8109649390	6.5952494363
O	-0.8812446568	2.8249100781	5.7421705734
O	0.5285267463	1.8225649213	4.4627556066
N	4.2632036943	3.5935246796	1.4606531446
O	5.2213846113	4.3832718295	1.4259158383
O	3.9993840442	2.9828456572	2.5096947237
O	3.5688418685	3.4144564079	0.4463480551
N	-1.3635325442	11.5479881455	-0.2626860266
O	-0.3440807467	11.9559609997	0.3180842325
O	-1.2614148080	10.9229322479	-1.3312809001
O	-2.4851029807	11.7650713638	0.2251389805
N	0.3384341885	3.3040769152	-1.1315040042
O	-0.6469790058	3.8029123356	-1.6999597816
O	1.4860985917	3.6222709753	-1.4845434760
O	0.1761828489	2.4870467770	-0.2100080131
N	2.1335706261	-0.2060600387	1.2798527391
O	1.0765690920	-0.8533857180	1.3619205938
O	2.6518721563	-0.0012850136	0.1696957125
O	2.6722710636	0.2364909717	2.3079427387
N	6.1949722593	2.5333054278	6.1100135726
O	6.6441847397	3.6030346037	6.5537539794
O	5.5748425091	1.7613668627	6.8600588081
O	6.3658896669	2.2355145773	4.9162269691
N	5.7768245485	7.4325799620	5.4117872539
O	5.2776779960	7.5926260059	6.5379559331
O	6.8044096097	6.7473057352	5.2795522847
O	5.2483856145	7.9578085678	4.4178527439
N	-5.9131001687	5.1566303841	6.3264258189
O	-5.4867982155	4.1346003712	5.7636299338
O	-5.5674114892	5.4141872649	7.4914072355
O	-6.6850914231	5.9211041316	5.7242398027
N	2.8404211113	4.2438351937	5.3896812212
O	3.1313244535	3.0397026942	5.4816490429
O	3.7002448953	5.0647588267	5.0293961117
O	1.6896930587	4.6270443685	5.6579987248
N	4.1532315638	4.4112552239	-3.7218050959
O	3.4587355518	3.9183434313	-4.6260899873
O	4.3359181247	5.6391584906	-3.6782493953
O	4.6650414269	3.6762631582	-2.8610752121
N	-4.1478432744	6.8593912800	1.5366363758
O	-3.2182903920	7.4736632776	0.9874376297
O	-4.1771596750	5.6180013029	1.5033289521
O	-5.0480804809	7.4865097642	2.1191430145
N	-1.9209128269	2.6541786238	8.8597951974
O	-2.1875356364	3.8632990458	8.9595849441
O	-0.7523697455	2.2612303393	9.0118367160
O	-2.8228338250	1.8380058293	8.6079637294
N	1.7292124207	6.2204500194	2.1660764173
O	2.3961167401	6.7597097564	1.2674911538
O	0.8889506750	5.3515885266	1.8796190629
O	1.9025699867	6.5500520406	3.3511199893
N	3.6489205619	4.7585035091	9.0769152285
O	2.9318374231	3.7730161252	9.3169658465
O	3.2911235006	5.5959406554	8.2321086235
O	4.7238016272	4.9065538658	9.6816717023
N	1.9565831058	-0.9684888993	6.6702758187
O	1.8117522683	-0.6967262152	7.8736822267
O	1.0211483517	-1.4787359827	6.0318185800

O	3.0368495671	-0.7300043080	6.1053261945
N	-5.2910390681	2.5213688096	-3.2845535677
O	-5.6531818932	1.3363695926	-3.3720026443
O	-4.2479146332	2.8950819634	-3.8460228927
O	-5.9720212262	3.3326555259	-2.6356346437
N	4.0470212159	8.8626259769	-0.5989248971
O	3.7704269970	9.8215576160	0.1406450188
O	3.6459013308	8.8490167711	-1.7744824407
O	4.7247358655	7.9173027827	-0.1629369185
N	-4.7824176106	7.6260964970	-1.5919203043
O	-4.0038950468	6.7821124374	-2.0658521936
O	-5.8531871462	7.2634562969	-1.0771751267
O	-4.4901704035	8.8327217281	-1.6327336255
N	-3.3722288525	2.6415837433	-0.2727893234
O	-2.7967662117	1.8107684762	-0.9950127320
O	-4.1027897036	2.2572764223	0.6554383625
O	-3.2171305174	3.8567073095	-0.4787937665
N	0.2175679550	1.0067813930	-3.5284735314
O	1.4491483827	1.1662299622	-3.5001236696
O	-0.4979756313	1.8063127102	-4.1543848351
O	-0.2984693020	0.0478007347	-2.9309116083
N	-0.9729189233	8.5059279966	2.4664304637
O	-0.9482370337	7.7245675347	3.4317721357
O	-1.6518482806	9.5444933206	2.5252089170
O	-0.3186709288	8.2487229276	1.4423095140
N	-7.0512218839	2.2236352639	1.4079819660
O	-7.0076614737	3.4646262582	1.4405665284
O	-6.6071299422	1.5613499249	2.3604410926
O	-7.5388746283	1.6449291428	0.4229374839
N	-1.8407924195	2.4451317422	2.6994946707
O	-2.9159890304	2.3139284026	3.3075701943
O	-1.3218866814	3.5684764616	2.5906305937
O	-1.2845010989	1.4529895637	2.2002828222
N	-0.6138480826	7.2483461354	7.8491710526
O	-1.4952001914	8.1217803453	7.7913000566
O	-0.5673541903	6.4763607392	8.8212280349
O	0.2210108060	7.1468972400	6.9349843303
N	-4.2160921144	10.8049773692	3.9248940857
O	-3.9458184360	10.2818215102	5.0186390272
O	-5.2035903946	10.4178560464	3.2783632365
O	-3.4988669352	11.7152552836	3.4776796335
N	0.5434155705	7.1226526068	-2.0233106785
O	-0.6078895415	6.7378726362	-1.7597602579
O	1.4583722536	6.2942248862	-2.1632381266
O	0.7797641897	8.3358612746	-2.1469337505

n=32 OPLS/AA

N	-1.6768252884	-11.0735242292	5.8251476984
H	-2.4553779301	-10.6233343394	5.3297479155
H	-0.7862321790	-10.7377192222	5.4400326555
H	-1.7387645699	-12.0915486227	5.7067188859
H	-1.7269272328	-10.8414942939	6.8240908541
N	-5.7341693907	-11.9641693248	7.8562857650
H	-5.3001224847	-12.5139343712	7.1055542719
H	-5.0166009381	-11.6872649036	8.5364726091
H	-6.4500427627	-12.5311253293	8.3256701793
H	-6.1699109546	-11.1243532306	7.4574452687
N	-12.6266234647	-6.0606811397	-0.9822274097
H	-12.2310573654	-5.6963422223	-0.1075727853
H	-13.5985979916	-6.3510233966	-0.8234773596
H	-12.0754398750	-6.8677015998	-1.2971085804
H	-12.6013982414	-5.3276569854	-1.7007500616
N	-15.3095492880	-4.3874280660	3.0274602766
H	-15.7075375560	-4.6863138386	3.9255185882
H	-15.7895418276	-4.8742710793	2.2614147637
H	-14.3086716502	-4.6152948022	3.0038757745
H	-15.4324465058	-3.3738328351	2.9190328547
N	-10.6967259064	-11.1325708611	8.9708527284
H	-10.5422276140	-12.0189410999	8.4761667927
H	-10.4177852213	-11.2359481417	9.9535733995
H	-11.6912742170	-10.8821448955	8.9219035940
H	-10.1356164229	-10.3932501705	8.5317666456
N	-10.0696204386	-6.8192960876	8.3585299048
H	-10.7630889090	-7.4802192159	8.7280135940
H	-10.5138629789	-5.9060655062	8.2072250594
H	-9.7010208455	-7.1723948046	7.4676364431
H	-9.3005096965	-6.7185054672	9.0312448825
N	-9.0891209835	-4.4559129591	-1.1950906671
H	-9.4430317443	-5.2755726550	-1.7021816391
H	-9.6995585030	-3.6507348193	-1.3775430811
H	-8.1364277547	-4.2420467692	-1.5126948056
H	-9.0774662765	-4.6552983913	-0.1879436365
N	-11.1748436291	-9.1829209488	1.2708441362
H	-10.1836260990	-9.2986665839	1.5123536672
H	-11.7057055468	-9.9957929551	1.6049989074
H	-11.2720592188	-9.1061192931	0.2515864854
H	-11.5379826865	-8.3311050759	1.7144377202
N	-7.0707127259	0.4174104163	5.3496429002
H	-7.4029987479	-0.5491891590	5.2521372172
H	-6.1738705390	0.5219721731	4.8607915769
H	-6.9458393146	0.6376615200	6.3446964699
H	-7.7601426256	1.0591961897	4.9409462418
N	-13.5464393120	-5.9284406274	6.5183709863
H	-12.8164521336	-6.2983996537	5.8983023322
H	-13.6469915443	-6.5439870488	7.3339851949
H	-14.4375946043	-5.8852822128	6.0102222676
H	-13.2847182549	-4.9860939547	6.8309735468
N	-3.2234077049	-2.3912789500	1.5306790032
H	-2.3603053266	-2.8311621518	1.1903950763
H	-3.3114994959	-2.5473373649	2.5416786535
H	-4.0308063823	-2.8040204459	1.0490283441
H	-3.1910187741	-1.3825962658	1.3416136075
N	-15.1530430021	-2.5563539117	-0.6711727037
H	-15.3139683400	-2.2920640080	0.3078523113
H	-15.2227742654	-3.5763224850	-0.7662324008
H	-14.2166522431	-2.2489987532	-0.9591669346
H	-15.8587773167	-2.1080301433	-1.2671428372
N	-4.6634891669	-3.7926744708	7.9564122761
H	-5.3004281647	-4.5008886388	8.3397887268
H	-3.6934926579	-4.1133309195	8.0589837147
H	-4.7876012274	-2.9094762574	8.4651289323
H	-4.8724352378	-3.6470027575	6.9617481039
N	-7.5280508436	-8.5388376359	4.1264062538
H	-8.4940645120	-8.2037623457	4.0327139915
H	-7.5213785243	-9.4218749051	4.6502720255
H	-6.9701074057	-7.8385436339	4.6289228752
H	-7.1266538734	-8.6911693327	3.1937160316
N	-1.4776429978	-8.6584919573	1.4626548339
H	-0.8485687888	-8.9948818253	2.2011286613
H	-1.4450004613	-9.3040722607	0.6649110015
H	-1.1800451428	-7.7238954472	1.1590096395
H	-2.4369569857	-8.6111186235	1.8255707524
N	-7.0522866871	-0.9468750584	1.7046733458
H	-7.2343597475	-1.6296669710	2.4495764955
H	-7.7846768167	-0.2273181629	1.7134629802
H	-7.0522092498	-1.4240631576	0.7955380224
H	-6.1379011120	-0.5064526073	1.8601166104
N	-10.7943114613	-11.6622574592	4.8294569400
H	-11.7335236984	-11.9391252271	4.5204897731
H	-10.5222291217	-12.2250039731	5.6440256891
H	-10.7976212582	-10.6680673160	5.0859925682
H	-10.1238726814	-11.8168335902	4.0673194286
N	-6.8927192565	-12.8969662482	4.3743060153
H	-7.3749777327	-13.2975591979	5.1874407817
H	-6.5713657662	-11.9482902891	4.6000947315
H	-7.5391993957	-12.8587674478	3.5775376642
H	-6.0853346010	-13.4832484481	4.1321516757
N	-6.4269790367	-4.8721304390	2.9221805608
H	-7.0772765526	-4.7893711718	2.1319273469
H	-6.9349244072	-5.2053149582	3.7499572591
H	-6.0121578485	-3.9543174900	3.1216255317
H	-5.6835579718	-5.5395180557	2.6852113358
N	-4.5261233370	-6.1622269478	-0.6464374296
H	-4.8544990161	-6.3797080814	0.3017753458
H	-5.2605600254	-5.6564441824	-1.1553787135
H	-4.3058717257	-7.0347234068	-1.1408812391
H	-3.6835629008	-5.5780323323	-0.5912641882
N	-5.6679516518	-7.8550660072	7.8404416921
H	-6.3951951537	-8.5777144315	7.7844752612
H	-6.0232490892	-7.0524891551	8.3732350975
H	-5.4135155223	-7.5514447070	6.8931683556
H	-4.8398182918	-8.2386164391	8.3108879997
N	-2.8270756048	-0.5644552903	5.9670837009
H	-2.9164866547	-0.4808184625	4.9476492289
H	-3.7224630672	-0.3267709931	6.4098275912
H	-2.0990713392	0.0781697382	6.3006678525
H	-2.5702814450	-1.5284013624	6.2101891383
N	-11.8995581133	-1.6416017928	4.7637729680
H	-12.8097562589	-1.7743855303	4.3075450507
H	-11.2680016198	-2.4057378162	4.4964394332
H	-12.0243426070	-1.6414047661	5.7829217870
H	-11.4961328540	-0.7448791880	4.4681851566
N	-1.5523042639	-5.1859410427	3.9877024462
H	-2.5705821048	-5.3011919726	4.0514485014
H	-1.1093408723	-6.1066632567	3.8862691004
H	-1.2052828935	-4.7312559638	4.8403883037
H	-1.3240121769	-4.6046530897	3.1727039414
N	-11.4111461184	-0.8755259186	0.9493279835
H	-10.6862066250	-0.2211404913	1.2663114863
H	-12.3377463971	-0.4617364478	1.1056126047
H	-11.3346862116	-1.7529109804	1.4771393704
H	-11.2859445339	-1.0663151177	-0.0517512186
N	-14.1408917535	-9.5662317708	5.8532034783
H	-13.2945865890	-9.1489007776	5.4484337805
H	-14.9286366208	-8.9168100874	5.7439140147

H	-13.9869255317	-9.7526547833	6.8510894372
H	-14.3534174484	-10.4465610282	5.3693762864
N	-15.9009222127	-8.5176807243	1.9496003518
H	-15.4356130889	-7.9784735210	1.2100199919
H	-15.2415859775	-9.1998533640	2.3422209117
H	-16.7102231971	-9.0115842779	1.5554852410
H	-16.2162661342	-7.8808112091	2.6906745424
N	-10.3096671142	-5.4695445599	3.3217787220
H	-10.0078881588	-4.6585484179	3.8744547730
H	-11.3343638871	-5.4775639456	3.2572272048
H	-9.9886255049	-6.3322717915	3.7766078470
H	-9.9077906120	-5.4097932947	2.3788256015
N	-1.6979703128	-7.2923308517	7.4809717100
H	-2.5015408665	-6.9494644384	6.9415710375
H	-1.0501467610	-7.7844365568	6.8545602588
H	-2.0251391657	-7.9370114196	8.2100691186
H	-1.2150552408	-6.4984106582	7.9176858997
N	-7.4107471381	-9.3468671785	-0.4003394884
H	-7.2244018823	-8.3790295490	-0.1125863544
H	-8.0274409174	-9.3471451369	-1.2212678598
H	-7.8656517684	-9.8512938675	0.3696287037
H	-6.5254938030	-9.8099992180	-0.6371321630
N	-8.3920823638	-2.7825907221	7.7564798002
H	-8.2986678932	-3.3353494157	8.6166935300
H	-8.0047121338	-3.3113172331	6.9661461574
H	-7.8803813120	-1.8985201835	7.8604838399
H	-9.3845680253	-2.5851765946	7.5825965114
N	-4.5165304144	-11.3273562902	2.0521059064
H	-4.3014640969	-10.5557406374	2.6944390450
H	-5.5245244480	-11.3394781536	1.8570782822
H	-4.2404275676	-12.2178351758	2.4822804018
H	-3.9997053357	-11.1963704427	1.1746265221
N	-4.1770725940	0.1487838985	3.0984125759
O	-4.6163071590	-0.0318489328	1.9506048010
O	-4.9194596276	0.6073343324	3.9825001864
O	-2.9954500442	-0.1291339278	3.3621329527
N	-9.6355677198	0.6937140495	3.0877883726
O	-10.7223553144	0.3030716817	3.5452915699
O	-8.8745102323	1.3769885027	3.7927319401
O	-9.3098373504	0.4010817286	1.9253406720
N	-3.0828224648	-9.9148902079	8.7796892020
O	-3.8053864267	-10.8476518043	9.1681155606
O	-3.4475502624	-8.7383655870	8.9402549549
O	-1.9955298298	-10.1586534286	8.2306966486
N	-0.4189137429	-8.5854208921	4.4315192440
O	-0.3751703253	-8.6203170703	5.6724409503
O	-0.4773213755	-7.4932938356	3.8425668251
O	-0.4042495160	-9.6426526216	3.7795494318
N	-8.3742587703	-9.9603645371	6.8735656371
O	-7.2844427609	-9.8634117209	7.4617145365
O	-9.4103798763	-9.5363707258	7.4118037003
O	-8.4279537170	-10.4813115838	5.7471777675
N	-3.8327939794	-13.3331661863	4.8938579314
O	-2.5998116936	-13.3749111273	5.0388750118
O	-4.3267271478	-13.3942069618	3.7557358115
O	-4.5718436919	-13.2303803872	5.8869637704
N	-8.7717428827	-2.6359019623	4.2384195497
O	-8.3071146857	-2.7929181820	3.0971551385
O	-8.2206377379	-1.8544503653	5.0312842493
O	-9.7874770422	-3.2603378424	4.5868195418
N	-6.2439104890	-3.4126489051	-0.4018601188
O	-5.3288912962	-3.4645370934	0.4366298357
O	-7.0619931300	-2.4783942425	-0.3714146147
O	-6.3408471187	-4.2950160898	-1.2707962768
N	-5.6948945738	-0.6375172797	7.9412410718
O	-5.1426880464	-1.4019331045	8.7497918177
O	-5.0078075324	0.0964191099	7.2116749784
O	-6.9341891404	-0.6070378198	7.8622563556
N	-12.0577726303	-3.9346709507	1.3558320182
O	-13.2631964516	-3.9867686770	1.0604388073
O	-11.6622978487	-3.1252087426	2.2110445893
O	-11.2478229387	-4.6920360422	0.7960122073
N	-16.1874091086	-6.7991404643	4.8951992109
O	-16.7482113269	-6.9412978878	3.7959676385
O	-15.8250248975	-5.6704033993	5.2662089582
O	-15.9889909418	-7.7857209000	5.6234216223
N	-12.7876230817	-8.8399632811	8.5736856770
O	-13.5244733841	-7.9297682596	8.1594217931
O	-11.7884405678	-8.5666869866	9.2592309213
O	-13.0499555041	-10.0234355497	8.3024040983
N	-4.5572253658	-2.7344771779	4.3984170018
O	-5.3917160601	-2.5719800679	3.4927471495
O	-3.3582680432	-2.8959874402	4.1165771809
O	-4.9216922876	-2.7354640264	5.5859276311
N	-8.3219548888	-11.3313160760	2.1322672456
O	-8.9767280077	-11.9824855173	2.9630911936
O	-7.1438354760	-11.6411722265	1.8892822126
O	-8.8453016039	-10.3702897106	1.5444278575
N	-1.6615290953	-4.0126990549	7.0997739459
O	-1.9937817615	-2.8413069899	6.8538741558
O	-0.8173840855	-4.5813720636	6.3877008299
O	-2.1734218509	-4.6154185964	8.0577476233
N	-10.2148190488	-7.9443102071	-1.7577553476
O	-9.3240251322	-8.7639674290	-2.0364418160
O	-10.0569947557	-6.7411864942	-2.0234670988
O	-11.2634381028	-8.3277770069	-1.2133566898
N	-11.9031624806	-2.6597911952	-1.5987646955
O	-12.1849698333	-3.7411176687	-2.1412917313
O	-12.7969525373	-1.8278881834	-1.3705222266
O	-10.7275641249	-2.4103675327	-1.2844798754
N	-10.9896938521	-8.0968105952	5.0516269761
O	-10.1115195556	-8.0308138320	4.1755720446
O	-11.3072612831	-7.0799833960	5.6905567725
O	-11.5503011690	-9.1796354294	5.2887523022
N	-14.5248884021	-1.4557523583	2.3121868064
O	-14.1339691352	-1.7719906811	3.4480535947
O	-15.5403446510	-1.9881211876	1.8342224278
O	-13.9003509175	-0.6071445230	1.6542838670
N	-7.3182004468	-5.6037821722	9.4587472207
O	-7.9556904983	-4.5449453766	9.5832019739
O	-6.1351072453	-5.5757470395	9.0812258058
O	-7.8638040361	-6.6906549754	9.7118140863
N	-8.8554098387	-13.0909927030	7.3027391182
O	-8.3530655641	-12.7452077883	8.3849132878
O	-8.1553684878	-13.6537225806	6.4446627213
O	-10.0577964322	-12.8740475656	7.0786411650
N	-13.0707988827	-6.8369212235	3.1185675024
O	-13.0142485855	-5.6943578625	3.6026841629
O	-12.4808230651	-7.0908637588	2.0553367493
O	-13.7173255179	-7.7255427645	3.6976820613
N	-1.6347467880	-5.0018129109	0.7735480429
O	-1.2111880465	-6.1680537492	0.8326937140
O	-1.2362454230	-4.1541695079	1.5894617848
O	-2.4568075564	-4.6832152190	-0.1015120747
N	-11.6008298498	-3.4658328173	7.2840424518
O	-11.1396783541	-4.5018038538	7.7911136701
O	-10.9284493394	-2.4213609293	7.2849565167
O	-12.7343627684	-3.4743336754	6.7760567598
N	-4.6680793415	-10.2457499165	5.1171626225
O	-5.7786544260	-10.5860780136	4.6769153295
O	-4.5533093748	-9.8829429751	6.2996248370
O	-3.6722734220	-10.2682287790	4.3749471033
N	-7.7821516068	-5.7908937220	5.9034419006
O	-7.2991905405	-4.7650215198	6.4107413626
O	-8.0343614313	-6.7765957036	6.6160425735
O	-8.0129030343	-5.8310639751	4.6835407837
N	-4.4369905123	-6.4512168462	5.1733513406
O	-3.7670624579	-6.4347089871	6.2192680694
O	-4.0834726771	-5.7673486024	4.1984835156
O	-5.4604372258	-7.1515935129	5.1023023797
N	-15.5215298112	-5.9545248032	0.0251026285
O	-16.2545827766	-5.6803147219	0.9897062217
O	-14.9576628475	-7.0598813610	-0.0319836082
O	-15.3523436731	-5.1233776576	-0.8824154586
N	-3.9921802354	-9.7922746052	-0.5975460519
O	-3.0278451424	-10.3229536335	-0.0218243239
O	-5.0764117667	-10.3934087452	-0.6756334592
O	-3.8722837006	-8.6604605257	-1.0951807732
N	-13.4630897579	-11.0740864716	3.2093959041
O	-14.5066765972	-10.4862059539	2.8802440902
O	-12.4841919299	-11.0768897604	2.4446939394
O	-13.3984006945	-11.6591641715	4.3032505634
N	-5.0277089928	-8.0509955789	1.9941217219
O	-5.1219432712	-6.8319609347	1.7748220456
O	-3.9130973931	-8.5619081745	2.1931875712
O	-6.0480871355	-8.7591181975	2.0143555651
N	-8.2569538307	-6.5294889245	0.9404497373
O	-7.4068310230	-6.7877524120	0.0723431230
O	-8.4768253766	-5.3502457264	1.2630328957
O	-8.8872055999	-7.4504693764	1.4859736323

n=33 OPLS/AA

N	5.0979847879	12.0549908040	-6.8985242025
H	5.0479088541	11.9137702681	-5.8827558930
H	5.1075523886	13.0609020821	-7.1041589443
H	4.2809132972	11.6221570996	-7.3449373379
H	5.9555645631	11.6231336285	-7.2622436456
N	-0.0406718516	4.1915192627	-2.3856241400
H	0.9046262638	3.7969741080	-2.4562052390
H	-0.0480250779	5.1364296158	-2.7872770249
H	-0.3197289543	4.2370186885	-1.3985615990
H	-0.6995587173	3.5956542541	-2.9004527656
N	0.0854119754	-1.8216747405	-8.8279214288
H	0.6070180709	-1.9839427931	-7.9585352902
H	-0.2887996118	-2.7129929471	-9.1739737774

H	0.7126707806	-1.4170404274	-9.5329412781
H	-0.6892408300	-1.1727229524	-8.6462345226
N	-0.0571604100	7.5114528876	3.3593992268
H	0.0091099925	6.8623035777	2.5666511553
H	-1.0390880552	7.6039072821	3.6448899795
H	0.3061733733	8.4304055496	3.0805621940
H	0.4951631140	7.1491945086	4.1454928063
N	3.5207540470	5.7427114121	-4.8852489991
H	4.3763528922	5.2309655921	-5.1308155468
H	2.7016100654	5.1595487447	-5.0929967461
H	3.4714109608	6.6097531379	-5.4330075496
H	3.5336431027	5.9705776752	-3.8841763931
N	0.8211568609	2.1129226503	3.4965959488
H	1.6492289610	1.5058929247	3.5034957195
H	0.8888769620	2.7753219350	2.7150104697
H	0.7695797932	2.6315234989	4.3812588733
H	-0.0230574661	1.5389516515	3.3866187393
N	-2.9512202198	2.9064722731	-5.6039816417
H	-3.7914734383	3.3187621510	-5.1818114355
H	-3.1789509987	1.9809324376	-5.9857530269
H	-2.2204211566	2.8114392885	-4.8890431932
H	-2.6140361040	3.5147556171	-6.3593185001
N	6.7115480613	9.5626154660	-3.9280980669
H	6.2713434429	10.4870731786	-3.8517179046
H	7.4473170610	9.4741271699	-3.2174304329
H	7.1236013006	9.4558976126	-4.8624743037
H	6.0039300119	8.8333648034	-3.7807695521
N	2.8083413385	1.9187855874	-6.1667944772
H	3.4851831355	1.6042086187	-5.4616968881
H	1.9893914863	2.3238675813	-5.6983241973
H	2.5141861499	1.1190456399	-6.7396157979
H	3.2446052416	2.6280202033	-6.7675403390
N	-1.7016119286	7.7732116575	-0.9579566309
H	-2.3105092188	7.6988262435	-0.1345812622
H	-1.8238975782	6.9433993250	-1.5501495035
H	-0.7225779568	7.8362486050	-0.6550468268
H	-1.9494635536	8.6143723838	-1.4920481292
N	4.4716833182	3.5729875139	-1.8974920197
H	3.5411387478	3.2867629730	-2.2236797147
H	4.5687642329	3.3486440705	-0.9002558386
H	4.5859419749	4.5842652963	-2.0334983804
H	5.1908874108	3.0722774368	-2.4325344628
N	8.2610834710	6.7299929270	-6.6442467821
H	7.8724030625	6.1824038126	-7.4209769795
H	7.6679175961	6.6151865561	-5.8140610033
H	8.2933572619	7.7219588304	-6.9073010977
H	9.2106555851	6.4004219756	-6.4346488045
N	8.6077740139	1.8460071407	-2.4770782725
H	8.7624124955	2.2525295540	-1.5469908760
H	7.6149740829	1.9281541916	-2.7257444028
H	8.8771480415	0.8552677180	-2.4666593589
H	9.1765615864	2.3480774951	-3.1689175462
N	4.1540821657	2.8330585273	-10.5977140681
H	4.1250012427	3.4095318431	-11.4468718354
H	3.4489302360	3.1693294390	-9.9314428656
H	5.0860052936	2.8980777361	-10.1716538490
H	3.9563918624	1.8552956523	-10.8408885497
N	-0.2556590421	2.3522133610	-8.7136039491
H	-1.0010714903	1.6514277689	-8.8001830085
H	0.0344891720	2.4236813936	-7.7312898346
H	-0.6034356581	3.2632859676	-9.0349038946
H	0.5473810819	2.0704576314	-9.2880391431
N	3.4319552322	4.5749449197	3.9656848490
H	3.0462503016	4.3865925320	3.0329516706
H	4.2628488948	5.1717853340	3.8783239497
H	3.6927848311	3.6886007156	4.4135658558
H	2.7259365255	5.0528009137	4.5378970115
N	2.4516786413	6.4136661397	0.2750587881
H	1.4721628992	6.7202904996	0.3027447125
H	2.4906813884	5.4124359310	0.0508902350
H	2.8873398842	6.5766769817	1.1904069573
H	2.9565294395	6.9452614453	-0.4438067256
N	9.0777543853	6.0768193794	-2.2804904985
H	9.4291431837	5.6879363567	-1.3975794019
H	9.7493820754	5.8810418184	-3.0320369561
H	8.9585523085	7.0920516611	-2.1838995925
H	8.1739403157	5.6462473027	-2.5084451837
N	3.7467768538	-1.1024827749	-3.2571389099
H	3.7718986432	-1.1073441225	-4.2835800240
H	3.0888160101	-1.8184821138	-2.9274918863
H	3.4419633250	-0.1794031816	-2.9266116020
H	4.6844294613	-1.3047016862	-2.8908731272
N	1.6645073172	10.1545255849	-2.1952039348
H	1.8522296503	9.2479042073	-2.6390891689
H	1.9176265588	10.1078866460	-1.2012270013
H	0.6665365450	10.3790064662	-2.2840757291
H	2.2216366976	10.8833041370	-2.6564242722
N	-3.3786422534	3.7720869291	0.3362038881
H	-3.7315956967	4.4632748150	1.0084532993
H	-3.9072416818	3.8467733049	-0.5408589803
H	-3.4915293783	2.8283258868	0.7245428075
H	-2.3842026007	3.9499743828	0.1526790806
N	-0.5788310922	0.5350393439	0.2261159062
H	-0.9994333688	0.3773645010	1.1494080953
H	-1.2361119576	1.0577366877	-0.3646497295
H	-0.3683762491	-0.3689125504	-0.2129914337
H	0.2885967969	1.0739685837	0.3326975918
N	-2.1696466652	6.7326612106	-5.0759239870
H	-2.2552613811	6.8098889522	-6.0961896848
H	-2.9046462202	6.1121542616	-4.7167840896
H	-2.2699220507	7.6627377159	-4.6526769369
H	-1.2487570923	6.3458639879	-4.8380462306
N	5.4015569408	7.6314477845	-9.0772518000
H	4.6633050819	8.2588172083	-9.4172928217
H	5.4656495091	6.8127071555	-9.6935233397
H	5.1777088183	7.3250099864	-8.1231955029
H	6.2995636350	8.1292573988	-9.0749958669
N	3.6945790704	0.1709851240	0.3553403761
H	3.4769202095	0.3047493135	1.3498090078
H	3.3346683311	0.9671793083	-0.1838940497
H	3.2546857317	-0.6948789884	0.0221606744
H	4.7120417975	0.1068909926	0.2332868405
N	1.1184264420	6.8981034714	-7.8389791993
H	1.1699304376	7.8445718451	-8.2336702856
H	2.0275431685	6.4329220333	-7.9455047493
H	0.3982155301	6.3594393015	-8.3343302652
H	0.8780166820	6.9554816274	-6.8424118817
N	-0.3127409589	-0.8332103146	-4.1894691164
H	-1.0909965467	-0.5441070032	-3.5853386561
H	-0.6664797532	-1.0285450805	-5.1333694713
H	0.1226240328	-1.6799429723	-3.8051038547
H	0.3838876735	-0.0802459208	-4.2340638950
N	4.8802833823	9.8654043463	0.1515382950
H	5.8043838985	10.0945224969	-0.2328823166
H	4.3489230499	10.7306416627	0.3040350780
H	4.9939297305	9.3716541269	1.0445840679
H	4.3738977503	9.2647993218	-0.5095840237
N	7.1126566592	6.2471383221	1.6218343925
H	6.8256398013	6.6678300983	2.5133924951
H	7.4828157266	6.9774316597	1.0022469561
H	6.3020616498	5.8000082556	1.1777105828
H	7.8401091797	5.5432836845	1.7939884042
N	6.9648337491	3.0790155403	-6.6223594338
H	6.2993109159	3.6862461220	-6.1298287477
H	6.7196495410	3.0396738880	-7.6186383225
H	6.9262646893	2.1339653088	-6.2228246899
H	7.9141092019	3.4561774338	-6.5181454956
N	-2.9201601247	4.4955572299	4.5512954740
H	-2.7872580258	3.5146035436	4.2787097168
H	-3.2303665087	5.0381319725	3.7366671332
H	-2.0327468459	4.8773843150	4.8990615933
H	-3.6302689889	4.5521081333	5.2907431870
N	1.6567959645	10.0573860929	-5.7342453086
H	1.5950299982	10.3282610886	-6.7227025913
H	0.7132111758	10.0134228280	-5.3318156090
H	2.2147617599	10.7515805151	-5.2233599991
H	2.1041808642	9.1362802038	-5.6591039979
N	7.2342347129	1.5077588985	1.9804652910
H	6.3993073399	1.4602828298	2.5761820040
H	7.0199729290	1.1097639440	1.0585506585
H	7.9957226258	0.9736021995	2.4152621442
H	7.5219351440	2.4873865746	1.8718669374
N	1.2248748732	9.3482001030	0.7921253755
O	0.6815976148	8.5031973325	0.0614831586
O	0.8599008991	9.4750978801	1.9726798484
O	2.1331268369	10.0663056746	0.3422127574
N	2.7233414576	9.9053401909	-8.6278860829
O	1.6103670625	9.3975029875	-8.4124625892
O	3.5138609277	9.3583347818	-9.4145765943
O	3.0457966421	10.9601836525	-8.0566186054
N	6.7015079957	-0.4681166907	-1.0703090220
O	6.3159011221	0.1367385479	-0.0561712650
O	7.9064695335	-0.4694403073	-1.3721100351
O	5.8821526720	-1.0716487986	-1.7826463394
N	1.2904544921	-1.8249845923	-1.5098117664
O	2.3004848062	-1.6831613637	-0.8007681519
O	1.4021025256	-2.2483541077	-2.6722707468
O	0.1687752414	-1.5434380790	-1.0563960356
N	1.9316460343	3.2413677137	0.6496055378
O	2.1851228159	3.7513235823	1.7535586366
O	1.5029401765	2.0771925022	0.5869535210
O	2.1068752517	3.8955875834	-0.3916963823
N	0.8169932162	7.2914810987	-4.0661190755
O	0.1228139821	6.6490811175	-3.2608518118
O	0.5872337565	7.2136117634	-5.2843821292
O	1.7409326538	8.0117509952	-3.6531229528

N	2.1653807198	0.4113035287	-10.6184423567
O	1.6263954535	1.4577712743	-10.2216829194
O	1.5429089689	-0.6628051328	-10.5755772977
O	3.3268386722	0.4389444670	-11.0580672068
N	3.4729870919	4.4761430109	-7.8857690858
O	3.4205642519	5.7076135026	-8.0398800359
O	4.2479534264	3.9876242448	-7.0468444727
O	2.7504430157	3.7331906873	-8.5705833002
N	7.5566251485	9.6980714444	-7.4346320809
O	7.1806426334	10.8498520883	-7.7085963259
O	7.8846782653	9.4120565417	-6.2711912621
O	7.6045545853	8.8323050062	-8.3241093707
N	-3.5310288890	4.9661857962	-2.7873128653
O	-3.7535817188	4.9717863247	-4.0093835601
O	-4.2444054811	4.2884220976	-2.0291914254
O	-2.5950987137	5.6383495075	-2.3233632370
N	6.3812040983	4.6433818079	-9.2804402626
O	7.0894402492	5.2938520299	-8.4941146585
O	6.3206690524	3.4064410213	-9.1838436298
O	5.7335024718	5.2298528447	-10.1633632103
N	-2.0043259567	-0.0718636944	-7.2265268343
O	-1.6631746617	0.1268307708	-8.4043019462
O	-2.9192543935	0.6007427590	-6.7230147437
O	-1.4305483530	-0.9431653144	-6.5522632701
N	3.8945758668	1.1900188957	3.5014340165
O	5.0802750969	0.9083398179	3.2610340503
O	2.9841710342	0.6551072670	2.8471783317
O	3.6192812477	2.0066102596	4.3960903878
N	-3.1345086890	6.6280856274	2.2501583362
O	-3.0021771925	7.2662137950	1.1926618928
O	-2.6615980288	7.0747143371	3.3084098252
O	-3.7397513330	5.5433278769	2.2494032898
N	-2.3154523305	1.4776213704	2.7343198880
O	-2.9929847263	1.5693061939	1.6972270008
O	-2.6633537237	2.0869051388	3.7593837012
O	-1.2900177161	0.7766522141	2.7463489718
N	8.8711150692	3.8993739433	0.5322414616
O	8.8819886566	2.8473590267	-0.1281858934
O	9.3152336286	4.9483415892	0.0368189208
O	8.4161225562	3.9024212164	1.6880922879
N	6.5932669193	5.1546883606	-3.9489082742
O	7.1191850098	6.1878341947	-4.3950447539
O	6.8408960981	4.7823132608	-2.7900146269
O	5.8197190273	4.4939170944	-4.6616660157
N	4.5126081331	7.3198145754	2.7697564262
O	3.3036393832	7.0913014062	2.5988933110
O	5.0582144646	8.2595130102	2.1678440623
O	5.1759710856	6.6086287374	3.5425325273
N	0.3428107868	3.8667820930	-5.6372304522
O	-0.6225136486	4.4802950348	-5.1527012832
O	0.1667064865	2.7678283093	-6.1888696166
O	1.4842404414	4.3522233257	-5.5701204026
N	7.8069454719	8.8375789195	-1.0875085641
O	7.7776424189	8.0051927852	-0.1659378585
O	7.1920880479	9.9110027908	-0.9746836925
O	8.4511064675	8.5965409884	-2.1219049741
N	5.1622379870	3.8774462775	1.1497933874
O	5.0162122193	4.9719681895	0.5808327823
O	4.8961813318	2.8240514750	0.5476379611
O	5.5743207417	3.8363191347	2.3209103614
N	-1.8030356970	1.6198764078	-2.4992685701
O	-1.9867836596	0.4002448775	-2.6467593407
O	-1.5669973053	2.3362467119	-3.4862424178
O	-1.8553261681	2.1231380393	-1.3648030385
N	-1.5338842127	5.2558167175	-8.0358354744
O	-2.2607207713	4.3136322778	-7.6794438224
O	-1.7659600299	6.4069301446	-7.6307470841
O	-0.5749710650	5.0468875619	-8.7973161298
N	4.4252631233	8.3599926886	-6.1746879284
O	3.2417885129	7.9826322209	-6.1768867500
O	5.3346152378	7.5540847375	-6.4327751562
O	4.6993858400	9.5432620599	-5.9144016695
N	4.1722513756	11.4928662237	-3.5944877575
O	5.1759508435	11.8047619447	-4.2565499512
O	4.2976688121	11.1096647740	-2.4195643445
O	3.0431335622	11.5641720099	-4.1073493897
N	9.6661189266	4.4479454117	-4.9621105137
O	9.2425813024	4.1519728359	-6.0917272745
O	10.0601291024	5.6027771740	-4.7294358629
O	9.6956463986	3.5890855339	-4.0651676816
N	2.0674014181	-1.0753168534	-6.5583702819
O	3.0123843762	-0.9686010274	-5.7592227033
O	1.7940079083	-0.1375360211	-7.3257300479
O	1.3958114291	-2.1198143524	-6.5901581200
N	0.4600828716	5.0790725516	5.2444174103
O	-0.7077238407	5.4736841325	5.3978028516
O	1.3575581878	5.8899252045	4.9614440141
O	0.7304144855	3.8736073472	5.3740054694
N	2.1515903744	1.7091475171	-2.9902604682
O	2.1871138715	2.9473247265	-3.0833904326
O	2.8015071926	1.1342583075	-2.1013687780
O	1.4661495072	1.0458589833	-3.7860228348
N	-1.2420850387	9.6626884319	-3.4845905345
O	-0.9503232188	10.0604388331	-2.3445521274
O	-2.2033991320	8.8928804284	-3.6467286323
O	-0.5725322263	10.0347463337	-4.4624916311
N	4.1871477858	7.3482851243	-2.2548354226
O	3.8894636881	6.1439354263	-2.3176358512
O	3.7593781513	8.0458898974	-1.3202881359
O	4.9126021021	7.8550304570	-3.1265829825
N	-0.5475521719	4.9113351886	1.1848616698
O	-0.8705258823	4.4547546393	0.0756997811
O	-0.0117349966	6.0291967217	1.2642559919
O	-0.7603958083	4.2500536725	2.2146300653
N	5.8350951034	1.4545744191	-4.1477663982
O	6.0967284149	1.9051741719	-3.0201468773
O	4.6526719666	1.3199120247	-4.5037993689
O	6.7558856698	1.1386368062	-4.9193535696

n=34 OPLS/AA

N	13.5947172199	-6.7273636679	-4.7662308784
H	14.2056041468	-6.4563663821	-5.5457266971
H	12.6293749670	-6.8189916474	-5.1038006246
H	13.6345428825	-6.0084301865	-4.0342581399
H	13.9093474784	-7.6256661918	-4.3811388113
N	3.6678365621	-3.1263589061	-5.6377330195
H	3.9243908451	-3.8308451237	-6.3392422259
H	3.8515327085	-2.1880267146	-6.0119296382
H	4.2252632008	-3.2710005251	-4.7876801572
H	2.6701597440	-3.2155639474	-5.4120807398
N	11.9578073844	-11.1955597738	-5.6826190134
H	11.8728727204	-10.3555091471	-6.2668596701
H	11.0402643437	-11.4312581201	-5.2866445907
H	12.6234642692	-11.0172209241	-4.9214805959
H	12.2946281217	-11.9782500859	-6.2554917657
N	8.6793633753	-12.7896406907	-7.1246685907
H	7.8252470041	-13.0847828947	-7.6121236158
H	8.4616115947	-12.5943617198	-6.1404506418
H	9.0505748675	-11.9411421057	-7.5679379259
H	9.3800192029	-13.5382763300	-7.1781626542
N	9.2458469117	-7.8753111019	-6.4401576911
H	9.2738472410	-7.3431623783	-7.3178078578
H	10.1995933555	-7.9857171176	-6.0762718325
H	8.8365723650	-8.8007949858	-6.6139663510
H	8.6733747125	-7.3715694078	-5.7525855779
N	1.1319556339	-8.0895670039	-6.0747670925
H	0.6147100377	-8.6649559888	-6.7497638382
H	1.6761459471	-8.6983093471	-5.4522525560
H	0.4697926085	-7.5357386329	-5.5188446272
H	1.7671734386	-7.4592646070	-6.5782080058
N	7.9881866560	-3.5620815202	-14.8111798730
H	8.7565650167	-3.3911088840	-14.1519385287
H	7.0919206515	-3.3645918363	-14.3508107173
H	8.0087073929	-4.5430346007	-15.1137447046
H	8.0955543111	-2.9495905932	-15.6282248994
N	12.6319946949	-2.4555440718	-5.6547243998
H	13.4833102063	-2.9182251356	-5.9944658173
H	12.6240211428	-1.4786580230	-5.9707382531
H	12.6135819157	-2.4834493127	-4.6285091245
H	11.8070663437	-2.9418442667	-6.0251847351
N	-0.2206186928	-4.6304911222	-8.0626511160
H	-0.7212779324	-3.8794160984	-8.5520004106
H	-0.2714338014	-4.4721120148	-7.0494539148
H	0.7623467184	-4.6363876520	-8.3592649313
H	-0.6521102434	-5.5340479919	-8.2898856839
N	11.4964947210	-11.8914439516	-10.7278904801
H	10.6110716550	-11.5904339393	-10.3040331169
H	11.3209531493	-12.2357982055	-11.6791212565
H	11.9085783741	-12.6429216115	-10.1624668217
H	12.1453748432	-11.0966217568	-10.7659403126
N	9.2156388496	-5.4036138302	-2.4237721320
H	8.9208426066	-6.2014388340	-1.8486142963
H	8.4524309426	-4.7187588974	-2.4759458532
H	9.4475133390	-5.7303493010	-3.3691364414
H	10.0417682232	-4.9639090656	-2.0013913771
N	4.6389946984	-12.0213444871	-9.5842403360
H	4.3280057697	-12.2721664828	-8.6384019194
H	4.2193815581	-11.1244857859	-9.8559091934
H	4.3462801119	-12.7503788109	-10.2453474754
H	5.6623110508	-11.9383471132	-9.5973018347
N	2.8303556161	-10.1919699978	-13.6703630707
H	2.4438537796	-9.8827597409	-12.7707845888
H	2.6394217977	-11.1925723439	-13.7991027589
H	3.8447083642	-10.0331143490	-13.6797922494
H	2.3934381464	-9.6594332561	-14.4317718095

N	12.7375923693	-6.8369369896	-0.7818140461
H	11.7666560027	-6.8085532031	-0.4490783700
H	12.8665090685	-6.1365950435	-1.5215006883
H	12.9443440205	-7.7708795432	-1.1549635944
H	13.3728594400	-6.6317201410	-0.0017132078
N	11.7672482523	-3.1187315436	-9.4304925270
H	10.8915651988	-3.6311853387	-9.2729920659
H	11.7263707387	-2.2142889461	-8.9461916885
H	11.8960476313	-2.9624522869	-10.4370823332
H	12.5550085875	-3.6670001017	-9.0657038669
N	3.5335535598	-4.5160811262	-13.1551546646
H	3.9717682985	-5.0415171581	-12.3895693221
H	2.6707204141	-4.0740840715	-12.8169354285
H	3.3105566286	-5.1565357502	-13.9260799316
H	4.1811693249	-3.7921880366	-13.4880332307
N	6.7847282108	-4.8850152902	-8.7110087795
H	7.7643144649	-4.8847678629	-9.0186559917
H	6.2521178978	-5.5657768135	-9.2651970943
H	6.3844765694	-3.9486621208	-8.8424503621
H	6.7380048653	-5.1408543635	-7.7177319693
N	6.2533590369	-13.4089898909	-12.3882052530
H	6.3720409091	-13.8523106869	-11.4697187900
H	5.9545949140	-12.4348004606	-12.2619925829
H	5.5415000748	-13.9185118497	-12.9247494875
H	7.1453003654	-13.4303369983	-12.8963592569
N	4.3338980252	-8.2216151721	-7.9360917085
H	4.2394700870	-7.4187613506	-7.3030473155
H	5.3289647658	-8.4033199733	-8.1123308425
H	3.8612548050	-8.0149155728	-8.8238524793
H	3.9059023509	-9.0494630097	-7.5051355801
N	7.0260081888	-8.8160922685	-2.7539679265
H	7.4473078544	-9.7424245910	-2.8905370214
H	7.1612713438	-8.2504650811	-3.6001383875
H	7.4740111599	-8.3506366816	-1.9559203348
H	6.0214428075	-8.9208436227	-2.5692760952
N	0.6497144392	-6.7075447341	-11.4353046215
H	-0.0504188111	-7.4384142510	-11.2624483849
H	1.4677780542	-6.8686761068	-10.8361024119
H	0.2448597466	-5.7884264185	-11.2218808083
H	0.9366380853	-6.7346628717	-12.4207867127
N	12.8143840075	-7.4293707815	-8.9217252137
H	13.2294290545	-7.8170819812	-9.7770928143
H	12.0810708346	-6.7557113629	-9.1720639116
H	13.5414043848	-6.9556493968	-8.3728491396
H	12.4056321603	-8.1890407628	-8.3648958223
N	0.2737307940	-11.1522436518	-9.9307758477
H	0.9669648113	-11.4684639385	-10.6190108549
H	0.6774327617	-11.2071637316	-8.9883093010
H	-0.5579901263	-11.7520919740	-9.9823299474
H	0.0085164045	-10.1812552712	-10.1334539578
N	8.2497159138	-3.0144790877	-5.4729391161
H	7.4183160157	-2.8136398998	-6.0409830648
H	7.9880583940	-3.0401430407	-4.4804113384
H	8.9520811604	-2.2806928065	-5.6228600221
H	8.6404072752	-3.9234404079	-5.7475025921
N	3.9206711127	-6.3530383510	-3.5375010632
H	3.0391573265	-5.8358849021	-3.6361005184
H	4.3326532817	-6.5071909680	-4.4652630740
H	3.7379307167	-7.2586077605	-3.0894100943
H	4.5729422676	-5.8104692698	-2.9592306621
N	10.6356132136	-10.8305905083	-1.8726848008
H	9.9103216411	-10.8586906120	-2.5989069546
H	10.4312800853	-10.0697816356	-1.2141618191
H	11.5508400779	-10.6689315670	-2.3091039772
H	10.6500103437	-11.7249582461	-1.3685671594
N	11.0037723949	-6.0327615042	-12.6052374171
H	10.7914768093	-6.2090369218	-13.5942235945
H	11.3840540240	-5.0847886886	-12.5005086782
H	11.6949742812	-6.7170751508	-12.2763144632
H	10.1445842583	-6.1201454275	-12.0499038958
N	5.2249632464	-12.1372286020	-5.5001624280
H	4.6777534187	-11.6337565508	-4.7921275448
H	4.6797462014	-12.9329389303	-5.8520404361
H	6.0969440036	-12.4791186460	-5.0794531685
H	5.4454088290	-11.5030997903	-6.2770278729
N	8.0491927110	-8.4277357664	-10.3681542374
H	8.9579530503	-8.7938782983	-10.0610217094
H	7.6436959213	-9.0609200016	-11.0673571860
H	8.1775205203	-7.4971794948	-10.7826811037
H	7.4176022373	-8.3589656274	-9.5615566515
N	9.7877995176	-15.3870616492	-10.0652487267
H	10.5789162031	-14.8890794244	-9.6405321255
H	9.5609599075	-14.9599053533	-10.9709616920
H	8.9722187777	-15.3265349424	-9.4444427195
H	10.0391039526	-16.3727263916	-10.2050579560
N	5.8453213321	-7.1877352761	-14.5485910690
H	5.7636962277	-8.1740073038	-14.2750203703
H	6.5668663787	-7.0920220972	-15.2727777982
H	6.1057096805	-6.6258218769	-13.7296363914
H	4.9450129622	-6.8590907869	-14.9169294498
N	8.2398380057	-1.8332816532	-10.6338605611
H	9.1500304270	-1.6554428923	-11.0744955903
H	8.2889565171	-1.5913126131	-9.6372297162
H	7.9996058243	-2.8265605331	-10.7334652291
H	7.5207601408	-1.2598104012	-11.0902521379
N	3.4507408912	-2.6791763252	-10.0170012552
H	3.9127134400	-2.3199347807	-10.8606617167
H	2.4367692199	-2.5380473989	-10.0956063536
H	3.8033488990	-2.1772008817	-9.1936399912
H	3.6501324558	-3.6815218898	-9.9180977808
N	9.3818496183	-10.1425545635	-13.6590705720
H	8.6684531421	-10.1473689510	-12.9206408957
H	9.1261884096	-9.4549711892	-14.3774723643
H	9.4410462421	-11.0754483011	-14.0838535263
H	10.2917099848	-9.8924298175	-13.2543147827
N	7.7073075616	-5.3092371672	-11.9960864232
O	8.5107310840	-6.2075564048	-11.6951693217
O	6.9721171553	-5.4404340051	-12.9887093965
O	7.6390743904	-4.2797202629	-11.3043799944
N	2.8406446914	-12.8471883797	-12.1780495685
O	3.6228558193	-13.5111968696	-11.4778664584
O	1.9000343986	-12.2335975163	-11.6472233819
O	2.9990439837	-12.7967707127	-13.4090598563
N	10.7735383737	-2.8799456571	-12.3535374977
O	10.5754257068	-1.8307940712	-11.7186751253
O	9.9913100243	-3.2155633135	-13.2582460192
O	11.7538801792	-3.5934801611	-12.0836911312
N	8.8968315658	-6.8826504817	-15.2321993602
O	10.0373987228	-6.4926356396	-14.9321778703
O	8.5975951156	-8.0817682381	-15.1073311739
O	8.0555001818	-6.0735469159	-15.6570893784
N	9.4261382420	-13.3221952498	-13.0159807668
O	10.5483082388	-12.7900428036	-12.9921861631
O	9.2240959829	-14.3740618786	-12.3868755040
O	8.5060097635	-12.8024806487	-13.6688811589
N	6.9463408517	-9.7780376990	-7.3633688902
O	6.9242555616	-8.7082546879	-7.9943161623
O	5.9466638718	-10.5153334090	-7.3556953778
O	7.9681039442	-10.1105252677	-6.7400946289
N	3.7492828024	-9.4559539861	-3.5662499775
O	2.8082203289	-8.8999306868	-4.1563833713
O	4.0245329183	-10.6414762283	-3.8148305434
O	4.4150956960	-8.8264545363	-2.7275353425
N	10.7734406726	-9.8352663498	-8.3349988641
O	10.3058410385	-9.1520250169	-9.2610386980
O	11.6477015663	-9.3591434948	-7.5920366661
O	10.3667790857	-10.9946314711	-8.1519210808
N	8.5417540657	-11.7946529445	-4.0779270252
O	8.2110427137	-11.1369269732	-3.0774049319
O	9.7239266562	-11.7930263931	-4.4593496667
O	7.6902921418	-12.4540059981	-4.6970269754
N	5.9435309760	-10.0350772973	-12.8825441369
O	5.3565192634	-9.6262237771	-13.8980607341
O	7.0131031015	-9.5108888446	-12.5300390372
O	5.4609701746	-10.9681200213	-12.2195321057
N	14.1513430607	-4.9859451357	-7.3527979088
O	14.3813430422	-4.2141521444	-6.4070423917
O	14.4306241875	-6.1921337622	-7.2521551719
O	13.6420615425	-4.5515491507	-8.3991970052
N	11.7072289477	-13.8291518298	-8.0095855609
O	11.9239423098	-14.0046287452	-9.2200652517
O	10.7234587115	-14.3630864852	-7.4709504180
O	12.4742864392	-13.1197396880	-7.3377404722
N	10.6555474286	-5.1671072492	-5.9308610357
O	9.7685088016	-5.3713560346	-5.0856020111
O	10.7645350609	-4.0479980554	-6.4588138928
O	11.4335990498	-6.0819683940	-6.2481674587
N	8.3608532388	-11.7306418099	-10.3106067231
O	8.9112090488	-10.9376368313	-11.0924439807
O	7.2020719774	-11.5135422252	-9.9193118606
O	8.9692791801	-12.7407471863	-9.9200640135
N	2.4378927862	-7.0702551440	-14.6867161277
O	2.2527260783	-8.1833806792	-15.2060260005
O	3.4083970865	-6.3768043729	-15.0335090660
O	1.6525545616	-6.6505800420	-13.8206126193
N	-0.6234351302	-8.2073867412	-9.0181369476
O	-0.8528288343	-7.0018732381	-8.8254360564
O	-0.6857296263	-8.6755762046	-10.1670215950
O	-0.3317466952	-8.9447113743	-8.0619524216
N	5.5476791287	-2.1168231525	-7.8264732534
O	6.1510681553	-2.3706110661	-6.7707596140
O	4.4124329743	-1.6141096871	-7.7875727205
O	6.0795366847	-2.3657489046	-8.9210883070
N	1.0090117575	-4.8126758638	-5.1285953322
O	1.8162631611	-5.0565197842	-4.2165080420
O	1.0756054939	-3.7367103803	-5.7457512797

O	0.1351659141	-5.6447980967	-5.4235269121
N	10.2449582001	-1.2466480549	-7.3910960274
O	9.3175155816	-1.3675744527	-8.2085585076
O	10.0019912499	-1.2379731516	-6.1729376969
O	11.4153687111	-1.1343964700	-7.7917922002
N	6.2556746163	-4.2531105833	-3.1167543612
O	7.3474361336	-3.6606070605	-3.1129760283
O	5.2855114366	-3.7637442551	-3.7186861784
O	6.1340761809	-5.3349813053	-2.5186003954
N	13.4260929217	-9.6163088822	-2.8629565508
O	14.1773556932	-8.9748593576	-3.6160599080
O	12.9005580968	-10.6695447709	-3.2598510222
O	13.2003647933	-9.2045221864	-1.7129577966
N	2.6148087640	-8.9681928107	-10.4791518747
O	1.8996073499	-9.2312200378	-11.4601317552
O	3.4150672254	-9.8152885732	-10.0489971641
O	2.5297516483	-7.8580689274	-9.9283262613
N	0.6410604774	-3.5684535805	-10.9276884003
O	1.4382373755	-3.6551731128	-11.8763751909
O	-0.3134454189	-4.3590069059	-10.8442507303
O	0.7983896020	-2.6911800166	-10.0624385831
N	3.0053026514	-5.3107952283	-7.8562539881
O	4.0930924509	-4.7433996628	-7.6618483931
O	2.2509292905	-4.9145902243	-8.7601125711
O	2.6718859445	-6.2743965735	-7.1468004291
N	2.5283486497	-11.2462403541	-7.2804764357
O	2.9829358356	-10.1596293019	-6.8859579664
O	1.3347200712	-11.3364029946	-7.6123490407
O	3.2673906371	-12.2426895679	-7.3431223505
N	4.9103453318	-6.2696312803	-10.9130568193
O	5.6276116716	-6.8450070461	-10.0778983692
O	4.6151458961	-6.8400526910	-11.9763040241
O	4.4882780877	-5.1238331815	-10.6849678811
N	6.4706760685	-6.5213221514	-5.5969902355
O	7.1710815735	-7.2231657494	-4.8487485611
O	6.9813139269	-5.5742534050	-6.2177379122
O	5.2596317301	-6.7665474972	-5.7244843360
N	5.6676876551	-2.2058988201	-12.9810172904
O	5.5551518356	-2.9786565785	-13.9470395713
O	6.7152047776	-1.5571550049	-12.8233554651
O	4.7327055993	-2.0818847771	-12.1726561842
N	9.5333407105	-7.8179244117	-0.4403649816
O	10.2792485875	-6.9005043874	-0.0596027872
O	8.4545886424	-7.5513688268	-0.9955766589
O	9.8661851695	-9.0019009742	-0.2659153583
N	10.7733966324	-8.1366863072	-3.8376579260
O	11.3499337061	-8.5223880001	-4.8681224414
O	9.6872636713	-8.6411948517	-3.5078030636
O	11.2829929299	-7.2464753531	-3.1370477090
N	12.4577206870	-4.2129175818	-2.5582511289
O	12.5030666352	-3.1497185500	-3.1990080834
O	13.3378399453	-5.0742473147	-2.7210979689
O	11.5322547354	-4.4147870432	-1.7546466873
N	12.3770197505	-8.9361611107	-11.6340446190
O	12.6827133228	-7.7322400143	-11.6460172679
O	11.5493535068	-9.3749476102	-12.4497975889
O	12.8989928421	-9.7012963236	-10.8063183338
N	9.8307380031	-5.5539265375	-9.1391824480
O	10.9096163456	-5.8499620337	-9.6789994707
O	9.1450275649	-6.4362065530	-8.5965950541
O	9.4375697823	-4.3756100772	-9.1419528215
N	6.7643848701	-14.4353604057	-8.8574129361
O	6.3124491029	-13.6777758946	-7.9828629479
O	6.2876411783	-14.4142163288	-10.0042722524
O	7.6930650766	-15.2140896204	-8.5851033888

n=35 OPLS/AA

N	-0.5051740974	-7.4436140403	-4.1942760674
H	-0.4097585186	-8.3305801077	-4.7026294486
H	-1.3281517799	-6.9370952318	-4.5412358867
H	0.3353549073	-6.8725971474	-4.3415385012
H	-0.6181409055	-7.6341845381	-3.1917009282
N	-1.8563528059	-6.5061074053	5.1311049503
H	-2.1425560485	-6.3089836284	4.1649444988
H	-2.1522918833	-5.7341531518	5.7399903078
H	-0.8349699341	-6.6024970796	5.1725969238
H	-2.2955936366	-7.3787955694	5.4468871300
N	2.6943139470	-5.6851313782	5.2119586884
H	2.3535664389	-4.8935959107	4.6537483005
H	1.9057018209	-6.2946197500	5.4586356524
H	3.1367186140	-5.3339596934	6.0693921159
H	3.3812685823	-6.2183493876	4.6660581412
N	-0.0431192431	-10.6019202123	-1.1395346606
H	-0.9470122583	-11.0716125110	-1.0106833542
H	-0.1901921737	-9.5876191954	-1.2011600259
H	0.3965425369	-10.9371914842	-2.0047087573
H	0.5681840425	-10.8112581161	-0.3415863796

N	-0.5121872206	-2.6681679955	6.1243225125
H	-0.5982437342	-1.7248167649	6.5204523268
H	-1.2513945084	-3.2690057635	6.5074690220
H	0.4060550278	-3.0585958908	6.3664645282
H	-0.6051657516	-2.6202526440	5.1029045589
N	3.7313278480	-10.4403949493	4.4736112237
H	3.8536164931	-11.1085865197	5.2435473315
H	4.6004498872	-9.9105496336	4.3389802170
H	2.9674141993	-9.7929221265	4.7004380413
H	3.5038309316	-10.9495221681	3.6114800549
N	-5.9802682915	-5.8873306806	4.9649094900
H	-5.4441904531	-5.1719128026	5.4699183104
H	-5.3983527444	-6.7233355849	4.8356625114
H	-6.2611571340	-5.5207285852	4.0478817446
H	-6.8173723123	-6.1333450530	5.5061758856
N	-3.3723055149	0.7194963031	0.8675499180
H	-2.8117041091	0.0170812121	1.3641133091
H	-2.8788315521	1.6198521755	0.8763497299
H	-4.2816514890	0.8230458090	1.3329561096
H	-3.5170343633	0.4180053318	-0.1032189931
N	3.0629208887	-1.8681637848	1.9927631043
H	2.0586961891	-1.6734646210	1.9041003390
H	3.5169420010	-1.0999882453	2.5007058722
H	3.4788830685	-1.9533172547	1.0579048928
H	3.1971613182	-2.7458848287	2.5083412267
N	0.2118202024	-7.0023592410	1.5366279950
H	-0.0256375725	-6.3774413306	2.3159401969
H	0.3725257086	-6.4461577737	0.6886608061
H	-0.5623584219	-7.6571195910	1.3748507846
H	1.0627508643	-7.5287176602	1.7670609515
N	2.9278362719	-5.0386279750	0.2325230923
H	3.4434211414	-4.9139344458	1.1116475794
H	3.4449157821	-4.5918907129	-0.5338249253
H	1.9996483421	-4.6076640116	0.3160261068
H	2.8233603240	-6.0410226082	0.0362444647
N	8.6849438942	-3.3660889632	1.9390803807
H	9.1756568322	-2.5279669521	1.6059311581
H	9.3001026859	-3.8955777615	2.5679332720
H	8.4277714108	-3.9535412014	1.1372090034
H	7.8362451258	-3.0872691218	2.4452477647
N	2.8717204569	-9.4857373986	-4.4052235500
H	2.2720060698	-10.2050753520	-4.8260936728
H	3.0610991399	-8.7506099601	-5.0965686512
H	3.7573065441	-9.9105636594	-4.1060656414
H	2.3964694898	-9.0767013235	-3.5921666445
N	6.7245004812	-0.7963122901	-0.5057024253
H	6.2531133923	-0.2517084233	-1.2374379912
H	7.4479865339	-0.2187158634	-0.0616472657
H	7.1589114812	-1.6273840934	-0.9238438665
H	6.0379900581	-1.0874402498	0.2001187094
N	3.0534374245	0.2296236501	5.8719766148
H	3.8446724145	0.7679499242	6.2439821401
H	3.0632221521	-0.7152028122	6.2737749481
H	3.1318242460	0.1677955407	4.8500821412
H	2.1740316560	0.6979524720	6.1200675922
N	-4.9162008063	-2.8500063018	2.4280366788
H	-4.6049921673	-2.4908192047	1.5178883887
H	-4.2010891821	-3.4800369055	2.8100203025
H	-5.7972908036	-3.3638206032	2.3100926062
H	-5.0614307689	-2.0653481438	3.0741445313
N	3.0589650007	0.1496062487	-3.1617449147
H	2.7612982889	0.1716170921	-4.1441634350
H	3.8506117869	-0.4960286613	-3.0583736696
H	2.2789041297	-0.1686074551	-2.5748202173
H	3.3450455073	1.0914440407	-2.8696232936
N	6.1842972063	1.5710189048	2.5405839496
H	5.3484105427	2.1588219955	2.4404984369
H	6.5454746962	1.6498943642	3.4984796476
H	6.9032206563	1.8825380904	1.8770024595
H	5.9400821158	0.5928217418	2.3463551572
N	-1.1171361505	0.5046634165	-2.1228916119
H	-1.2494715962	1.4468175184	-1.7367969659
H	-2.0236824224	0.0252947206	-2.1740208165
H	-0.4822178413	-0.0276557703	-1.5164672158
H	-0.7131728707	0.5741981150	-3.0642810735
N	-3.6381699042	-9.9718326583	-3.0225171817
H	-3.6786956132	-8.9918515951	-3.3262125558
H	-4.3633972823	-10.5113461122	-3.5095543144
H	-3.7960618642	-10.0229442419	-2.0092584491
H	-2.7145248968	-10.3611877296	-3.2450437032
N	-3.5473529440	0.0771889514	6.2656587979
H	-3.8821578863	0.3113625776	5.3236904009
H	-4.2150217400	0.4268183267	6.9629480952
H	-2.6306078501	0.5126653368	6.4211395835
H	-3.4616246256	-0.9420902076	6.3548561945
N	-1.2263525353	-3.0655357122	0.8067499560
H	-1.0530592676	-2.8027969602	-0.1705800237
H	-1.9092210614	-3.8316079522	0.8392858532

H	-1.5958539282	-2.2557127637	1.3185028620
H	-0.3472757153	-3.3720249167	1.2397901806
N	-4.3650063810	-5.1661024431	-1.2103675383
H	-5.1544195253	-5.2268222740	-0.5566274620
H	-4.6700411714	-5.4652091996	-2.1440289385
H	-3.6050382657	-5.7760494616	-0.8868731450
H	-4.0305273303	-4.1963288964	-1.2539399710
N	5.2750476805	-6.1099060701	-3.4397042085
H	5.0718176816	-5.6280358149	-4.3232961518
H	4.4140484981	-6.1861351882	-2.8855343654
H	5.9755854123	-5.5760562292	-2.9119857629
H	5.6387389320	-7.0493965787	-3.6380014144
N	7.1510994867	-6.6620453384	4.5412441166
H	7.1809647026	-7.4216930434	3.8511140341
H	7.9903701737	-6.0785276296	4.4444635249
H	7.1182036420	-7.0600770000	5.4871427284
H	6.3148594575	-6.0878844205	4.3822555068
N	-1.3920312323	-10.7358961953	3.2976429619
H	-1.0190247011	-9.7911682725	3.4479472441
H	-0.6145685524	-11.3957877278	3.1779365204
H	-1.9789943161	-10.7420898188	2.4552229352
H	-1.9555369962	-11.0145380418	4.1094652944
N	-0.7081963829	1.8120807926	3.6875750042
H	0.3129256514	1.8714640383	3.5980232376
H	-0.9912467231	0.8260331723	3.7303217094
H	-1.1495697016	2.2599761410	2.8759008857
H	-1.0048937637	2.2908498767	4.5460540968
N	5.9446069290	-2.7620183111	6.1879236013
H	5.1986758412	-3.2127816589	6.7307268016
H	6.2452853753	-1.9044638361	6.6658644760
H	5.5929061642	-2.5270534776	5.2523309426
H	6.7415596087	-3.4037747109	6.1027727138
N	-4.3428950502	-8.5011073226	1.5877474436
H	-4.3980128266	-9.0821708275	0.7430206643
H	-5.1875626624	-7.9218953752	1.6604442825
H	-3.5159489861	-7.8949857479	1.5328624020
H	-4.2700557796	-9.1053779059	2.4146616030
N	-2.0055763397	-3.5257774350	-3.6952927616
H	-2.3658533459	-4.2317904349	-4.3479658960
H	-1.3186665032	-3.9592182127	-3.0671855620
H	-2.7815339843	-3.1433274470	-3.1422491244
H	-1.5562518762	-2.7687743328	-4.2237710998
N	2.1748855046	-3.8218429205	-3.6381258362
H	2.8636443480	-3.6770706426	-2.8905374864
H	1.3527847743	-4.3048657878	-3.2572400469
H	2.5926398686	-4.3923697711	-4.3825826369
H	1.8904736981	-2.9130653396	-4.0221424464
N	2.2652607266	2.9046414594	0.4229200287
H	2.8301273954	2.9352636534	-0.4339487077
H	1.4828514743	3.5644380809	0.3408294348
H	2.8487670211	3.1640141839	1.2269608972
H	1.8992975658	1.9548500492	0.5578376561
N	3.7839124676	-9.8053673450	0.3684293332
H	4.1253520894	-10.0082864389	-0.5783963760
H	3.3558844087	-8.8722306232	0.3852378054
H	4.5695376236	-9.8334915909	1.0289122361
H	3.0848760812	-10.5074609245	0.6379627451
N	9.2397804826	-0.4999496328	5.1125868098
H	10.0044835581	-0.1117564116	5.6771851865
H	9.2285635580	-0.0446824560	4.1923473498
H	8.3449254083	-0.3334735759	5.5877243084
H	9.3811501509	-1.5098857095	4.9930909445
N	6.6892152259	-7.1170242156	0.5318486816
H	6.4844266934	-7.6347027951	-0.3308833990
H	7.2552365320	-6.2899848980	0.3085539581
H	7.2079142877	-7.7221544911	1.1791539860
H	5.8092831910	-6.8212551825	0.9705693412
N	-3.7140620433	-8.9234558002	4.6615838966
O	-2.6983499035	-9.0286993934	5.3688819569
O	-3.9787143979	-9.7985510908	3.8206408103
O	-4.4651224332	-7.9431161272	4.7952290303
N	1.7298318977	-11.5555444895	1.9851182714
O	1.4630567350	-11.0945988444	0.8628981344
O	2.9108903634	-11.6234810745	2.3639367394
O	0.8155478585	-11.9485538660	2.7285205388
N	-1.9715643498	-8.0819813774	-0.7466694433
O	-1.5450382574	-8.4990087774	0.3429091684
O	-3.0634090284	-7.4921707075	-0.8015919568
O	-1.3062452280	-8.2547647865	-1.7813263745
N	-3.0735194492	-5.5286797682	2.3781363147
O	-3.0313680294	-4.8952138095	1.3104462624
O	-2.5118992266	-6.6330259539	2.4676915417
O	-3.6772915778	-5.0577991621	3.3562719276
N	4.7357310771	1.7438848046	-1.2022376545
O	5.4287470883	0.9899349114	-1.9053068385
O	3.7259111131	2.2858034216	-1.6814096110
O	5.0525352848	1.9559162516	-0.0199955621
N	-3.1589888897	-10.9134395037	-0.0357835950
O	-2.8828259907	-10.9796997232	1.1734978228
O	-4.1489795674	-10.2643607128	-0.4121253175
O	-2.4451605361	-11.4962585442	-0.8687239610
N	6.3644933619	-9.2454843628	3.0174699194
O	5.6736567736	-9.8180041156	2.1584100542
O	7.2944235007	-8.4987772408	2.6700849431
O	6.1253996188	-9.4196718724	4.2239157321
N	2.3561243303	-7.5750870526	-1.6768491604
O	2.3629813104	-7.6339966661	-0.4360830462
O	3.0151997280	-6.6988643457	-2.2606910046
O	1.6901914164	-8.3924008041	-2.3337739593
N	-1.1636946325	3.0336904976	0.3739912505
O	-1.6947656803	2.7277635256	1.4544495518
O	-0.1687949356	3.7773185356	0.3595052200
O	-1.6275236550	2.5959890791	-0.6919818785
N	-0.1028081771	-10.5556458785	-4.1689549559
O	0.1720116514	-9.8126519335	-5.1257474990
O	0.8028317550	-11.1634188921	-3.5744415514
O	-1.2832688881	-10.6908669187	-3.8066755258
N	-1.8829967284	-1.5212690741	3.4316375837
O	-1.5545760736	-0.6000392716	4.1974661778
O	-1.6012036224	-2.6968174859	3.7174635585
O	-2.4932109805	-1.2669502600	2.3799821683
N	5.1800572490	-4.6887001847	3.0143517897
O	5.2505078488	-4.8577320495	4.2429619201
O	6.1844138923	-4.8638802444	2.3047056873
O	4.1052491406	-4.3444879832	2.4953873439
N	5.9839930321	-1.5749432480	3.2296810832
O	5.0140112961	-1.6708832963	3.9997128032
O	5.8512362785	-1.0112200599	2.1307681101
O	7.0867324096	-2.0427267644	3.5585626010
N	2.9355780424	-3.2686970612	6.9921460921
O	3.2372947714	-2.0638604086	6.9733427145
O	3.8266223028	-4.1310255513	7.0659540337
O	1.7428160929	-3.6112054995	6.9371414837
N	8.9178092292	-4.0881178194	4.9631296936
O	9.5753401644	-3.0349401820	4.9245571110
O	8.0633338602	-4.2547447346	5.8492026171
O	9.1147538216	-4.9746692554	4.1156286704
N	0.8973065284	-4.3300174526	3.0858274533
O	1.5877358262	-3.6786218346	3.8870821222
O	0.1485122376	-5.2303861944	3.5001475590
O	0.9556715683	-4.0810441285	1.8702517002
N	0.7272508898	-8.3179651964	4.8110702472
O	0.6521011694	-7.2581975587	5.4547028943
O	1.6229196983	-9.1391022398	5.0690028979
O	-0.0932688590	-8.5565959829	3.9095042235
N	3.6181836845	-7.5209822932	2.5734425717
O	2.4976539700	-8.0131358307	2.3608021117
O	4.3221682885	-7.1449310354	1.6215975004
O	4.0347291304	-7.4048799202	3.7379290404
N	6.4470421209	0.6701748703	5.9821816931
O	6.9842905727	-0.3306933183	6.4848196155
O	5.3896286730	1.1134321943	6.4601122904
O	6.9672075357	1.2277861838	5.0016123841
N	-4.9735842279	0.1795173898	3.4512020951
O	-5.1988973690	-0.8575426630	4.0967703302
O	-4.2454015250	1.0640409328	3.9311874740
O	-5.4764541946	0.3320540225	2.3256475750
N	0.3939223656	-0.9539391095	-4.5533624952
O	-0.6782825513	-1.5640506524	-4.6988218787
O	0.3869292783	0.2586269587	-4.2838211476
O	1.4731212386	-1.5563941197	-4.6774445591
N	5.2425760099	-9.0801649355	-2.3921335668
O	4.4787168968	-10.0280042128	-2.1448728325
O	5.4570644224	-8.7461041917	-3.5691705463
O	5.7919471528	-8.4663859079	-1.4623565730
N	-0.4900440684	0.8231524209	6.5950293779
O	-0.7704227696	-0.2763051346	7.1005971563
O	-1.3939659557	1.6313514962	6.3252936052
O	0.6942574735	1.1144111355	6.3591971825
N	-3.5277011580	-6.4056723125	-4.1949224612
O	-2.5555998611	-5.8816480276	-4.7636363859
O	-3.5855704546	-7.6407219965	-4.0752506865
O	-4.4419338944	-5.6946463411	-3.7458799497
N	2.8394803862	-6.3128829713	-5.3825010644
O	3.3210992673	-7.3817523578	-5.7930852809
O	1.6426802039	-6.2562815057	-5.0546542880
O	3.5546622631	-5.3006142356	-5.2997635576
N	0.3672167697	-0.2084604778	1.0808931561
O	0.5876208561	-1.1129494687	1.9032918520
O	1.2574694767	0.6139610283	0.8086967289
O	-0.7434409177	-0.1263929270	0.5306904444
N	0.7416664502	-2.0172530540	-1.3739242458
O	1.4464766222	-2.8881901617	-0.8375203992
O	1.2429834530	-0.9303935197	-1.7062426570
O	-0.4644616957	-2.2331756542	-1.5780098453
N	4.3424573810	-2.6901628361	-1.4671907317

O	4.0736321775	-3.8591595328	-1.7899873304
O	4.5559348371	-1.8370345033	-2.3444690994
O	4.3978051728	-2.3742942180	-0.2671147993
N	-3.7340429603	-1.7979172562	-1.1036279200
O	-4.0599480326	-2.0557088052	0.0669904260
O	-3.4120343785	-0.6401392278	-1.4180773135
O	-3.7301464667	-2.6979044602	-1.9597975618
N	7.5717121119	-4.3824388283	-0.9613296681
O	7.6743566331	-3.1468499047	-1.0375006929
O	8.0897528429	-4.9912676459	-0.0105514695
O	6.9510263601	-5.0091994390	-1.8359375459
N	-3.4950188904	-3.6894228978	6.4805943101
O	-2.4943014873	-4.4224824124	6.5454505992
O	-4.6159095726	-4.1850380193	6.2781402640
O	-3.3748455145	-2.4607472726	6.6181921777
N	8.9649680943	0.1428249588	1.7015156597
O	9.4248345744	-0.9609856448	1.3651484753
O	8.2378083449	0.7771908520	0.9193158620
O	9.2322615786	0.6122700471	2.8200835422
N	-6.3777052105	-5.6377689089	1.7892190182
O	-6.6979204835	-4.8294441693	2.6764006260
O	-6.1621995184	-5.2379104118	0.6330666105
O	-6.2729955451	-6.8459531183	2.0581900347
N	-0.3952453085	-5.1911363774	-1.5435820412
O	0.0758769597	-5.0375008207	-2.6826421077
O	-1.5914556664	-4.9361294956	-1.3266254076
O	0.3298433649	-5.5997791448	-0.6214778660
N	3.0106695694	1.7056831817	3.1135185014
O	3.4489518143	0.5546759856	3.2751013566
O	3.7369309450	2.5828807305	2.6174471500
O	1.8461250115	1.9794930496	3.4480072670

n=36 OPLS/AA

N	-9.0710493707	1.4254760727	2.3674567026
H	-9.4403144388	2.0803518027	1.6681590655
H	-9.8507286474	0.9458417230	2.8325220759
H	-8.5225955558	1.9425794734	3.0646209659
H	-8.4705592002	0.7331319295	1.9045240221
N	-6.0522799922	-1.3628284830	14.1744296833
H	-7.0189244846	-1.1019522567	14.4019777387
H	-6.0143582927	-2.3605777393	13.9350700132
H	-5.4490925579	-1.1822686709	14.9854775224
H	-5.7267455752	-0.8065150109	13.3751936804
N	-6.6817470241	9.3119537221	7.4620081431
H	-7.2558171181	10.0820082095	7.0991091059
H	-5.7512655076	9.3453055891	7.0291958444
H	-7.1308587133	8.4158720125	7.2393609236
H	-6.5890473164	9.4046298274	8.4803663452
N	-6.9281848483	0.6499744804	6.0495447494
H	-6.2504063240	1.0659494844	5.4000702191
H	-7.7089858186	0.2474525653	5.5179804182
H	-7.2836784928	1.3748553511	6.6839007347
H	-6.4696680977	-0.0883590743	6.5962269932
N	-1.5912969660	-2.4834019520	5.9786339375
H	-1.2746712031	-2.7478711388	5.0383997788
H	-2.4850008571	-1.9825120226	5.9105360730
H	-0.8903389168	-1.8746786275	6.4172103293
H	-1.7151765787	-3.3285462768	6.5483886531
N	-2.2010179248	2.8057040504	13.7224901304
H	-2.7186450775	2.1807345511	13.0934342081
H	-1.9192678098	2.2882475445	14.5633767593
H	-2.8008916094	3.5945527610	13.9910134968
H	-1.3652677066	3.1592807363	13.2421354447
N	-9.8097577105	6.3991398684	8.7627086781
H	-9.2148643665	6.7046186315	7.9835933258
H	-10.6682253650	5.9750669328	8.3919929960
H	-10.0521669979	7.2085905344	9.3460292424
H	-9.3037735333	5.7082836724	9.3292183893
N	2.1404463509	1.8160360231	10.2634736587
H	3.0007686166	2.1008065778	9.7807859462
H	2.3642056757	1.5377312116	11.2261329349
H	1.7163232724	1.0231580571	9.7677926429
H	1.4804886768	2.6024485232	10.2791826410
N	-6.3470149729	-3.2498191932	8.3952122805
H	-7.0684084607	-3.7337237355	7.8477965999
H	-5.4384928374	-3.3485335473	7.9271549219
H	-6.2951196682	-3.6627888800	9.3338274515
H	-6.5860396280	-2.2542310814	8.4720696156
N	-9.8353924269	-2.5487234824	11.3660138114
H	-9.6562214781	-1.6792849606	11.8819697450
H	-9.5336296763	-2.4365055414	10.3910363442
H	-10.8396773174	-2.7608873007	11.3911911223
H	-9.3120410613	-3.3182152801	11.7998585364
N	-2.3974891167	8.0154142911	7.0682691881
H	-3.0844097275	8.5951501447	6.5720005995
H	-1.5315982627	8.5505150195	7.2029351636
H	-2.1971428518	7.1735751398	6.5156357277
H	-2.7768062937	7.7424174249	7.9825047783
N	-11.8424449207	0.9185381026	6.4527748762
H	-12.4715984632	1.7285044173	6.5013185330
H	-12.1095710144	0.2341930633	7.1700964237
H	-10.8762326194	1.2254463621	6.6154912958
H	-11.9123781984	0.4860093567	5.5241932994
N	-0.8979204252	-0.8435623820	15.6080079113
H	-0.3552402855	-0.3181067088	14.9125722931
H	-1.7304463277	-0.3035517392	15.8716815976
H	-0.3193842268	-1.0086520374	16.4400396396
H	-1.1866103325	-1.7439385308	15.2077374376
N	-3.5079665530	-0.9249966423	2.8555474553
H	-2.7895140262	-0.2451698016	2.5800523899
H	-3.1023164550	-1.8682079686	2.8614700737
H	-4.2857933199	-0.8911530545	2.1861629199
H	-3.8542417111	-0.6954550824	3.7945041696
N	-4.1669061371	-5.8321963295	6.1010136106
H	-4.1368955247	-6.8263962174	6.3557724057
H	-3.6239700466	-5.2879406356	6.7816315847
H	-5.1410688307	-5.5077881275	6.1023879566
H	-3.7656901171	-5.7066613058	5.1642627434
N	-0.7890161762	-1.6990143675	10.6918359261
H	-0.1401457213	-1.0840974408	10.1867810742
H	-0.3217807948	-2.0814275611	11.5223092449
H	-1.0792444157	-2.4688163282	10.0775004848
H	-1.6148931412	-1.1617155411	10.9807524088
N	-1.6808768399	1.9077972321	10.0386199252
H	-1.0234143831	1.6076947982	10.7679468373
H	-2.3772913915	2.5428983617	10.4459134471
H	-2.1551810997	1.0860346442	9.6462205849
H	-1.1676198449	2.3945608322	9.2943995420
N	-4.2884158572	5.4096776990	5.0851275491
H	-5.2623795641	5.7188108998	5.1854642791
H	-4.2608715132	4.5328793402	4.5515540935
H	-3.8801275465	5.2575137702	6.0148491839
H	-3.7502857535	6.1295070869	4.5886427377
N	0.8741959927	6.1478312554	8.5151653490
H	0.5322807816	5.9766097095	9.4680626510
H	0.7857193464	7.1465988123	8.2940998932
H	0.3185820078	5.5991940883	7.8484376064
H	1.8602015020	5.8689222447	8.4500621733
N	3.0017469815	5.9498715034	5.2107554096
H	3.8954155291	6.2220156825	4.7846918342
H	3.1802237923	5.4339240545	6.0803409106
H	2.4798668957	5.3521680455	4.5591210976
H	2.4514825791	6.7913784961	5.4188673812
N	-6.8707843895	6.6477450093	12.0192517687
H	-6.1915837014	6.5783145959	11.2523737206
H	-7.5455659394	7.3934609345	11.8123404246
H	-6.3785272796	6.8646036513	12.8938321202
H	-7.3674599760	5.7546007879	12.1184600625
N	-10.6899144627	-3.3794759401	7.0228191035
H	-11.3121387689	-4.1061427507	6.6499540386
H	-11.0514753511	-3.0493882043	7.9253436163
H	-10.6473782717	-2.5938163638	6.3631485595
H	-9.7486660651	-3.7685571493	7.1528298366
N	1.0209052714	2.4932979561	5.6559076486
H	0.6049817762	2.0317327302	6.4733434479
H	0.4999335251	3.3528836356	5.4462994791
H	0.9782962959	1.8615597010	4.8476213278
H	2.0004090833	2.7270153081	5.8563671357
N	-3.8892229577	-3.3678358073	11.2389588897
H	-3.8436134280	-3.8889808431	10.3554637171
H	-4.7464993722	-3.6231613209	11.7430627647
H	-3.8952402424	-2.3601609500	11.0420053463
H	-3.0715387440	-3.5990406228	11.8153028703
N	-6.9726857559	-2.7776657769	3.5331920389
H	-7.6544650063	-3.5088588649	3.7672215656
H	-6.1202917065	-3.2114092712	3.1596607135
H	-6.7420079705	-2.2417401401	4.3780630322
H	-7.3739790044	-2.1486555434	2.8278230724
N	-1.5606537803	3.2559183471	2.3155170224
H	-1.6474353600	3.5302532438	3.3011363300
H	-1.5270502031	2.2320036018	2.2469307312
H	-0.6987284104	3.6536236518	1.9241723259
H	-2.3694012322	3.6077931582	1.7898296625
N	-2.6228600483	6.2548964884	11.3377238644
H	-2.8927552381	5.2913405595	11.1076094234
H	-2.9128345494	6.8821774117	10.5783371925
H	-1.6040098386	6.3064448460	11.4540103256
H	-3.0818408301	6.5396221981	12.2109382919
N	-10.2977387830	4.3881855049	4.8974190198
H	-10.3652901473	4.2291924093	3.8852954571
H	-9.8344690042	5.2876264575	5.0724097916
H	-9.7512294826	3.6324473785	5.3268640022
H	-11.2399665638	4.4034756194	5.3051058428
N	-6.5122481562	1.0319044398	10.1678422584
H	-6.7928984416	0.3665841736	9.4378967411

H	-5.8472452505	1.7091676588	9.7762808289
H	-6.0677180841	0.5238173316	10.9414545834
H	-7.3411311219	1.5280479474	10.5157361692
N	-6.9324911235	7.0095774399	2.8679538954
H	-6.9314220184	8.0363215518	2.8735649057
H	-6.9048126735	6.6620126535	3.8337009162
H	-6.1088709926	6.6692484144	2.3579915846
H	-7.7848588085	6.6707281400	2.4065581807
N	-6.2370139702	2.9710796849	13.7275423839
H	-5.5651081919	2.4007474331	14.2543249247
H	-5.8276786977	3.2344439158	12.8234856368
H	-6.4597553196	3.8187873487	14.2623447138
H	-7.0955130173	2.4303394864	13.5700147734
N	-10.4686551507	1.4407856793	9.7148186282
H	-11.0282165327	0.7873154312	10.2752682623
H	-9.9955466491	0.9275986546	8.9617966439
H	-9.7677930227	1.8923591172	10.3140758615
H	-11.0830649434	2.1558688776	9.3081342910
N	-1.0712640164	7.6441377667	3.1592577686
H	-1.8664024975	6.9945640078	3.1525626088
H	-0.9883578796	8.0740687776	4.0879776316
H	-1.2215532493	8.3763492339	2.4553294784
H	-0.2087432134	7.1315684149	2.9411613492
N	-6.2798364662	4.6235901726	8.0699580920
H	-7.1397899647	4.6196200882	8.6309396571
H	-5.9237149812	5.5837873124	7.9962449574
H	-6.4820035543	4.2605135562	7.1310548152
H	-5.5738382022	4.0304397300	8.5215934845
N	-6.0029294935	3.3491882776	1.1164265415
H	-6.8193262140	3.6803092796	0.5890828448
H	-5.1473343473	3.7363353517	0.7013256208
H	-5.9662614268	2.3236576460	1.0821059844
H	-6.0787967810	3.6564511556	2.0931912023
N	-2.8720459681	1.6197605642	6.4617419300
H	-2.2156701136	0.9629294659	6.0235869991
H	-2.3544398598	2.2693932446	7.0653112986
H	-3.5533377476	1.0979206631	7.0254441145
H	-3.3647355122	2.1487982435	5.7326248810
N	-8.2904558124	8.7529288900	10.0161885947
O	-8.9995884375	9.0763362267	9.0489468847
O	-7.1022790277	9.1122019992	10.0628434142
O	-8.7695003577	8.0702478944	10.9367762263
N	-11.9915303014	-1.4070204622	9.3023220612
O	-12.3675566992	-0.8274913233	8.2699616414
O	-11.8128600829	-0.7602143379	10.3476625327
O	-11.7941739632	-2.6333567127	9.2893419990
N	-4.6422988173	7.0832258261	9.1105658866
O	-3.6104553885	7.7245146018	9.3695364553
O	-5.0728290512	7.0431513049	7.9460678568
O	-5.2436124963	6.4820110875	10.0160940767
N	-6.8906544961	-3.9629450040	11.9910445280
O	-6.0511281635	-3.6119644897	12.8366367030
O	-8.0707183121	-4.1530578679	12.3291634421
O	-6.5501167385	-4.1238127839	10.8073324859
N	-4.6144106905	5.6857423233	13.9066067180
O	-4.2875842292	6.7131932078	13.2897217572
O	-3.7750983311	4.7947096672	14.1178730908
O	-5.7805504499	5.5493239849	14.3122256327
N	-8.6073387969	-1.0416731782	8.4616419247
O	-9.4377718931	-0.2375747534	8.0068623001
O	-8.9871172959	-2.1250959999	8.9359637481
O	-7.3971262276	-0.7623485565	8.4420997101
N	-7.8127374321	6.6575315218	5.8705809014
O	-6.6716003215	6.4253054152	5.4382673103
O	-8.8017296398	6.4791883879	5.1404397845
O	-7.9648824575	7.0681010929	7.0330365453
N	-8.8010876369	2.8830752790	7.2548754079
O	-7.9018243292	2.2914589694	7.8748218222
O	-8.9125382534	4.1166426993	7.3492580887
O	-9.5889009623	2.2411236514	6.5405457377
N	-8.2718893333	3.9816179382	10.9881989916
O	-8.1860904812	4.7844293367	10.0441914036
O	-8.4693780056	2.7762008202	10.7623988434
O	-8.1601994231	4.3842239818	12.1580076697
N	-2.3723748155	4.5875214144	7.9832928371
O	-1.7549581168	3.5106250612	7.9373714302
O	-3.4828100246	4.6906254174	7.4361903588
O	-1.8793559081	5.5613145485	8.5763171996
N	-0.8368885036	5.0873015625	5.4740027103
O	-1.1270772921	4.0819277337	4.8046509027
O	0.2137199320	5.1104691964	6.1363493617
O	-1.5973087629	6.0695085480	5.4810078721
N	-3.9989066203	-0.4036460399	8.7385446128
O	-2.8015335429	-0.1473089510	8.9473673160
O	-4.5427542371	-1.3526551349	9.3272970381
O	-4.6524326070	0.2890265239	7.9409688424
N	-9.9975272356	-0.6001221958	4.6546069917
O	-10.5559795967	-1.4947797916	5.3109122998
O	-8.7574258904	-0.5486746960	4.6044101865
O	-10.6791767685	0.2430885791	4.0484980007
N	-4.7550001934	-2.0401426470	5.9400798021
O	-5.9645996683	-1.8292405903	5.7519114098
O	-4.3906929373	-3.0469563865	6.5698608974
O	-3.9097072943	-1.2442303236	5.4984667436
N	-3.8247850920	0.6184379510	15.4911846761
O	-4.1090489867	-0.5631729663	15.7481040978
O	-4.6750330873	1.3642156868	14.9774611840
O	-2.6902722887	1.0542714834	15.7479889532
N	-3.3793213130	-4.0277046592	3.4916885336
O	-3.2033518863	-5.2090594203	3.8329415193
O	-4.5133757613	-3.6367566646	3.1690461854
O	-2.4212355201	-3.2372972562	3.4730778813
N	-7.7908396841	-4.6655846646	5.9861594579
O	-8.4238385230	-4.4828167481	4.9331037758
O	-6.6297023025	-5.1049575403	5.9445737850
O	-8.3189786519	-4.4089794990	7.0808016940
N	-6.9071303543	3.5522051702	4.4538597205
O	-6.4571052057	3.5904721940	5.6110247526
O	-7.8939520769	2.8437909778	4.1943406831
O	-6.3703333483	4.2223528783	3.5562130032
N	-1.2137123954	-3.0884113185	13.4464618116
O	-1.6631158307	-3.0846279372	14.6044946908
O	-0.2208918416	-2.3976673250	13.1632693076
O	-1.7571299513	-3.7829392525	12.5716207322
N	-8.3952564933	-0.0398344121	12.6420043531
O	-8.8541922905	-3.744638E-40	11.4883845035
O	-7.6830355693	0.8884508399	13.0591922691
O	-8.6485418241	-1.0075803915	13.3784368796
N	-9.2033809682	4.4880299258	1.6742227624
O	-8.5994613212	3.7720700187	0.8583181220
O	-8.8761899998	5.6776822681	1.8180674205
O	-10.1344923333	4.0143371094	2.3462832857
N	-6.6802017499	-0.3526882225	1.4384096215
O	-6.6790974940	0.8299969381	1.0585774184
O	-7.7356088138	-0.8786037425	1.8289819018
O	-5.6258980931	-1.0094583918	1.4276695357
N	-0.0191867854	4.5043506512	11.0990969365
O	0.5161062075	3.7979199639	10.2287895157
O	-0.0200758377	5.7409342806	10.9812918095
O	-0.5535911564	3.9741972822	12.0872102797
N	0.5822211447	0.9676780647	12.8676763522
O	1.8151164655	1.0338988349	12.7312877586
O	-0.1528250634	0.9405135779	11.8666833085
O	0.0843716312	0.9286217499	14.0050589051
N	-12.0419127513	3.9443862253	7.4984317979
O	-11.7961060070	5.1455025431	7.6982230549
O	-11.9388939687	3.1264215908	8.4275921167
O	-12.3907385589	3.5612342335	6.3694793132
N	-4.4800145441	3.4912079351	10.5864582890
O	-5.3564515591	3.7235177593	11.4355214288
O	-3.2999967682	3.8091937270	10.8088565707
O	-4.7835955494	2.9409118760	9.5149960050
N	0.1776042719	0.0728374731	8.0561869413
O	0.6673449939	-0.0854706267	9.1867224257
O	-0.0662543452	1.2174183781	7.6396711101
O	-0.0682780309	-0.9134361261	7.3421667132
N	-2.5742023016	-4.2523100492	8.4932146286
O	-3.6920484619	-4.6057611779	8.9037145341
O	-2.1697219942	-4.6373999039	7.3836564302
O	-1.8608358743	-3.5137684714	9.1922734844
N	0.5337698243	8.4162388710	6.2362409781
O	-0.2788800922	8.7823050112	5.3710163800
O	1.6581319856	8.0093186815	5.8997241131
O	0.2220573285	8.4570929532	7.4379834087
N	-4.3035201832	1.9820422289	3.6867332133
O	-5.0417335393	1.1779194223	4.2795615727
O	-4.1035303568	1.8586422212	2.4669814277
O	-3.7652962203	2.9095657899	4.3136571441
N	3.3700575757	3.9182139228	7.6630314616
O	3.1977681650	5.1362392611	7.8355091271
O	3.2837850847	3.4341310371	6.5223129476
O	3.6286196855	3.1842708793	8.6312730894
N	1.1440521752	5.1118063228	2.7694776199
O	0.5480031517	4.6146864516	1.7996243805
O	1.7375172065	4.3774453158	3.5766523711
O	1.1466361694	6.3432881925	2.9321562392
N	-3.8697358832	0.0603320144	11.9903732870
O	-5.0794143596	-0.2177892639	12.0387928019
O	-3.4967345407	1.2366715575	12.1321942614
O	-3.0330580759	-0.8378869735	11.8001326445
N	-3.7872593793	5.4758987410	1.7955268235
O	-4.8689787464	6.0757258687	1.6810342976
O	-2.9446418455	5.8666779895	2.6203329715
O	-3.5481573534	4.4852915671	1.0852126298
N	-4.6519984787	8.7762358526	4.9533480285
O	-3.7832708248	8.0246629886	4.4806235798

O	-5.7066659898	8.9767402056	4.3284541526
O	-4.4660584716	9.3273048073	6.0509672368
N	-0.8209555592	0.5609825523	3.7559902542
O	0.3896441085	0.8330722737	3.8145866627
O	-1.4257567078	0.6492938964	2.6745862649
O	-1.4267545660	0.2005811967	4.7787986584

n=37 OPLS/AA

N	-0.1140688594	-0.3588673736	2.5406379933
H	0.7901014343	0.0413999435	2.2640548186
H	-0.8717101061	0.2621202155	2.2330850045
H	-0.1462977495	-0.4581997137	3.5620732134
H	-0.2283681356	-1.2807895498	2.1033386672
N	-3.5547599397	-3.0051354606	-1.3828684997
H	-4.3394315117	-2.6953296412	-0.7975933714
H	-2.7507254677	-3.2312073593	-0.7856627145
H	-3.2967864161	-2.2548362071	-2.0345863953
H	-3.8320971273	-3.8391683330	-1.9136309477
N	-0.3855334096	-5.8985135171	0.9431750719
H	-0.9933112869	-6.5050168776	1.5061988930
H	-0.1100671839	-6.3912948682	0.0855506879
H	0.4533616340	-5.6577034394	1.4840088137
H	-0.8921173936	-5.0400394739	0.6969424411
N	-7.1318309576	5.7123505158	1.3735135791
H	-6.9920448641	6.6232193088	0.9207288758
H	-7.0907526260	5.8270358649	2.3930205809
H	-6.3952006861	5.0636826996	1.0721144560
H	-8.0493255182	5.3354650769	1.1081899626
N	0.8358805878	-3.9500839977	-2.0046374572
H	-0.0846935020	-3.6019649565	-2.2971995462
H	0.8903797801	-4.9607155256	-2.1775201871
H	0.9687688400	-3.7665129895	-1.0031995303
H	1.5690663366	-3.4711421802	-2.5406308503
N	-12.3915623396	-3.4516071973	-3.2069215771
H	-12.1399604707	-3.1227435351	-4.1464859915
H	-11.6389616506	-3.2167209593	-2.5491561604
H	-12.5250686617	-4.4694908566	-3.2249338577
H	-13.2622583304	-2.9974731181	-2.9071112140
N	0.6887242282	4.9435353899	0.3670099345
H	1.4128391085	4.3878485680	0.8372289193
H	-0.1217133568	4.3470910045	0.1628538636
H	1.0641282061	5.3228642480	-0.5101542433
H	0.3996436604	5.7163371981	0.9781116566
N	-7.4358933859	-1.6417196660	-1.9462660167
H	-8.1472780831	-1.0719265065	-2.4190277596
H	-6.9067940149	-1.0542944512	-1.2911170580
H	-6.7956520699	-2.0382055388	-2.6442098067
H	-7.8938500685	-2.4024516125	-1.4307099029
N	-3.9954414010	-6.8024871105	-3.8704685484
H	-4.5626576954	-6.9105043288	-4.7194880143
H	-3.8182866732	-5.8052920318	-3.7017887839
H	-3.1016777786	-7.2932383057	-3.9912384776
H	-4.4991440092	-7.2009138809	-3.0693597448
N	-2.8763084816	5.5089963837	2.1384023090
H	-2.4805353716	4.6518319860	2.5419725487
H	-3.1011651295	5.3494781573	1.1493481605
H	-2.1924015513	6.2709604918	2.2153278100
H	-3.7311314884	5.7637140647	2.6469611100
N	-5.8418360213	7.3551293916	-4.3492716671
H	-6.3081246520	7.9044596881	-3.6178024362
H	-6.5466816032	6.8536710163	-4.9024144055
H	-5.2031792906	6.6787837160	-3.9146343109
H	-5.3093589937	7.9836036811	-4.9622348035
N	-8.3627502861	-6.9080495438	-3.8749245705
H	-8.0719980698	-7.0565763327	-4.8483921043
H	-9.3830491461	-6.8001520251	-3.8351370459
H	-7.9143691555	-6.0597493022	-3.5094432633
H	-8.0815844897	-7.7157206598	-3.3067268167
N	-8.5542101768	-5.9897017236	0.2021107341
H	-8.9676380174	-5.9949267046	1.1419438139
H	-9.2603327097	-6.2809527564	-0.4840368374
H	-7.7605345904	-6.6404769863	0.1737180383
H	-8.2283357924	-5.0424504524	-0.0231811629
N	-9.2017243389	5.4259138820	-2.6773277379
H	-8.8084308578	4.5713878012	-2.2658164834
H	-8.9872444024	6.2272430206	-2.0722619119
H	-10.2198490152	5.3262403566	-2.7652075193
H	-8.7913726973	5.5787835172	-3.6060246362
N	-0.0114348453	-3.8502460126	4.5618685971
H	0.6591802411	-3.9296533367	3.7884308294
H	0.4108380810	-4.2224727973	5.4205696405
H	-0.8582171892	-4.3856132033	4.3369749396
H	-0.2575398608	-2.8632447906	4.7014982256
N	-11.5156688943	2.7822361731	0.1958308513
H	-10.8156797273	3.5049545122	0.4005977089
H	-11.7523952268	2.2804931919	1.0598038311
H	-11.1339820851	2.1207656777	-0.4904690012
H	-12.3606178563	3.2227320145	-0.1866089341
N	-6.5116669991	2.0016357471	-1.9465884432
H	-6.6537166652	1.5246483674	-2.8446647371
H	-7.3417794551	2.5625976110	-1.7219336345
H	-5.6910472326	2.6153641207	-2.0110570889
H	-6.3601247818	1.3039324247	-1.2086991871
N	-4.3902384950	-0.1926972728	1.0329291944
H	-4.5841489476	0.7761063084	0.7535469205
H	-4.4813928747	-0.2798154150	2.0519169244
H	-3.4355772866	-0.4453990332	0.7518610148
H	-5.0598350599	-0.8216800078	0.5743916460
N	0.3224439945	8.5420240675	-2.3931195512
H	-0.5070909853	8.7366692086	-2.9660253758
H	0.0498034476	8.4831406600	-1.4049725166
H	1.0073061709	9.2972709968	-2.5147805525
H	0.7397565369	7.6510155942	-2.6867003177
N	-13.5484863833	0.6028098805	-2.9002759261
H	-13.2795151419	1.5713944281	-2.6911478717
H	-14.5168744113	0.5824680980	-3.2409304793
H	-13.4734900757	0.0351575410	-2.0479955148
H	-12.9240656425	0.2222203984	-3.6210296348
N	-11.2708215977	-1.2853735592	0.7931438389
H	-10.2817758794	-1.1051211517	0.5844964466
H	-11.6360769157	-0.5363940045	1.3930212136
H	-11.3597051343	-2.1861371806	1.2778707099
H	-11.8057274982	-1.3138417247	-0.0828132179
N	-8.8711332962	-3.1520072749	-5.0887448508
H	-8.4610639476	-3.9664435078	-5.5607330388
H	-8.1941780375	-2.3800189825	-5.0881620915
H	-9.1077165435	-3.4004817114	-4.1210029712
H	-9.7215742567	-2.8610856912	-5.5850817615
N	-4.2824193171	-3.1421447281	3.9050894780
H	-4.3901079652	-4.1632012922	3.9141859360
H	-3.5833063280	-2.8654844886	4.6043282253
H	-5.1824493305	-2.7010709213	4.1278795047
H	-3.9738137497	-2.8388232048	2.9739642547
N	-4.3676605076	-6.3966241646	0.2885571590
H	-4.9192861327	-5.5367229726	0.3911008440
H	-3.6535277039	-6.2607050097	-0.4365462871
H	-4.9865789861	-7.1701377806	0.0186458275
H	-3.9112497447	-6.6189300580	1.1810283515
N	-4.7300961439	2.4585572314	-5.3993147794
H	-4.5506029305	2.4352113279	-4.3886351800
H	-3.8437082042	2.5865627343	-5.9014758065
H	-5.3622684021	3.2378645849	-5.6167401892
H	-5.1638048638	1.5745902558	-5.6904069577
N	-3.9861951870	7.8307821043	-1.0307920386
H	-3.7877370441	6.8701363954	-1.3341240104
H	-3.8818789758	8.4697682699	-1.8276919656
H	-3.3284023261	8.0983159276	-0.2891925984
H	-4.9467622088	7.8849068886	-0.6721598753
N	-3.6534968430	1.9415914349	4.5047176274
H	-3.1459002139	1.3318587522	5.1564904523
H	-4.1271032749	2.6856099090	5.0304249064
H	-4.3498710738	1.3890136686	3.9909508836
H	-2.9911123151	2.3598828158	3.8410049020
N	-8.5016394155	-1.8778036316	3.4308258936
H	-9.2856307119	-1.2255635168	3.5498913265
H	-8.8653996727	-2.8232845296	3.2635565534
H	-7.9248335887	-1.8783227972	4.2802552860
H	-7.9306944522	-1.5840430475	2.6296005244
N	-2.4276094728	2.2483850465	-1.5391753257
H	-2.9624312466	2.1003399921	-2.4030528805
H	-1.7793280687	1.4645287343	-1.3994069875
H	-3.0729428807	2.3050199018	-0.7425745411
H	-1.8957362162	3.1236514137	-1.6116677349
N	1.3912608431	2.1561954850	-2.8862554564
H	1.3415224168	2.0392761798	-1.8673874263
H	1.9710344290	1.4103152182	-3.2884493176
H	1.8068927375	3.0689620038	-3.1061257242
H	0.4455937407	2.1062284245	-3.2830583650
N	2.3955285015	-1.0203540380	-0.1012531687
H	2.8946804746	-0.7704902387	-0.9630254629
H	2.4309341171	-0.2319943069	0.5556106393
H	2.8397153121	-1.8417485425	0.3256480719
H	1.4167845884	-1.2371828205	-0.3232467624
N	-12.8690122950	-5.2052603578	0.3169163540
H	-12.6367071255	-5.5761374418	-0.6119111835
H	-12.0389680063	-5.2476842042	0.9197939381
H	-13.6220138358	-5.7670141438	0.7312313159
H	-13.1783599862	-4.2302060027	0.2285504409
N	-0.8783066193	-0.6238362782	-4.4867479587
H	0.1450083704	-0.6149611300	-4.5703168593
H	-1.2762864965	0.1165446670	-5.0763942584
H	-1.1432007001	-0.4614764297	-3.5081234497
H	-1.2387466544	-1.5354522114	-4.7921573487
N	-1.5460384749	5.8678779388	-4.5678899015
H	-1.7805492247	5.9314444788	-3.5702927379

H	-0.5387110251	6.0222381065	-4.6931856833
H	-2.0690477632	6.5817251031	-5.0885808216
H	-1.7958461149	4.9361041285	-4.9194993916
N	-4.6547056850	-2.5336650189	-5.5208327590
H	-3.6290301906	-2.5808196598	-5.5193104045
H	-4.9543352293	-1.6093935164	-5.8527649983
H	-5.0057130786	-2.6854499141	-4.5679473098
H	-5.0297432427	-3.2589970311	-6.1433083219
N	-9.6962807648	1.6038693403	-4.6824849010
H	-9.3841953121	1.0585163890	-3.8704326082
H	-8.9651116944	2.2750244876	-4.9455005740
H	-10.5566197774	2.1110009588	-4.4440109425
H	-9.8791959713	0.9709349947	-5.4699946885
N	-7.6314232802	2.3757262714	3.3395854296
H	-8.6529585390	2.2829691982	3.3853886830
H	-7.3200052367	2.2551448900	2.3686510250
H	-7.1957443164	1.6578508482	3.9304104809
H	-7.3569860237	3.3069400590	3.6738915740
N	-6.3993818761	-8.0565141433	-1.4561011581
O	-6.6299267542	-7.6974852927	-0.2894970031
O	-5.2482122942	-7.9737174782	-1.9154171898
O	-7.3200073210	-8.4983400148	-2.1633898509
N	-1.6812752686	2.3929130051	-5.0823897796
O	-2.1639068519	3.4995344434	-5.3747485628
O	-0.8932293465	2.2916927166	-4.1275306336
O	-1.9866898532	1.3875110460	-5.7448906759
N	1.8546839146	1.9542687411	0.8810950532
O	2.1244295603	3.0568393454	1.3856674069
O	1.9476601554	0.9199022142	1.5626075018
O	1.4919617361	1.8860646089	-0.3049907038
N	-10.4603121758	-4.4389842183	2.2471078316
O	-10.7283329838	-5.5621880390	1.7892913459
O	-9.3699739025	-4.2410435654	2.8083563137
O	-11.2826303030	-3.5137203057	2.1436757520
N	-6.4798569961	-5.4817511234	-6.0488665340
O	-7.7052147042	-5.2796073759	-6.0232871786
O	-6.0137179237	-6.5323586646	-5.5777443943
O	-5.7206377492	-4.6332866468	-6.5455684290
N	-8.5941590986	3.5232263440	-0.0824521710
O	-7.7631861670	2.9322604728	0.6269569049
O	-9.3605521254	4.3592724558	0.4241902458
O	-8.6587390553	3.2781459063	-1.2985046426
N	-1.3060120083	-6.6243103442	-2.0180925216
O	-2.2718842959	-6.2625644831	-1.3258147110
O	-0.1466281217	-6.4186036972	-1.6224516619
O	-1.4995237631	-7.1917633091	-3.1060120678
N	-1.8379337926	5.4701129727	-1.4192514943
O	-1.7105333572	6.5398223524	-2.0377245195
O	-1.1476982573	4.4858483944	-1.7320070803
O	-2.6555704217	5.3846681025	-0.4880221334
N	-2.1093006761	-3.4993609590	-4.1047048632
O	-2.0368647763	-2.8924154624	-5.1860873652
O	-2.9935818064	-4.3541289482	-3.9302443286
O	-1.2974547919	-3.2515382669	-3.1977821657
N	-7.6807894733	-0.1453938547	0.7721923196
O	-8.7842377872	-0.6457596565	0.4982328450
O	-6.9038643001	0.1825322215	-0.1398765784
O	-7.3542660698	0.0270460098	1.9582216471
N	-12.1560818671	3.4976498374	-2.8445728055
O	-11.8103798975	2.8436745648	-3.8424828225
O	-13.0183745708	3.0449665443	-2.0735040406
O	-11.6394907172	4.6043092939	-2.6177313708
N	-1.9886900502	-3.0825010016	1.3818740611
O	-1.7614029958	-3.7898458191	0.3863732752
O	-3.1386234425	-2.6653885014	1.5979487389
O	-1.0660429695	-2.7922684505	2.1613007969
N	-7.3617613179	4.6173164999	-5.2013866826
O	-7.9991670536	3.5719398993	-4.9918166421
O	-7.8687029754	5.7186435251	-4.9310038978
O	-6.2174130033	4.5613660302	-5.6813398942
N	-7.2373722580	7.7099235696	-1.1019384760
O	-8.3425100186	7.1598950084	-0.9635295781
O	-6.4372436698	7.7438938935	-0.1523816476
O	-6.9323628402	8.2259822225	-2.1899050781
N	-2.8059782862	-5.9448389401	3.0756131830
O	-2.4951567659	-6.7727138172	2.2032422289
O	-1.9215882219	-5.3909865385	3.7494971805
O	-4.0011908331	-5.6708162442	3.2741002992
N	-11.2874716855	-1.1573781591	-5.3732511121
O	-10.2948710463	-0.6506862793	-5.9219087656
O	-11.4391298280	-2.3902495814	-5.3800168198
O	-12.1284148593	-0.4311980321	-4.8178273036
N	1.6497701219	-3.6057569352	1.6966985866
O	1.3786996497	-4.5791126544	2.4192754711
O	1.6848070967	-3.7393133526	0.4622136719
O	1.8858038092	-2.4988439076	2.2086070289
N	-1.7132892954	2.5338935364	1.8456686043
O	-1.9182600142	1.3098012562	1.7946489528
O	-1.9957016892	3.1685318859	2.8754721371
O	-1.2259057904	3.1233479417	0.8668839351
N	-2.8776422901	8.6062836816	-4.1304047579
O	-3.2200843524	7.8992917805	-5.0926489040
O	-3.7328311545	9.0287098528	-3.3346497409
O	-1.6800103994	8.8908496406	-3.9639154948
N	2.3381706618	-0.8811309972	-3.2199909181
O	1.6792644899	-0.4004161250	-4.1568867389
O	3.0010789460	-0.1353914778	-2.4800969481
O	2.3341685464	-2.1075863763	-3.0229889087
N	-6.0108700897	-3.2262275531	0.8876636742
O	-6.3712400840	-2.8485917006	2.0148471291
O	-5.6229714052	-2.3959824584	0.0490662713
O	-6.0383988021	-4.4341094728	0.5990773898
N	-11.2049829392	-6.2856279280	-2.4423643365
O	-10.3871611593	-6.6267582749	-1.5718383268
O	-10.8672385447	-6.2642987959	-3.6375595937
O	-12.3605500441	-5.9658264556	-2.1176948275
N	-4.0497150101	-0.1735070726	-2.8544671862
O	-3.8836893571	0.9651492790	-3.3223508326
O	-4.9159555157	-0.3677414012	-1.9856068276
O	-3.3494995937	-1.1179298558	-3.2554442214
N	-1.6848324541	-1.1942115528	5.2496425861
O	-0.4807967811	-1.2802661488	4.9565374590
O	-2.3639621400	-2.2241769343	5.3944600350
O	-2.2097388638	-0.0781906768	5.3979303837
N	-1.0475314653	7.7697913750	0.8558031406
O	-2.2500166833	8.0793367693	0.8908582430
O	-0.6160826324	6.8697015815	1.5952003135
O	-0.2764944594	8.3603362496	0.0813502420
N	-10.4628800288	-0.0448316982	-2.0943054914
O	-9.3898607282	-0.1003376535	-2.7176565806
O	-10.7917066953	1.0042324834	-1.5160708700
O	-11.2070732619	-1.0383907242	-2.0491889872
N	1.5078697078	5.6715089808	-3.0400058642
O	1.5317525136	6.0482198008	-1.8565632249
O	2.0208177227	4.5853647233	-3.3565384800
O	0.9710384551	6.3809429895	-3.9069165857
N	-4.8621372005	2.9867531216	1.0270218645
O	-5.0770139285	4.1995023177	0.8655124526
O	-5.0423833308	2.4669708862	2.1407344266
O	-4.4670140240	2.2937856032	0.0748179476
N	-13.9148449708	-1.8953246004	-0.9706370114
O	-13.6386001174	-0.7349059545	-0.6240316765
O	-14.3380407346	-2.1169418273	-2.1172882492
O	-13.7678939423	-2.8341267751	-0.1705904643
N	-5.7609638385	-0.4129163682	4.3739663952
O	-6.2929922574	0.6418448993	4.7579486096
O	-6.2109495389	-1.5080981171	4.7496094669
O	-4.7789489286	-0.3724958543	3.6143404977
N	-6.5876506533	-0.1429157923	-4.9305128115
O	-7.1716094467	-1.2312010008	-4.7976770352
O	-5.6665150627	-0.0290301268	-5.7560775816
O	-6.9248277221	0.8314845352	-4.2377832599
N	-5.0267546360	4.7407038734	-2.7284647489
O	-4.3274553044	5.7309905114	-2.9992612229
O	-6.1908372680	4.8944877925	-2.3231500031
O	-4.5619709616	3.5966323954	-2.8629831289
N	-0.9998189479	-0.6511196112	-0.9508833928
O	-0.0952960770	-1.4279357139	-0.6024396677
O	-0.8169872509	0.1292626166	-1.8998797363
O	-2.0871743911	-0.6546857391	-0.3503302908
N	-5.6185049713	4.9815688092	4.0272674121
O	-5.0726387474	5.9146840591	3.4154309088
O	-6.8387343510	4.7860673713	3.9014208401
O	-4.9441412727	4.2439544033	4.7649510812
N	-9.7872490345	-3.5451673079	-1.4161983123
O	-8.7752424881	-3.5038951615	-0.6970618366
O	-10.9127109956	-3.6426503153	-0.8996227143
O	-9.6737935287	-3.4889564017	-2.6519113808
N	-10.7347346354	1.0463934079	2.8538767458
O	-11.7130311056	0.9868361637	2.0907208888
O	-10.2960142528	0.0117936504	3.3831646434
O	-10.1951581134	2.1405512904	3.0877448933
N	-6.0238458466	-4.3543545351	-2.8072129149
O	-5.7680647394	-3.2245380256	-3.2556642399
O	-5.1575604269	-4.9832093413	-2.1770469722
O	-7.1459132767	-4.8553166418	-2.9889276788

n=38 OPLS/AA

N	-3.8764179612	-4.9058514699	2.7808874840
H	-3.7241501414	-5.8989187013	2.5690666103
H	-4.7789217309	-4.6075539849	2.3926346778
H	-3.1228021742	-4.3473607829	2.3632908818
H	-3.8797976500	-4.7695733776	3.7985575599
N	1.0247477995	-5.2619661987	1.1302153504
H	1.9231012506	-5.7467994723	1.2403692038
H	0.5440464556	-5.2219210742	2.0366133266

H	0.4415436696	-5.7710308077	0.4557068694
H	1.1903006973	-4.3081139129	0.7881721089
N	-2.3567579086	-8.5329362088	0.8172674878
H	-1.6684928183	-8.9509652679	0.1802596631
H	-1.9080630884	-7.7904645489	1.3664882796
H	-2.7199658648	-9.2550698445	1.4503855786
H	-3.1305091926	-8.1352455810	0.2719358097
N	-12.6187823083	-6.1545214514	-1.5554058818
H	-12.1541017097	-6.8254180789	-2.1784674792
H	-11.9144355040	-5.6745956373	-0.9828663830
H	-13.2750666183	-6.6535873747	-0.9434748132
H	-13.1315249484	-5.4644853682	-2.1168154585
N	-8.0254366421	0.2651550273	-3.9894284996
H	-7.5619283576	-0.5214559998	-3.5197059646
H	-8.6063408872	-0.0894745802	-4.7582085624
H	-7.3161744669	0.9061799383	-4.3639545393
H	-8.6173024053	0.7653699847	-3.3158444746
N	-8.2434873443	-5.3679219330	-4.4451901319
H	-7.3599989711	-5.5866023309	-4.9204408822
H	-8.7638527832	-6.2362544339	-4.2735708932
H	-8.8047764355	-4.7437134189	-5.0364208916
H	-8.0453203268	-4.9051177611	-3.5503283237
N	-13.0865581991	-0.8815878859	0.3410265959
H	-12.5801567411	0.0064828090	0.2455064316
H	-13.7039902833	-1.0123251202	-0.4688623363
H	-13.6482212031	-0.8645842263	1.2003758088
H	-12.4138640758	-1.6559241412	0.3870863864
N	-5.5034816213	-2.7713960208	-4.7606068621
H	-6.3720296399	-2.6022311131	-5.2814174524
H	-5.5927879475	-2.3878755339	-3.8123605325
H	-4.7207832371	-2.3138406668	-5.2425423987
H	-5.3283265067	-3.7816366047	-4.7061075721
N	-4.4945954667	2.4499949241	-0.6522375652
H	-3.4984171354	2.4676834373	-0.4041409178
H	-4.6162558270	2.8105632593	-1.6058747697
H	-5.0215336681	3.0366842281	0.0053088716
H	-4.8421742659	1.4850487890	-0.6042432032
N	-12.2476083460	-2.6405045982	6.4141925743
H	-12.1182195640	-2.2497595836	7.3548375521
H	-12.5683427766	-3.6130325759	6.4887140011
H	-11.3557793026	-2.6122137769	5.9061806542
H	-12.9480916147	-2.0870120758	5.9070390059
N	-8.7721379669	2.4917414410	4.0222832653
H	-8.3155321292	3.2647182135	3.5240322083
H	-9.4988878860	2.8666438863	4.6431837380
H	-8.0747028511	1.9840953599	4.5791631803
H	-9.1994285566	1.8515090571	3.3427534494
N	-5.0148237188	-2.0814560217	0.4511325071
H	-4.3230515953	-2.7875016279	0.1733069708
H	-5.0054140899	-1.9764060927	1.4724603961
H	-5.9490164334	-2.3790865858	0.1462791925
H	-4.7818120830	-1.1828304680	0.0124831985
N	-0.8662907203	0.4930475645	1.7002271353
H	-1.2213527860	1.1235876957	0.9718136744
H	-0.5206861602	1.0463147106	2.4931253876
H	-0.0999547720	-0.0737333926	1.3184888939
H	-1.6231695090	-0.1239781416	2.0174798759
N	-11.6035173420	-2.4104898818	-3.4811455592
H	-11.2614839766	-2.8983471299	-2.6449371043
H	-12.6082365211	-2.5890851572	-3.5946258848
H	-11.0995147337	-2.7531586563	-4.3074596676
H	-11.4448338036	-1.4013690591	-3.3775587656
N	-7.8202212868	-5.7106678430	-0.0241841329
H	-7.1463243933	-5.6940135397	-0.7986657070
H	-8.2527723272	-4.7845624786	0.0730945242
H	-7.3345579536	-5.9547812105	0.8468929674
H	-8.5472298169	-6.4093141267	-0.2180590704
N	-12.8995588606	1.0996254144	4.5102923078
H	-12.2160810216	1.3182668767	3.7759286021
H	-12.4439457744	1.1533599426	5.4288585828
H	-13.2664239646	0.1516918125	4.3651461542
H	-13.6717840159	1.7751832387	4.4712351766
N	-8.3126741227	3.1924870802	-0.5062285252
H	-7.9888437888	2.7741654202	-1.3862154576
H	-7.5025776043	3.4942887681	0.0477516707
H	-8.8510951870	2.4981572624	0.0250382494
H	-8.9081795953	4.0033364627	-0.7114894203
N	-4.0321356373	2.1036943912	3.9665095874
H	-4.4330692920	1.9263477040	4.8949687706
H	-4.3131809754	1.3541776948	3.3234909834
H	-3.0080043488	2.1295862772	4.0352135773
H	-4.3742883236	3.0046657160	3.6123659229
N	-6.0044541959	-9.9934224542	1.0510878912
H	-5.7145357573	-10.4907730804	0.2008962944
H	-5.5301038300	-10.4017355610	1.8650331121
H	-7.0208333795	-10.0797833306	1.1683511771
H	-5.7523435344	-9.0013983292	0.9700701530
N	-10.1004141847	-6.6890150786	7.5922414106
H	-9.1057439964	-6.9409644913	7.5549747024
H	-10.4782533601	-6.6448508251	6.6385529071
H	-10.2047745570	-5.7731399493	8.0444771726
H	-10.6128838567	-7.3971052939	8.1309608240
N	-3.2076623112	-4.8660223450	-2.3286969505
H	-2.4441027650	-5.5489707223	-2.3979387805
H	-4.1093710804	-5.3544009700	-2.3801002542
H	-3.1374996309	-4.1945920700	-3.1023209601
H	-3.1396750248	-4.3661262828	-1.4344278747
N	-3.8653820731	-7.4835059042	5.4633694921
H	-3.0215576333	-6.9517485726	5.2195856685
H	-3.6758723883	-8.4882266021	5.3692574094
H	-4.6308506294	-7.2190088722	4.8322318420
H	-4.1332468196	-7.2750390520	6.4324028111
N	-0.7345234716	-2.6910156461	4.0178256411
H	-1.4360990722	-2.8886836179	4.7409820192
H	0.0506704288	-3.3454692319	4.1147572056
H	-0.3933098816	-1.7286670057	4.1259639988
H	-1.1593560448	-2.8012429213	3.0896000453
N	-3.4016841121	1.2017643853	-4.5234022724
H	-4.3940262200	1.4469622063	-4.4265969678
H	-3.3162860916	0.2043850381	-4.7518235969
H	-2.9140161838	1.3936403658	-3.6404531664
H	-2.9824089194	1.7620701696	-5.2747352645
N	-3.4796205327	-0.5168931238	7.2587091309
H	-2.6634580853	-0.2131262723	6.7147967695
H	-3.7509946825	-1.4637465842	6.9687793450
H	-4.2606820931	0.1287340687	7.0933492470
H	-3.2433464750	-0.5194334116	8.2579106325
N	0.0055312986	-1.7768228393	-1.9430648457
H	-0.1554745756	-0.8358537428	-1.5650564258
H	1.0152203121	-1.9530029864	-2.0040840001
H	-0.4110670605	-1.8455810374	-2.8789887964
H	-0.4265536384	-2.4728526742	-1.3241297923
N	-10.7913467465	-9.5060489275	-0.2500876203
H	-10.1087547416	-10.0458901433	0.2947754031
H	-10.5497162321	-8.5089387178	-0.2098308229
H	-11.7300272960	-9.6468154365	0.1414467203
H	-10.7768880513	-9.8225519382	-1.2267412510
N	-6.5019885961	-4.3480461735	7.7071410524
H	-5.7120666026	-3.7478679305	7.4424801026
H	-7.0439040036	-3.8924084063	8.4507515699
H	-6.1478466318	-5.2504230152	8.0455712946
H	-7.1041363772	-4.5014847576	6.8897609849
N	-12.0213339100	-8.5653392842	3.8623937604
H	-11.6524692544	-7.6606233973	4.1780900184
H	-12.2016950950	-9.1643343497	4.6765862684
H	-11.3347878136	-9.0195372314	3.2487210412
H	-12.8963831179	-8.4168612774	3.3461780213
N	-7.1212940800	-10.5248876638	5.3358524142
H	-7.4236383716	-10.1636962308	4.4235121401
H	-6.7193996845	-9.7600749281	5.8906289995
H	-6.4135201053	-11.2556788548	5.1971593718
H	-7.9286184530	-10.9201002897	5.8321082566
N	-8.9235951919	-0.2548631380	7.4639301697
H	-9.1326422696	-0.3671551985	6.4649677529
H	-9.2905363934	0.6465261162	7.7911705283
H	-7.9071026955	-0.2825348914	7.6061019073
H	-9.3640996125	-1.0162886879	7.9934795172
N	-6.7925686914	-1.1907531627	4.2897587644
H	-7.2305456601	-1.7141902326	5.0568480072
H	-7.1635531492	-0.2335043642	4.2730269011
H	-7.0000461980	-1.6538691772	3.3971714210
H	-5.7761301848	-1.1614493867	4.4319894754
N	-8.4272112377	-6.8688505375	4.2249083304
H	-8.4428124270	-6.0081612538	4.7845570216
H	-7.4644531773	-7.0665145661	3.9278215848
H	-9.0231555801	-6.7498131893	3.3973118257
H	-8.7784237816	-7.6509123024	4.7899434346
N	-7.0176358093	-9.2915552032	-3.0744774968
H	-6.5095870737	-10.1740763290	-3.2059272681
H	-6.7400188470	-8.8664752633	-2.1820255080
H	-8.0276506891	-9.4760451858	-3.0661761149
H	-6.7932861326	-8.6496248943	-3.8437812243
N	-10.7197928979	-3.6071448351	2.8592297785
H	-9.7473643220	-3.9364906188	2.8714820113
H	-11.0082313338	-3.4278379486	1.8902679657
H	-10.7944741676	-2.7403640391	3.4045272053
H	-11.3291008211	-4.3238870546	3.2706419435
N	-14.0868287143	-4.9746134025	1.7228865879
H	-14.3886633660	-4.5683080683	0.8295513116
H	-14.1323142851	-4.2585263494	2.4573149919
H	-13.1219168046	-5.3145628640	1.6356232261
H	-14.7044206953	-5.7570559328	1.9690559521
N	-8.6834814296	-0.8086310504	0.4697542120
H	-8.2475776565	-1.6859239389	0.1622136682
H	-8.0070270940	-0.0412015160	0.3820266394
H	-8.9756769951	-0.8969153954	1.4500924954

H	-9.5036435484	-0.6104842059	-0.1153162547
N	-2.6007455192	-8.9077907126	-3.4635349194
H	-3.2570928820	-9.6563235552	-3.2122525672
H	-3.1253142574	-8.0559311847	-3.6946142760
H	-2.0449426395	-9.1985597540	-4.2764137834
H	-1.9756329372	-8.7203490856	-2.6708588062
N	-6.3982726299	-7.4616691175	7.2979187828
O	-7.5338916456	-7.0786678035	7.6245643943
O	-5.4027923422	-6.7837711341	7.6020424941
O	-6.2581337891	-8.5225692690	6.6671489521
N	-3.7668099318	-3.5130339807	5.9697018245
O	-4.2077478066	-4.4996301280	5.3571499951
O	-2.6334170650	-3.0761722431	5.7097203972
O	-4.4592654812	-2.9632991286	6.8422357837
N	-10.6848688835	-3.5346937281	-0.2548467060
O	-9.5513030634	-3.7177804569	0.2189947149
O	-11.5663925214	-2.9951959845	0.4342658910
O	-10.9369112686	-3.8911050300	-1.4178016602
N	1.3125639000	-2.2073571148	0.9634635734
O	0.9470191799	-1.0425326737	0.7341624834
O	1.7184224629	-2.9252036546	0.0344893632
O	1.2722500248	-2.6543353759	2.1217398060
N	-3.7772359401	-1.2706801213	3.4328965133
O	-4.1459970099	-0.6603542042	4.4500176759
O	-4.4600125142	-2.2090881120	2.9899241622
O	-2.7256974497	-0.9425977836	2.8587472397
N	-7.4924755557	-2.9461917671	-1.9777201761
O	-7.2249842240	-2.6456124700	-0.8025102440
O	-7.6879622622	-4.1335189109	-2.2860335104
O	-7.5644802388	-2.0594432065	-2.8446174719
N	-9.0472771656	-2.5054157949	-5.5366601921
O	-9.8643582671	-3.3944367342	-5.2450471712
O	-9.4026871101	-1.3152348296	-5.5497350530
O	-7.8747851755	-2.8065760634	-5.8151985763
N	-10.6480583554	-6.4931531193	1.1358938243
O	-10.0388022437	-6.6461086410	2.2075418587
O	-11.7087392310	-5.8471160057	1.1111948563
O	-10.1966332282	-6.9862351082	0.0889439152
N	-5.2757112669	-6.5657456378	-4.4714903931
O	-5.4761470480	-5.3539896659	-4.6572075795
O	-6.1986622888	-7.3804694335	-4.6370333142
O	-4.1523235595	-6.9627781335	-4.1202300029
N	-1.1183630988	-6.0691716601	3.4294142586
O	-0.0935429983	-5.3713415160	3.5055375971
O	-1.4052560922	-6.6370490161	2.3625376819
O	-1.8562907998	-6.1991245528	4.4201682942
N	-6.0904833081	0.9513931458	6.3693152798
O	-6.9834522002	1.2793870893	5.5705384388
O	-4.9888140122	1.5244714129	6.3389183551
O	-6.2991838800	0.0503202098	7.1984897131
N	-2.0263013320	-3.6257451429	0.5050619307
O	-1.1089072653	-3.4551993400	-0.3148893889
O	-3.1777448081	-3.8684701359	0.1072172248
O	-1.7922517341	-3.5535658946	1.7228589365
N	-10.0233224803	-0.7987999062	4.0167236019
O	-11.1542121169	-1.2739699541	4.2124784649
O	-9.4423299612	-0.1833286836	4.9259324766
O	-9.4734249202	-0.9391011937	2.9117589746
N	-10.7893310144	2.0528271784	6.6793741684
O	-9.9140607378	2.1410463614	7.5563788603
O	-11.6816060293	1.1940961414	6.7765555177
O	-10.7723262624	2.8233396528	5.7051873431
N	-4.1413764369	-10.3329530521	3.5859572738
O	-5.2090486779	-10.9424452895	3.4081565169
O	-3.3998769820	-10.0844979659	2.6208321320
O	-3.8152033882	-9.9719156101	4.7288840927
N	-10.0741655496	-9.7186470645	6.0995604685
O	-9.5258248083	-10.8073167443	6.3385974334
O	-9.3914389300	-8.7448375299	5.7409558615
O	-11.3052339016	-9.6037868268	6.2191282067
N	-0.8218382068	-7.2335272944	-1.4991111760
O	-0.9238318545	-8.4715137499	-1.5012889390
O	-1.2638475439	-6.5722358627	-2.4532282019
O	-0.2778347840	-6.6568318063	-0.5428156174
N	-4.6076799638	-10.6849420174	-1.8124023533
O	-5.6550497342	-11.0773611250	-1.2719935091
O	-4.5499182818	-10.5751580370	-3.0483752400
O	-3.6180710787	-10.4023066625	-1.1168377508
N	-10.8719314286	1.6119684941	1.5402585438
O	-11.1524623213	1.7249399748	2.7450646584
O	-9.7041390089	1.7882411158	1.1552822610
O	-11.7591936699	1.3227241590	0.7204280520
N	-2.4426975077	-2.0621997888	-4.5503189218
O	-2.7505898845	-3.2424161105	-4.3151389286
O	-1.3616450408	-1.6086829222	-4.1396293476
O	-3.2158582203	-1.3354997487	-5.1961890091
N	-14.0364981126	-1.6965682437	3.5965952922
O	-14.0698276420	-2.8872849391	3.2443158513
O	-13.9397396851	-1.4076157165	4.8008220792
O	-14.0999270618	-0.7948033493	2.7446472602
N	-10.3455807752	0.1167782157	-2.0490871753
O	-9.4313219466	0.9279829622	-1.8275119860
O	-10.8907750103	0.0849097540	-3.1647777946
O	-10.7146456659	-0.6625586963	-1.1549710254
N	-9.6458810720	-3.2168532592	8.6830443753
O	-8.4442237212	-3.0585610880	8.9550390644
O	-10.1349190363	-4.3586626485	8.6715945254
O	-10.3585010320	-2.2333352495	8.4224993264
N	-6.2930934644	4.0021364263	1.9872317067
O	-7.4382508533	4.1094310443	2.4564015380
O	-6.1316270732	3.9030947087	0.7595765216
O	-5.3094016749	3.9938835193	2.7457176711
N	-8.9458403294	-9.8483314782	2.2405112138
O	-10.1432879971	-9.6684219628	2.5175844180
O	-8.0736942121	-9.6641654740	3.1056481648
O	-8.6205385172	-10.2124072908	1.0983001392
N	-5.7310424377	-7.2862882199	2.5587219706
O	-6.7511477855	-7.3338356692	1.8515146243
O	-5.8322744742	-7.1056388672	3.7835222796
O	-4.6097041508	-7.4193902306	2.0411285910
N	-14.0558462729	-3.3412106332	-1.6904754312
O	-14.4001799106	-3.8850246742	-0.6280637664
O	-14.0605878660	-2.1025765744	-1.7841829373
O	-13.7067707610	-4.0360312102	-2.6591803699
N	-8.7464596602	-3.6438893645	5.7545444917
O	-9.9360336600	-3.4364102785	5.4631958364
O	-7.9866461414	-2.6876925901	5.9812360356
O	-8.3166988334	-4.8075661618	5.8192016551
N	-4.7804209699	-0.8726992826	-2.2935366952
O	-5.3182266182	-0.3702320021	-3.2941912948
O	-4.4961750568	-2.0818724582	-2.2824731018
O	-4.5268610304	-0.1659928184	-1.3039448924
N	-13.6933963824	-8.1271742353	1.1535439491
O	-13.0333599039	-9.1676992501	0.9964533650
O	-14.0384904354	-7.4556923241	0.1671160256
O	-14.0083390614	-7.7581308347	2.2970633774
N	-10.0687068660	-8.3262352373	-3.0796791875
O	-11.2081477795	-7.9703883976	-2.7360728107
O	-9.3702271464	-7.5780995523	-3.7835520376
O	-9.6277453170	-9.4302186506	-2.7194124240
N	-5.2259463298	-7.1599700825	-1.1438649584
O	-5.5395182144	-6.0124279245	-1.5014087734
O	-6.1028583909	-8.0288438229	-1.0056565243
O	-4.0354614258	-7.4386387244	-0.9245294009
N	-6.1545966768	0.8680887718	1.6032848225
O	-4.9209773510	0.8227599773	1.7416559548
O	-6.8907653059	0.9057133120	2.6031138351
O	-6.6520477741	0.8757930322	0.4650837613
N	-7.2293082334	-3.7455799088	2.2776178704
O	-6.2536450659	-4.4060434514	1.8840607545
O	-7.2404795133	-2.5112289507	2.1387984706
O	-8.1938008973	-4.3194677862	2.8099948147
N	-1.0764870889	0.6508775670	4.8285378868
O	-1.4538882191	1.6221667465	4.1523843336
O	-1.5274025538	0.4780738749	5.9730168243
O	-0.2481698269	-0.1476085633	4.3602121255
N	-1.6122331002	1.4244673744	-1.2376731132
O	-1.9640287480	1.9829931775	-0.1853867166
O	-2.1706240106	1.7110389463	-2.3096312450
O	-0.7020458093	0.5793693190	-1.2180013621
N	-12.0063178813	-5.6351328146	5.1449311558
O	-12.5132944807	-5.2148696523	6.1981982222
O	-12.3980103862	-5.1988567601	4.0498247241
O	-11.1076480535	-6.4916727207	5.1867705549
N	-6.0531458375	2.5969457534	-3.4864096528
O	-7.0753274187	2.6723883200	-2.7846467689
O	-6.0901977366	2.0010636954	-4.5757074269
O	-4.9939115045	3.1173856638	-3.0988744507

n=39 OPLS/AA

N	-6.0777871669	2.8210701909	1.1124240831
H	-6.0500397733	1.7968273187	1.0461464973
H	-6.6887613436	3.0972957163	1.8900131134
H	-6.4424935257	3.2110347004	0.2354106982
H	-5.1298539982	3.1791220307	1.2781259591
N	-14.7660346130	-2.1991922962	5.4556036822
H	-15.0713778435	-2.5498819368	6.3710371233
H	-15.0918267117	-1.2332268916	5.3331086580
H	-15.1600821189	-2.7899309872	4.7139906523
H	-13.7408520753	-2.2237297107	5.4042791867
N	-7.9856587266	-6.8385671410	9.3509688507
H	-8.8858785204	-7.2127631920	9.0287612531
H	-7.9594260163	-5.8247063338	9.1908664025
H	-7.2225999321	-7.2870938821	8.8305838368
H	-7.8747313146	-7.0297055206	10.3536635965

N	-4.0848130181	-1.1563132141	5.6674679629
H	-4.3242587615	-0.2075821520	5.9786129538
H	-3.3958600781	-1.1037537264	4.9079839063
H	-4.9311085470	-1.6295381196	5.3297142202
H	-3.6880249189	-1.6843779343	6.4535610742
N	-13.1463273339	-2.0894427571	1.6155066333
H	-13.4777900896	-1.5021881420	0.8412324208
H	-13.8951054420	-2.7299749573	1.9040972270
H	-12.8815710244	-1.4931201164	2.4083111040
H	-12.3308431024	-2.6324872409	1.3083850273
N	-5.1395537320	-4.4366741746	11.2633035997
H	-5.6103545977	-3.5506105836	11.0454184118
H	-5.6996098751	-4.9635323171	11.9437389749
H	-4.2124228725	-4.2431509238	11.6598040976
H	-5.0358280412	-4.9894020109	10.4042527025
N	-11.2161347657	3.8627960666	1.8228645496
H	-10.7615273370	4.4769990381	1.1370636359
H	-11.7737735138	4.4291650903	2.4728644274
H	-10.5002729801	3.3458356194	2.3468200095
H	-11.8289647893	3.1991851170	1.3347094576
N	-2.2333316425	-5.0089449517	8.2056177966
H	-1.7680986205	-4.9055652998	9.1150720178
H	-1.5412795490	-4.9086322637	7.4537960425
H	-2.9544781262	-4.2849119211	8.1058207833
H	-2.6694698212	-5.9366702213	8.1477832286
N	-4.7464088690	-6.1828955051	2.6109981955
H	-4.2670727108	-6.1187670892	1.7052606555
H	-4.0512005432	-6.2848122518	3.3596857444
H	-5.3710439774	-6.9977982140	2.6107145342
H	-5.2963177777	-5.3302044030	2.7683309657
N	-5.9835045045	4.2526576705	10.2961070570
H	-6.3669787174	3.3356925971	10.0385052716
H	-5.0984807838	4.4055600205	9.7985243294
H	-5.8162176531	4.2814548050	11.3087379663
H	-6.6523412370	4.9879223664	10.0386604096
N	-6.5026802871	5.5798847905	5.1849744842
H	-6.1035753441	5.4286977555	6.1188341217
H	-7.1202328688	6.3999140874	5.2053569859
H	-7.0419667998	4.7521754819	4.9051479630
H	-5.7449457470	5.7387516898	4.5105597756
N	-3.7129675245	-0.9101504404	1.4534065607
H	-4.0341384903	-1.4674126004	2.2537466092
H	-3.0170526443	-0.2250765724	1.7705988690
H	-4.5118878478	-0.4183533593	1.0361566691
H	-3.2887914282	-1.5297597722	0.7531248750
N	-5.9751965977	-0.0746849880	10.0275175961
H	-5.2757017783	0.6275553930	10.2954756947
H	-5.7891808401	-0.9514954286	10.5283531725
H	-5.9231340575	-0.2408472877	9.0156306923
H	-6.9127690335	0.2660480553	10.2706110858
N	-14.5351783110	2.0935704499	3.4439721127
H	-14.8334380348	3.0576953286	3.6330235685
H	-14.7651168908	1.8481776626	2.4738456700
H	-15.0208528048	1.4534862708	4.0832279721
H	-13.5213058039	2.0149234765	3.5857914243
N	-6.9121781756	2.4919845709	6.9893520673
H	-7.3948744392	3.3785723595	7.1769753809
H	-6.6652204628	2.4407035388	5.9940547101
H	-7.5304741549	1.7081951272	7.2293931342
H	-6.0581441155	2.4404681218	7.5569852268
N	-14.6230294164	4.2979245654	7.6009431163
H	-14.7279198250	3.3232196872	7.2956817524
H	-15.5486971226	4.7371352126	7.6677861442
H	-14.0496179555	4.8099204239	6.9202853010
H	-14.1658828645	4.3214219886	8.5200189702
N	-7.5818218479	6.5032528419	0.7055351296
H	-8.0381921045	6.9942347670	1.4832876839
H	-8.1868552582	5.7400230243	0.3804974342
H	-6.6850469854	6.1173594696	1.0235236123
H	-7.4171934882	7.1613945850	-0.0651674546
N	-7.4238728786	-7.4600346100	5.7220693732
H	-6.5676152205	-7.6189599609	6.2659458788
H	-7.2385517284	-7.6468325629	4.7295990807
H	-7.7259339718	-6.4853828745	5.8362999061
H	-8.1633897598	-8.0889631963	6.0564331568
N	-9.4815758395	0.9286401618	5.0247022896
H	-9.8169502539	1.3304465886	4.1413497580
H	-9.9434565621	0.0261781800	5.1873775950
H	-9.7002125445	1.5691172494	5.7968568178
H	-8.4656843239	0.7888190203	4.9732241272
N	-3.2188309721	3.1513493216	4.6171214940
H	-3.1888329369	3.7439090934	3.7791422983
H	-4.1461269929	2.7177232020	4.6966523911
H	-3.0369186031	3.7252812767	5.4488338992
H	-2.5034453261	2.4184842912	4.5438565711
N	-10.6951798162	-5.3305730862	2.9117733273
H	-10.5549781577	-5.0942301548	3.9010769265
H	-10.2727898440	-4.6031896699	2.3229314448
H	-11.7008108579	-5.3903471691	2.7133576250
H	-10.2521402685	-6.2345251207	2.7097282765
N	-10.1975844761	0.2737164609	0.2263400981
H	-9.6841806593	-0.5561250232	-0.0930589153
H	-11.1535262531	0.2496930173	-0.1476022484
H	-9.7210521680	1.1201074014	-0.1064884754
H	-10.2315783239	0.2811896400	1.2525097206
N	-6.2775798354	2.2550388120	14.2262290330
H	-7.2198660836	2.6313372692	14.0689643034
H	-5.6125700020	2.7250693580	13.6008748192
H	-6.2750762394	1.2466471898	14.0329013518
H	-6.0028079343	2.4171017975	15.2021755045
N	-10.7763551599	4.7453796696	10.1777352376
H	-10.5728398518	3.7945221655	9.8480571768
H	-10.3760489366	5.4276070738	9.5230938228
H	-10.3635337222	4.8807547915	11.1080510770
H	-11.7929979309	4.8786337216	10.2317385527
N	-10.8721805890	-4.4268680003	9.9899747379
H	-10.9324502160	-3.5162266749	10.4604383880
H	-10.3951066285	-5.0980450055	10.6032907652
H	-10.3431708538	-4.3261118886	9.1157708543
H	-11.8179947166	-4.7670875454	9.7803994022
N	-1.6235093175	-3.5636962486	3.9773144759
H	-0.6852671587	-3.8337209817	4.2951578219
H	-1.6841121321	-2.5403308056	3.9199982090
H	-2.3236250804	-3.9112554431	4.6431042693
H	-1.8010319850	-3.9694780269	3.0509979128
N	-10.5717455482	-1.4291203168	8.0399860473
H	-10.3312549468	-1.7850409180	7.1073977706
H	-10.6361497259	-0.4048431164	8.0092776770
H	-9.8448772372	-1.7064097232	8.7100670631
H	-11.4747000486	-1.8201878563	8.3332007703
N	-12.6480903493	-5.4706502968	6.4638334168
H	-13.0289368887	-5.1921997252	7.3757858773
H	-11.8848074719	-4.8352546939	6.2032449246
H	-13.3892511872	-5.4241710343	5.7547806582
H	-12.2893662202	-6.4309754627	6.5215230954
N	-8.4463430585	-1.7852949503	12.4431521510
H	-7.7298372214	-1.2062748584	12.8965738269
H	-8.2631285608	-2.7755723330	12.6431967423
H	-8.4197831315	-1.6304923658	11.4284767082
H	-9.3726226226	-1.5288396800	12.8043617683
N	-13.5250557602	0.6002666348	9.2071652839
H	-14.0015440770	1.4243812624	9.5919120345
H	-14.1770595156	0.0738833850	8.6138302498
H	-13.2024192073	0.0013560451	9.9762213836
H	-12.7192007049	0.9014466493	8.6466978426
N	-10.9919582566	1.9014474673	12.5004834088
H	-10.4674295325	1.9280731594	11.6182154293
H	-11.1534947683	0.9248599411	12.7732790182
H	-11.8940587019	2.3759470872	12.3767885371
H	-10.4528495127	2.3769097075	13.2336497914
N	-1.7838749393	3.0359191130	9.6648549981
H	-2.2296651099	3.9126882990	9.3702654262
H	-0.7721671811	3.0939899177	9.4995979889
H	-2.1746150241	2.2549801234	9.1247698152
H	-1.9590528763	2.8820189657	10.6647864754
N	-8.3974857137	-1.9036122490	3.5683480041
H	-8.5776756190	-2.6671764836	2.9059754841
H	-7.5716646842	-2.1298176373	4.1349920149
H	-9.2116150624	-1.7839789643	4.1824463714
H	-8.2289876646	-1.0334766544	3.0499775009
N	-0.6909681963	-0.0582152025	6.9318284985
H	-0.9764266744	0.4976607759	6.1171190709
H	-1.5256204626	-0.3854580809	7.4323306247
H	-0.1211210815	0.5215237736	7.5590545418
H	-0.1407048447	-0.8665867374	6.6188089631
N	-6.2217437548	-3.7294730035	7.1868311336
H	-6.9403761256	-4.1996042635	7.7496634457
H	-5.8576093904	-4.3818194106	6.4824981049
H	-5.4555183504	-3.4222914378	7.7973830474
H	-6.6334718526	-2.9141773601	6.7177804846
N	-10.5425889071	5.1240570347	5.8319123162
H	-10.2974848252	4.1363855262	5.9685325125
H	-10.1508510448	5.4539736321	4.9420071688
H	-10.1576857398	5.6820490357	6.6030997141
H	-11.5643337798	5.2238189828	5.8140100025
N	-2.4399424861	-0.5430611595	11.9881163428
H	-3.1126199487	-0.2698406954	12.7141271601
H	-2.9457449800	-0.8025525158	11.1330942620
H	-1.8888404979	-1.3447741547	12.3164125442
H	-1.8125651729	0.2449229942	11.7888321120
N	1.1231098201	-2.6214503166	10.3261236929
H	1.3223272537	-1.6157537327	10.2702408442
H	0.8504876987	-2.9683846027	9.3990053264
H	0.3570223172	-2.7821964391	10.9905829814
H	1.9626022050	-3.1194655127	10.6446655652
N	-6.7200839313	-4.1099889217	0.2654004215

```
H     -5.7310776792    -4.3478397486     0.1256516648
H     -7.1903996641    -4.0529484927    -0.6455244856
H     -6.7899586993    -3.2042694162     0.7439662146
H     -7.1688987193    -4.8348982610     0.8375081561
N    -13.6117432916    -3.2879934443     8.4633278671
O    -12.9236480255    -2.2875439470     8.7253271402
O    -13.2180482079    -4.4175901011     8.7980419016
O    -14.6935345122    -3.1588461808     7.8666140791
N     -8.0966906420    -5.3603756608    12.0460378106
O     -7.8849410283    -4.2919103415    12.6431658317
O     -9.2388494567    -5.6276854717    11.6373366753
O     -7.1662806920    -6.1615318141    11.8576107732
N     -8.5733702264     1.6987888355    10.2852393519
O     -9.6836453917     2.2557814970    10.2771064983
O     -8.4355261016     0.5965416890    10.8411783199
O     -7.6009384030     2.2440437595     9.7374327964
N     -9.5526193726    -4.7852870634     6.5210289740
O    -10.1402592305    -4.9378075716     5.4373155419
O     -8.4059497102    -5.2375231633     6.6748116325
O    -10.1116496272    -4.1805299684     7.4509604962
N    -15.1282819967     0.7464203294     6.3629188232
O    -15.4214644794     0.3896163744     5.2097697086
O    -14.8554562915     1.9359298922     6.5945457767
O    -15.1079252028    -0.0862859487     7.2844417262
N     -1.8816088054     0.2683179203     3.8031870524
O     -1.5892671613     1.0509291255     4.7224661044
O     -2.0124922793    -0.9444266651     4.0380018682
O     -2.0430671055     0.6984516467     2.6490922555
N     -4.5866974930     5.1116115789     2.4248531114
O     -5.2948629014     5.6440809987     1.5542234643
O     -3.9669166260     4.0662911643     2.1675695743
O     -4.4983128807     5.6244629867     3.5527672036
N     -1.6263758792    -3.6518695608    11.4711364425
O     -1.2178721875    -4.3164279968    10.5044385941
O     -0.9199271213    -2.7470431747    11.9457281348
O     -2.7413292262    -3.8921377042    11.9632429946
N     -3.8904756683     4.5799356198     7.6957753640
O     -5.0127685926     4.7445767044     7.1894456135
O     -2.9206135148     4.2904470414     6.9756492104
O     -3.7380447748     4.7047832142     8.9222322553
N    -11.8938041736     0.5042492033     3.3137493110
O    -11.9557333630     1.6701195953     3.7379295976
O    -12.8627369955    -0.2601886559     3.4545394274
O    -10.8629413323     0.1028163474     2.7487784532
N     -5.4558204663    -0.6426132898    13.1841673440
O     -6.5855615887    -0.2747524551    13.5466329471
O     -4.4437167009    -0.1242896579    13.6841718357
O     -5.3381830144    -1.5287984698    12.3216965548
N     -3.9785058636    -5.1185412168     5.3876293426
O     -3.2198361281    -5.8057158748     4.6839041599
O     -3.5685142329    -4.0602526424     5.8925551459
O     -5.1471681706    -5.4896554320     5.5864288820
N     -3.4024570094    -2.0592590679     8.7367060228
O     -4.0937195485    -3.0801744659     8.5853595226
O     -2.6320660978    -1.6914197293     7.8343701789
O     -3.4815854457    -1.4061824827     9.7903892152
N     -7.4332472898    -0.7905547925     7.1594763901
O     -8.1248309815     0.2096171171     7.4132204727
O     -6.2435605105    -0.8294149307     7.5146568624
O     -7.9313507782    -1.7518673378     6.5505513450
N    -14.1184831293    -4.5094989008     3.4359461170
O    -14.7880107033    -3.5253653021     3.0806549709
O    -14.2981239679    -5.0104507840     4.5583516991
O    -13.2693140331    -4.9926810053     2.6688310634
N     -3.8805934072     2.4750126544    11.9805097871
O     -2.6421240943     2.5290751628    12.0598124794
O     -4.5782424860     3.3127463637    12.5759137727
O     -4.4214140768     1.5832157186    11.3058025660
N    -13.3392741554     1.0066957814     0.5927377766
O    -12.8504107288     0.0410757761    -0.0168591939
O    -14.4808816905     0.9231363343     1.0751981018
O    -12.6865295213     2.0558760784     0.7198745242
N     -5.4766232929    -3.0672967977     3.6342247812
O     -4.3525844450    -2.6689910713     3.2865077585
O     -6.0316332984    -3.9850646399     3.0075797520
O     -6.0456525932    -2.5478342638     4.6085876174
N     -6.7843888051    -0.4794877790     1.2289998893
O     -7.8079484092    -0.0152153625     1.7579561928
O     -6.5885235279    -1.7061280216     1.2260982600
O     -5.9566938119     0.2828806607     0.7029447916
N     -3.2053887921    -4.1153388270     0.7588394459
O     -2.5013020501    -4.5248452338     1.6967009886
O     -4.1771484070    -4.7859848007     0.3729182885
O     -2.9377157038    -3.0351855769     0.2068986161
N     -7.7720994076    -3.0743990909     9.7133135711
O     -7.8512560148    -4.2323121954     9.2706066876
O     -8.7697351782    -2.3344419660     9.6990225383
O     -6.6953061631    -2.6564427747    10.1703118552
N    -11.3959956427    -1.1007806586    11.7712102016
O    -10.8430370231    -0.7256552858    12.8183661740
O    -12.2472097105    -0.3792499617    11.2254575331
O    -11.0977399543    -2.1974376113    11.2698064939
N     -7.7693361438    -7.0456488790     2.4890986600
O     -6.8889194846    -7.6146566211     3.1555179024
O     -8.9683267555    -7.2424026556     2.7474150850
O     -7.4507619347    -6.2798867439     1.5643622483
N     -8.7331421277     3.7570651574    -0.3348238081
O     -7.5574979951     3.9553011853    -0.6835088547
O     -9.2002838080     2.6061884395    -0.3515373276
O     -9.4416451504     4.7097066142     0.0305750521
N     -8.6254714205     3.8883005548    12.9428817457
O     -9.6025795723     4.4891877332    12.4662139688
O     -7.4729807845     4.2304490328    12.6302834990
O     -8.8008540459     2.9452641391    13.7321484047
N    -10.4181343278    -7.6662475183     7.5106995925
O    -11.3614864248    -7.6915288116     6.7029461721
O    -10.5207214104    -7.0180141652     8.5653499237
O     -9.3721943061    -8.2892000797     7.2638024828
N    -11.1450316939     2.2746246783     7.5703872746
O    -11.8757952417     3.1144163464     8.1215247368
O    -10.2404103430     2.6458633609     6.8043194166
O    -11.3188896370     1.0635933526     7.7853178432
N     -3.9495877050     1.4719474871     7.7437306535
O     -2.8215809725     1.2066787624     8.1912250401
O     -4.8169632390     1.9532726592     8.4913961352
O     -4.2102191134     1.2558908656     6.5485698230
N     -9.0981699630     6.8682954826     3.6211079803
O     -9.9530032687     5.9965600587     3.3922952160
O     -8.6924481297     7.5970755214     2.7006258459
O     -8.6490581290     7.0112509827     4.7704038043
N     -8.4816691339     5.5068997436     8.3230594708
O     -7.9441618586     4.8185732131     7.4397076567
O     -9.6247504012     5.9603215675     8.1475836122
O     -7.8760946544     5.7418046393     9.3818879957
N     -5.9182726393     1.1627004634     4.2140046095
O     -5.0533626382     0.5567186430     3.5599903634
O     -6.9472580367     0.5713575991     4.5807750826
O     -5.7541971111     2.3600261118     4.5012486139
N     -8.3566192833     3.4889813617     3.5300445650
O     -9.2118613825     2.7403885892     3.0288582487
O     -7.5657056732     4.1063251870     2.7976784177
O     -8.2922907425     3.6202304146     4.7635980217
N     -4.9415521595    -6.8044216687     8.3101324718
O     -5.8788873904    -7.4990187915     7.8835701517
O     -5.1751375184    -5.8262382293     9.0392503977
O     -3.7706306272    -7.0880082137     8.0075766224
N    -11.2857206923    -2.2970185322     4.8819843277
O    -10.4484774961    -1.4825908126     5.3047717812
O    -12.2115995759    -2.6750015223     5.6187984583
O    -11.1970849337    -2.7334636130     3.7223818099
N      0.0375242732    -3.2985345157     6.7692871081
O      0.1747170709    -2.3361126090     5.9960212284
O     -0.4722892061    -4.3552669140     6.3613351925
O      0.4101452548    -3.2042239480     7.9505058543
N    -13.6513892287     3.5957717919    10.5231331183
O    -13.4471555945     4.7089592180    10.0111562750
O    -14.5179067784     2.8431645708    10.0480040645
O    -12.9891047800     3.2351912967    11.5102398101
N    -13.5791416701     4.8376188079     4.6636178373
O    -14.7635885955     4.4709711782     4.5882991325
O    -13.1431992310     5.3157156807     5.7239929978
O    -12.8306365810     4.7261694750     3.6785605885
N     -9.7995192684    -3.0041507681     0.7825172692
O     -9.2701858234    -2.2163003514    -0.0188039747
O    -11.0181603275    -3.2347575188     0.7135795681
O     -9.1102110995    -3.5613948825     1.6527769147
N      0.4010677679     0.6878556583     9.8978121071
O      0.3006823445     1.4029588977     8.8870856185
O      1.3422926684    -0.1164834228     9.9985704318
O     -0.4397723862     0.7770915719    10.8077810035
     360
n= OPLS/AA
N      1.8954295727    -0.9614029704    -3.4195231142
H      2.1168596953    -1.7110316034    -2.7537440885
H      2.2859787485    -1.1947624210    -4.3399846254
H      2.3024717492    -0.0786833282    -3.0888295645
H      0.8764083134    -0.8611352592    -3.4955335301
N     -3.5016035788     2.1880800672   -10.7405540801
H     -3.6769866471     2.5063423307   -11.7008592443
H     -4.3877891335     2.1575498796   -10.2228884006
H     -2.8565858862     2.8385787792   -10.2768309665
H     -3.0850528191     1.2498495892   -10.7616386441
N     -6.0734559363    -1.9911071409     0.1856189953
H     -6.1839146457    -1.1831534471     0.8095146596
H     -6.9488707507    -2.5271131036     0.1615109845
H     -5.3148868553    -2.5899944321     0.5322347564
```

H	-5.8461516012	-1.6641667938	-0.7607838115
N	-6.2113714271	-2.4721456247	-8.1351192036
H	-6.7006294268	-1.6249947149	-7.8233531963
H	-6.6111903102	-2.7900888495	-9.0257884193
H	-6.3196398688	-3.2094355903	-7.4287786517
H	-5.2140265789	-2.2640625191	-8.2625562433
N	-10.5961745050	-2.2132634009	-3.9797100223
H	-9.9558283477	-2.8197321861	-4.5054366707
H	-11.5519840907	-2.3357280900	-4.3342027885
H	-10.3114132151	-1.2335026894	-4.0946686242
H	-10.5654717429	-2.4640912286	-2.9845325179
N	2.3466397467	1.7383833420	0.4051083286
H	2.7322810341	2.6283835527	0.7418820212
H	2.5877627505	1.6137610507	-0.5851258190
H	1.3253129832	1.7462811899	0.5102976356
H	2.7412025945	0.9651084414	0.9533798046
N	-7.7303628630	6.5105472586	1.5430874367
H	-7.9374614856	7.4556855990	1.1994891604
H	-7.2480171669	6.5731603802	2.4473312710
H	-7.1289939564	6.0248353733	0.8673101607
H	-8.6069790446	5.9885086023	1.6582188200
N	-11.0149592187	-4.3776788383	-8.0944845797
H	-11.5237405212	-3.7437030512	-7.4672267499
H	-11.6557708482	-5.0991576565	-8.4452885932
H	-10.2460401416	-4.8234992327	-7.5804387130
H	-10.6342858592	-3.8443547956	-8.8849836517
N	-4.6822596170	0.0189254095	-5.7294557032
H	-5.2309553946	0.2827423732	-6.5562384140
H	-4.5409874895	-0.9980460565	-5.7226801066
H	-5.1862295527	0.3001218447	-4.8802334750
H	-3.7708665654	0.4908837337	-5.7586716224
N	-6.7091578734	-0.7626926969	-12.0931507751
H	-7.6107839800	-0.9956549613	-11.6606752905
H	-6.6330194571	-1.2352376120	-13.0015223567
H	-5.9455536997	-1.0729653572	-11.4808803558
H	-6.6472752348	0.2530869162	-12.2295246762
N	0.5704416632	-2.1491273479	0.5218363546
H	1.1961930814	-1.9105062116	1.3001237138
H	0.9688608350	-2.9318135117	-0.0100463561
H	0.4651013138	-1.3348282089	-0.0946503420
H	-0.3483879681	-2.4193612269	0.8919191608
N	-11.2210405832	1.7084170846	-5.8316950562
H	-11.5522075680	0.7379318307	-5.8838764481
H	-11.6759159485	2.2651521850	-6.5647497032
H	-10.2034664515	1.7305518625	-5.9669298969
H	-11.4525726874	2.1000315152	-4.9112242275
N	-5.6373960509	6.2525558566	-12.5564452023
H	-6.1370152950	6.7649627237	-11.8202007382
H	-5.7014834957	6.7728066978	-13.4393192290
H	-6.0601843005	5.3243808974	-12.6746689951
H	-4.6509015989	6.1480736064	-12.2915911299
N	-4.4196779365	8.1791018542	0.2365723128
H	-4.7109241369	8.1947502697	1.2210350472
H	-3.4250551373	8.4251149377	0.1699559831
H	-4.9765226121	8.8577917243	-0.2959120454
H	-4.5662101435	7.2387505005	-0.1487887749
N	-1.1027810048	6.2526545402	1.6956101600
H	-1.2288165445	7.0911061538	1.1165160122
H	-1.9096616326	6.1443715696	2.3212723181
H	-0.2467467674	6.3481945251	2.2544598891
H	-1.0258991974	5.4269467289	1.0901918565
N	-9.0003786643	0.4534400541	0.0459314102
H	-9.8766061849	0.6784780244	-0.4396809267
H	-8.8814933501	1.0841735766	0.8473527670
H	-8.2112471475	0.5691572186	-0.6006878836
H	-9.0321688281	-0.5180483839	0.3767412110
N	-8.5469853547	7.4407384714	-3.2901890873
H	-7.8466395556	7.4857903781	-4.0396703645
H	-9.4602385349	7.1942743597	-3.6895119592
H	-8.6115085157	8.3546451414	-2.8266733009
H	-8.2695541307	6.7282440502	-2.6049014544
N	-4.5118239073	8.6940980503	-3.3728691188
H	-5.2453716609	9.1461591190	-2.8144923814
H	-4.4720693013	9.1290311959	-4.3021093544
H	-4.7240792585	7.6943897487	-3.4716947313
H	-3.6057761228	8.8068125777	-2.9031794642
N	-7.9670446747	3.2988597221	-10.4405082618
H	-8.9044119815	2.9569679070	-10.1982509264
H	-7.9161450792	4.3096740241	-10.2675968295
H	-7.7802167083	3.1115072699	-11.4325918741
H	-7.2674058429	2.8172893543	-9.8635931815
N	-6.2581638975	3.1467321457	3.9013426501
H	-6.2811920254	2.8708619896	4.8900799559
H	-5.8130213735	2.4043233984	3.3491542123
H	-5.7215998001	4.0163752516	3.8010662230
H	-7.2168424133	3.2953676743	3.5650711719
N	1.0789631840	2.4616862369	-6.5507118997
H	0.0876369442	2.4617502049	-6.2833013917
H	1.3942965150	1.4953532964	-6.6956264393
H	1.6349065207	2.8947750429	-5.8039879771
H	1.1990117906	2.9948664036	-7.4199315301
N	-5.0004888855	7.9658404325	-7.9502893740
H	-4.2196691289	7.5286609144	-8.4537083588
H	-5.5084847179	7.2495946428	-7.4181550974
H	-5.6372463905	8.4077972547	-8.6236760173
H	-4.6365545444	8.6773084925	-7.3056185130
N	-4.2176938280	3.9686829669	0.9985682840
H	-5.1695943298	3.6494890739	0.7835408651
H	-4.1726732967	4.2761009833	1.9771907855
H	-3.9719694740	4.7521271762	0.3820699166
H	-3.5565391385	3.1970143234	0.8514713593
N	-4.6796044852	3.9433999054	-7.7562154265
H	-4.1676218168	3.0662259973	-7.6056306333
H	-4.3295192980	4.6594658095	-7.1089715249
H	-5.6804545575	3.7889161622	-7.5868949320
H	-4.5408217697	4.2589907982	-8.7233644689
N	1.2074879079	5.5647656471	-1.5319794437
H	1.6058097166	6.1357714934	-2.2866503375
H	0.1834453078	5.6365210055	-1.5525368567
H	1.5561221911	5.9021267721	-0.6270611424
H	1.4845748040	4.5846438737	-1.6616701732
N	-9.9827524591	-0.1914898811	-8.9774235659
H	-10.8389034191	-0.6478682228	-8.6413276860
H	-9.6825893961	-0.6311576926	-9.8553922650
H	-9.2421605672	-0.2923792955	-8.2734487795
H	-10.1673572878	0.8054452420	-9.1395252057
N	-0.0327734480	-3.9115105554	-5.7817853564
H	-1.0396097299	-3.7589771691	-5.6504447110
H	0.1190822983	-4.8166997947	-6.2420102566
H	0.4357828714	-3.9091927088	-4.8681743192
H	0.3536497876	-3.1611724005	-6.3665120107
N	-6.6129332186	-3.0893096324	-3.8997074245
H	-7.0955585940	-3.1894996003	-2.9990016930
H	-5.6050468355	-3.2322912205	-3.7657074634
H	-6.7782639226	-2.1474800288	-4.2736837342
H	-6.9728639924	-3.7879677775	-4.5604359304
N	-2.1654793023	0.3189745829	2.4430306827
H	-2.4064152758	0.9168865169	3.2422098075
H	-1.2290904508	-0.0783365174	2.5828680661
H	-2.1748680490	0.8782852291	1.5820318578
H	-2.8515436683	-0.4409363148	2.3650137780
N	-9.0094997980	5.5500430778	-7.4337586665
H	-9.7289163378	5.1983077105	-8.0763768823
H	-8.2732117866	4.8428689238	-7.3241257990
H	-9.4337632367	5.7469398759	-6.5197194389
H	-8.6021085317	6.4120554584	-7.8148131715
N	-1.0645776184	6.0351039205	-7.6228348161
H	-1.8562505424	5.7290018604	-7.0450878059
H	-1.3420739575	6.8530772322	-8.1779475332
H	-0.7852738942	5.2732719061	-8.2519905485
H	-0.2747128506	6.2850643852	-7.0163128143
N	-0.6653681316	3.3974175845	-3.5026025221
H	-1.1190828313	4.1830974839	-3.0218882986
H	-1.1904984155	3.1681955735	-4.3546178624
H	-0.6476236832	2.5802132411	-2.8812349017
H	0.2957319615	3.6581648049	-3.7526685575
N	-2.4819980575	-2.7592631444	-1.9493600081
H	-2.9698216128	-2.9090421140	-1.0583883269
H	-3.0509239930	-3.1329188545	-2.7180858212
H	-1.5757087715	-3.2413884390	-1.9286126130
H	-2.3315383279	-1.7537033159	-2.0923524036
N	0.6917076514	3.0531851079	4.1453745286
H	0.9823347559	3.2344018603	5.1133272508
H	-0.3245733249	2.9102246054	4.1142303889
H	1.1664908781	2.2119746480	3.7972633768
H	0.9425785796	3.8561394942	3.5566780407
N	-8.2796273685	1.8289513436	-3.8146762264
H	-7.8530049193	1.9070943742	-2.8840189048
H	-7.5420095097	1.8075670507	-4.5286070997
H	-8.8920166171	2.6364768022	-3.9793503467
H	-8.8314780124	0.9646672234	-3.8667276480
N	-3.4936366954	1.4131984577	-1.7265146127
H	-3.9611159314	1.1626775300	-0.8473453876
H	-2.5699455727	0.9662755961	-1.7624221479
H	-3.3827444314	2.4327156403	-1.7767455213
H	-4.0607413015	1.0911248204	-2.5195445377
N	-10.1467038264	3.9053250070	-0.9513763695
H	-10.2241083963	4.3545859545	-0.0313713525
H	-9.1866550813	3.5677618045	-1.0877295019
H	-10.3777511687	4.5855556280	-1.6849559166
H	-10.7983007348	3.1133970786	-1.0014478108
N	-0.5236096669	7.9933471082	-3.9510857062
H	-0.5163667238	8.1273459794	-2.9331328881
H	0.4419866073	7.9503063354	-4.2975045460
H	-1.0122275332	8.7790429374	-4.3962487874
H	-1.0078310110	7.1166933112	-4.1774556119

N	-1.3559675351	-0.3940898572	-8.0064064096
H	-1.1428971485	0.5057463326	-8.4526484286
H	-0.5322322949	-1.0049121682	-8.0573087084
H	-2.1429080438	-0.8394900994	-8.4928000610
H	-1.6058324456	-0.2377026175	-7.0228688751
N	-5.1630416710	4.1189879782	-3.7119721259
H	-6.0316335483	4.3377056724	-4.2139151691
H	-4.6110176607	4.9765740357	-3.5934380318
H	-4.6198180403	3.4339680269	-4.2503876508
H	-5.3896982807	3.7277043909	-2.7901481407
N	1.9035188980	5.1087582512	1.7080978966
O	1.8174265107	6.0498321053	0.9018807962
O	2.6074064759	4.1216342311	1.4376420634
O	1.2857232100	5.1548084541	2.7847716969
N	-1.0010823322	2.7969248788	0.7981720330
O	-2.1676672684	2.3839743776	0.6906109750
O	-0.7238704585	3.9650932819	0.4794965398
O	-0.1117085538	2.0417063690	1.2244089274
N	-2.6366989175	2.4233388066	-5.9009668797
O	-1.4282729681	2.7108449904	-5.8929151728
O	-3.3238229115	2.5993973450	-4.8812208727
O	-3.1580012926	1.9597737112	-6.9287654210
N	-1.7322153768	-0.6868827285	-4.3208741780
O	-0.6800752619	-1.2228332356	-3.9351590714
O	-2.6772929128	-0.5331615155	-3.5295359756
O	-1.8392780419	-0.3046531267	-5.4979284345
N	-3.6544365092	-3.2256864440	-5.0624742470
O	-2.5392684226	-3.5780978699	-5.4810721884
O	-4.0136155116	-3.5364141219	-3.9146690957
O	-4.4104262019	-2.5625468064	-5.7916820438
N	-6.7662699488	3.0227516227	-0.7667864571
O	-7.8526372100	2.7468395692	-1.3022270453
O	-5.7275204685	3.0499064236	-1.4474332413
O	-6.7186521296	3.2715090755	0.4493018943
N	-4.8759622338	6.5284424070	2.7418325198
O	-3.6796605775	6.3430322577	2.4634383128
O	-5.5781654211	5.5792556142	3.1278027609
O	-5.3700611006	7.6630402625	2.6342563992
N	-2.4764644450	5.0676880152	-1.4907247168
O	-1.3881715833	5.4481261707	-1.9532216010
O	-3.0478445596	5.7385636660	-0.6152428285
O	-2.9933776083	4.0163733626	-1.9037100535
N	-7.2170187606	9.2355938048	-1.0153217262
O	-6.0520096152	9.4961132758	-1.3586891785
O	-8.1615338960	9.4878859759	-1.7816491565
O	-7.4375129482	8.7227817500	0.0943740497
N	-6.6389793189	6.2649554632	-5.5548930859
O	-7.1279031609	5.3330671794	-4.8949267124
O	-6.6851692014	7.4267306964	-5.1176715023
O	-6.1038651636	6.0350683287	-6.6520819264
N	-1.5167729518	8.3930904610	-0.9790734736
O	-0.3767583647	8.0938446271	-1.3712935430
O	-1.7887903436	8.3475047578	0.2321021508
O	-2.3847708459	8.7379222758	-1.7980296878
N	-6.0648012915	-0.1194254351	-2.4634731567
O	-5.4107231520	-1.1395190259	-2.1903271073
O	-7.1490868308	0.0935861289	-1.8960501629
O	-5.6345935454	0.6876572415	-3.3040428765
N	-7.2611580945	6.9578898585	-9.7814253522
O	-7.6533531359	5.8507983904	-10.1858378170
O	-7.6957031202	7.4183077519	-8.7126859569
O	-6.4344173619	7.6045639538	-10.4457528174
N	-6.4594511580	6.1129917564	-1.3127959008
O	-5.8461233714	6.5473423532	-2.3018290126
O	-7.6674016085	5.8376958015	-1.4027167407
O	-5.8648280155	5.9539369864	-0.2338410806
N	2.1344797939	2.0802381570	-2.7427262440
O	1.8152905546	3.1189391691	-2.1408655059
O	1.7216250933	1.8794045517	-3.8969509925
O	2.8665243232	1.2423700759	-2.1903617889
N	-0.5847576278	0.3014006181	-1.2792418128
O	0.5760257583	-0.0557693093	-1.0184171889
O	-0.7825002351	1.3187209209	-1.9640594336
O	-1.5477991818	-0.3587502887	-0.8552484745
N	-2.8460218650	9.1606727525	-5.9784147527
O	-2.3568264117	8.4750836320	-6.8914718579
O	-4.0683370424	9.3805331643	-5.9534164075
O	-2.1129015506	9.6264018360	-5.0903552779
N	1.3389939811	5.8087467818	-4.9227423731
O	1.7185854354	6.4840431979	-3.9517104534
O	0.9360061867	6.3740067090	-5.9528394380
O	1.3623903402	4.5681894397	-4.8636771805
N	-9.2495121150	3.7327818705	2.0622961615
O	-9.6928604241	4.7433091591	1.4919499625
O	-8.5825358886	3.8496964211	3.1036854317
O	-9.4731402123	2.6053391236	1.5912527111
N	-7.5512796094	2.7113507196	-6.6965036444
O	-7.2848957796	3.5278098512	-7.5939734503
O	-6.6788814927	2.3852899793	-5.8745402446
O	-8.6900624727	2.2209519336	-6.6209971773
N	-3.0800094795	-2.4948456145	1.2037802983
O	-1.8445569254	-2.5950533494	1.2852254174
O	-3.6978584667	-1.7671484986	1.9985994270
O	-3.6976135435	-3.1223355008	0.3275153450
N	-2.3824516912	3.4668247209	3.8280480615
O	-1.6395046785	4.4564752707	3.7201618411
O	-3.6009379419	3.5786534113	3.6140305112
O	-1.9069120704	2.3653445939	4.1499520913
N	-5.4171912749	0.7662722295	1.4634044905
O	-4.7014806347	0.7008951877	0.4502401244
O	-6.5208097273	0.1964507284	1.4820506510
O	-5.0292831505	1.4014712839	2.4579234968
N	-0.8913667277	3.1292893658	-8.9536362437
O	-0.7619468140	1.8967310438	-9.0377099006
O	-1.9832811955	3.6586874753	-9.2191138279
O	0.0711286012	3.8324501443	-8.6040847213
N	-11.0253908939	1.2116503742	-2.4467901633
O	-10.3297174576	0.2717425945	-2.8658721917
O	-11.6701633918	1.9113270912	-3.2453756806
O	-11.0762918732	1.4518816304	-1.2291216372
N	-3.7078922317	5.8482084501	-10.2675364325
O	-3.3276999276	6.2159294834	-11.3914817001
O	-4.4727820256	4.8755961702	-10.1580518800
O	-3.3231944323	6.4531001835	-9.2530749008
N	-2.8815526917	5.8380966523	-5.0643951156
O	-3.2622427831	5.4517841484	-6.1819172024
O	-3.7092814859	6.2475367664	-4.2335872273
O	-1.6731328332	5.8149690235	-4.7776806862
N	-5.9472206929	2.8726315921	-12.7385813111
O	-6.4638235521	1.7729049761	-12.4802389670
O	-4.7230304590	2.9534040805	-12.9331371831
O	-6.6548086372	3.8915865401	-12.8023678345
N	-12.0341290585	-1.7293121848	-6.7344220373
O	-12.1067686076	-0.7240459394	-6.0083496010
O	-11.8899940292	-1.6001335818	-7.9614329960
O	-12.1056245965	-2.8637579465	-6.2334831116
N	-8.3503490433	-4.3902605144	-6.1508343291
O	-7.1332908470	-4.1948747669	-6.3044952142
O	-8.9449423106	-5.2052073808	-6.8756366209
O	-8.9728144733	-3.7706988966	-5.2723704450
N	0.9102240184	-3.6286034864	-2.3059559698
O	1.8309434058	-3.0182808665	-1.7378010435
O	-0.1521161024	-3.8521060570	-1.7022192891
O	1.0518448657	-4.0154238470	-3.4778485202
N	-10.8157207047	3.1517970075	-8.9578889207
O	-11.6751224115	2.8228352822	-8.1234844621
O	-10.5203741493	4.3492021712	-9.1062438903
O	-10.2516650992	2.2833528699	-9.6439389621
N	-8.2660567391	-0.7493350070	-5.9233999334
O	-7.2366140180	-0.9594180596	-5.2607305545
O	-9.2714739527	-0.2697545598	-5.3737120834
O	-8.2900822662	-1.0188326185	-7.1357581382
N	1.4360541538	-1.2604164407	-6.7574932822
O	2.1611325418	-1.7597447131	-5.8811654220
O	1.4888020814	-0.0406695294	-6.9865195677
O	0.6582272120	-1.9808356596	-7.4047953780
N	-8.8902863280	-2.7189588714	-1.0427503874
O	-8.7475168841	-2.2042472282	0.0787248558
O	-10.0212283342	-2.7980299149	-1.5504273119
O	-7.9021129702	-3.1545998217	-1.6565492004
N	-6.3034551051	0.9306198143	-9.0383339217
O	-7.3226215080	0.4814667157	-9.5884059610
O	-5.7615644017	1.9579871163	-9.4786619849
O	-5.8261790216	0.3524051453	-8.0479330220
N	1.4446537897	-0.0654897568	2.7291166376
O	0.2971635280	-0.5208887832	2.8665784025
O	1.8194542857	0.8938543509	3.4235238540
O	2.2173441775	-0.5694352437	1.8972469866
N	-8.7908864428	-2.5534980457	-10.4439994694
O	-7.6317514475	-2.9762097795	-10.2999898727
O	-9.7454019008	-3.1783634917	-9.9526123249
O	-8.9955061447	-1.5059200227	-11.0793967220
N	-10.1039708671	5.0374578142	-4.1493188746
O	-10.4361595670	5.4114378621	-3.0123019432
O	-10.0406425639	5.8584257564	-5.0793828911
O	-9.8351102540	3.8425088620	-4.3562719562
N	-3.7971445126	-1.1328458446	-9.8781456293
O	-3.6531796177	-1.6334780369	-8.7504668506
O	-4.7775817042	-1.4431599794	-10.5748995042
O	-2.9606715426	-0.3218988647	-10.3090708801
